# Clonal Evolution and Timing of Metastatic Colorectal Cancer

**DOI:** 10.3390/cancers12102938

**Published:** 2020-10-12

**Authors:** Sarah Siraj, Tariq Masoodi, Abdul K. Siraj, Saud Azam, Zeeshan Qadri, Saeeda O. Ahmed, Wafaa N. AlBalawy, Khadija A. Al-Obaisi, Sandeep K. Parvathareddy, Hadeel M. AlManea, Hussah F. AlHussaini, Alaa Abduljabbar, Samar Alhomoud, Fouad H. Al-Dayel, Fowzan S. Alkuraya, Khawla S. Al-Kuraya

**Affiliations:** 1 Human Cancer Genomic Research, King Faisal Specialist Hospital and Research Centre, Riyadh 11211, Saudi Arabia; sarah-siraj@kfshrc.edu.sa (S.S.); TMASOODI@kfshrc.edu.sa (T.M.); asiraj@kfshrc.edu.sa (A.K.S.); saazam@kfshrc.edu.sa (S.A.); sqadri96@kfshrc.edu.sa (Z.Q.); ahmsaeeda@kfshrc.edu.sa (S.O.A.); wafa.n23@gmail.com (W.N.A.); kalobaisi@kfshrc.edu.sa (K.A.A.-O.); psandeepkumar@kfshrc.edu.sa (S.K.P.); 2 Department of Pathology and Laboratory Medicine, King Faisal Specialist Hospital and Research Centre, Riyadh 11211, Saudi Arabia; halmanea@kfshrc.edu.sa (H.M.A.); halhussaini@kfshrc.edu.sa (H.F.A.); dayelf@kfshrc.edu.sa (F.H.A.-D.); 3 Department of Surgery, King Faisal Specialist Hospital and Research Centre, Riyadh 11211, Saudi Arabia; aabduljabbar@kfshrc.edu.sa (A.A.); shomoud@kfshrc.edu.sa (S.A.); 4 Department of Genetics, King Faisal Specialist Hospital and Research Centre, Riyadh 11211, Saudi Arabia; FAlKuraya@kfshrc.edu.sa; 5 Department of Anatomy and Cell Biology, College of Medicine, Alfaisal University, Riyadh 11533, Saudi Arabia

**Keywords:** clonal evolution, colorectal cancer, metastasis, tumour heterogeneity, clonal spread, timing, treatment

## Abstract

**Simple Summary:**

Half of all colorectal cancer (CRC) patients develop metastasis, despite current management. The aim of this study was to help guide precision medicine in metastatic CRC patients, by performing genomic characterisation of primary CRC and metastatic tumours, and revealing the effects of therapy on the metastatic process. We confirmed common ancestry between paired primary CRC and metastatic tumours, with most metastases seemingly having disseminated late (after acquiring most genomic diversity) from their corresponding primary tumour, via either a single clone (monoclonal spread) or multiple clones (polyclonal spread). Treatment prompted the selection for distinct resistant clones, through monoclonal seeding to distant metastatic sites. Overall, this study supports the importance of early clinical detection and surgical removal of the CRC tumour, whilst further highlighting the challenges for treating and managing metastatic CRC with increased intratumour heterogeneity (either due to early dissemination or metastatic spread through multiple clones) and the underlying risk of future therapeutic resistance in treated patients.

**Abstract:**

Colorectal cancer (CRC) is the third most frequently diagnosed cancer worldwide, where ~50% of patients develop metastasis, despite current improved management. Genomic characterisation of metastatic CRC, and elucidating the effects of therapy on the metastatic process, are essential to help guide precision medicine. Multi-region whole-exome sequencing was performed on 191 sampled tumour regions of patient-matched therapy-naïve and treated CRC primary tumours (*n* = 92 tumour regions) and metastases (*n* = 99 tumour regions), in 30 patients. Somatic variants were analysed to define the origin, composition, and timing of seeding in the metastatic progression of therapy-naïve and treated metastatic CRC. High concordance, with few genomic differences, was observed between primary CRC and metastases. Most cases supported a late dissemination model, via either monoclonal or polyclonal seeding. Polyclonal seeding appeared more common in therapy-naïve metastases than in treated metastases. Whereby, treatment prompted for the selection of distinct resistant clones, through monoclonal seeding to distant metastatic sites. Overall, this study reinforces the importance of early clinical detection and surgical excision of the CRC tumour, whilst further highlighting the clinical challenges for metastatic CRC with increased intratumour heterogeneity (either due to early dissemination or polyclonal metastatic spread) and the underlying risk of future therapeutic resistance in treated patients.

## 1. Introduction

Colorectal cancer (CRC) is the third most diagnosed cancer worldwide, and one of the top most in Saudi Arabia, with a distinctly earlier (≤10 years) disease onset compared to other ethnicities [1,2]. High mortality in CRC has been attributed to metastatic disease [1,2,3], whereby half of all patients develop metastasis, despite the improved management of metastatic CRC in recent years [4].

The evolutionary process of CRC is well documented. Whereby, a gradual multi-step model has been used to describe the transformation of intestinal epithelium into invasive adenocarcinoma, from which disseminating tumour cells can then migrate directly, or via haematogenous or lymphogenous spread and colonise regional or distant organs [5], such as the liver, lung, and peritoneum and less frequently the bone and brain [6].

Predominantly, seeding originates from the primary tumour and is spread via a single clone (monoclonal seeding) [7] or multiple subclones (either synchronous or asynchronous polyclonal seeding) to a metastatic site [8,9,10]. Recent studies have also shown metastatic cross-seeding, where one metastasis can seed secondary metastatic sites, via metastatic cascading [8,11].

Several studies have traditionally defined the process of CRC metastasis through a linear progression or late dissemination model, where metastatic divergence occurs late in the primary tumour relative to tumorigenesis [11,12,13,14]. However, few recent studies have challenged this model, suggesting that metastatic seeding might occur early [15,16], before clinical detectability (<0.01 cm^3^) and years before diagnosis or surgery, proposing most CRCs may be “born to be bad” [15].

Understanding the complexity of the metastatic process has been very problematic, even in this advanced genomic era, with difficulties going beyond the procurement of patient-matched paired primary tumours and their distant metastases. Hence, it is of high clinical importance to clarify the origin, seeding composition, and timing of spread to help extricate the evolutionary metastatic process in CRC.

We sought to expand upon current knowledge on the metastatic process in CRC, in the Saudi population, and further highlight the effect of therapy. We used whole-exome sequencing (WES) analysis from multiple tumour regions of paired primary CRC and distant metastatic lesions, from 30 patients with metastatic CRC, to define the origin, composition, and timing of seeding in the metastatic progression of therapy-naïve and treated metastatic CRC tumours.

Overall, this study reinforces the importance of early detection and removal of the CRC tumour, whilst further highlighting the clinical challenges for metastatic CRC with increased intratumour heterogeneity (either due to early dissemination or polyclonal metastatic spread) and the underlying risk of future therapeutic resistance in treated patients.

## 2. Results

### 2.1. Genomic Landscape

To explicate the relationship between primary CRC and metastatic tumours, multiregional WES (median coverage of 166×), with further validation using ultra-deep targeted capture sequencing (median coverage of 2691×), was performed on 191 temporally and spatially distinct tumour regions (resected from primary tumours, lymph node metastases, and distant metastases of either the liver, ovary, lung, urinary bladder, prostate, adrenal gland, and spleen) from 30 patients (primary tumour median 4, range 1–5 regions/patient; and metastatic tumour median 4, range 1–7 regions/patient). Three patients (CRC-141, CRC-190, and CRC-293) had additional lymph node metastases (single biopsy) available for study. Tumours were microsatellite stable (MSS) to eliminate the effect of potential underlying germline mutations. Fourteen patients received therapy, prior to surgical excision of either the primary tumour or metastatic lesion (Table 1, Tables S1 and S2).

**Figure 1:**
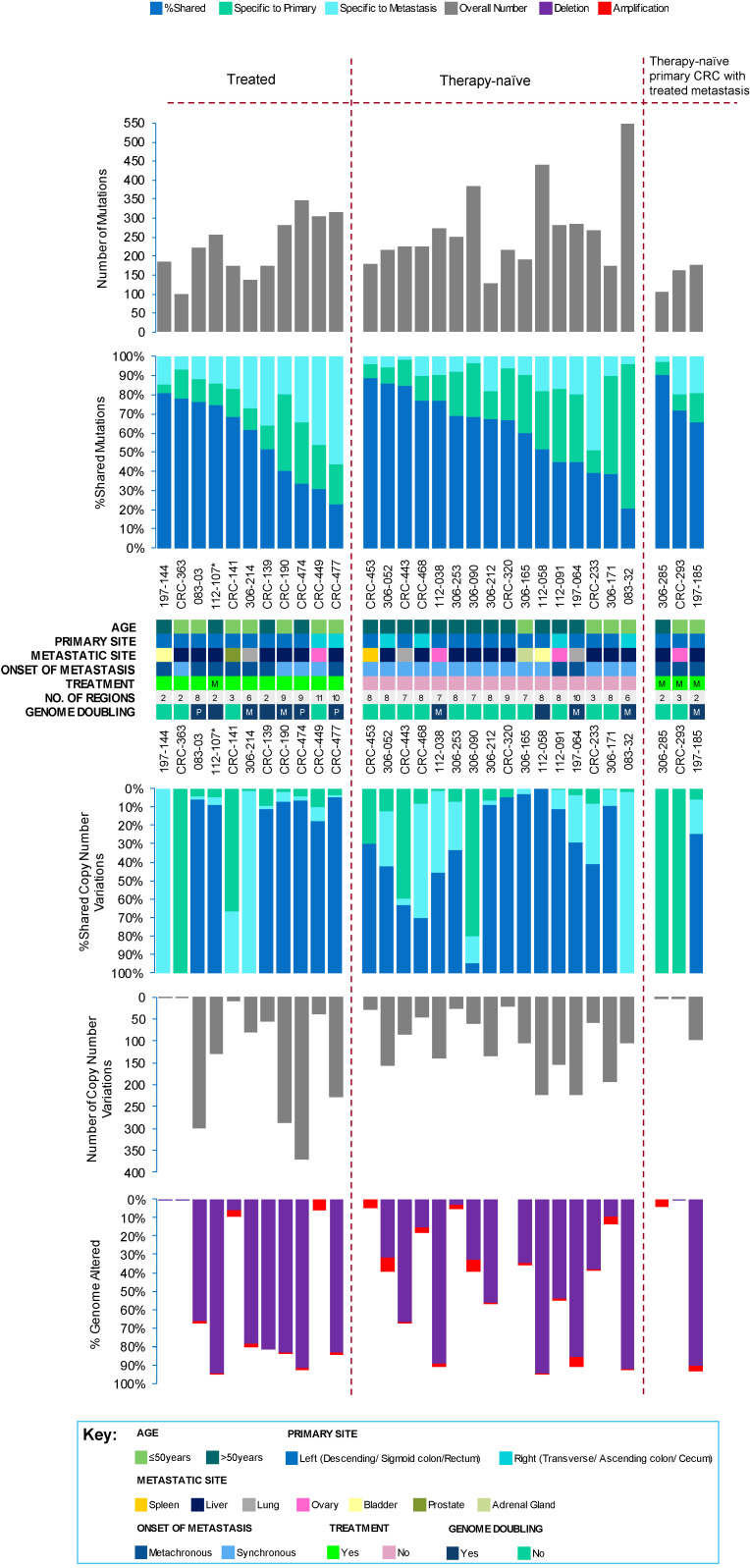
Genetic heterogeneity in the metastatic colorectal cancer (CRC) cohort. The total number of mutations (top panel), and copy number events, with percentage genome altered (bottom panel) detected in all tumour regions sampled per patient (*n* = 30), with clinical details and markers, such as age, primary site, metastatic site, onset of metastasis, treatment status, number of sequenced regions, and genome doubling. The percentage of variants shared between primary and metastasis, specific to primary, or specific to metastasis have been indicated in the middle panel. “P” refers to primary CRC tumour and “M”, refers to metastatic lesion. The treatment status of the primary tumour of patient 112-107, marked by an asterisk (*), was not available.

Direct comparison of the genomic landscape, including mutational (comprising single nucleotide variants, SNVs; and small insertions and deletions, indels) and copy number variation (CNV), between matched primary tumours and metastases revealed overall concordance, with 67% (range 21–90%) of all mutations and 71% (range 0–100%) of all CNVs across all sampled primary-metastasis pairs being shared (Figure 1 and Figure S1; Tables S3 and S4). However, significantly more clonal (median 140, range 74–227) but less subclonal (median 21, range 0–155) mutations were found in metastases compared to matched primary tumours (clonal: median 127, range 60–219; subclonal: median 57, range 0–436) (Benjamini–Hochberg, BH adjusted *p* = 0.001 for clonal and *p* < 0.0001 for subclonal mutations, Wilcoxon’s signed rank test), likely representing an evolutionary bottleneck.

Tumour heterogeneity was further assessed spatially, within tumours (intratumour heterogeneity; accounting for differences amongst multiple spatially distinct primary tumour regions or metastatic tumour regions), and temporally, between tumours (intertumour heterogeneity; accounting for differences between patient-matched primary tumour regions and metastatic tumour regions) in each patient, using Shannon’s diversity index (SDI) [17,18]. Following dissemination, all patients demonstrated significantly more intertumour (median 2.0, range 0.48–3.57) rather than intratumour (primary tumour-specific median 1.5, range 0.48–2.46, and metastatic-specific median 1.2 mutations, range 0.12–3.01) heterogeneity, where the tumours (primary and metastasis) continued to evolve independently after the metastatic seeding event (BH adjusted *p* = 0.030 for primary tumours and *p* = 0.002 for metastatic tumours; Mann–Whitney U test) (Figure S2a). Intertumour heterogeneity, and intratumour heterogeneity amongst metastases, were independent of clinicopathological variables, such as TNM (tumour-node-metastasis) classification and histological grading, where no association was observed (*p* > 0.05; ANOVA test). However, interestingly, less intratumour heterogeneity amongst primary tumours was significantly associated with a higher prevalence of multiple regional lymph node metastases (*p* = 0.0176; ANOVA test). Therefore, increased regional lymph node involvement may not necessarily reflect proportionally increased intratumour heterogeneity in the primary tumour.

Dynamic mutational processes were identified using the 30 published mutational signatures from the COSMIC database (https://cancer.sanger.ac.uk/cosmic/signatures) (Figure S3). Whereby, mutational signature 1, associated with age at diagnosis and mutational signatures related to defective DNA mismatch repair (MMR; 6 and/or 15), were activated consistently, across the cohort. However, upon further stratification according to treatment, mutational signature 3, associated with failure of DNA double-strand break-repair by homologous recombination (HR) and mutational signature 17, were specifically activated late in the progression of treated metastases only. Recently, the COSMIC mutational signature 17 (SBS17) has further been split into SBS17a (of unknown aetiology) and SBS17b (an exogenous signature related to capecitabine and 5- fluorouracil, or 5-FU, chemotherapy, and damage by reactive oxygen species, with significantly more T > G nucleotide changes [19,20]). Treatment, which mainly involved a therapeutic regimen comprising capecitabine and 5-FU chemotherapy, was significantly associated with more T > G nucleotide changes in the current metastatic CRC cohort (treated 3.73% vs. therapy-naïve 2.64%) (BH adjusted *p* = 0.014, Chi squared test). Therefore, the intratumor heterogeneity, post-dissemination, in treated metastases is probably resultant of the collective dysregulation of DNA repair processes, and the capecitabine and 5-FU chemotherapy.

Although CNV patterns were also largely similar between primary and metastatic tumours (Table S4), CNVs were significantly biased towards being deletions, with 90% (range 0–100%) of all CNVs identified as deletions compared to 10% (range 0–100%) of amplifications (BH adjusted *p* < 0.0001; Mann–Whitney U test). Despite treatment, CNVs persisted, as previously reported [21], which may confer resistance to therapy.

Evidence of chromosomal instability, generated through genome doubling (GD) or polyploidy, resulting in additional copies of the entire genome of a tumour cell, was also investigated. A region with mean ploidy ≥3 was considered to have undergone genome doubling. Whereby, 40% (12/30) of cases presented GD events, most of which were restricted to the metastatic lesion (Figure 1 and Figure S1, Table S4), in agreement with previous reporting [22] albeit, this was not statistically significant (*p* > 0.05; Chi-squared test). Although chromosomal instability is characteristically generated in CRC, treatment and the metastatic organ site colonised appeared to play a minimal role on copy number evolution in metastatic CRC.

### 2.2. Driver Events

**Figure 2:**
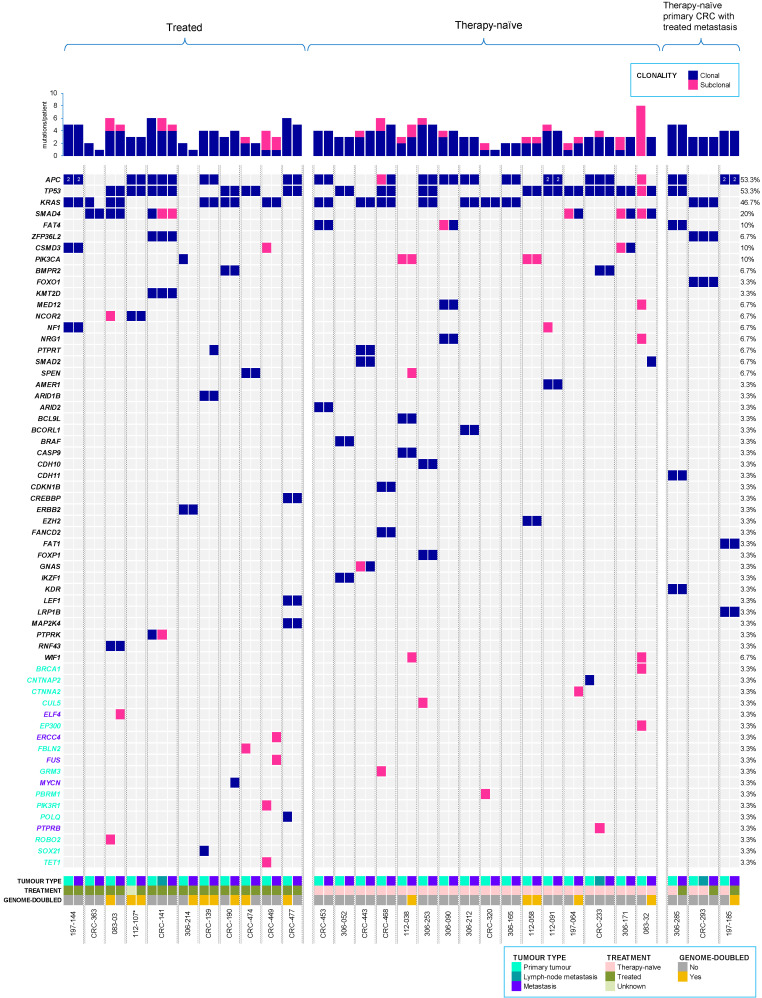
Driver mutations in metastatic colorectal cancer (CRC) cohort. All clonal and subclonal driver mutations (including single nucleotide variants—SNVs, and small insertions and deletions—Indels) detected for each CRC primary and corresponding metastasis, including lymph node metastasis, where relevant. The top panel displays the number of driver mutations/patient identified across tumours, whereas the right panel shows the percentage of driver mutations/gene. Primary-specific (turquoise) and metastasis-specific (purple) driver genes are indicated on the left panel. Treatment status and genome-doubling are also provided. The treatment status of the primary tumour of patient 112-107, marked by an asterisk (*), was not available.

Potential driver events were identified using a list of cancer genes, from the COSMIC cancer gene census (v90), The Cancer Genome Atlas–human colorectal carcinoma (TCGA-CRC), and other large genomic studies, which were then further assessed for functional impact using several prediction tools, as previously described [23] (see Materials and methods, Tables S3 and S4 for further details of ‘driver classification’). The distribution of driver events was further investigated in the multiple tumour regions sampled from each patient. A total of 131 driver mutations were identified, across the metastatic CRC cohort. Where, 94 were clonal (including shared clonal, primary-specific clonal, or metastatic-specific clonal) and 37 were subclonal (including shared subclonal, primary-specific subclonal, or metastatic-specific subclonal) (Figure 2).

All patients harboured driver mutations (median 4, range 2–9). Driver mutations were highly enriched on the trunk (primary tumour/metastasis shared clonal) of the phylogenetic tree (trunk driver mutations 78.2 ± 32.1% vs. branched driver mutations 21.2 ± 32.1%) (BH adjusted *p* < 0.0001, Mann–Whitney U test). However, amongst branched driver mutations, significantly more intertumour heterogeneity (median 25%, range 0–83%) was observed following dissemination, when comparing metastases (median 0%, range 0–38%) (BH adjusted *p* < 0.0001, Mann–Whitney U test) but not primary tumours (median 11%, range 0–100%).

Recurrent driver mutations in the cohort were found encompassing cancer genes with known roles in CRC [24,25], such as *APC* (MIM: 611731; 53.3%, 16/30 cases), *TP53* (MIM: 191170; 53.3%, 16/30 cases), *KRAS* (MIM: 190070; 46.7%, 14/30 cases), *SMAD4* (MIM: 600993; 20%, 6/30 cases), *PIK3CA* (MIM: 171834; 10%, 3/30 cases), and *SMAD2* (MIM: 601366; 6.7%, 2/30 cases), consistent with previous reports [26,27,28,29]. High concordance in driver genes was observed between primary CRC and metastatic tumours, in agreement with previous reporting [30]; of which, 88% of shared (between the primary tumour and corresponding metastatic lesions) driver mutations were clonal.

If not readily clonal in its primary-metastatic pair, most driver mutations became clonal in their corresponding metastatic tumours, likely upon migration due to evolutionary bottlenecking. In contrast, driver mutations in *PIK3CA* were consistently subclonal, in agreement with previous reporting [31]. The pattern of disseminating subclonal driver mutations in the primary tumour, either becoming clonal or remaining subclonal upon seeding/colonising the metastatic site, was only observed in therapy-naïve tumours. Whereas, the pattern of disseminating clonal driver mutations in the primary tumour, becoming subclonal upon seeding/colonising the metastatic site, was only observed in treated tumours; thus re-emphasising the relevance of adjuvant therapy following tumour excision in CRC for effectively eradicating and/or preventing subclones in the primary tumour, from either successful seeding/colonising and/or thriving at the metastatic site.

A clonal driver mutation in *MYCN* (MIM: 164840; CRC-190), and subclonal driver mutations in *ELF4* (MIM: 300775; 083-03), *PTPRB* (MIM: 176882; CRC-233 lymph node metastasis), *FUS* (MIM: 137070), and *ERCC4* (MIM: 133520) in CRC-449 were all specific to metastatic tumours. Excluding one sample (CRC-233), all metastatic-specific driver mutations were exclusive to treated patients. This suggests metastatic-specific driver mutations may confer mechanisms for acquired therapy resistance.

Similarly, several driver copy number amplifications encompassing oncogenes were found specific to metastatic lesions, including *MUC16* (MIM: 606154; CRC-449), *PIK3CA* and *TBL1XR1* (MIM: 608628; 197-064), *MAML2* (MIM: 607537) and *PRKACA* (MIM: 601639; 083-03), *CARD11* (MIM: 607210), *ETV1* (MIM: 600541), *HNRNPA2B1* (MIM: 600124), *HOXA11* (MIM: 142958), *HOXA13* (MIM: 142959), *HOXA9* (MIM: 142956), *MACC1* (MIM: 612646), and *RAC1* (MIM: 602048), all found exclusively in patient 306-090 (Table S4 and Figure S1).

### 2.3. Origin of Metastasis

Phylogenies were clustered spatially and temporally for each CRC tumour, lymph node, where relevant, and distant metastasis, based on mutational cancer cell fractions (CCFs) from both SNVs and Indels (Table S5 and Figure S4). All sampled regions indicated a common clonal ancestry, amongst matched primary-metastasis tumour pairs, whereby metastases were often seeded by major clones in the primary tumour, further supported by the increased driver homogeneity and fewer metastatic-specific driver mutations observed in the cohort.

Notably, distant metastasis-founding subclones were not detected in any primary region sampled from patient CRC-233, which instead underwent a metastatic cascade, from the regional lymph node to the liver. Despite the remaining two cases (CRC-141 and CRC-293) with available lymph node metastases having seemingly metastasised independent of lymphatic involvement to their corresponding distant metastatic sites (prostate and ovary), they may also have indeed spread lymphogenously by an alternative lymph node that was not sampled.

Phylogenetic analysis further revealed mixed seeding patterns, as previously reported in CRC [11,32], including monoclonal seeding (60.6%, 20/33 of all metastases), where only a single clone from the primary tumour/lymph node metastasis was involved in seeding the metastasis, and polyclonal seeding (39.4%, 13/33 cases), where more than one primary tumour/lymph node metastasis subclone contributed to seeding the metastasis. Thus, indicating that multiple subclones may be able to attain metastatic potential and colonise distant metastatic sites through mutual cooperation.

To confirm these patterns, we calculated the RDS (root diversity score) as previously described [33], to quantify the amount of homogeneity amongst the metastases with multiple sampled regions per tumour available (70%, 21/30 of cases) (Figure S2b). As expected, polyclonal metastases had significantly higher RDS values (median 0.21, range 0.01–1), demonstrating higher tumour heterogeneity than monoclonal metastases (median 0.01, range 0–0.44) (BH adjusted *p* = 0.001, Mann–Whitney U test).

Polyclonal seeding was found in lymph node metastases (66.7%, 2/3), and amongst distant metastases (36.7%, 11/30), mostly in liver (63.6%, 7/11), adrenal gland (100%, 1/1), and spleen (100%, 1/1) CRC metastases. In contrast, monoclonal spread was observed mainly in ovarian (75%, 3/4), bladder (100%, 2/2), lung (66.7%, 2/3), and prostate (100%, 1/1), amongst other CRC metastases. Furthermore, among distant metastases, polyclonal seeding appeared to be more in untreated metastases (56.3%, 9/16) than treated metastases (14.3%, 2/14) (BH adjusted *p* = 0.026, Chi-squared test). Polyclonal seeding occurred via both synchronous polyclonal cell clusters in 46.2% (6/13), and 53.8% (7/13) with asynchronous polyclonal seeding over multiple waves, arising from distinct spatial regions in the primary tumour.

All metachronous distant metastases (100%, 12/12), irrespective of the organ site colonised, appeared to incur exclusive monoclonal seeding, whereas synchronous metastases seemingly had near-equivalent proportions of monoclonal (38.9%, 7/18) and polyclonal seeding (61.1%, 11/18) (BH adjusted *p* = 0.002; Chi-squared test). However, this was likely because of treatment, as more (75%, 9/12) metachronous than synchronous (22.2%, 4/18) distant metastases were treated (BH adjusted *p* = 0.008; Chi-squared test).

### 2.4. Metastatic Divergence

**Figure 3:**
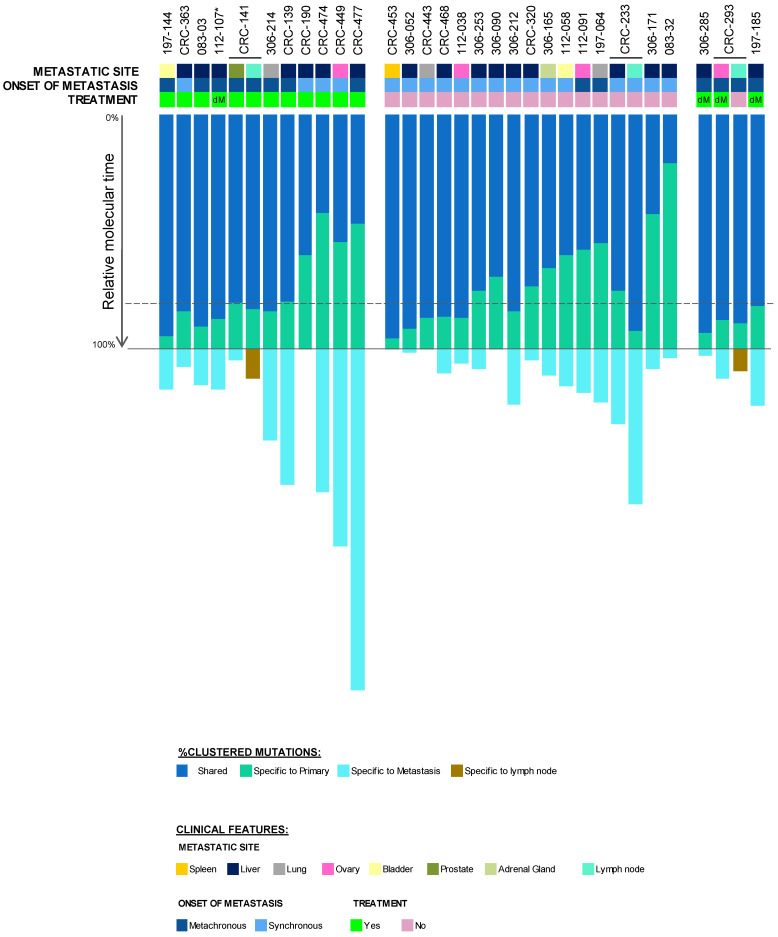
Relative metastatic divergence in metastatic colorectal cancer cohort. %Final molecular time at primary tumour (or lymph node metastasis, where relevant), before dissemination to the metastatic site (local and/or distant), for 30 patients. The dashed line represents the median %final molecular time (79.6%) at the primary tumour. The metastatic site, onset of metastasis, and treatment of each tumour is shown in the panel above. “dM” represents distant metastasis. The treatment status of the primary tumour of patient 112-107, marked by an asterisk (*), was not available.

To estimate the timing of dissemination in relation to the founding of the primary tumour, the final molecular time at the primary tumour was approximated and further classified as late (final molecular time ≥ 50%), or early dissemination (final molecular time < 50%) (Figure 3 and Figure S4).

Whereby, most clonal diversity emerged at the primary CRC site, which was distributed to distant metastatic sites, via metastatic divergence after a median of 79.6% (range 20.8–95.3) final molecular time at the primary tumour (Figure 3). Contrastingly, across the cohort, 78.6% (81/103) of all primary tumour clusters were exclusive to primary tumours, either never having metastasised due to natural negative selection (in therapy-naïve metastases), having arisen after the colonisation of the metastatic lesion, having successfully been eradicated under therapy (in treated metastases), or having seemingly not been present due to block sampling.

Nearly all patients exhibited linear metastatic progression patterns in their distant metastases (and regional lymph node metastases, where relevant), irrespective of treatment, clonal seeding, and site colonised, whereby all primary tumours disseminated relatively late in molecular time, gaining most of their genetic diversity at the primary tumour.

In contrast, four patients (083-32, 306-171, CRC-474, and CRC-477) incurred parallel metastatic progression to the liver, disseminating after a median final molecular time of 42.1% (range 20.8–46.7%) at the primary tumour. As expected, early dissemination was mostly found in synchronous metastases (75%), evident irrespective of treatment. Interestingly, early disseminating cases were significantly enriched for five altered (via mutation or copy-number deletion) tumour suppressor driver genes, including *BRCA2* (MIM: 600185; early: 100%, 4/4 cases vs. late: 26.9%, 7/26 cases), *FOXO1* (MIM: 136533; early: 100%, 4/4 cases vs. late: 30.8%, 7/26 cases), *GPC5* (MIM: 602446; early: 100%, 4/4 cases vs. late: 23%, 6/26 cases), *RB1* (MIM: 614041; early: 100%, 4/4 cases vs. late: 23%, 6/26 cases), and *SOX21* (MIM: 604974; early: 100%, 4/4 cases vs. late: 27%, 7/26 cases) (BH adjusted *p* < 0.01; Chi-squared test).

Unlike a previous report [11,32], where synchronous metastases were shown to disseminate significantly earlier than metachronous metastases, no significant difference was found between synchronous and metachronous metastases in the current metastatic CRC cohort. However, treated synchronous metastases (median 57.2%, range 41.8–83.9%) seemingly appeared to disseminate earlier (on average 23.4% earlier) than untreated synchronous metastases (median 79.5%, range 20.8–95.3%) and treated metachronous metastases (median 81.7%, range 46.7–94.3%). However, this did not reach statistical significance, possibly due to the small number of treated synchronous metastases analysed (*p* > 0.05, Chi-squared test).

### 2.5. Therapy-Resistant Subclones

Neutral selection (dN/dS, a ratio of the substitution rate at non-synonymous sites to those at synonymous sites, ~1) was consistently observed throughout the cohort, when considering all exonic mutations in both primary tumours and metastatic lesions (Table S6 and Figure S5). However, upon stratification according to treatment, in the metastases, a sharp decline in selection (dN/dS < 0.75) was observed in metastatic-specific clonal mutations, which substantially increased (dN/dS ≥ 1.5) in the subclonal mutations. Whereas, therapy-naïve tumours remained consistently neutral. This suggests very stringent selective pressure from treatment, before successful colonisation at the metastatic site, presumably to select therapy-resistant ‘fit’ clones in the micrometastasis, whereby positive selection followed during late metastatic progression, to produce diversity that can adapt to the changing microenvironment.

**Figure 4:**
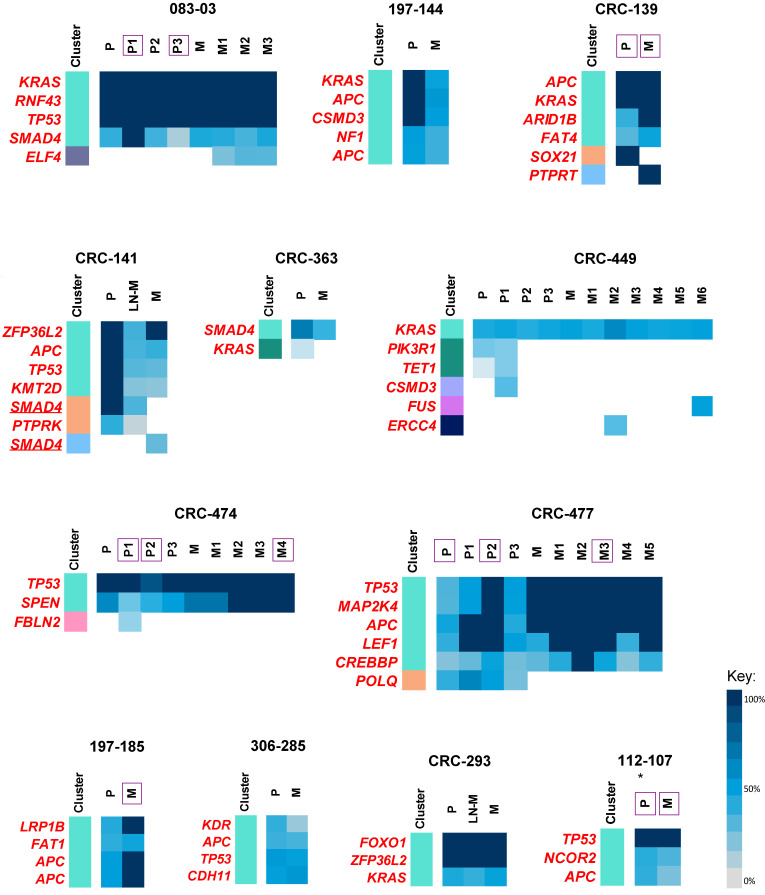
Therapy resistance in treated metastatic colorectal cancer (CRC) samples. Coloured bar represents mutation clusters, with corresponding %cancer cell fractions (%CCF) of each clustered driver mutation as a heatmap. The underlining represents individual parallel mutations converging at the same gene. Tumour regions with genome doubling are indicated with purple squares. “P” refers to primary CRC tumour, “LN-M”, refers to lymph node metastasis, and “M”, refers to the distant metastatic lesion, with multiple spatially distinct regions numbered accordingly, where relevant. The treatment status of the primary tumour of patient 112-107, marked by an asterisk (*), was not available.

In further support of this, the majority of all disseminating primary tumour subclones, across treated samples, showed significant resistance to therapy (see Materials and methods for quantification of ‘therapy resistance’), with significant resistance in *ARID1B* (MIM: 614556), *BMPR2* (MIM: 600799), *CREBBP* (MIM: 600140), *ERBB2* (MIM: 164870), *FAT4* (MIM: 612411), *KRAS*, and *MAP2K4* (MIM: 601335), amongst other driver mutations (Table 2 and Table S5, Figure 4). Moreover, patients showing parallel progression (CRC-474 and CRC-477) exclusively showed resistance in all primary tumour disseminating subclones, comprising *APC*, *TP53*, *CREBBP*, *LEF1* (MIM: 153245), *SPEN* (MIM: 613484), and *MAP2K4* driver mutations. Furthermore, metastatic-specific subclones, containing driver mutations in *ELF4*, *ERCC4*, *FUS*, *PTPRT*, and *SMAD4*, may also confer resistance to therapy. Although select variants in driver genes seemingly displayed resistance or were acquired following therapy, in particular genes including *APC*, *KRAS*, *NF1*, *RNF43*, and *TP53* may have potential to be clinically actionable, by indicating the possible suitability of certain therapies in patients harbouring these variants (Table 2).

## 3. Discussion

Through systematic analysis of WES data from multiple spatial regions of 30 paired primary CRC and distant metastatic tumours, we describe the overall genomic concordance between primary CRC and metastasis, with predominantly late metastatic divergence, through monoclonal or polyclonal spread from metastatic ‘fit’ clones in the primary tumour. Treatment had a diminutive effect on chromosomal instability and most driver mutations, assumingly to attain resistance mechanisms for increased chances of tumour cell survival. Furthermore, the few existing metastatic-specific driver mutations were mostly found in treated cases, similar to previous studies [34,35,36]. The intratumour heterogeneity, post-dissemination, in treated metastases was likely resultant of the collective dysregulation of DNA repair processes, from defective DNA MMR and HR, and the capecitabine and 5-FU chemotherapy, comparable to recent studies [14,32,37].

Of great clinical importance, most cases in our metastatic CRC cohort supported late dissemination, in accordance with the Fearon–Vogelstein multistage progression MSS CRC model [11,12,13,14]. This was consistent, irrespective of treatment status and the organ site colonised. Thus, further highlighting the clinical relevance of current routine screening, and the ability of sigmoid scoping and colonoscopy to successfully detect early CRC lesions, to aid the prevention of metastatic disease. Despite all metastatic-specific driver mutations being present in late disseminating metastases, most driver events were shared between the primary tumour and metastasis, underlining the prospect of using a single diagnostic biopsy from the primary tumour to represent the majority of genomic variation at the metastatic site.

The few metastatic cases in the cohort following an early dissemination model (and similarly cases that incurred polyclonal seeding of the metastasis) can make it difficult to design effective therapeutic strategies using a single biopsy or limited regions, thus warranting the use of additional clinical intervention, such as liquid biopsies (including circulating cell-free tumour DNA profiling) for early detection and metastasis prevention [38]. However, these exceptional cases must not overshadow the importance of screening methods for early detection and the excision of the primary tumour, as suitable ways to reduce CRC mortality in most cases. Two studies have argued that although cancer evolution can be traced using phylogenetic approaches, the timing of dissemination cannot be fully resolved without the aid of other factors, such as chronological references and tumour size or volume, whereby they proposed very early dissemination of primary CRC tumours, i.e., parallel metastatic progression [15,16]. Therefore, metastatic progression in our CRC cohort may also have disseminated earlier than indicated using phylogenetic divergence.

Similar to our metastatic cohort, recent studies have also demonstrated both monoclonal and polyclonal seeding [11,32]. As expected [10,39], polyclonal seeding was more common in lymph node metastases (irrespective of treatment) and therapy-naïve distant metastases (55.6%) compared to treated distant metastases (20%). Thus, in the absence of therapy, multiple primary tumour disseminating subclones acquired metastatic potential for seeding regional and distant sites. Polyclonal seeding occurred both synchronously (at the same time) and asynchronously (over multiple waves), arising from distinct spatial regions in the primary tumour. All lymph node metastases that were seeded through polyclonal spread incurred consecutive waves of seeding, possibly due to their geographical proximity to the primary tumour and higher seeding frequency (as the draining lymph nodes encounter higher rates of tumour cells) compared to distant organ sites [33]. Nascent micrometastases may attract subsequent waves of recurrent seeding for the successful colonisation of a distinct metastatic site [40]. Therefore, the removal of primary tumour (or metastasis) after detection in some cases may be clinically necessary to prevent further metastatic seeding. This intervention has particularly been successful in the management of synchronous metastatic disease, whereby excision of the primary tumour with subsequent adjuvant therapy was associated with better overall survival in prostate cancer and non-small cell lung cancer [41,42].

Furthermore, although adjuvant therapy may effectively target micrometastases, preventing (or at least delaying) further metastatic progression for many patients, it selects for resistant subclones that can make subsequent management and treatment more challenging for patients that do inevitably develop metastasis.

This study was mostly (>70% of primary CRC and distant metastases) based on multi-region WES of precious patient-matched primary CRC and metastatic tumour samples, with additional lymph node metastases in three cases. Furthermore, ultra-depth targeted sequencing (median 2691×, range 1921–3842) was used to validate all somatic variants. Collectively, this allowed a higher resolution for the detection of subclonal mutations, more accurate phylogenetic analysis, and identification of polyclonal seeding patterns. However, the results of this study need to be validated in a larger cohort of multi-region patient-matched primary and metastatic high-depth sequenced data.

## 4. Materials and Methods

### 4.1. Patients and Tumour Samples

We sampled a total of 191 temporally and spatially distinct tumour regions with a median of 8 regions per patient (ranging 2–11), consisting of 92 primary tumour regions (median of 4 regions/patient, ranging 1–5) and 99 matched-metastatic lesions (median 4 regions/patient, ranging 1–7) from the liver (*n* = 18), ovary (*n* = 4), lung (*n* = 3), urinary bladder (*n* = 2), prostate (*n* = 1), adrenal gland (*n* = 1), and spleen (*n* = 1), with corresponding normal colorectal tissue from 30 patients with advanced-stage CRC from King Faisal Specialist Hospital and Research Centre. To assess intratumour heterogeneity, ≥2 spatially distinct tumour regions from each primary CRC and metastatic lesion, separated by a margin of 0.5–1.0 cm (depending on tumour size), were used in this study. Three patients had available metastatic lymph node tissue in addition to their distant metastases. Although 10 patients had incurred further distant metastases to additional organ sites, including the liver, lung, bone, and adrenal gland, the tumour tissue from these sites were not surgically excised due to widespread disease. The median age of CRC onset in Saudi Arabia is ~10 years younger than reported for other ethnicities [1,2]. Clinical characteristics of the CRC cohort are provided in Table 1 and Table S1.

### 4.2. Ethics Statement

This study was conducted in accordance with the Declaration of Helsinki, and approved by the Institutional Review Board (IRB) of the King Faisal Specialist Hospital & Research Centre, Riyadh, Saudi Arabia under the Project RAC # 2190 016, on 8th October 2019. A waiver of consent was granted by the IRB for the use of archived formalin-fixed paraffin-embedded CRC clinical samples. All clinical data was de-identified.

### 4.3. Sample Processing

Formalin-fixed paraffin-embedded (FFPE) tumour tissues were used for genomic DNA extraction using Gentra DNA isolation kit (Gentra, Minneapolis, MN, USA), following the manufacturer’s recommendations previously described [43].

### 4.4. Genotyping

All biopsied tumour regions were amplified using AmpFISTR^®^ Identifiler^®^ (polymerase-chain reaction amplification kit, Applied Biosystems, Waltham, MA, USA), containing loci D8S1179, D21S11, D7S820, CSF1P0, D3S1358, TH01, D13S317, D16S539, D2S1338, D19S433, vWA, TPOX, D18S51, D5S818, FGA, and the Amelogenin locus for sex determination, according to the manufacturer’s recommendations. Fragment size was detected using Applied Biosystems ABI 3130XL genetic analyser and sized with GeneScan500-LIZ internal size standard (Applied Biosystems, Waltham, MA, USA) following the manufacturer’s protocols. Allele calling was performed using GeneMapper ID (v1.1) software (Thermofisher Scientific, Waltham, MA, USA).

### 4.5. Whole-Exome Sequencing

Agilent SureSelectXT Target Enrichment was used for library preparation. The quantification and quality of DNA was measured by PicoGreen and agarose gel electrophoresis. For each sampled biopsy, 1 μg of FFPE genomic DNA was diluted in EB Buffer and sheared to a target peak size of 150–300 bp, using the Covaris LE220 focused-ultrasonicator (Covaris, Woburn, MA, USA), according to the manufacturer’s recommendations. The fragmented DNA was repaired, and adapters were ligated to the fragments. Upon ligation, the adapter ligated product was polymerase chain reaction (PCR) amplified. The final purified product was then quantified using the TapeStation DNA screentape D1000 (Agilent). The captured DNA was then washed and further amplified. The final purified product was then quantified using qPCR, according to qPCR Quantification Protocol Guide (KAPA Library Quantification kits for Illumina Sequencing platforms) and qualified using the TapeStation DNA screentape D1000 (Agilent). Whole-exome sequencing (WES) was performed at a median coverage of 166× (range 199–255), using Illumina NovaSeq 6000 platform (Illumina, San Diego, USA), for each tumour region (*n* = 191) and matched normal colorectal tissue (*n* = 30) (Table S2). Phred quality (Q) scores were calculated to check the accuracy of each base nucleotide called, with greater Q values indicating a greater accuracy of sequencing. The median Q value obtained at the quality score of 30 was 94.21, and at the quality score of 20 was and 97.51 (Table S2). A base nucleotide quality score of 30 indicates the chances of having a base call error to be 1 in 1000 (base call accuracy 99.9%).

Sequencing reads were aligned to the human reference genome hg19 using the Burrows-Wheeler Aligner (BWA) v0.7.15 algorithm; followed by local realignment and PCR duplicate marking via Picard tools (v1.119, http://broadinstitute.github.io/picard/). Base-quality recalibration was performed with GATK v4.0.12.0. All the quality metrics were obtained using GATK and FastQC (http://www.bioinformatics.babraham.ac.uk/projects/fastqc/).

### 4.6. Variant Calling

Short somatic variants, including single nucleotide variants (SNVs), dinucleotide variants, and small insertions and deletions (indels), were called via the short variant caller GATK v4.0.12.0 tool—MuTect2 [44,45], using tumour with matched normal, to exclude germline variants. Variants presenting with the following features were also excluded: common single nucleotide polymorphisms (SNPs; minor allele frequency of >0.01) found in dbSNP, the National Heart, Lung, and Blood Institute exome sequencing project, 1000 Genomes, Exome Aggregation Consortium (ExAC), and in our in-house data from exome sequencing of ~800 normal samples. Only mutations with a variant allele frequency (VAF) ≥1%, minimum alternative reads as 4 and regional sequencing depth ≥15, where germline reads ≥8 with a VAF < 1%, were retained. Variants with more than 2 mapping quality zero reads were considered false-positives and removed from the analysis. In addition to sequencing depth, VAF and minimal counts of alternative reads, a minimal low log odds (LOD) threshold of 6.3 was used for somatic mutations to filter out artefacts.

To verify the accuracy of sequencing, variant calling, and downstream analysis, cancer genes with known roles in CRC [39,46,47] were found as reported previously in the Saudi population [26,27,28], including *APC* (53.3%, 16/30 cases), *TP53* (53.3%, 16/30 cases), *KRAS* (46.7%, 14/30 cases), *SMAD4* (20%, 6/30 cases), *PIK3CA* (10%, 3/30 cases), and *SMAD2* (6.7%, 2/30 cases). Al-Shamsi et al. [48]. by using hotspot mutation testing on 46- or 50-gene panels reported overall 27.3% *APC* positive cases, which were comparable with our results (26.6% in the regions covered by 46-gene panel and 33.3% in the regions covered by 50-gene panel). Furthermore, the low frequency of *APC* in our study as compared to TCGA (81%) has also been reported in other populations [29].

### 4.7. Mutation Validation

Further target capture sequencing using SureSelect DNA Design at a median depth of 2691× (range 1921–3842) was performed to validate the putative somatic variants (Tables S2 and S3). All filters were applied as above. SNVs were further confirmed using BamReadCount, whereas all indels were manually verified using the Integrated Genomics Viewer (IGV) v2.4.10 [49,50] to filter out false positives.

### 4.8. Using SNPs for Patient Sample Mismatch or Swaps

All tumour regions and the corresponding germline sample from a single patient should show a highly similar SNP profile. The VAF of 24 SNPs identified by Pengelly et al. [51] were checked in each tumour region to ensure no sample swaps or contamination.

### 4.9. Microsatellite Stability Classification

All samples were devoid of germline mutations in DNA mismatch repair (MMR) genes, *MLH1*, *MSH2*, *MSH6*, and *PMS2*. To further validate the MSI status from whole-exome sequencing data, the MSIsensor tool [52] was used to determine the MSI score of each sample. All samples were confirmed to be MSS with a lower MSI score (MSI sensor score < 3.5) (Table S7). Finally, immunohistochemistry (IHC) analysis was performed on MMR proteins (MLH1, MSH2, MSH6, and PMS2), as previously described [53], whereby all samples corroborated positive expression of MMR proteins. Collectively, all samples were categorised as MSS.

### 4.10. Mutational Signature Analysis

Mutational signature profiles were predicted using the deconstructSigs package in R, by comparing 30 published mutational signatures from the COSMIC database reported in different cancer types.

### 4.11. Gene Copy Number Profiling, Cancer Cell Fraction, and Genome Doubling

FACETS v0.5.13 [54] was used to determine copy number variations (CNVs) and regions of loss of heterozygosity (LOH). Mean allelic frequency (MAF) was produced from mutation data. ABSOLUTE v1.0.6 [55] defined the integer copy number and cancer cell fractions (CCFs) of mutations and CNVs. CNVs were further defined as amplifications and deletions, in relation to the average ploidy of all sampled regions from a given patient using the copy number for each segment from facets. Gene-level amplifications and deletions were classified by mean gene copy number ≥2× ploidy +1 copy or ≤2× ploidy −1 copy, respectively [23]. Genome doubling was calculated based on the mean ploidy of the sample. If the mean ploidy reported by FACETS was greater than 3, the sample was termed to have undergone genome doubling [56].

### 4.12. Clonality

If all tumour regions acquired the equivalent mutation, the mutation was classified as clonal/trunk. Absence of the mutation in any one region was classified as subclonal/branched. Similarly, if all tumour regions harboured the equivalent CNV, the gene was classified as clonally amplified or clonally deleted. Absence of the CNV in any one region was classified as subclonal.

### 4.13. Driver Mutation Classification

Non-silent variants were classified based on a list of potential driver cancer genes (*n* = 786), from the COSMIC cancer gene census (v90), The Cancer Genome Atlas (TCGA)-CRC [25], and other large genomic studies [35,57]. Putative driver mutations were classified: If the gene was identified as a tumour suppressor by COSMIC and the variant was found to be deleterious (stop-gain or predicted deleterious in two of the three computational tools–SIFT, PolyPhen-2 and MutationTaster); or if the gene was classified as tumour oncogene and an exact variant was found ≥3 times in COSMIC [23].

### 4.14. Driver Copy Number Classification

Copy number events were classified based on the same list of potential driver CNVs detailed above for driver mutation classification. Putative driver CNVs were classified: If the gene was identified as tumour suppressor by COSMIC and found to be a deletion; or if the gene was classified as tumour oncogene and was found to be amplified [23].

### 4.15. Phylogenetic Trees

Cancer cell lineages were reconstructed using LICHeE [58], by clustering all validated (at a median coverage of 2691×) somatic variants from all biopsied tumour regions from a single patient, whilst accounting for presence/absence and CCFs of a somatic variant across the sampled tumour sites and regions.

### 4.16. Molecular Time at Dissemination

Final molecular time at the primary tumour was estimated from the phylogenetic analysis of all mutations in genomic regions with consistent CCF across all sampled regions, by calculating the percentage of shared clustered mutations in the primary tumour and metastasis for each patient, where additional private mutations in either tumour are assumed to have occurred post-dissemination [59]. Molecular time at dissemination was further defined as late dissemination (linear progression), if the percentage of shared clustered mutations between the primary tumour and metastatic lesions ≥50%, or early dissemination (parallel progression), if the percentage of shared clustered mutations was <50% [60].

### 4.17. Quantifying Therapy Resistance

To ascertain the altering subclonal patterns of metastatic progression during treatment, the average CCF of disseminating primary tumour subclones were measured. Subclones found to be equivalent and/or significantly increasing in CCF during metastatic progression (if the average subclone CCF increase between the primary tumour and metastasis yielded *p* < 0.05; Chi-squared test) were considered to be ‘significantly resistant’. Furthermore, significantly resistant driver mutations (≥20% CCF difference) [61] contributing to the overall resistance of the subclone were also described, where relevant (Table 2 and Figure 4). In treated primary-metastasis pairs, all mutations in the primary tumour were either present prior to therapy, and further selected for and expanded in the metastasis (adaptive resistance) or were induced by therapy (acquired resistance) in both the primary tumour and metastatic lesion [62].

### 4.18. dN/dS Analysis

The effect of selection was estimated using a dN/dS (the substitution rate at nonsynonymous sites to those at synonymous sites) ratio [63]. Maximum-likelihood method implemented in dNdScv R package was used to estimate the dN/dS values for missense (ωmis) and nonsense (ωnon) mutations exome-wide.

### 4.19. Heterogeneity Analysis

Intra- and inter-tumour heterogeneity were calculated using the Shannon diversity index (SDI), a measure of species diversity [17,18]. The SDI for each sample (primary, metastasis or primary-metastasis pair) was analysed based on the presence of different tumour clusters using the vegan R package. To confirm the monoclonal or polyclonal origin of metastases, we calculated the root diversity score (RDS), as previously described [33]. The RDS values were estimated using a python script by Reiter et al. (https://github.com/johannesreiter/rootdiversity).

### 4.20. Statistical Analysis

Where relevant, Mann–Whitney U test was utilized to compare continuous variables, Chi-squared test for categorical variables, and Wilcoxon signed-rank test for nonparametric paired groups were used to determine associations. Associations between clinicopathological parameters and heterogeneity (SDI) were performed using ANOVA in R. For all the statistical tests performed, *p* < 0.05 was considered statistically significant. However, to decrease the false discovery rate, *p*-values were corrected using the Benjamini–Hochberg (BH) method, adjusted at <5%.

## 5. Conclusions

This study provides valuable insight into our understanding of tumour evolution and the effect of therapy on metastatic CRC, especially in the Saudi population, with recognised earlier disease onset. Despite most of our cases supporting a late dissemination model, the presence of cases that either incurred prior treatment or have additional intratumour heterogeneity (due to early dissemination or polyclonal spread to the metastasis) may raise clinical challenges for targeted therapy and metastasis prevention. However, the results of this study need to be validated in a larger cohort.

## Figures and Tables

**Table 1:** Clinicopathological variables for the metastatic colorectal cancer cohort (*n* = 30).

Clinicopathological Variables	*n* (%)
Age	
Median	53.0
Range	32–82
≤50 years	13 (43.3)
>50 years	17 (56.7)
Sex	
Male	13 (43.3)
Female	17 (56.7)
Histopathology	
Adenocarcinoma	29 (96.7)
Mucinous carcinoma	1 (3.3)
Tumour Site	
Right colon	6 (20.0)
Left colon and rectum	24 (80.0)
Histologic Grade	
Well differentiated	4 (13.3)
Moderately differentiated	24 (80.0)
Poorly differentiated	2 (6.7)
pT	
T1	0 (0.0)
T2	1 (3.3)
T3	20 (66.7)
T4	9 (30.0)
pN	
N0	5 (16.7)
N1	14 (46.6)
N2	11 (36.7)
Microsatellite Instability	
Microsatellite instable (MSI)	0 (0.0)
Microsatellite stable (MSS)	30 (100.0)
Site of Distant Metastasis	
Liver	18 (60.0)
Lung	3 (10.0)
Ovary	4 (13.3)
Urinary Bladder	2 (6.7)
Spleen	1 (3.3)
Adrenal gland	1 (3.3)
Prostate	1 (3.3)
Treated Primary Tumour	
No	19 (63.3)
Yes	10 (33.3)
Unknown	1 (3.3)
Treated Distant Metastatic Tumour	
No	16 (53.3)
Yes	14 (46.7)
Unknown	0 (0.0)

**Table 2:** Clinically relevant driver mutations implicated in colorectal cancer metastasis and therapy resistance.

Gene	HGVS ^a^ Nomenclature	Response to Treatment	Clinically Actionable
*APC*	c.G2752T:p.E918*	Sensitive	Sensitive to inhibitors of WNT signalling and anti-TRAIL antibodies
*APC*	c.C3340T:p.R1114*	Sensitive	
*APC*	c.G3925T:p.E1309*	Sensitive	
*APC*	c.C646T:p.R216*	Resistant	
*APC*	c.C3871T:p.Q1291*	Resistant	
*APC*	c.C2626T:p.R876*	Resistant	
*APC*	c.A4156T:p.R1386*	Sensitive	
*APC*	c.C3268T:p.Q1090*	Resistant	
*ARID1B*	c.G5515T:p.E1839*	Resistant	-
*CDH11*	c.G1732A:p.G578S	Resistant	-
*CREBBP*	c.C6310T:p.R2104C	Resistant	-
*CSMD3*	c.C7792T:p.R2598*	Sensitive	-
*ELF4*	c.G460T:p.E154*	Metastatic specific	-
*ERCC4*	c.G349C:p.D117H	Metastatic specific	-
*FAT1*	c.C10271T:p.T3424M	Resistant	-
*FAT4*	c.G7531C:p.E2511Q	Resistant	-
*FOXO1*	c.G1763A:p.G588D	Resistant	-
*FUS*	c.A1361T:p.D454V	Metastatic specific	-
*KMT2D*	c.C12634T:p.R4212W	Sensitive	-
*KRAS*	c.G35A:p.G12D	Resistant	Resistant to anti-EGFR therapies; sensitive to MEK inhibitors; and targetable by immunotherapies.
*KRAS*	c.G35T:p.G12V	Resistant	
*KRAS*	c.A183C:p.Q61H	Resistant	
*LEF1*	c.G614A:p.G205E	Resistant	-
*LRP1B*	c.G4551T:p.Q1517H	Resistant	-
*MAP2K4*	c.T776A:p.F259Y	Resistant	-
*NCOR2*	c.G2797A:p.D933N	Resistant	-
*NF1*	c.A7409T:p.N2470I	Sensitive	Sensitive to PI3K pathway inhibitors; and resistant to RAF and MEK inhibitors.
*PTPRK*	c.C3949T:p.Q1317*	Sensitive	-
*PTPRT*	c.C925G:p.P309A	Metastatic specific	-
*RNF43*	c.T158A:p.L53*	Resistant	Sensitive to porcupine inhibitors
*SMAD4*	c.T362G:p.L121*	Resistant	-
*SMAD4*	c.A1064T:p.D355V	Metastatic specific	
*SMAD4*	c.G1082A:p.R361H	Sensitive	
*SMAD4*	c.C1363T:p.Q455*	Sensitive	
*SPEN*	c.G578A:p.R193H	Resistant	-
*TP53*	c.G524A:p.R175H	Resistant	Sensitive to cell cycle inhibitors and p53 specific gene therapies or immunotherapies.
*TP53*	c.C586T:p.R196*	Resistant	
*TP53*	c.C742T:p.R248W	Resistant	
*TP53*	c.C378G:p.Y126*	Resistant	
*TP53*	c.G919T+1	Resistant	
*ZFP36L2*	c.T502C:p.C168R	Resistant	-
*ZFP36L2*	c.460dupT:p.Y154fs*320	Resistant	

Note: asterisk (*) = a termination code/stop codon. ^a^ Human Genome Variation Society (HGVS) nomenclature.

## Data Availability

The full metastatic CRC dataset is registered in Sequence Read Archive (SRA) database, under the accession PRJNA648037.

## Supplementary Materials

The following are available online at https://www.mdpi.com/2072-6694/12/10/2938/s1, Figure S1: Raw copy number plots using FACETS, Figure S2: Box plot representation of tumourheterogeneity, via (a) Shannon Diversity Index (SDI) (b) Root Diversity Scores (RDS) of 21 samples with multiple sampled regions, Figure S3: Contribution of mutational signatures, Figure S4: Phylogenetic trees, Figure S5: Selection in primary CRC and metastatic tumour lesions, Table S1: Clinicopathological details of 30 CRC metastatic cases, Table S2: The quality control metrics of the whole exome and targeted capture sequencing., Table S3: Somatic mutations validated by target capture sequencing, Table S4: Copy number variations predicted by using FACETS algorithm, Table S5: Variant allele frequencies and cancer cell fraction of somatic mutations, Table S6: dN/dS scores for different types of mutations, Table S7: Microsatellite instability predicted using whole exome sequencing data on MSIsensor platform.

## References

[1] Bazarbashi S. Cancer Incidence Report Saudi Arabia 2014. https://nhic.gov.sa/eServices/Documents/2014.pdf.

[2] Siegel R., Miller K.D., Sauer A.G., Fedewa S.A., Butterly L.F., Anderson J.C., Cercek A., Smith R.A., Jemal A. (2020). Colorectal cancer statistics, 2020. CA A Cancer J. Clin..

[3] Ghiringhelli F., Hennequin A., Drouillard A., Lepage C., Faivre J., Bouvier A.-M. (2014). Epidemiology and prognosis of synchronous and metachronous colon cancer metastases: A French population-based study. Dig. Liver Dis..

[4] Van Cutsem E., Cervantes A., Nordlinger B., Arnold D. (2014). Metastatic colorectal cancer: ESMO Clinical Practice Guidelines for diagnosis, treatment and follow-up. Ann. Oncol..

[5] Vogelstein B., Fearon E.R., Hamilton S.R., Kern S.E., Preisinger A.C., Leppert M., Smits A.M., Bos J.L. (1988). Genetic Alterations during Colorectal-Tumor Development. N. Engl. J. Med..

[6] Turajlic S., Swanton C. (2016). Metastasis as an evolutionary process. Science.

[7] Huang Y., Gao S., Wu S., Song P., Sun X., Hu X., Zhang S., Yu Y., Zhu J., Li C. (2013). Multilayered molecular profiling supported the monoclonal origin of metastatic renal cell carcinoma. Int. J. Cancer.

[8] MacIntyre G., Van Loo P., Corcoran N.M., Wedge D.C., Markowetz F., Hovens C.M. (2016). How Subclonal Modeling Is Changing the Metastatic Paradigm. Clin. Cancer Res..

[9] Heyde A., Reiter J.G., Naxerova K., Nowak M.A. (2019). Consecutive seeding and transfer of genetic diversity in metastasis. Proc. Natl. Acad. Sci. USA.

[10] Ulintz P.J., Greenson J.K., Hardiman K.M., Wu R., Fearon E.R. (2017). Lymph Node Metastases in Colon Cancer Are Polyclonal. Clin. Cancer Res..

[11] Dang H.X., Krasnick B., White B.S., Grossman J.G., Strand M.S., Zhang J., Cabanski C.R., Miller C.A., Fulton R.S., Goedegebuure S.P. (2020). The clonal evolution of metastatic colorectal cancer. Sci. Adv..

[12] Leung M.L., Davis A., Gao R., Casasent A., Wang Y., Sei E., Vilar E., Maru D., Kopetz S., Navin N.E. (2017). Single-cell DNA sequencing reveals a late-dissemination model in metastatic colorectal cancer. Genome Res..

[13] Fearon E.R., Vogelstein B. (1990). A genetic model for colorectal tumorigenesis. Cell.

[14] Ishaque N., Abba M.L., Hauser C., Patil N., Paramasivam N., Huebschmann D., Leupold J.H., Balasubramanian G.P., Kleinheinz K., Toprak U.H. (2018). Whole genome sequencing puts forward hypotheses on metastasis evolution and therapy in colorectal cancer. Nat. Commun..

[15] Hu Z., Ding J., Ma Z., Sun R., Seoane J.A., Shaffer J.S., Suarez C.J., Berghoff A.S., Cremolini C., Falcone A. (2019). Quantitative evidence for early metastatic seeding in colorectal cancer. Nat. Genet..

[16] Lote H., Spiteri I., Ermini L., Vatsiou A., Roy A., McDonald A., Maka N., Balsitis M., Bose N., Simbolo M. (2017). Carbon dating cancer: Defining the chronology of metastatic progression in colorectal cancer. Ann. Oncol..

[17] Shannon C.E. (1948). A mathematical theory of communication. Bell Syst. Tech. J..

[18] Park S.Y., Gonen M., Kim H.J., Michor F., Polyak K. (2010). Cellular and genetic diversity in the progression of in situ human breast carcinomas to an invasive phenotype. J. Clin. Investig..

[19] Alexandrov L.B., Kim J., Haradhvala N.J., Huang M.N., Ng A.W.T., Wu Y., Boot A., Covington K.R., Gordenin D.A., Bergstrom E.N. (2020). The repertoire of mutational signatures in human cancer. Nature.

[20] Christensen S., Van Der Roest B., Besselink N., Janssen R., Boymans S., Martens J.W., Yaspo M.-L., Priestley P., Kuijk E., Cuppen E. (2019). 5-Fluorouracil treatment induces characteristic T>G mutations in human cancer. Nat. Commun..

[21] Harada K., Okamoto W., Mimaki S., Kawamoto Y., Bando H., Yamashita R., Yuki S., Yoshino T., Komatsu Y., Ohtsu A. (2019). Comparative sequence analysis of patient-matched primary colorectal cancer, metastatic, and recurrent metastatic tumors after adjuvant FOLFOX chemotherapy. BMC Cancer.

[22] Bielski C.M., Zehir A., Penson A.V., Donoghue M.T.A., Chatila W.K., Armenia J., Chang M.T., Schram A.M., Jonsson P., Bandlamudi C. (2018). Genome doubling shapes the evolution and prognosis of advanced cancers. Nat. Genet..

[23] Jamal-Hanjani M., Wilson G., McGranahan N., Birkbak N.J., Watkins T.B., Veeriah S., Shafi S., Johnson D.H., Mitter R., Rosenthal R. (2017). Tracking the Evolution of Non-Small-Cell Lung Cancer. N. Engl. J. Med..

[24] Vogelstein B., Papadopoulos N., Velculescu V.E., Zhou S., Diaz L.A., Kinzler K.W. (2013). Cancer Genome Landscapes. Science.

[25] Network T.C.G.A. (2012). Comprehensive molecular characterization of human colon and rectal cancer. Nat. Cell Biol..

[26] Siraj A.K., Parvathareddy S.K., Pratheeshkumar P., Divya S.P., Ahmed S.O., Melosantos R., Begum R., Concepcion R.M.J., Al-Sanea N., Ashari L.H. (2020). APC truncating mutations in Middle Eastern Population: Tankyrase inhibitor is an effective strategy to sensitize APC mutant CRC To 5-FU chemotherapy. Biomed. Pharmacother..

[27] Siraj A.K., Masoodi T., Bu R., Pratheeshkumar P., Al-Sanea N., Ashari L.H., Abduljabbar A., Alhomoud S., Al-Dayel F., Alkuraya F.S. (2017). MED12is recurrently mutated in Middle Eastern colorectal cancer. Gut.

[28] Abubaker J., Bavi P., Al-Harbi S., Ibrahim M., Siraj A.K., Alsanea N., Abduljabbar A., Ashari L.H., Alhomoud S., Al-Dayel F. (2008). Clinicopathological analysis of colorectal cancers with PIK3CA mutations in Middle Eastern population. Oncogene.

[29] Liu Z., Yang C., Li X., Luo W., Roy B., Xiong T., Zhang X., Yang H., Wang J., Ye Z. (2018). The landscape of somatic mutation in sporadic Chinese colorectal cancer. Oncotarget.

[30] Uchi R., Takahashi Y., Niida A., Shimamura T., Hirata H., Sugimachi K., Sawada G., Iwaya T., Kurashige J., Shinden Y. (2016). Integrated multiregional analysis proposing a new model of colorectal cancer evolution. PLoS Genet..

[31] McGranahan N., Favero F., De Bruin E.C., Birkbak N.J., Szallasi Z., Swanton C. (2015). Clonal status of actionable driver events and the timing of mutational processes in cancer evolution. Sci. Transl. Med..

[32] Hu Z., Li Z., Ma Z., Curtis C. (2020). Multi-cancer analysis of clonality and the timing of systemic spread in paired primary tumors and metastases. Nat. Genet..

[33] Reiter J.G., Hung W.-T., Lee I.-H., Nagpal S., Giunta P., Degner S., Liu G., Wassenaar E.C.E., Jeck W.R., Taylor M.S. (2020). Lymph node metastases develop through a wider evolutionary bottleneck than distant metastases. Nat. Genet..

[34] Priestley P., Baber J., Lolkema M.P., Steeghs N., De Bruijn E., Shale C., Duyvesteyn K., Haidari S., Van Hoeck A., Onstenk W. (2019). Pan-cancer whole-genome analyses of metastatic solid tumours. Nat. Cell Biol..

[35] Yaeger R., Chatila W.K., Lipsyc M.D., Hechtman J.F., Cercek A., Sanchez-Vega F., Jayakumaran G., Middha S., Zehir A., Donoghue M.T. (2018). Clinical Sequencing Defines the Genomic Landscape of Metastatic Colorectal Cancer. Cancer Cell.

[36] Zehir A., Benayed R., Shah R.H., Syed A., Middha S., Kim H.R., Srinivasan P., Gao J., Chakravarty D., Devlin S.M. (2017). Mutational landscape of metastatic cancer revealed from prospective clinical sequencing of 10,000 patients. Nat. Med..

[37] Sun J., Wang C., Zhang Y., Xu L., Fang W., Zhu Y., Zheng Y., Chen X., Xie X., Hu X. (2019). Genomic signatures reveal DNA damage response deficiency in colorectal cancer brain metastases. Nat. Commun..

[38] Yamada T., Matsuda A., Koizumi M., Shinji S., Takahashi G., Iwai T., Takeda K., Ueda K., Yokoyama Y., Hara K. (2018). Liquid Biopsy for the Management of Patients with Colorectal Cancer. Digestion.

[39] Wei Q., Ye Z., Zhong X., Li L., Wang C., Myers R.E., Palazzo J.P., Fortuna D., Yan A., Waldman S.A. (2017). Multiregion whole-exome sequencing of matched primary and metastatic tumors revealed genomic heterogeneity and suggested polyclonal seeding in colorectal cancer metastasis. Ann. Oncol..

[40] Gundem G., Van Loo P., Kremeyer B., Alexandrov L.B., Tubio J.M.C., Papaemmanuil E., Brewer D.S., Kallio H.M., Högnäs G., Annala M. (2015). The evolutionary history of lethal metastatic prostate cancer. Nat. Cell Biol..

[41] Löppenberg B., Dalela D., Karabon P., Sood A., Sammon J.D., Meyer C.P., Sun M., Noldus J., Peabody J.O., Trinh Q.-D. (2017). The Impact of Local Treatment on Overall Survival in Patients with Metastatic Prostate Cancer on Diagnosis: A National Cancer Data Base Analysis. Eur. Urol..

[42] Fleckenstein J., Petroff A., Schäfers H.-J., Wehler T., Schöpe J., Rübe C. (2016). Long-term outcomes in radically treated synchronous vs. metachronous oligometastatic non-small-cell lung cancer. BMC Cancer.

[43] Siraj A.K., Masoodi T., Bu R., Beg S., Al-Sobhi S.S., Al-Dayel F., Al-Dawish M., Alkuraya F., Al-Kuraya K.S. (2016). Genomic Profiling of Thyroid Cancer Reveals a Role for Thyroglobulin in Metastasis. Am. J. Hum. Genet..

[44] Cibulskis K., Lawrence M.S., Carter S.L., Sivachenko A., Jaffe D., Sougnez C., Gabriel S., Meyerson M., Lander E.S., Getz G. (2013). Sensitive detection of somatic point mutations in impure and heterogeneous cancer samples. Nat. Biotechnol..

[45] Benjamin D., Sato T., Cibulskis K., Getz G., Stewart C., Lichtenstein L. (2019). Calling somatic snvs and indels with mutect2. BioRxiv.

[46] De Mattos-Arruda L., Sammut S.-J., Ross E.M., Bashford-Rogers R., Greenstein E., Markus H., Morganella S., Teng Y., Maruvka Y., Pereira B. (2019). The Genomic and Immune Landscapes of Lethal Metastatic Breast Cancer. Cell Rep..

[47] Obenauf A.C., Massagué J. (2015). Surviving at a Distance: Organ-Specific Metastasis. Trends Cancer.

[48] Al-Shamsi H.O., Jones J., Fahmawi Y., Dahbour I., Tabash A., Abdel-Wahab R., Abousamra A.O.S., Shaw K.R., Xiao L., Hassan M.M. (2016). Molecular spectrum of KRAS, NRAS, BRAF, PIK3CA, TP53, and APC somatic gene mutations in Arab patients with colorectal cancer: Determination of frequency and distribution pattern. J. Gastrointest. Oncol..

[49] Horvaldsdóttir H., Robinson J.T., Mesirov J.P. (2013). Integrative Genomics Viewer (IGV): High-performance genomics data visualization and exploration. Brief Bioinform..

[50] Robinson J.T., Thorvaldsdóttir H., Wenger A.M., Zehir A., Mesirov J.P. (2017). Variant Review with the Integrative Genomics Viewer. Cancer Res..

[51] Pengelly R.J., Gibson J., Andreoletti G., Collins A., Mattocks C.J., Ennis S. (2015). Erratum to: A SNP profiling panel for sample tracking in whole-exome sequencing studies. Genome Med..

[52] Niu B., Ye K., Zhang Q., Lu C., Xie M., McLellan M.D., Wendl M.C., Ding L. (2013). MSIsensor: Microsatellite instability detection using paired tumor-normal sequence data. Bioinformatics.

[53] Siraj A.K., Prabhakaran S., Bavi P., Bu R., Beg S., Al Hazmi M., Al-Rasheed M., Al-Assiri M., Sairafi R., Al-Dayel F. (2015). Prevalence of Lynch syndrome in a Middle Eastern population with colorectal cancer. Cancer.

[54] Shen R., Seshan V.E. (2016). FACETS: Allele-specific copy number and clonal heterogeneity analysis tool for high-throughput DNA sequencing. Nucleic Acids Res..

[55] Carter S.L., Cibulskis K., Helman E., McKenna A., Shen H., Zack T., Laird P.W., Onofrio R.C., Winckler W., Weir B.A. (2012). Absolute quantification of somatic DNA alterations in human cancer. Nat. Biotechnol..

[56] Arend R.C., Londoño A.I., Montgomery A.M., Smith H.J., Dobbin Z.C., Katre A.A., Martinez A., Yang E.S., Alvarez R.D., Huh W.K. (2018). Molecular Response to Neoadjuvant Chemotherapy in High-Grade Serous Ovarian Carcinoma. Mol. Cancer Res..

[57] Giannakis M., Mu X.J., Shukla S.A., Qian Z.R., Cohen O., Nishihara R., Bahl S., Cao Y., Amin-Mansour A., Yamauchi M. (2016). Genomic correlates of immune-cell infiltrates in colorectal carcinoma. Cell Rep..

[58] Popic V., Salari R., Hajirasouliha I., Kashef-Haghighi D., West R.B., Batzoglou S. (2015). Fast and scalable inference of multi-sample cancer lineages. Genome Biol..

[59] Yates L.R., Knappskog S., Wedge D., Farmery J.H., Gonzalez S., Martincorena I., Alexandrov L.B., Van Loo P., Haugland H.K., Lilleng P.K. (2017). Genomic Evolution of Breast Cancer Metastasis and Relapse. Cancer Cell.

[60] Hosseini H., Obradović M.M.S., Hoffmann M., Harper K.L., Sosa M.S., Werner-Klein M., Nanduri S.L.K., Werno C., Ehrl C., Maneck M. (2016). Early dissemination seeds metastasis in breast cancer. Nature.

[61] Ng C.K.Y., Bidard F.-C., Piscuoglio S., Geyer F.C., Lim R.S., De Bruijn I., Shen R., Pareja F., Berman S.H., Wang L. (2017). Genetic Heterogeneity in Therapy-Naïve Synchronous Primary Breast Cancers and Their Metastases. Clin. Cancer Res..

[62] Kim C., Gao R., Sei E., Brandt R., Hartman J., Hatschek T., Crosetto N., Foukakis T., Navin N.E. (2018). Chemoresistance Evolution in Triple-Negative Breast Cancer Delineated by Single-Cell Sequencing. Cell.

[63] Li S., Connors S., Pisapia D., Huang Y., Xu R., Greenfield J. (2015). Epig-09genetic And Epigenetic Tumor Evolution in Gliomatosis Cerebri. Neuro Oncol..

