# Supplementary material for: Clonal Evolution and Timing of Metastatic Colorectal Cancer
Source: Cancers (Basel). 2020 Oct 12;12(10):2938. doi: 10.3390/cancers12102938 (PMC7601934; doi:10.3390/cancers12102938)
Supplement: Supplementary file 1 [file cancers-12-02938-s001.zip › cancers-949224-suppl-final/Supplementary Data/Supplementary_Figures.pdf]

Supplementary Figures

Figure S1: Raw copy number plots using Facets. Tumour-specific (either primary or metastasis) driver copy-number events, including deleted tumour suppressor genes and amplified oncogenes, have also been indicated where relevant.

083-03-P

083-03-P1

083-03-P2

083-03-P3

083-03-M

083-03-M1

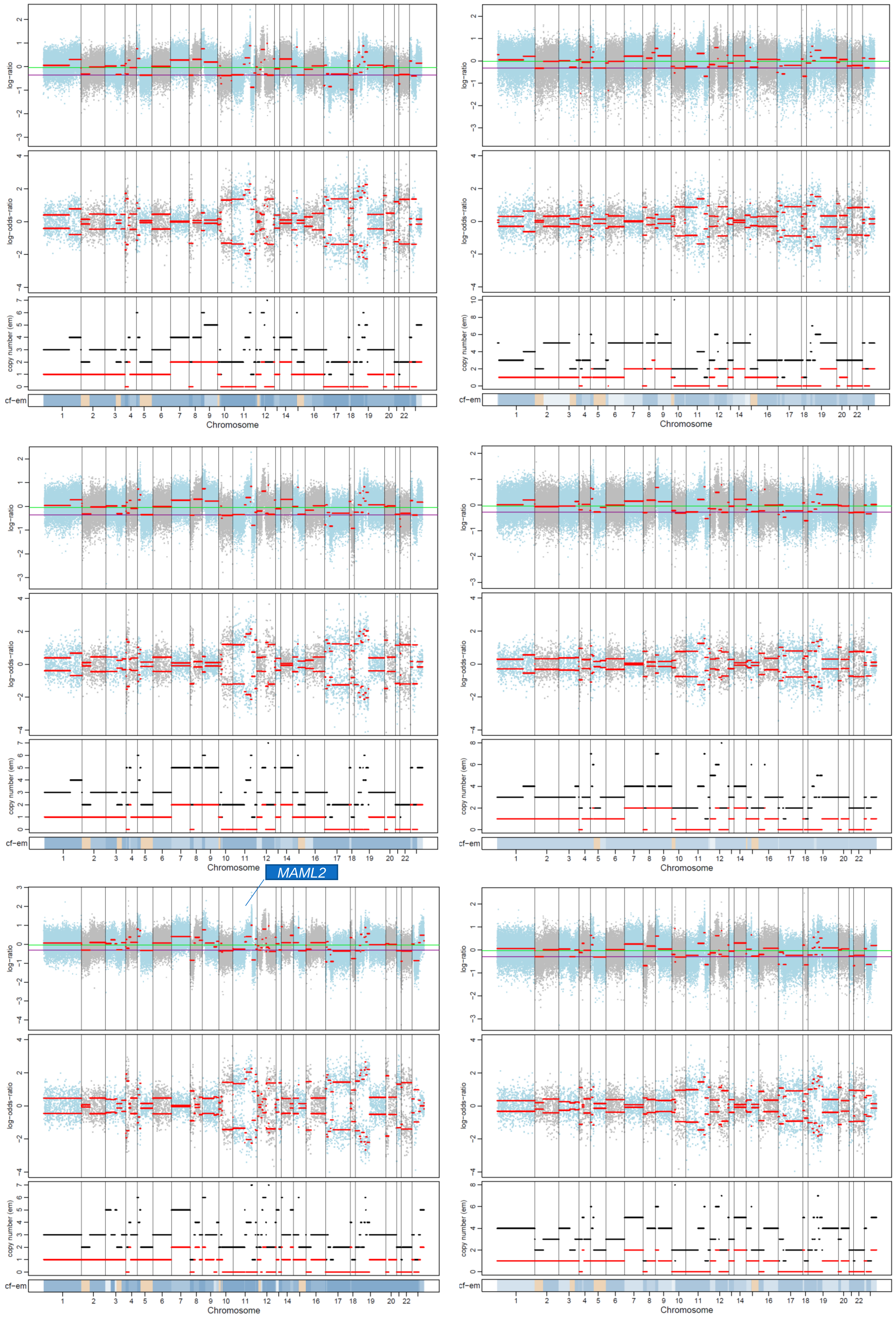

083-03-M2

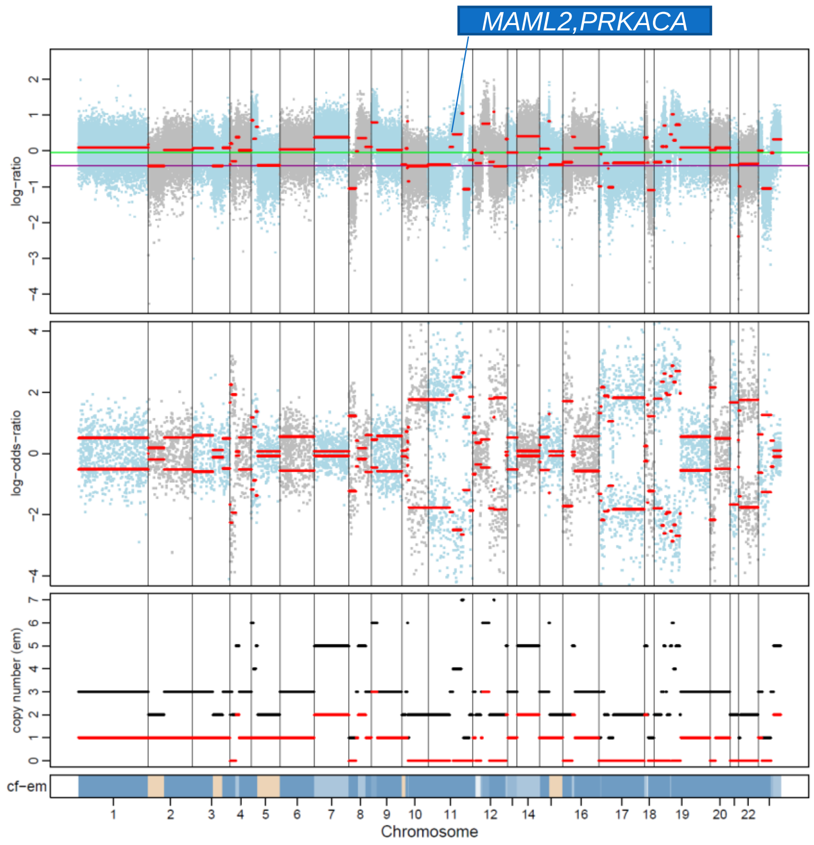

083-03-M3

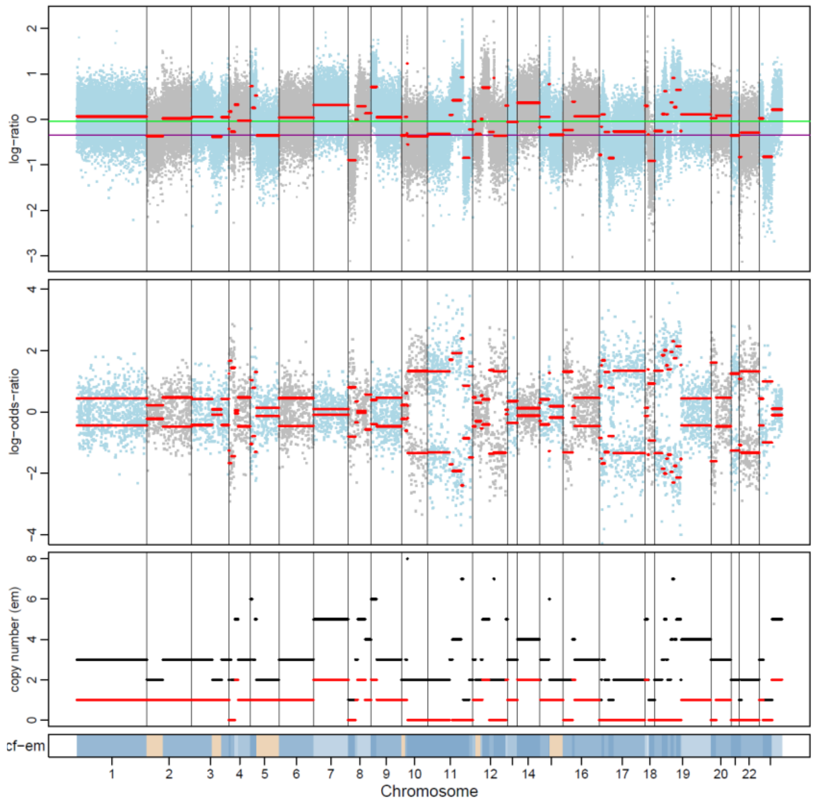

083-32-P

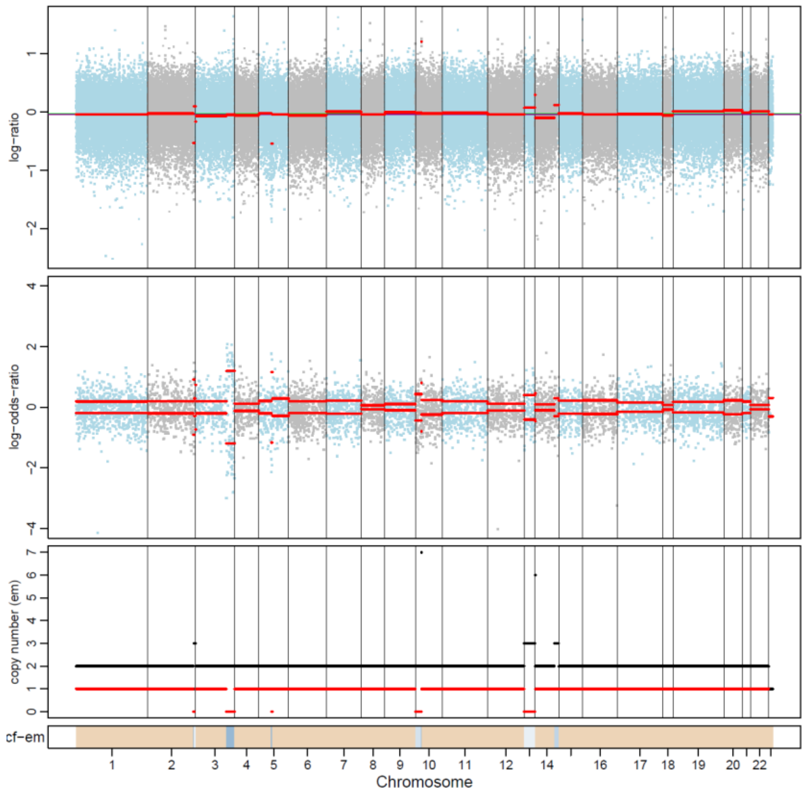

083-32-P1

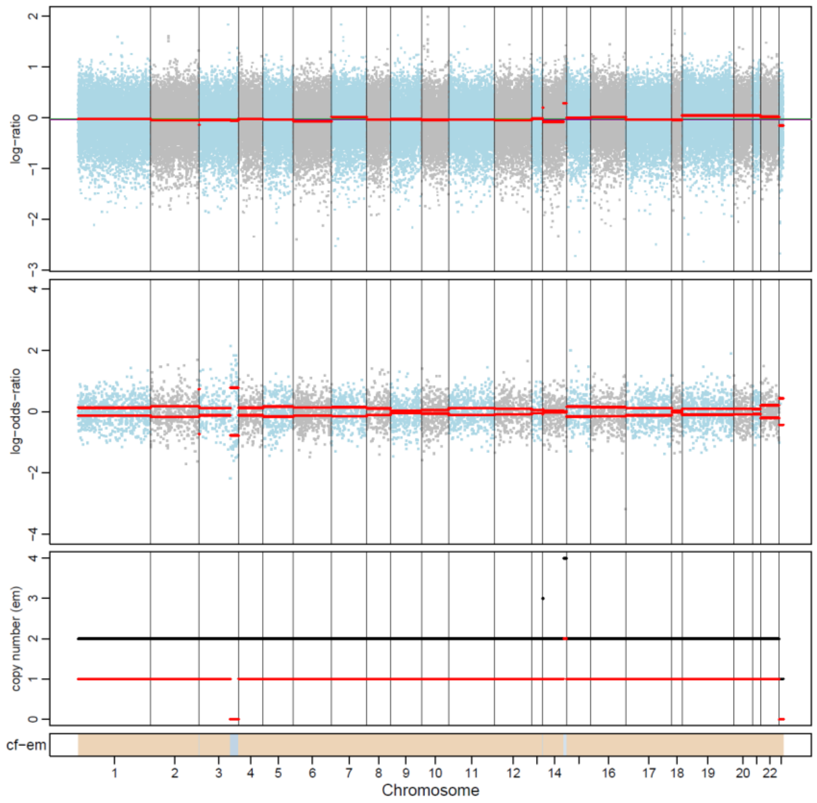

083-32-P2

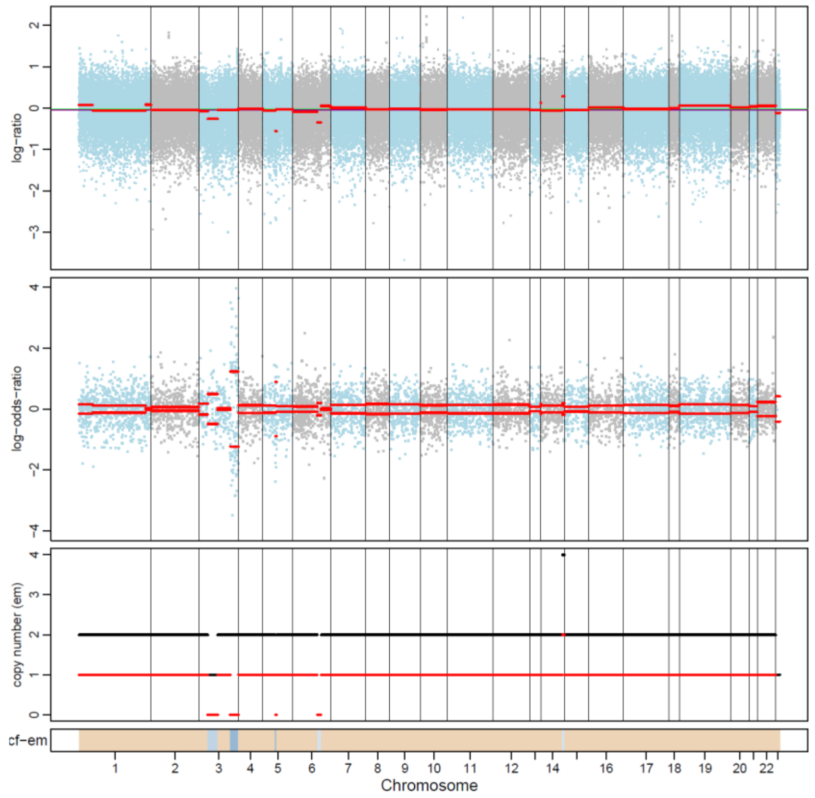

083-32-P3

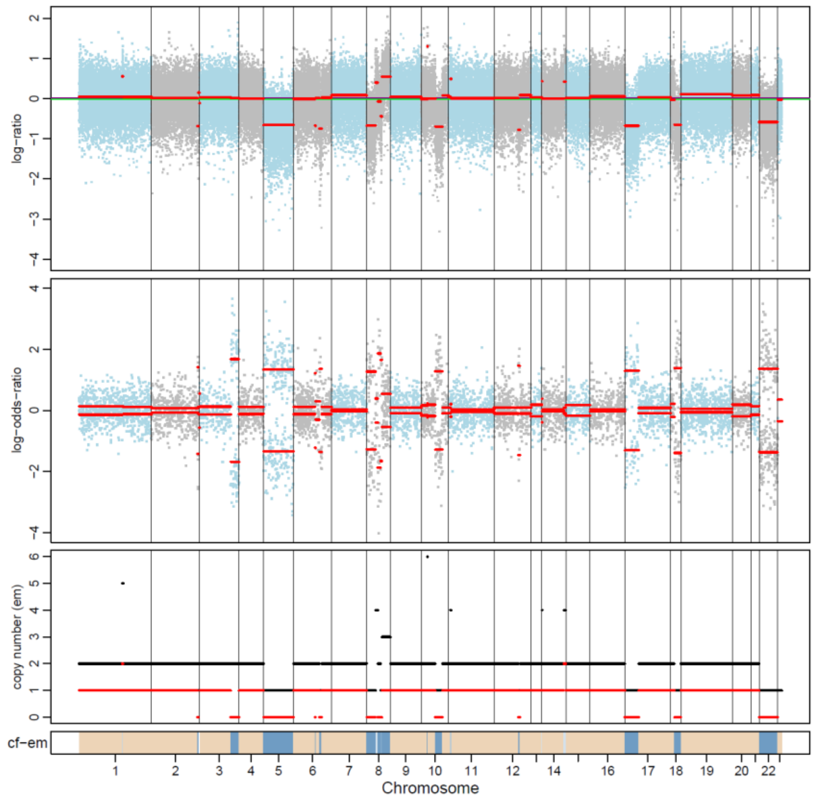

083-32-M

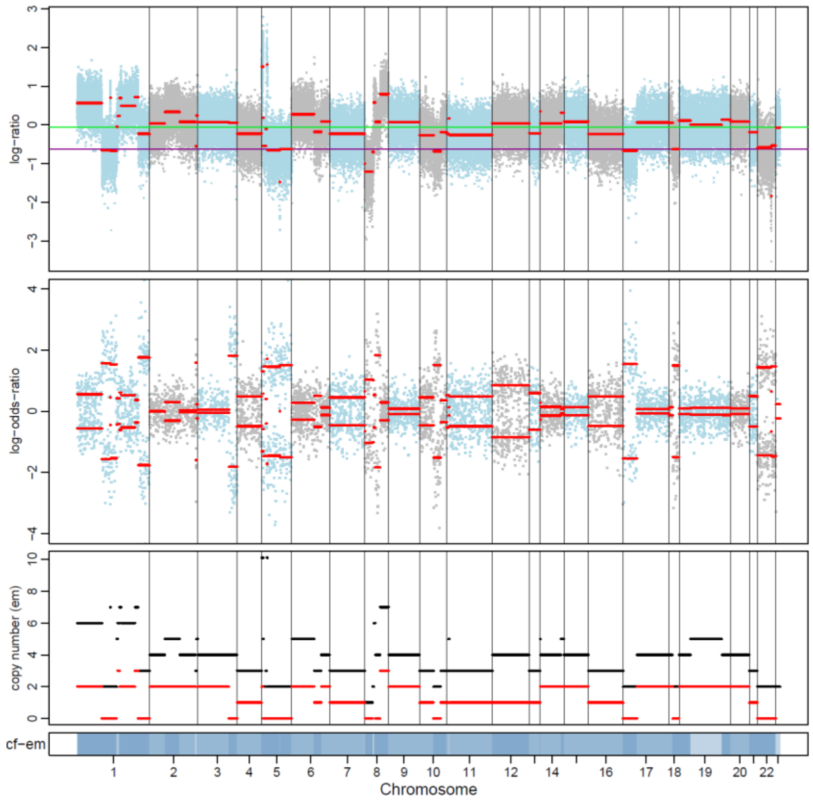

083-32-M1

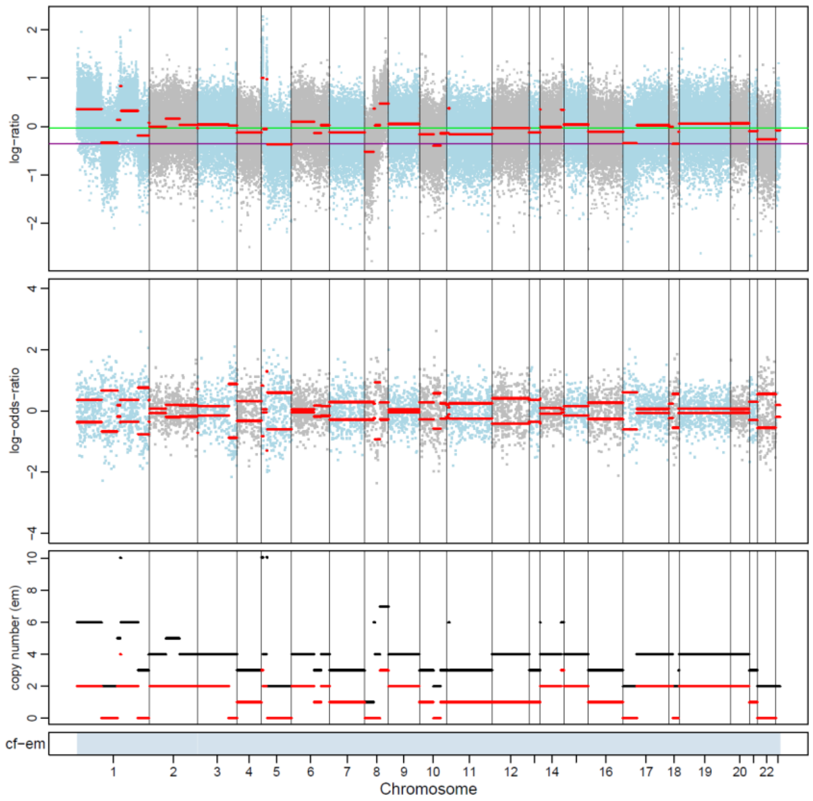

112-038-P

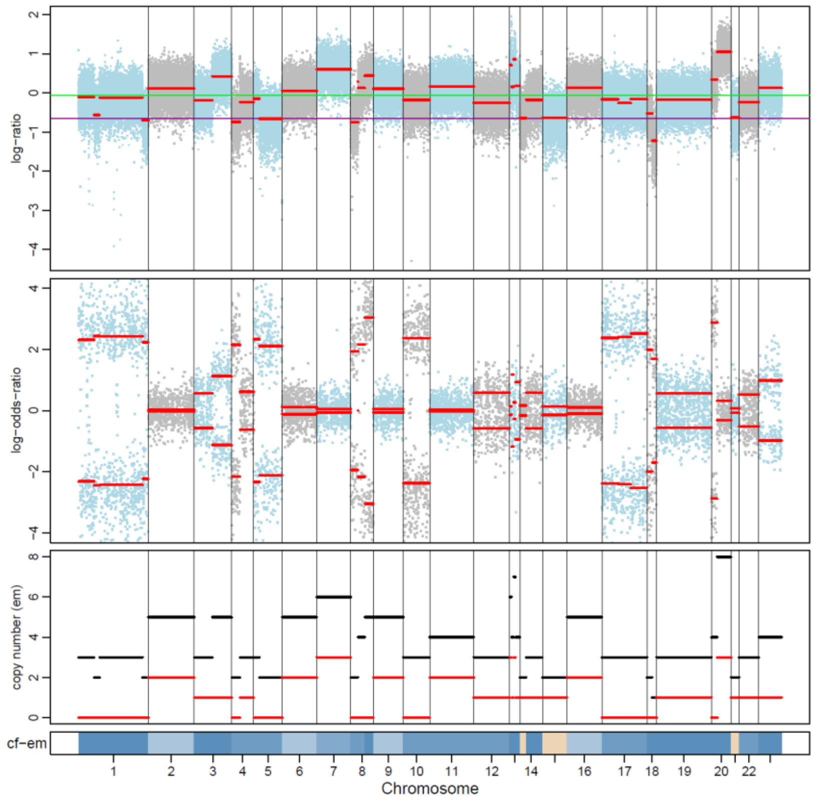

112-038-P1

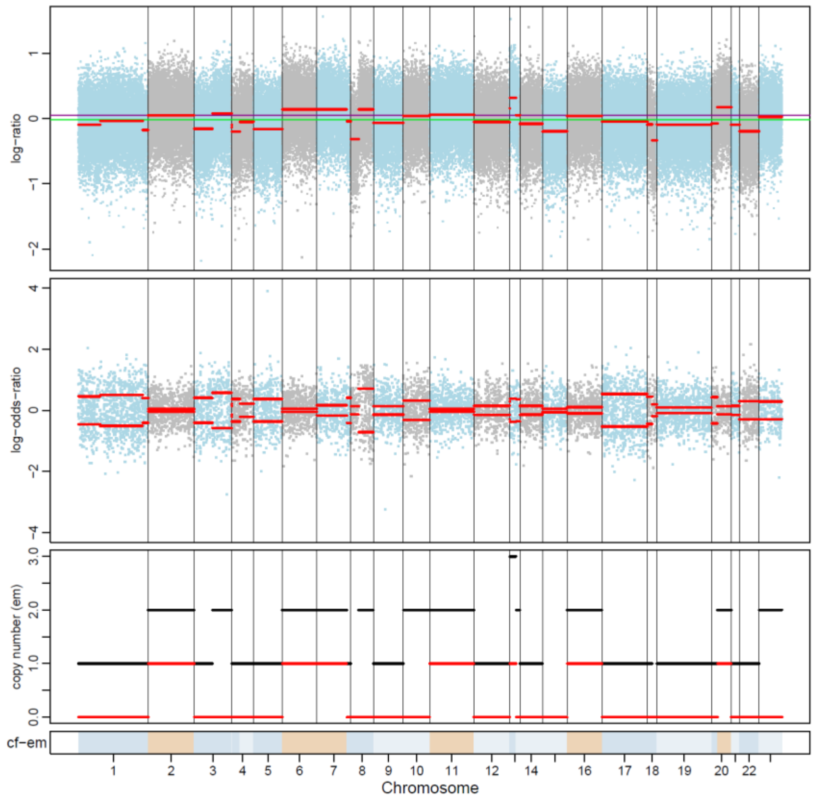

112-038-P2

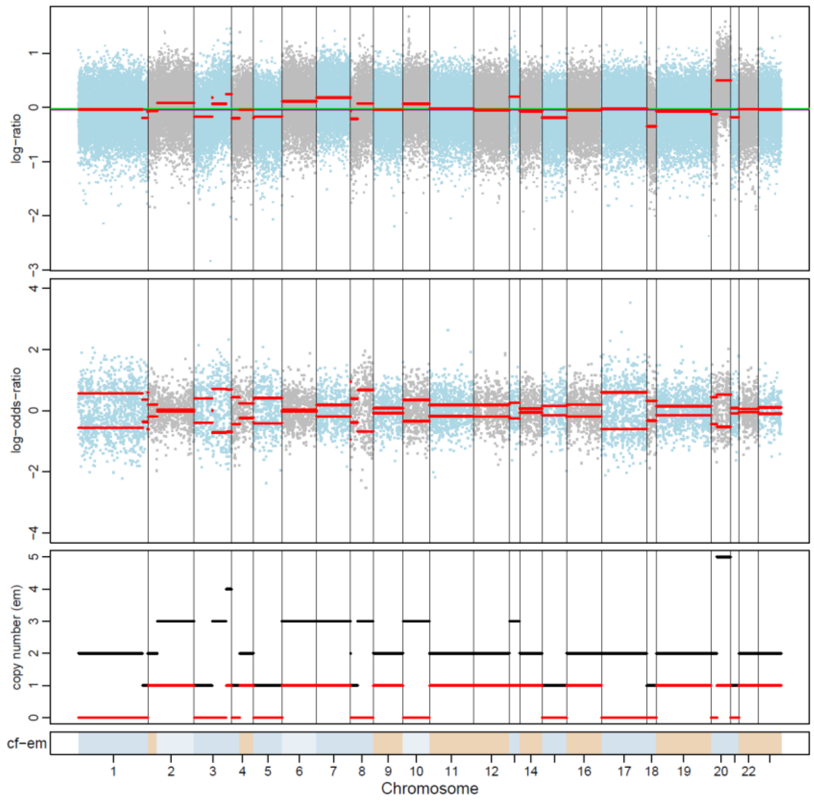

112-038-M

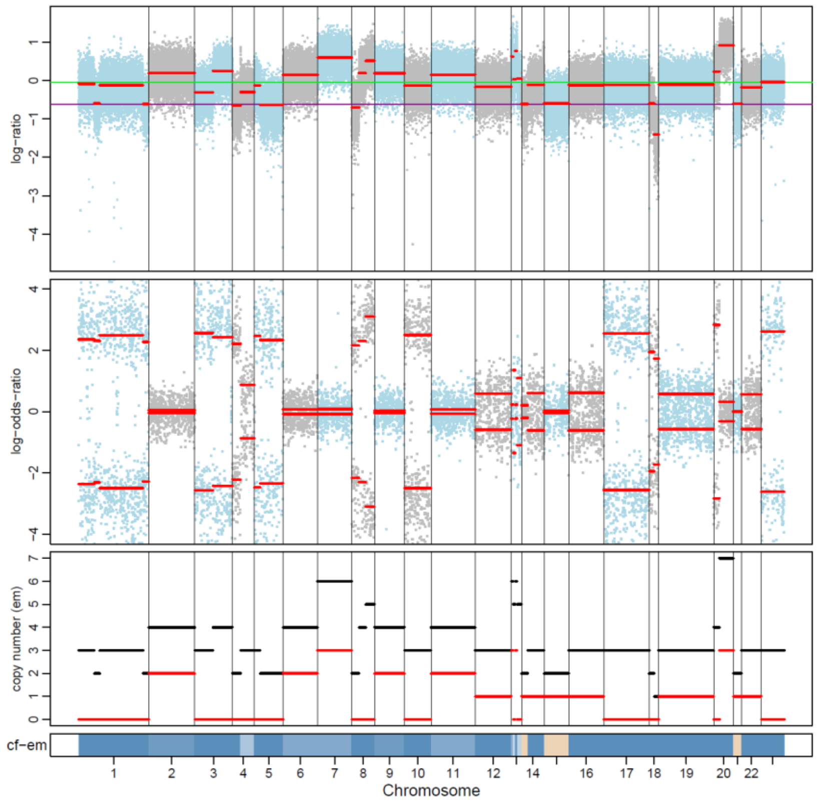

112-038-M1

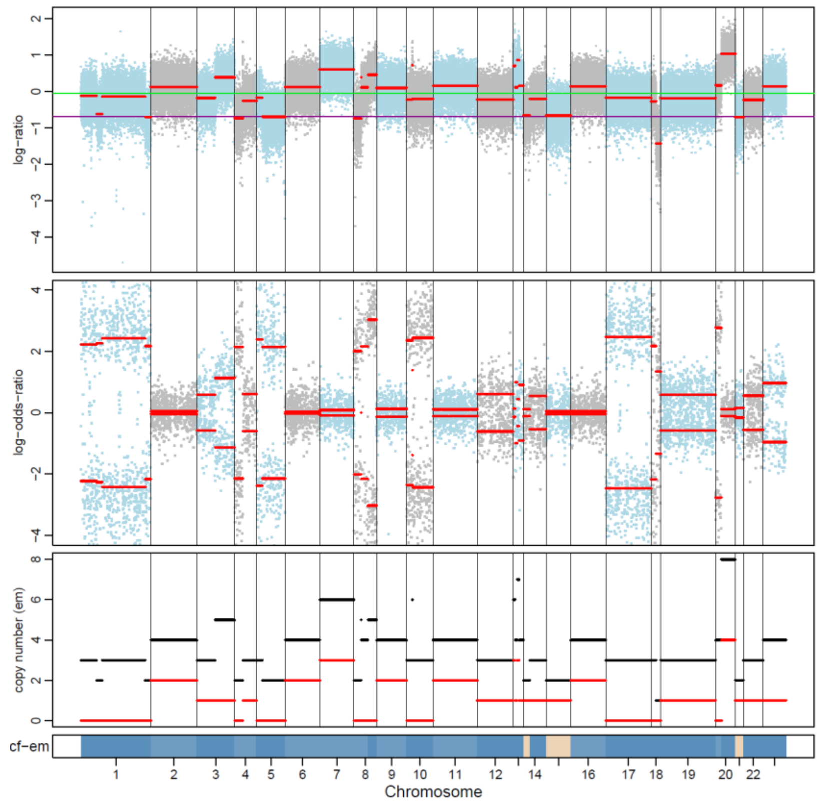

112-038-M2

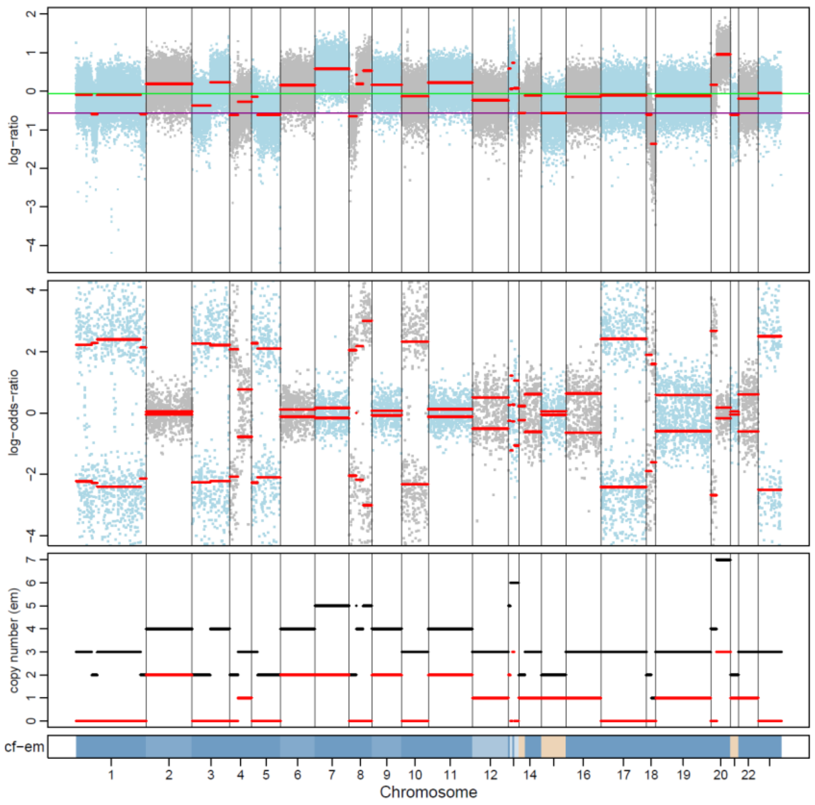

112-038-M3

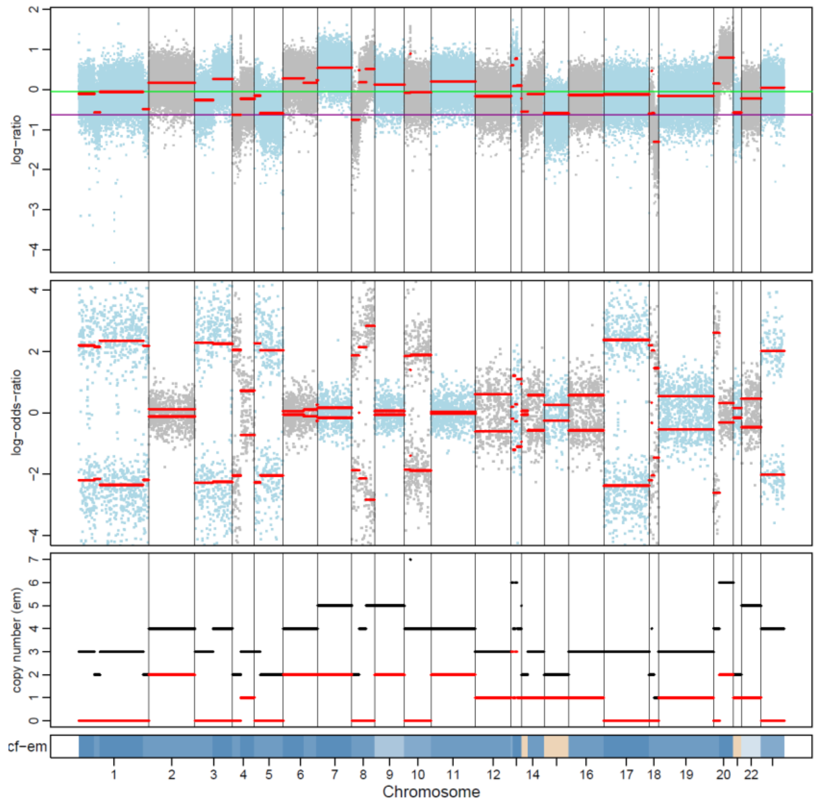

112-058-P

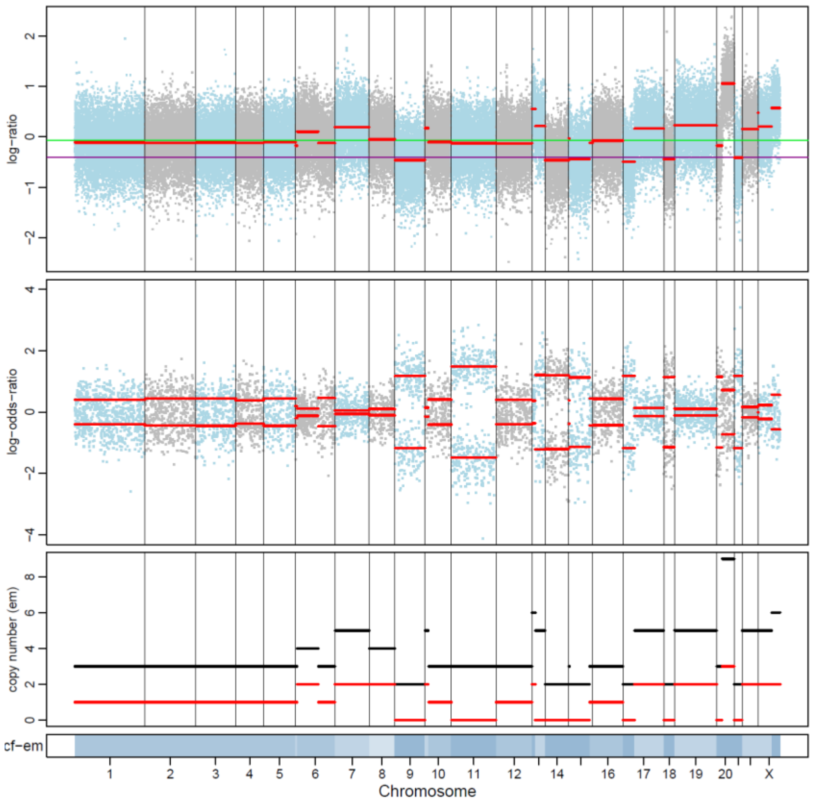

112-058-P1

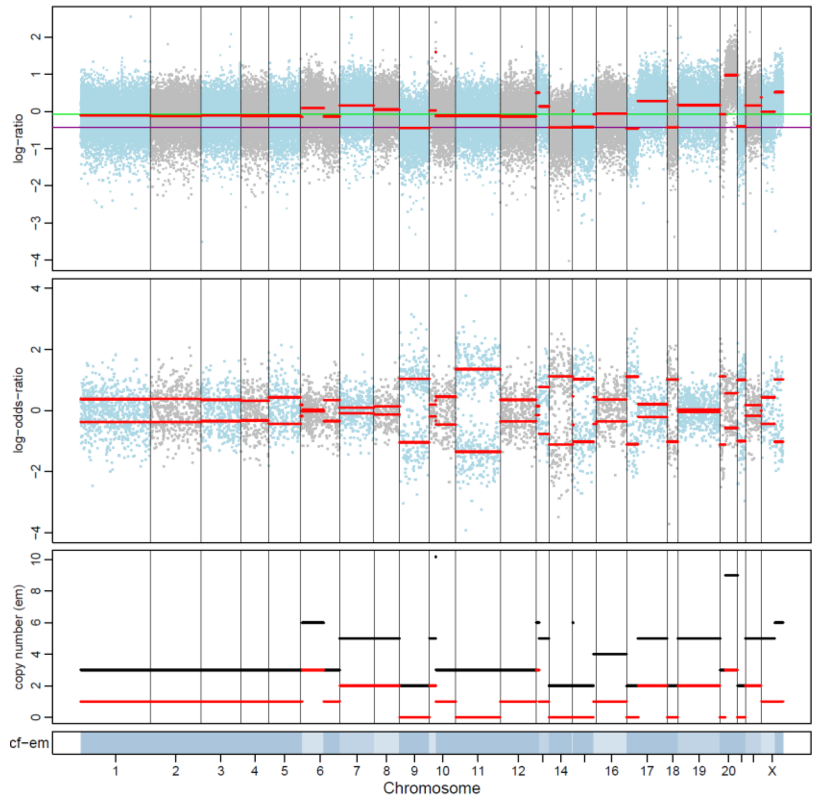

112-058-P2

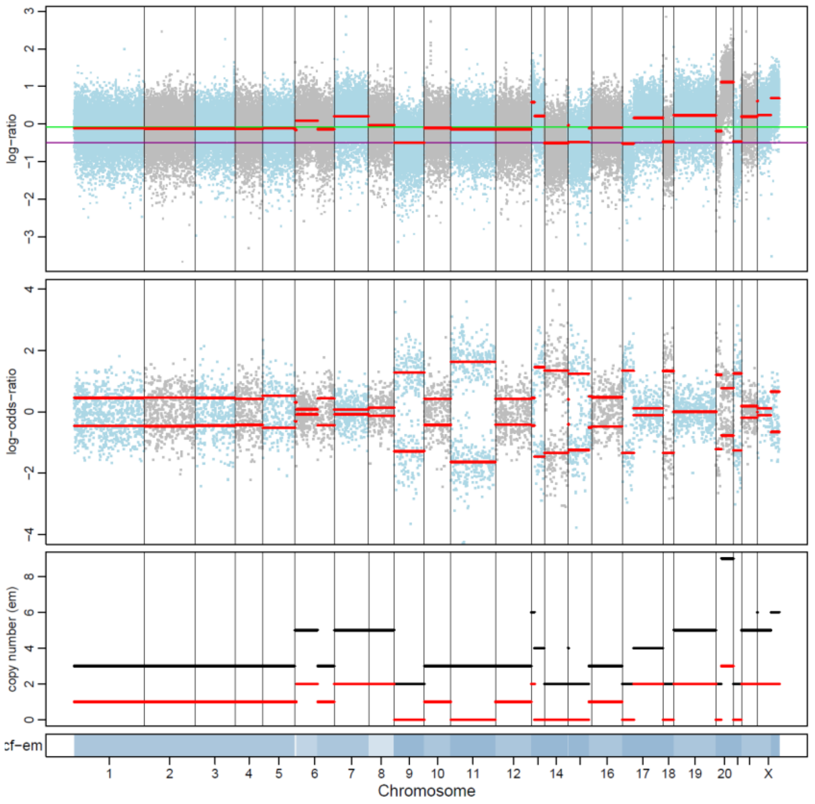

112-058-P3

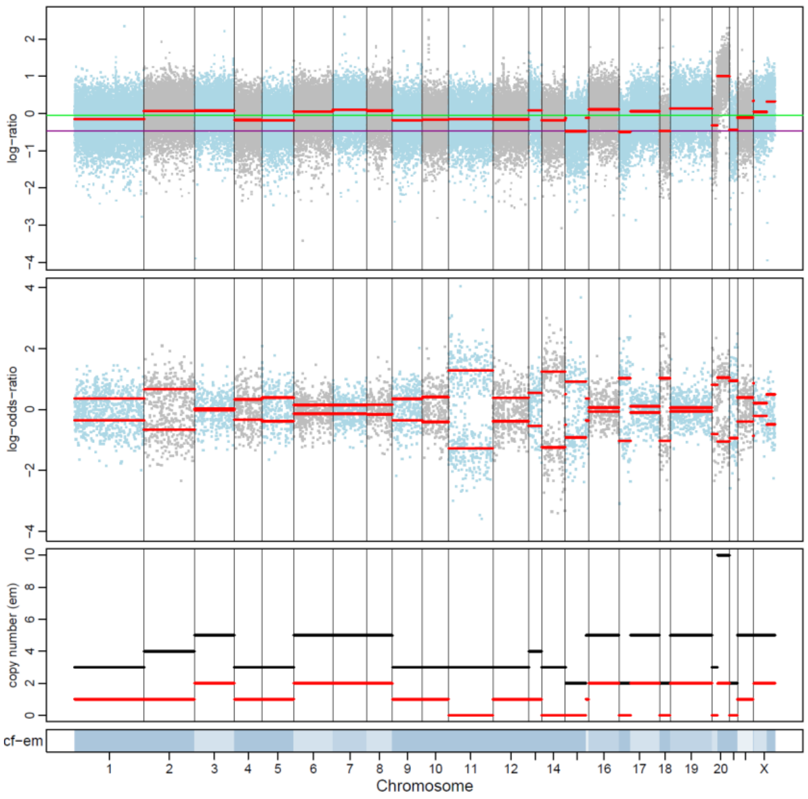

112-058-M

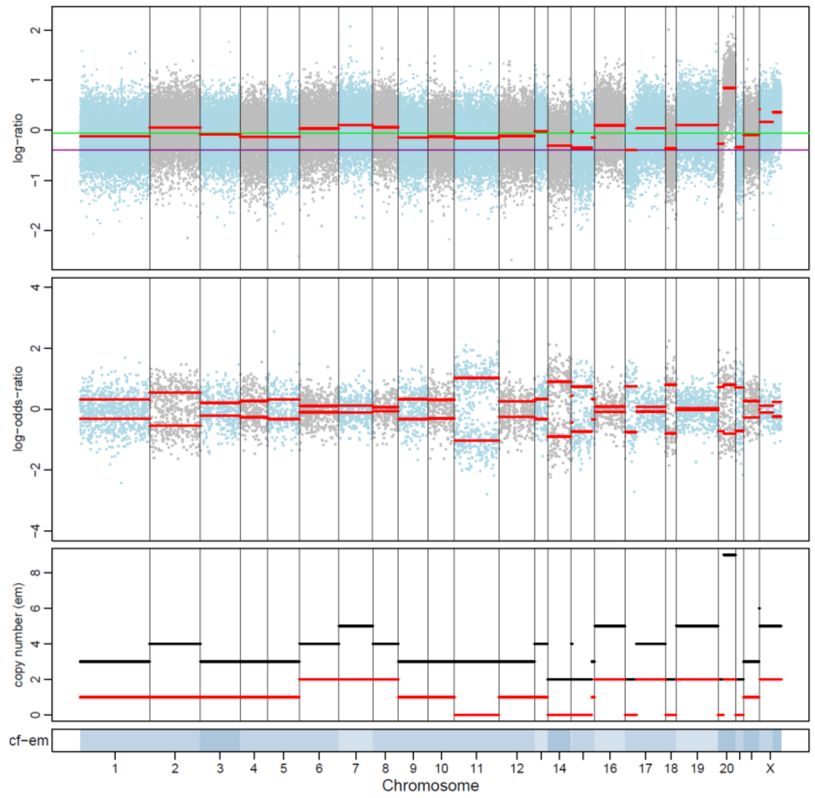

112-058-M1

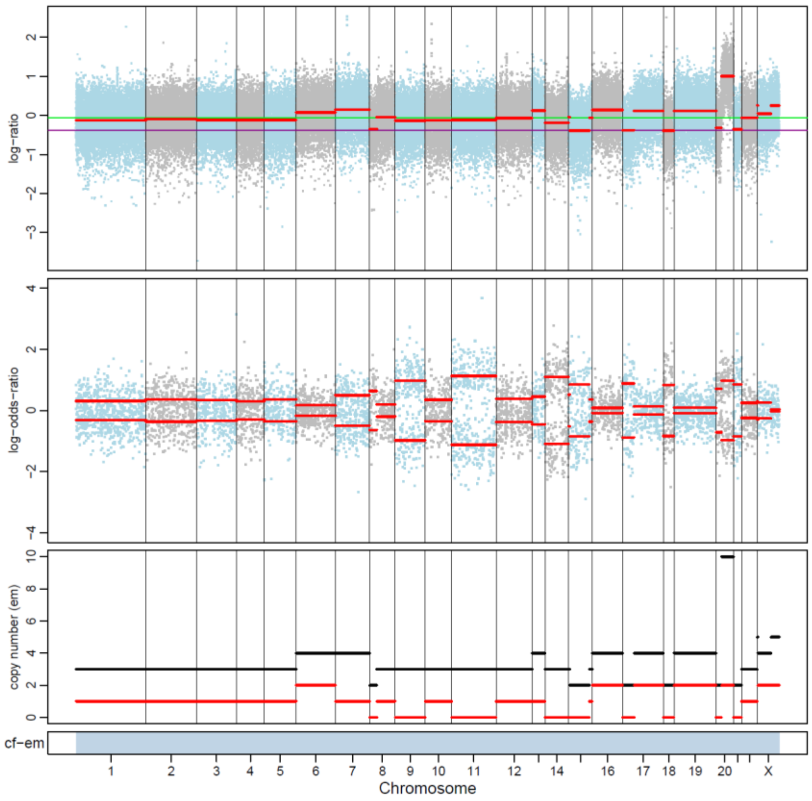

112-058-M2

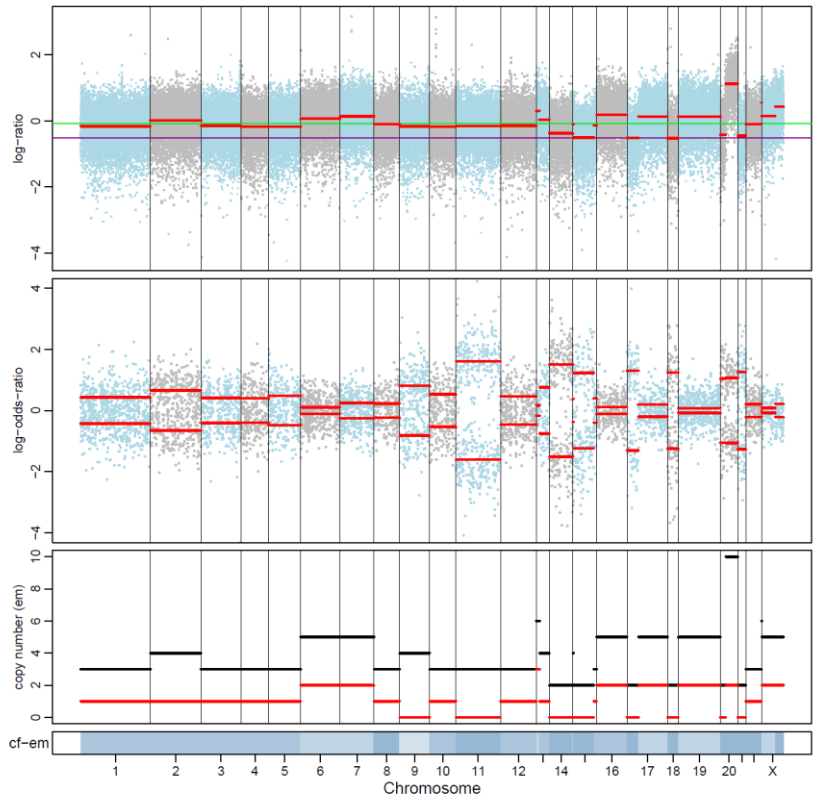

112-058-M3

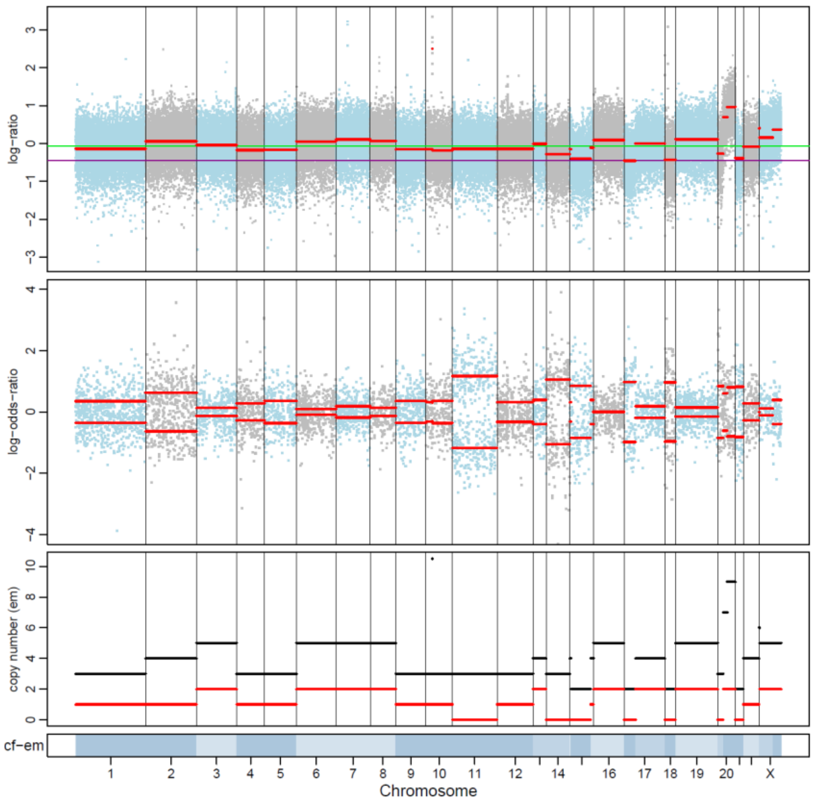

112-091-P

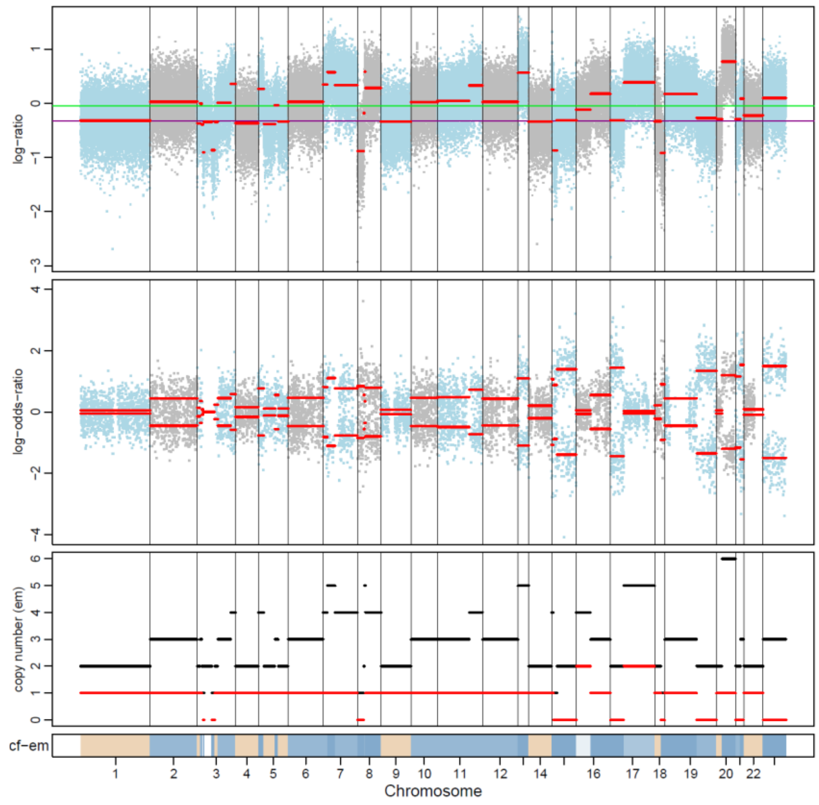

112-091-P1

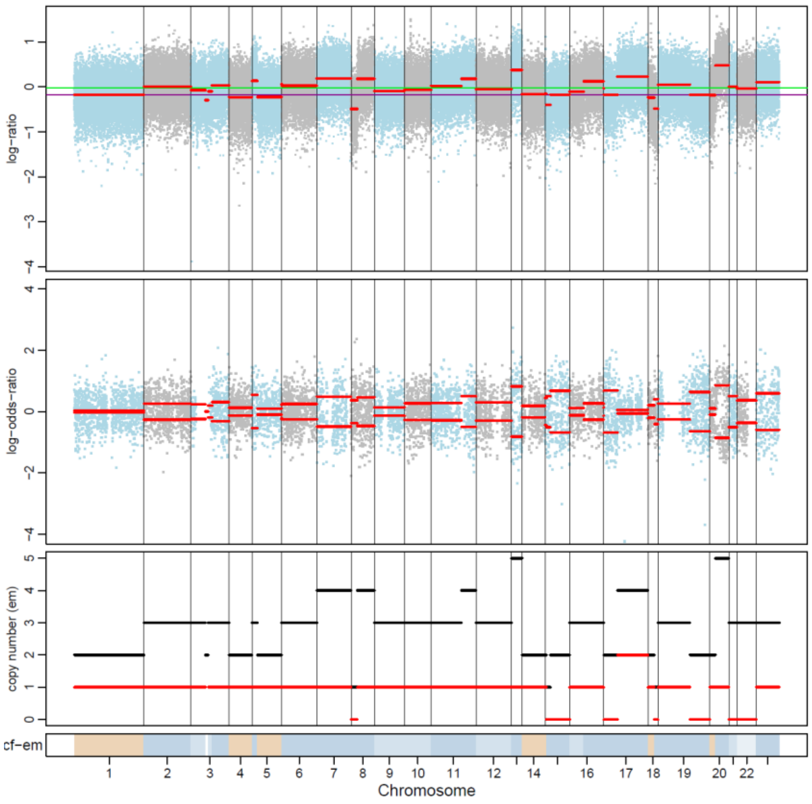

112-091-P2

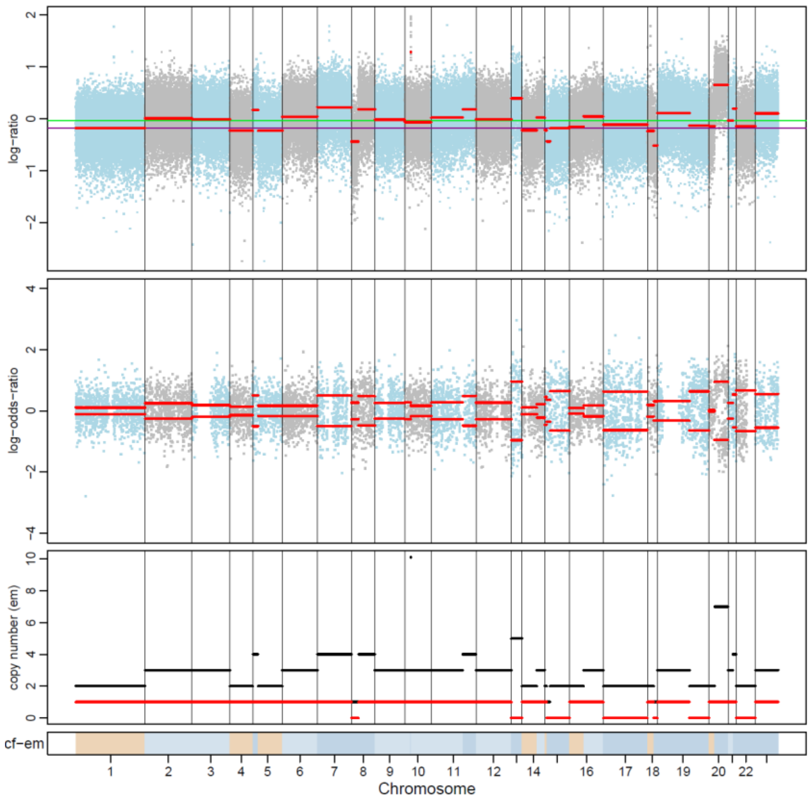

112-091-P3

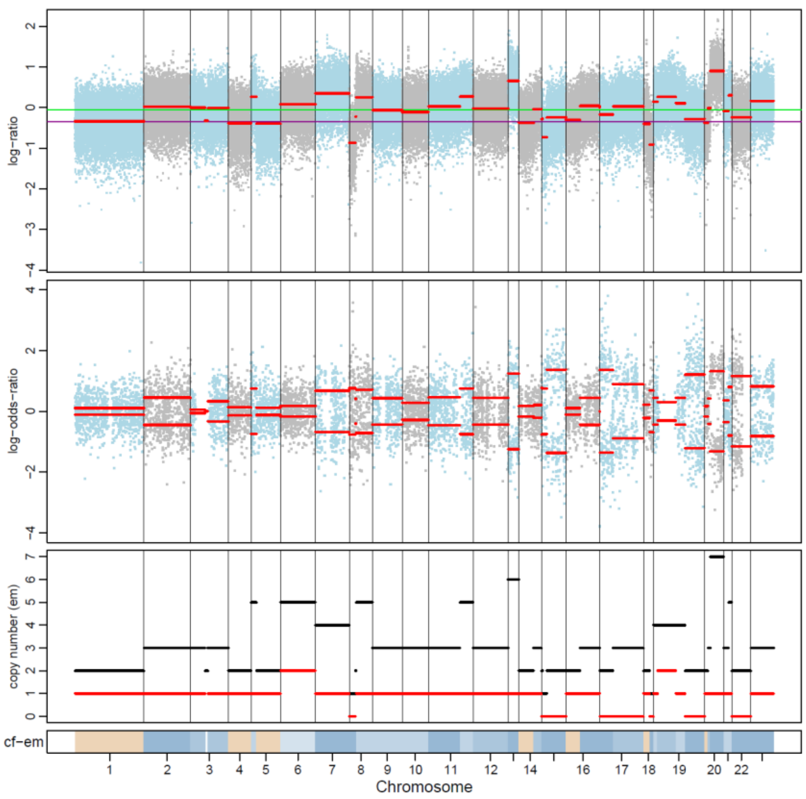

112-091-M

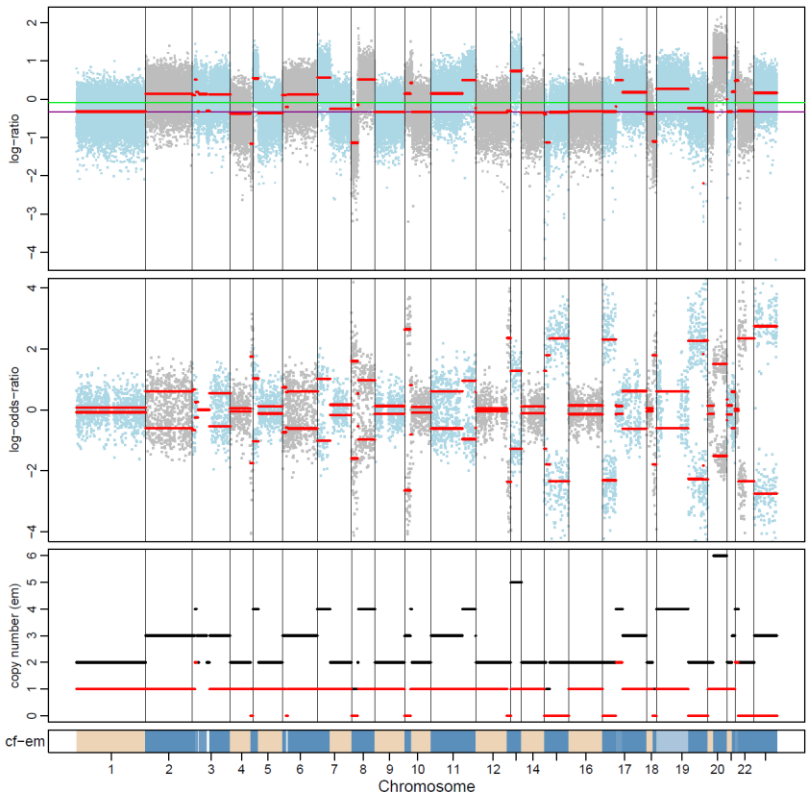

112-091-M1

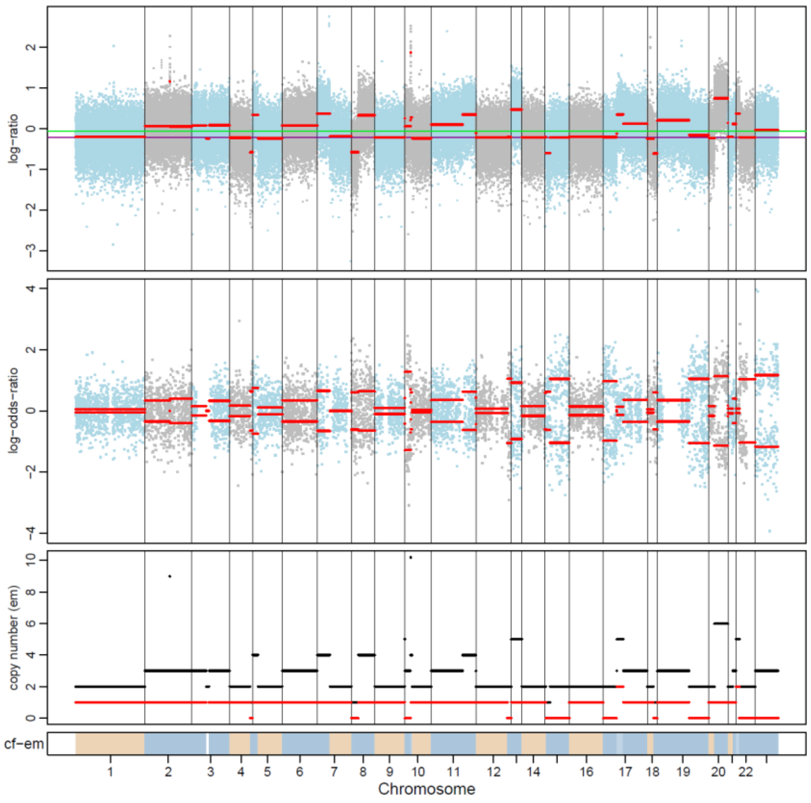

112-091-M2

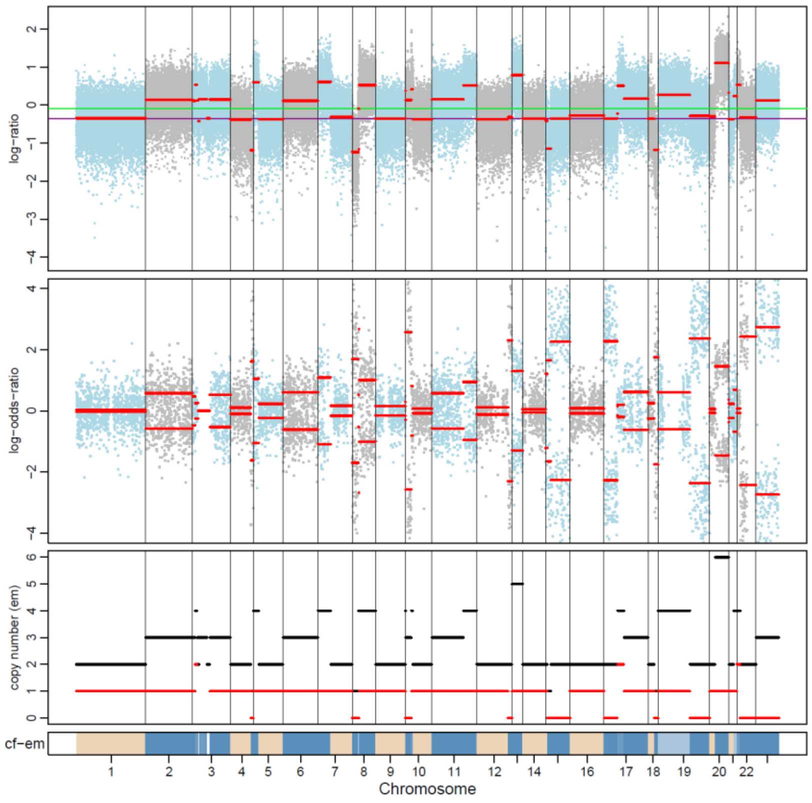

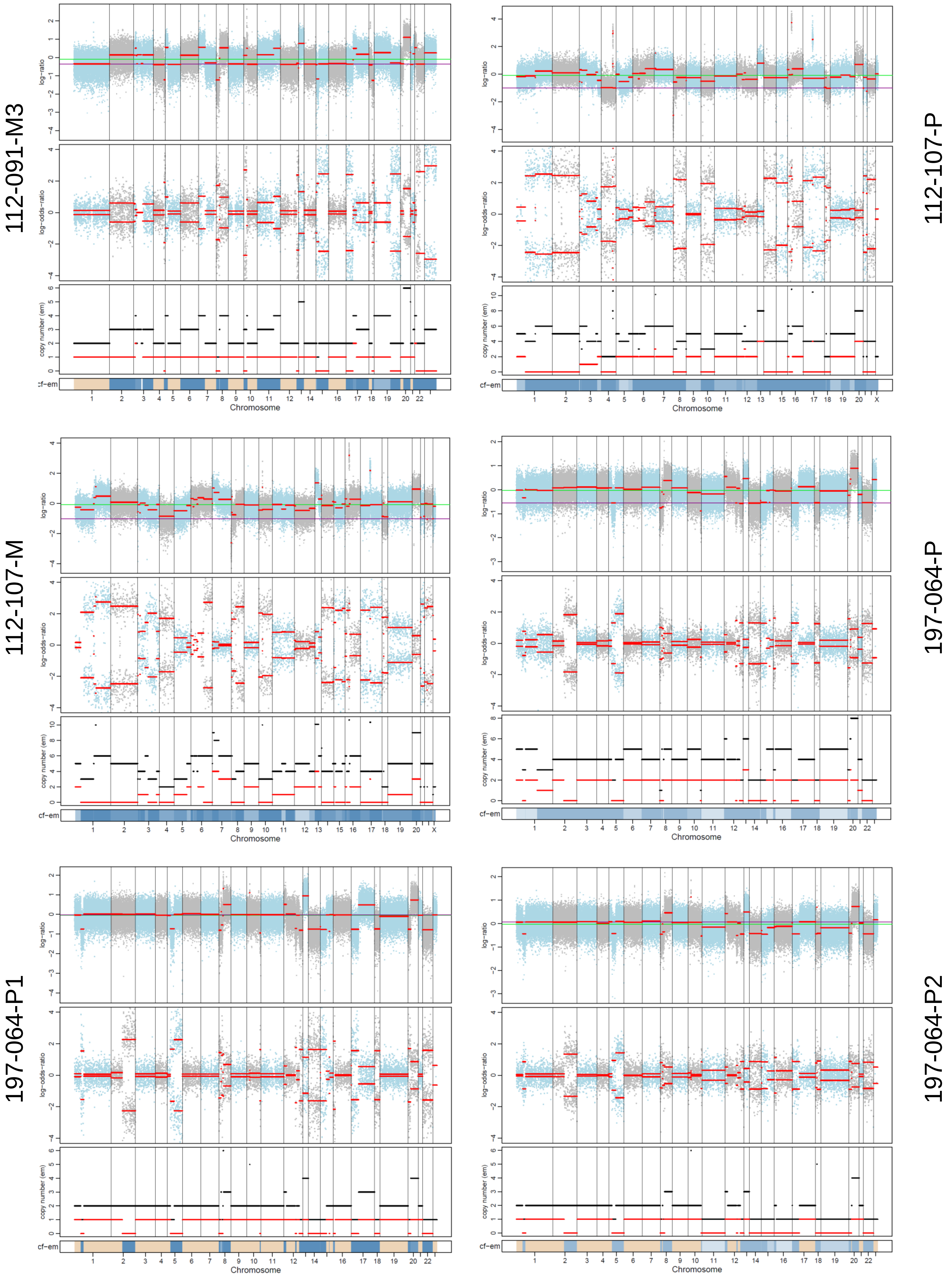
112-091-M3
112-107-P
112-107-M
197-064-P
197-064-P1
197-064-P2
log-ratio
log-odds-ratio
copy number (em)
cf-em
Chromosome

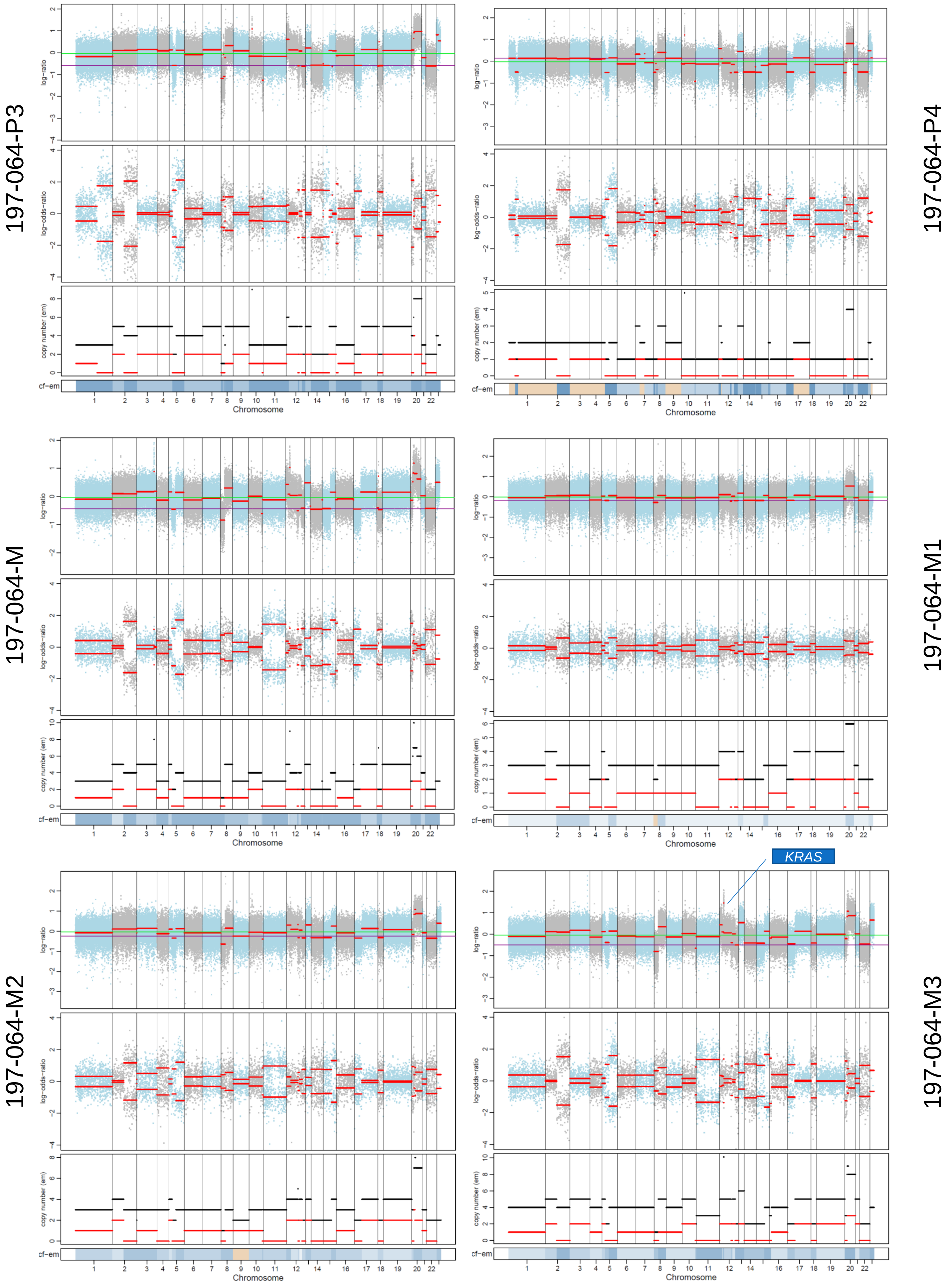
197-064-P3
197-064-P4
197-064-M
197-064-M1
197-064-M2
197-064-M3
KRAS
log-ratio
log-odds-ratio
copy number (em)
cf-em
Chromosome

197-064-M4

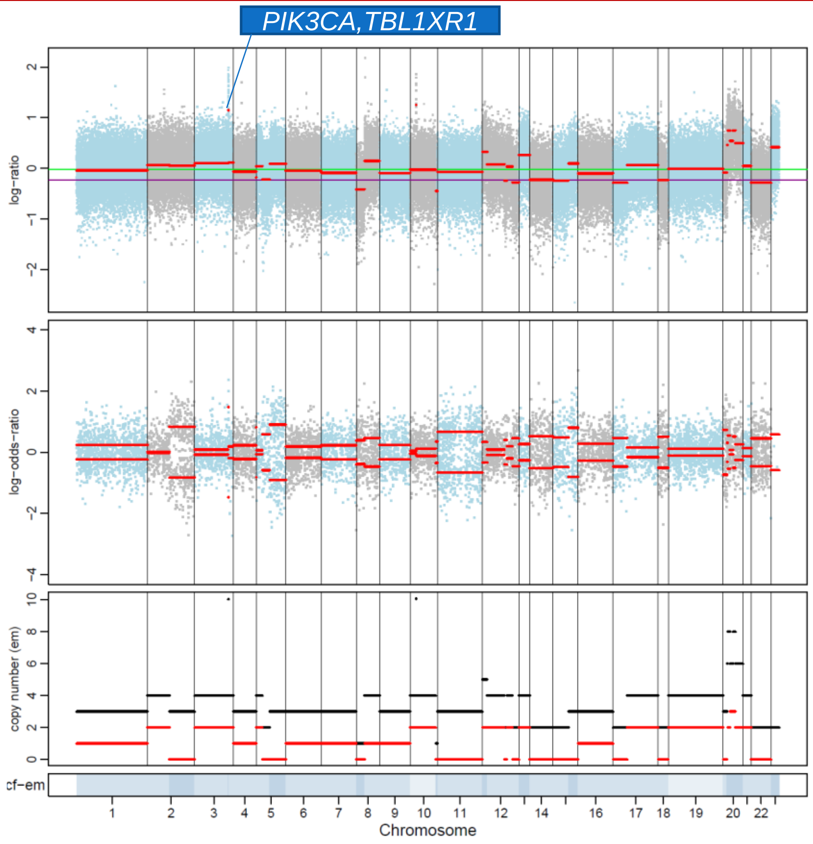

197-144-P

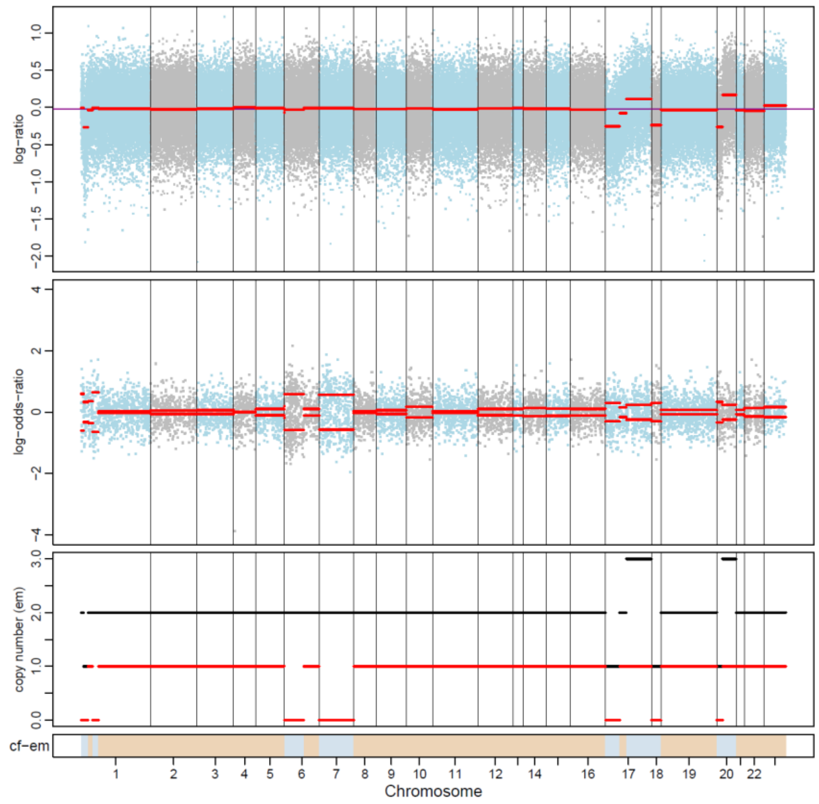

197-144-M

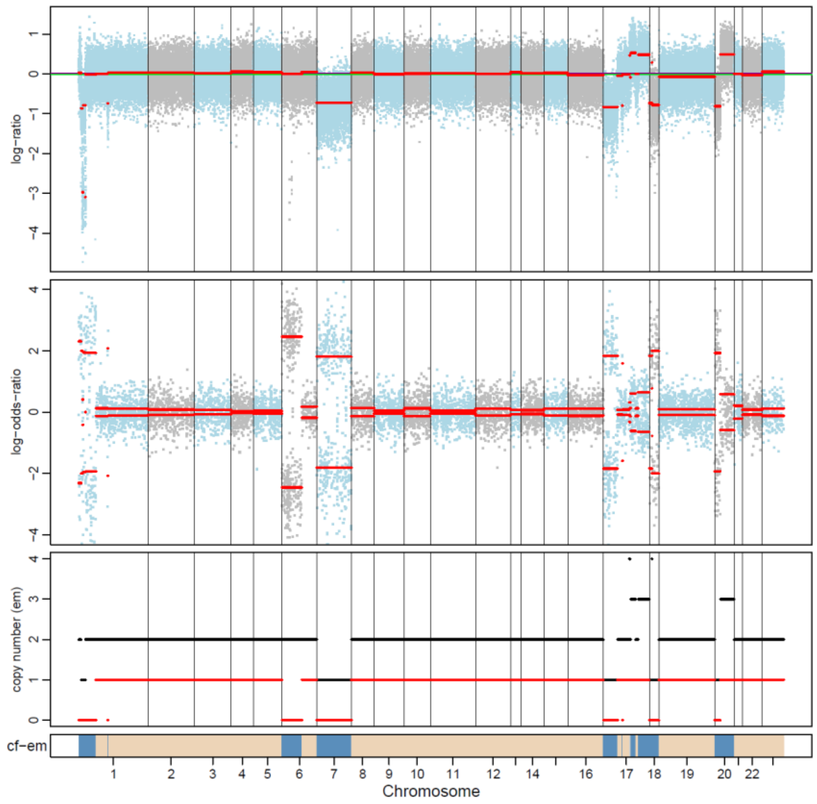

197-185-P

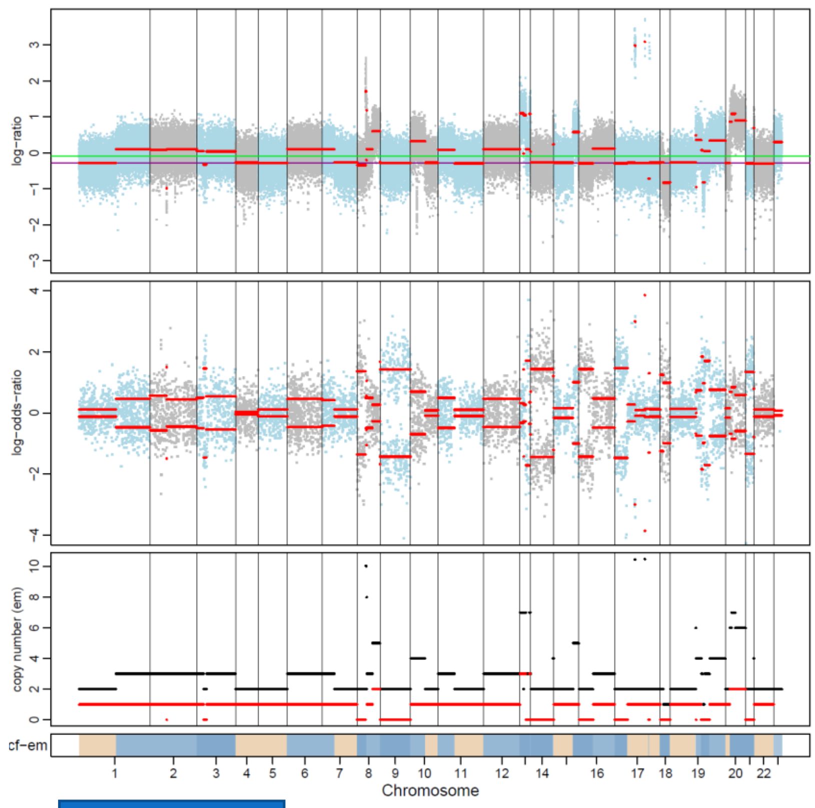

197-185-M

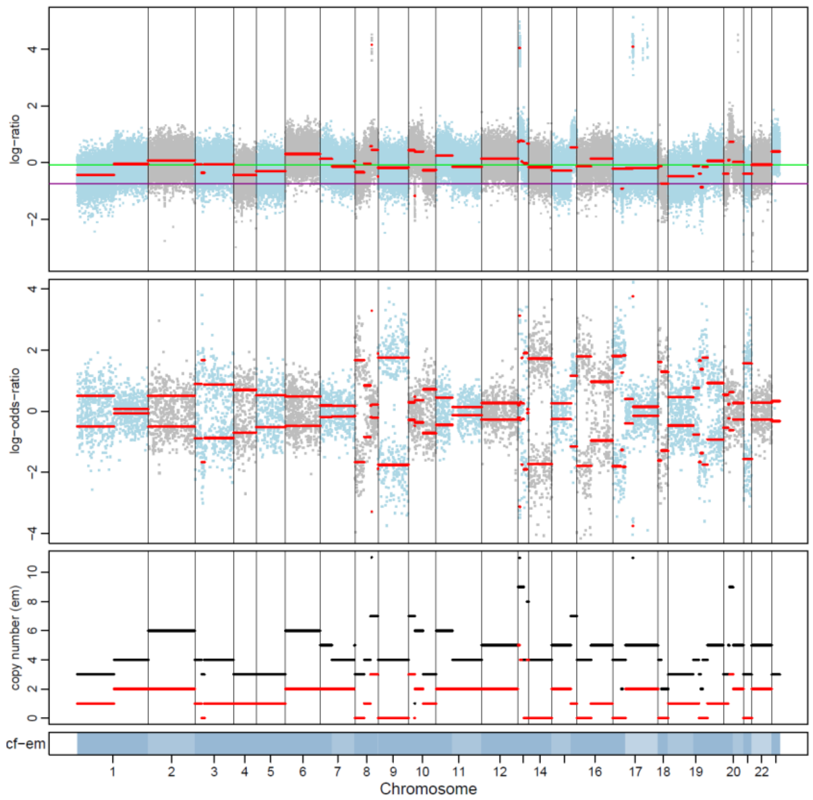

306-052-P

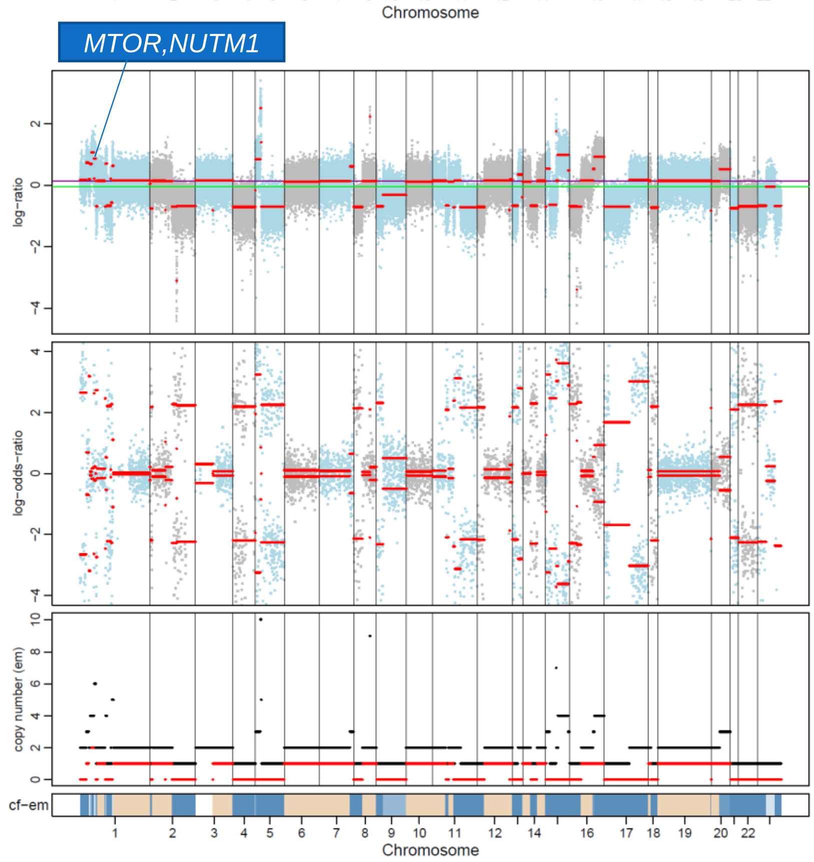

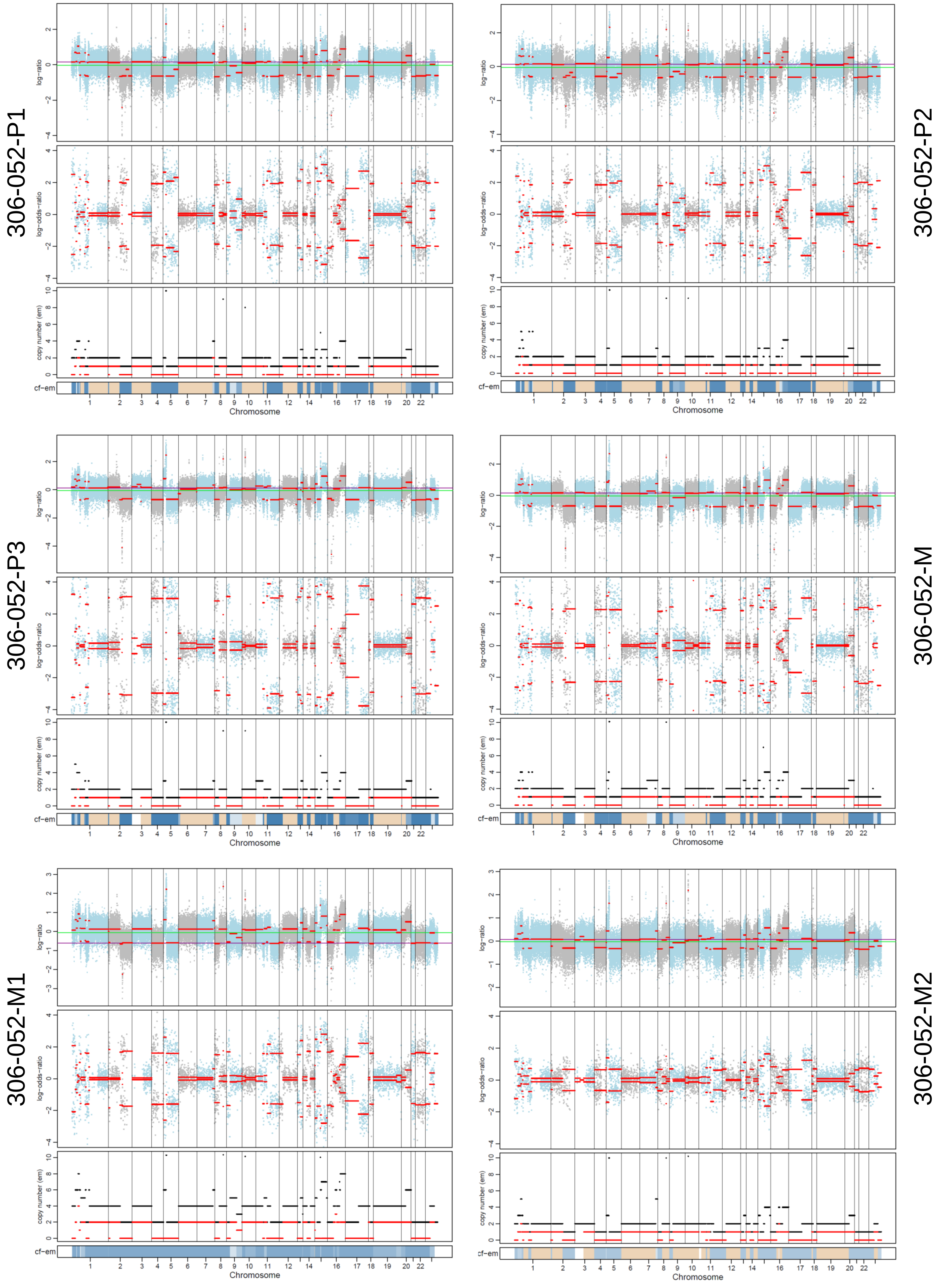
306-052-P1
306-052-P2
306-052-P3
306-052-M
306-052-M1
306-052-M2
log-ratio
log-odds-ratio
copy number (em)
cf-em
Chromosome

306-052-M3

306-090-P

306-090-P1

306-090-P2

306-090-P3

306-090-M

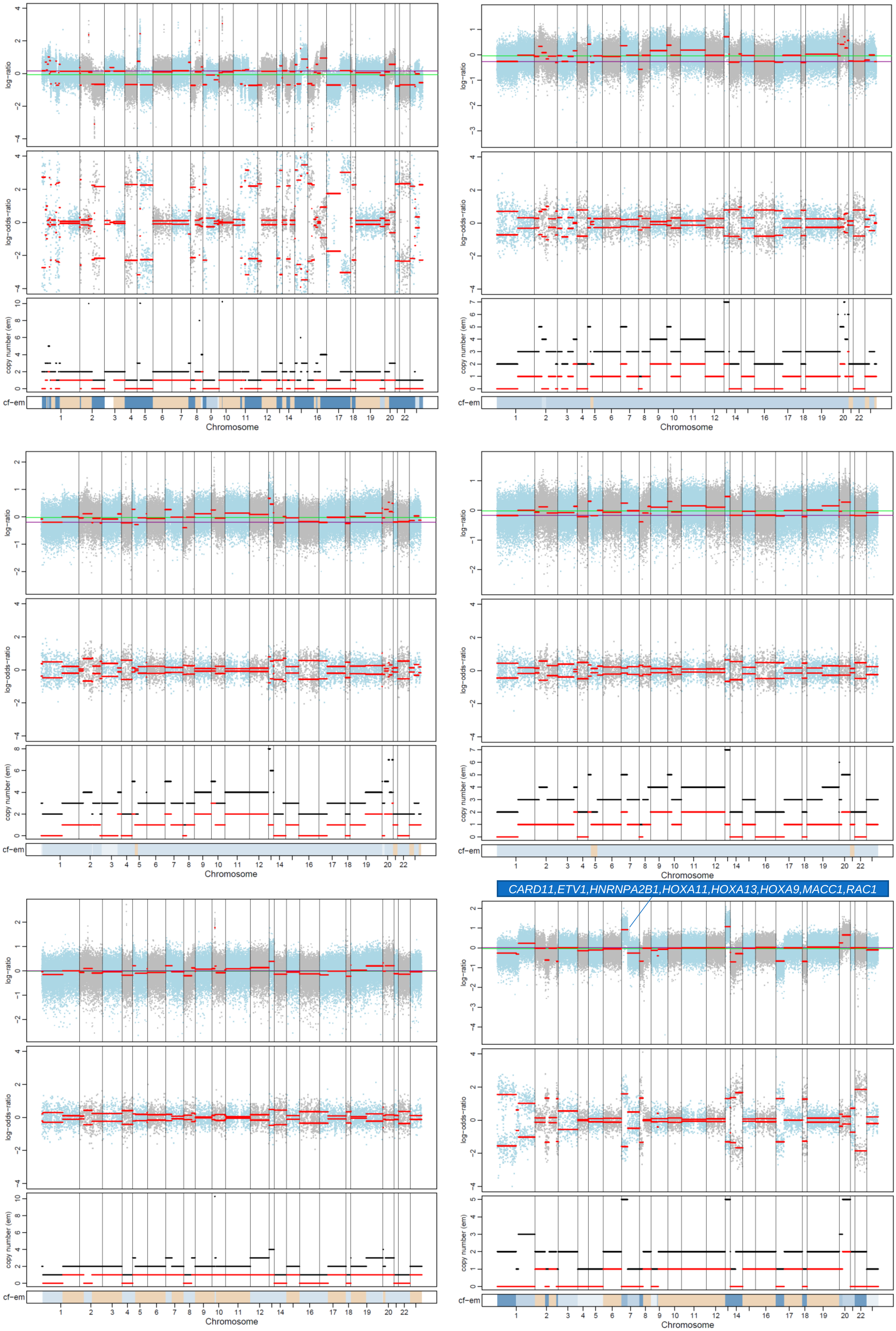

306-090-M1

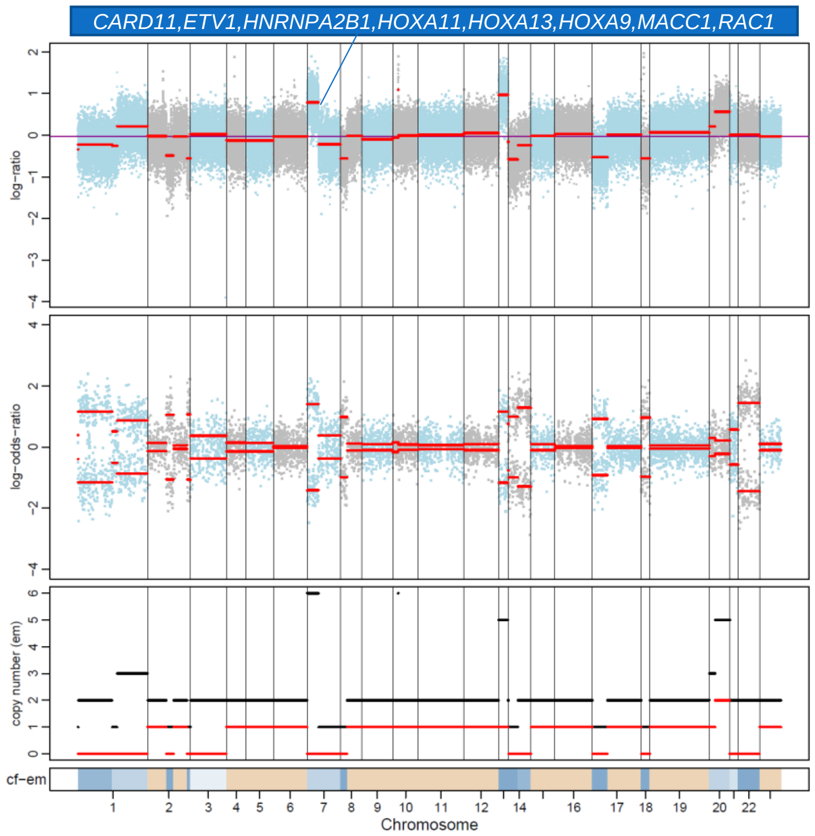

306-090-M2

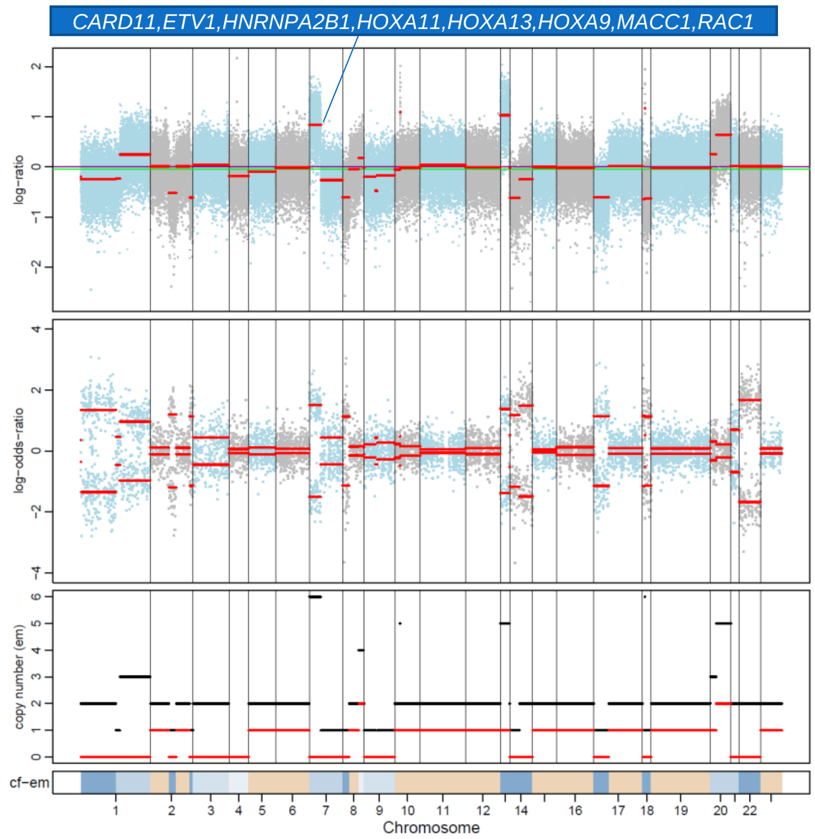

306-165-P

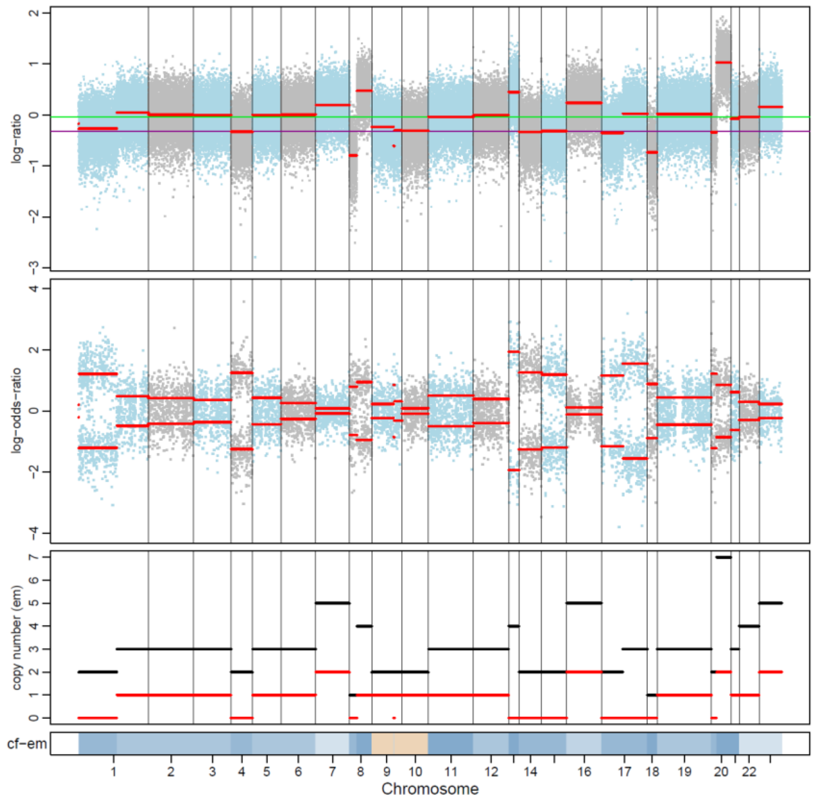

306-165-P1

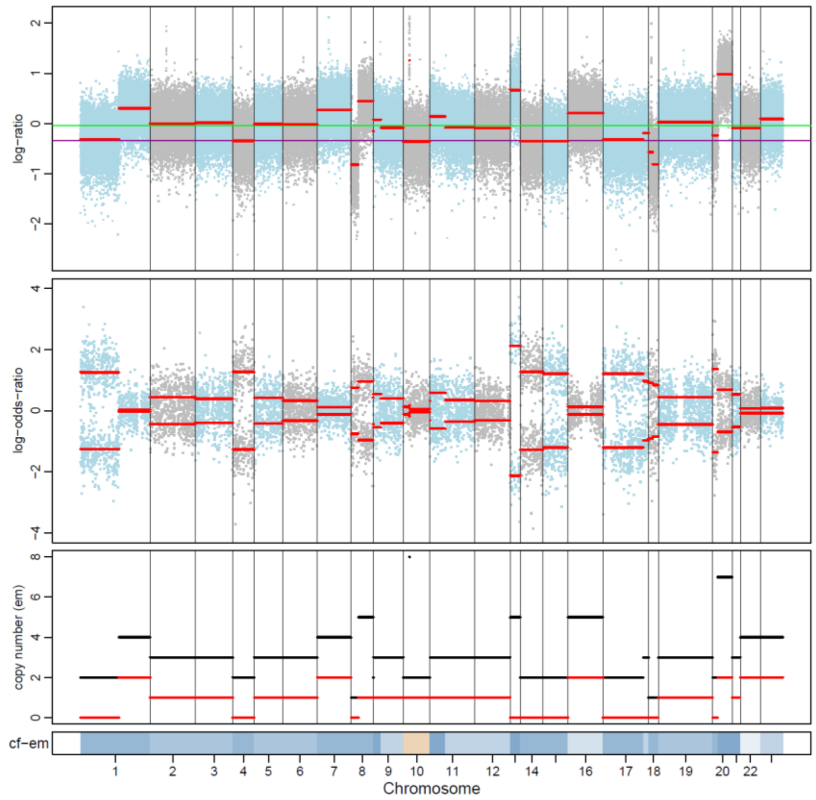

306-165-P2

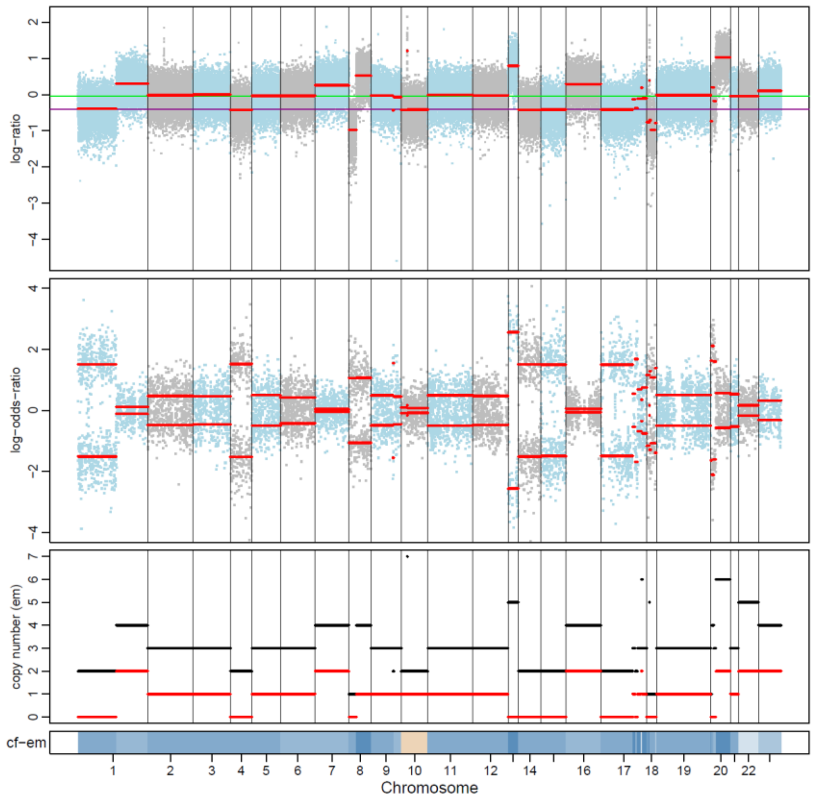

306-165-P3

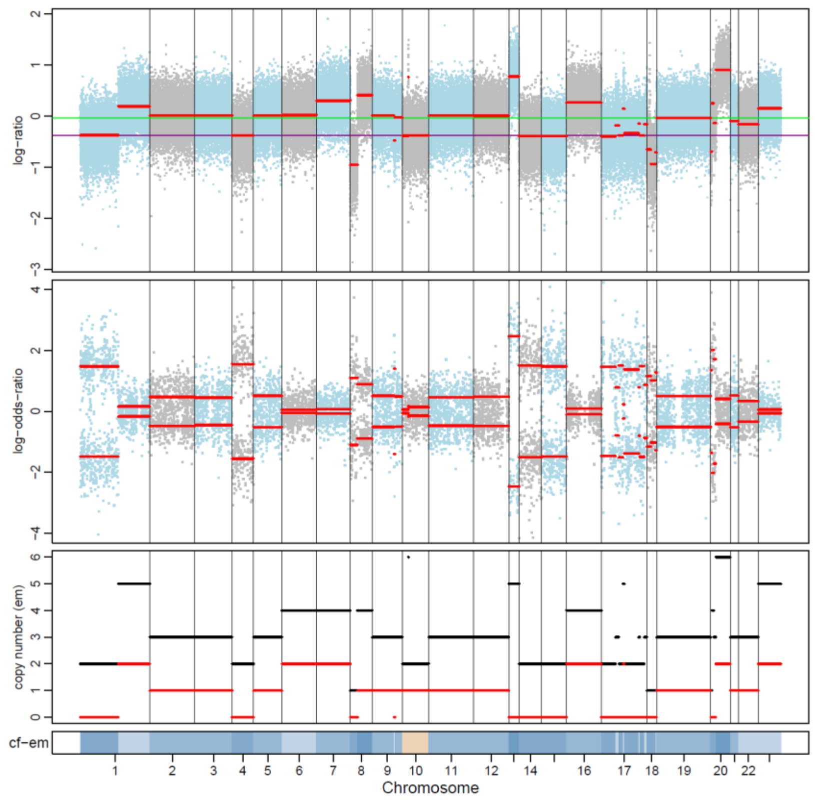

306-165-M

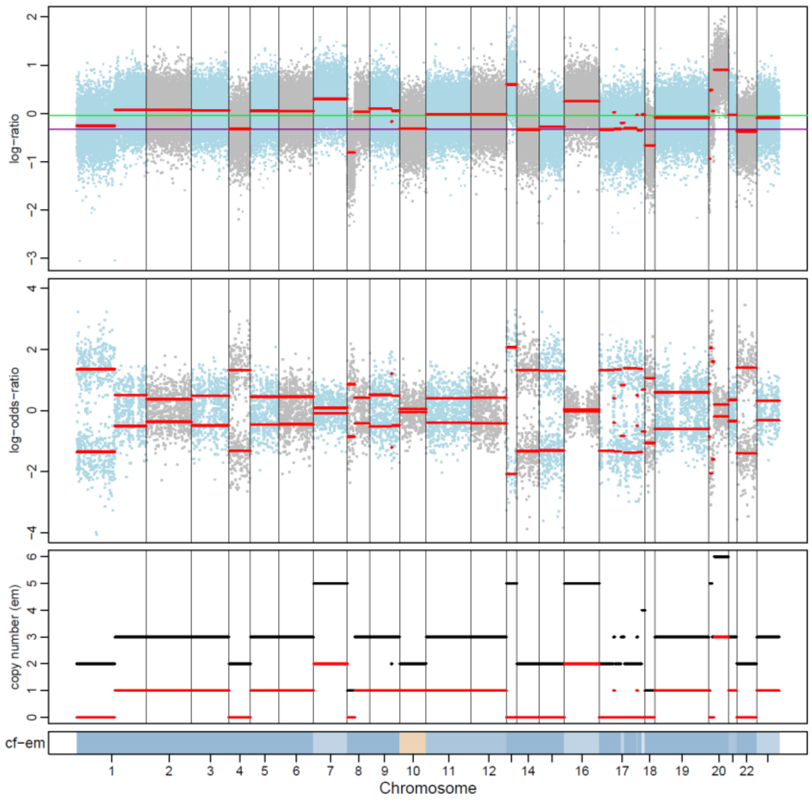

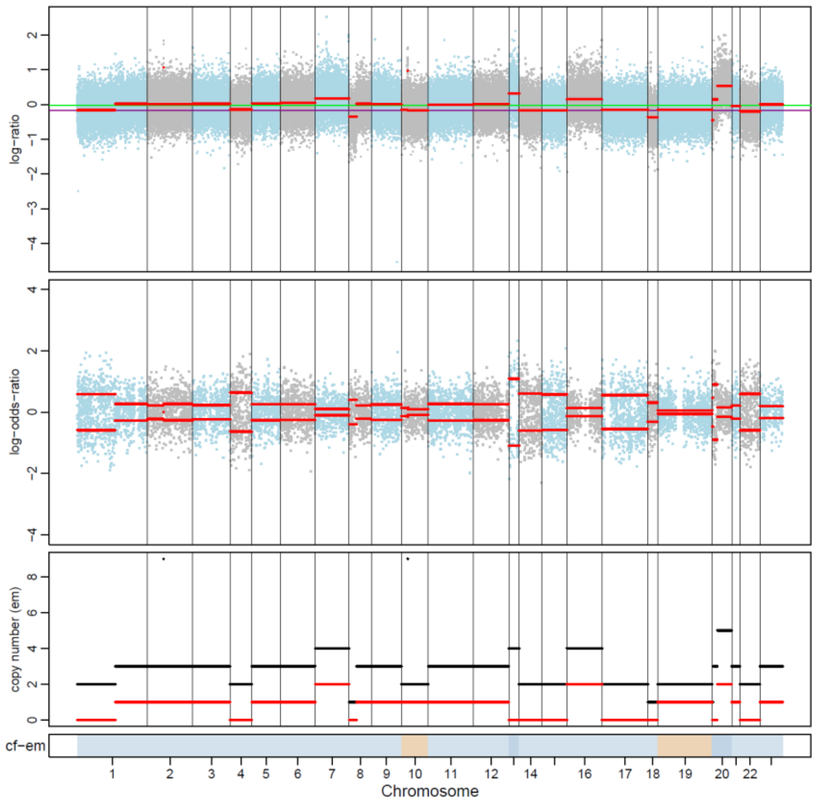

306-165-M1

306-165-M2

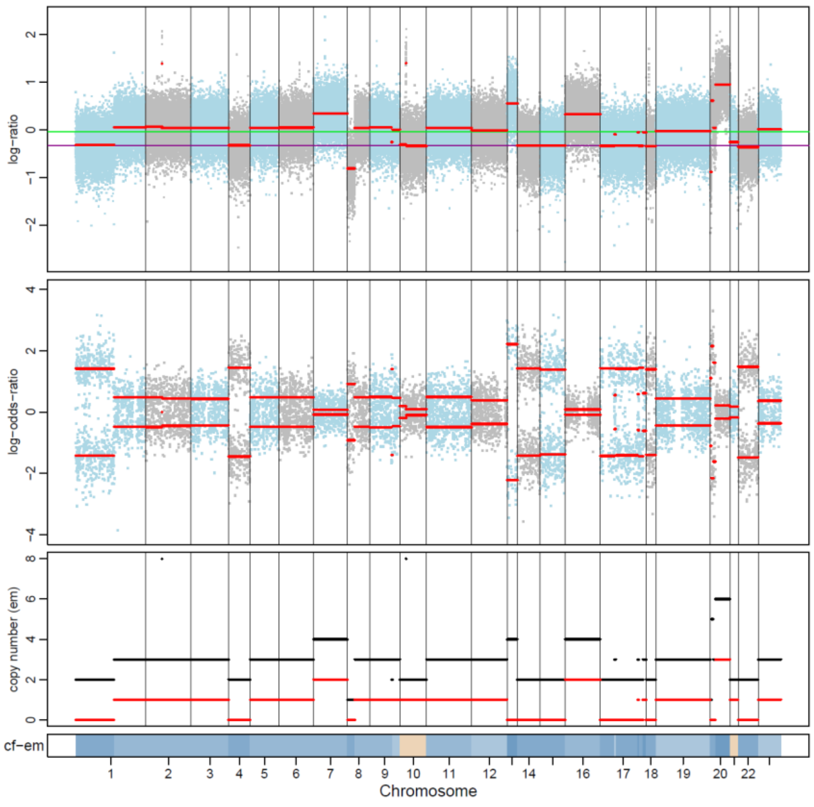

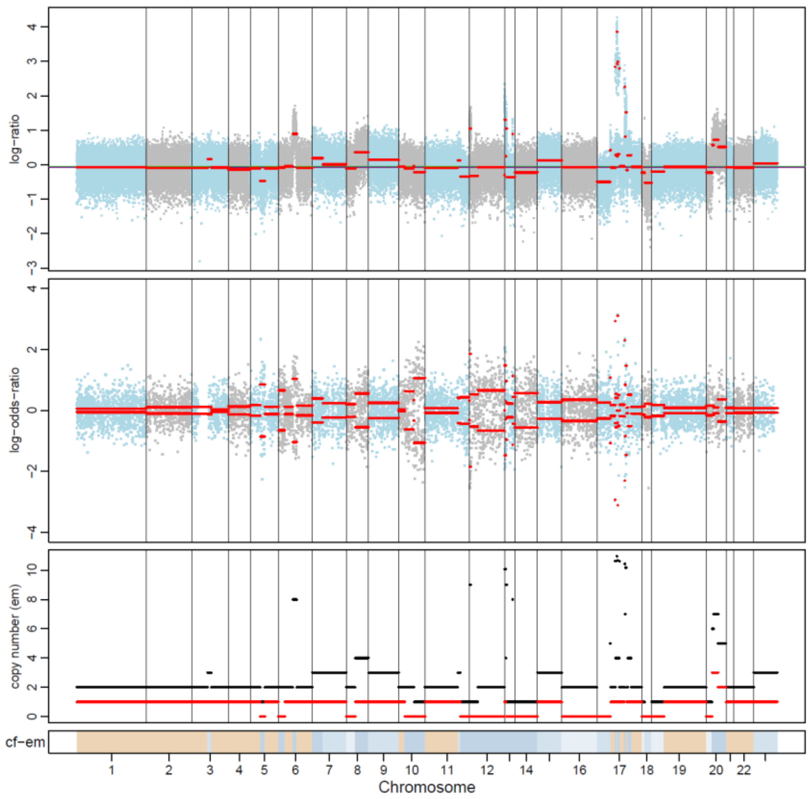

306-171-P

306-171-P1

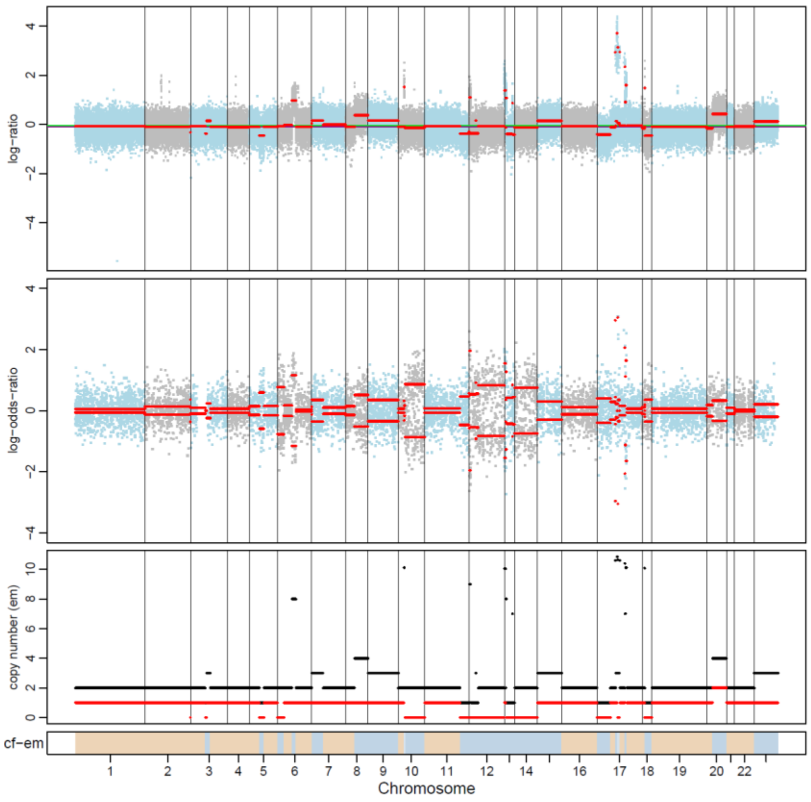

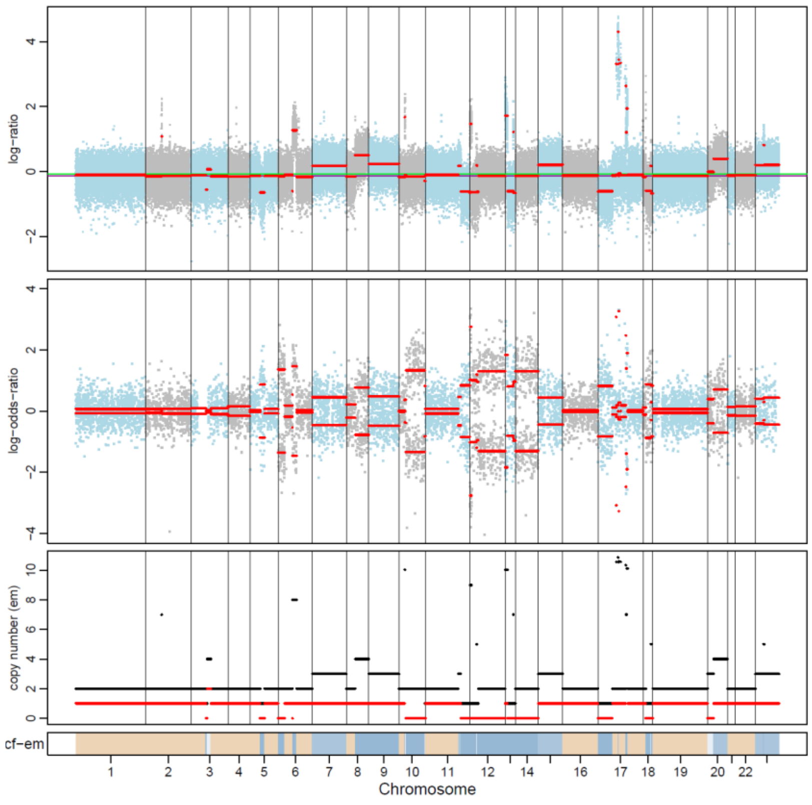

306-171-P2

306-171-P3

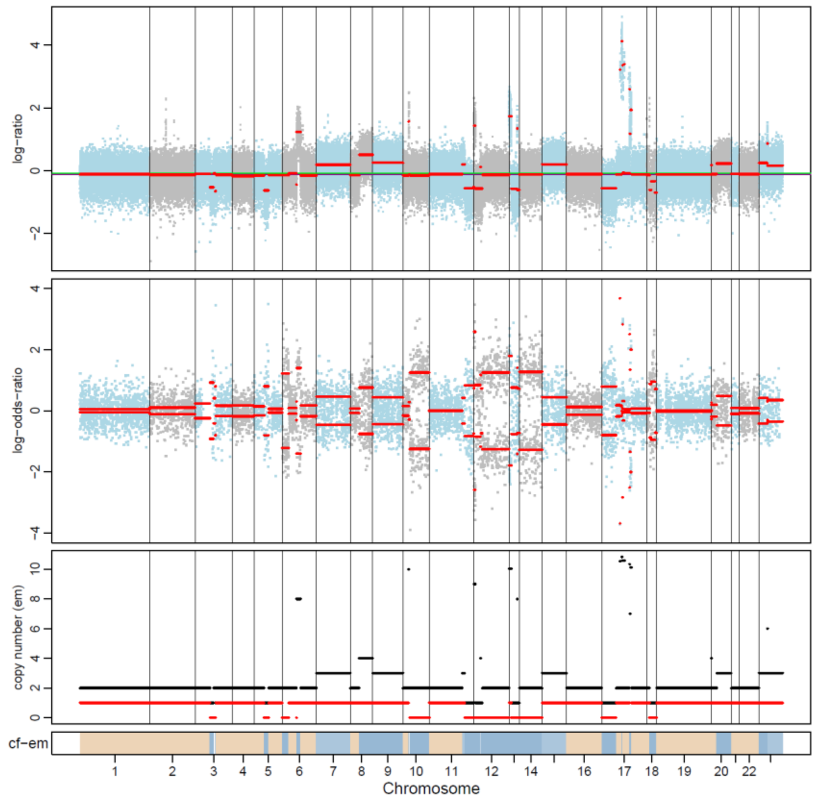

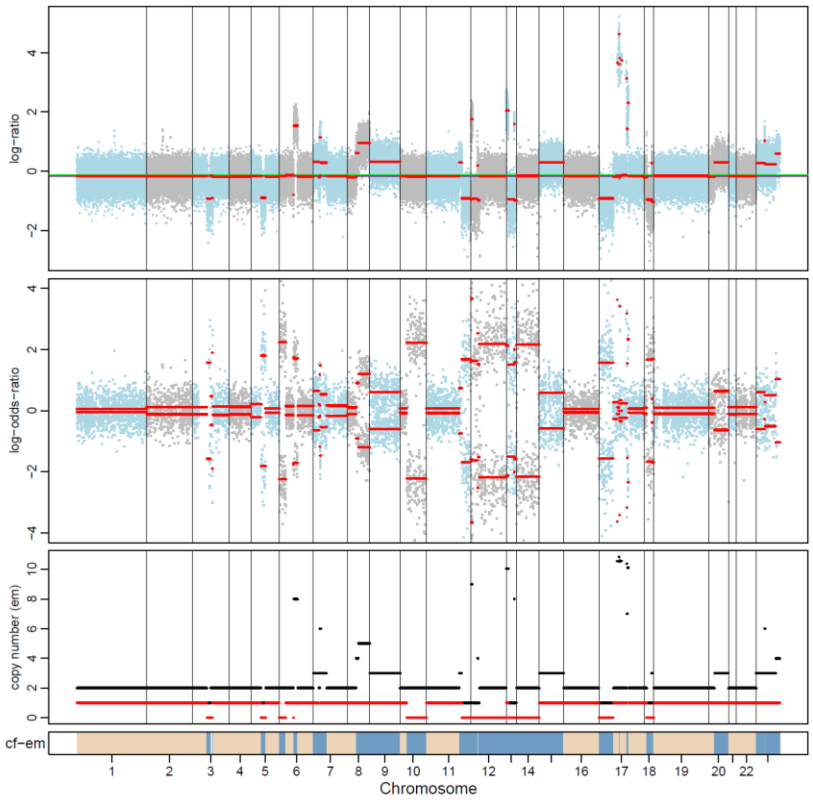

306-171-M

306-171-M1

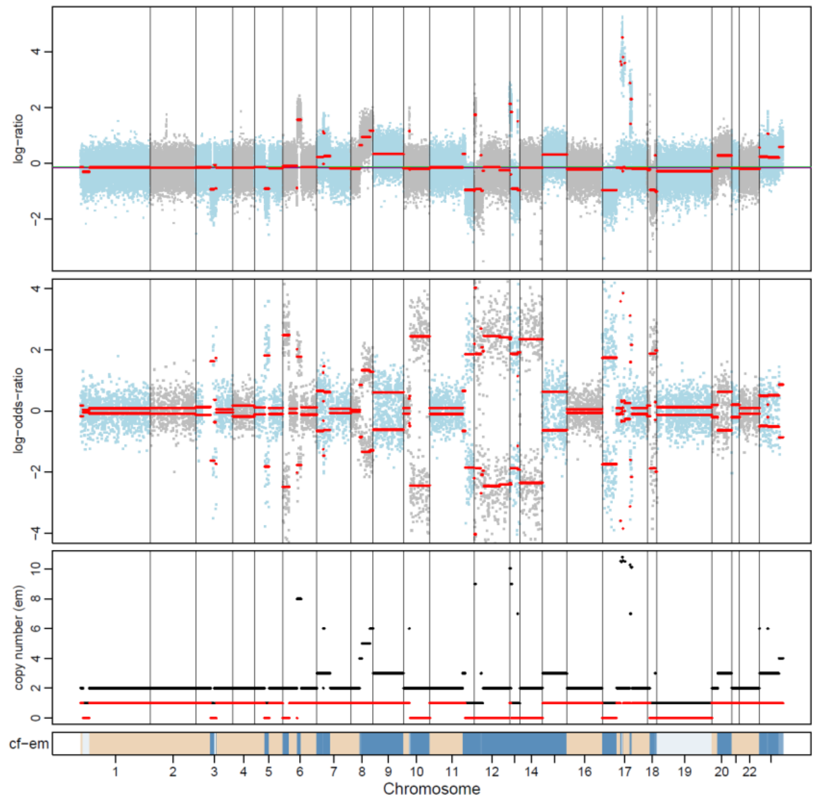

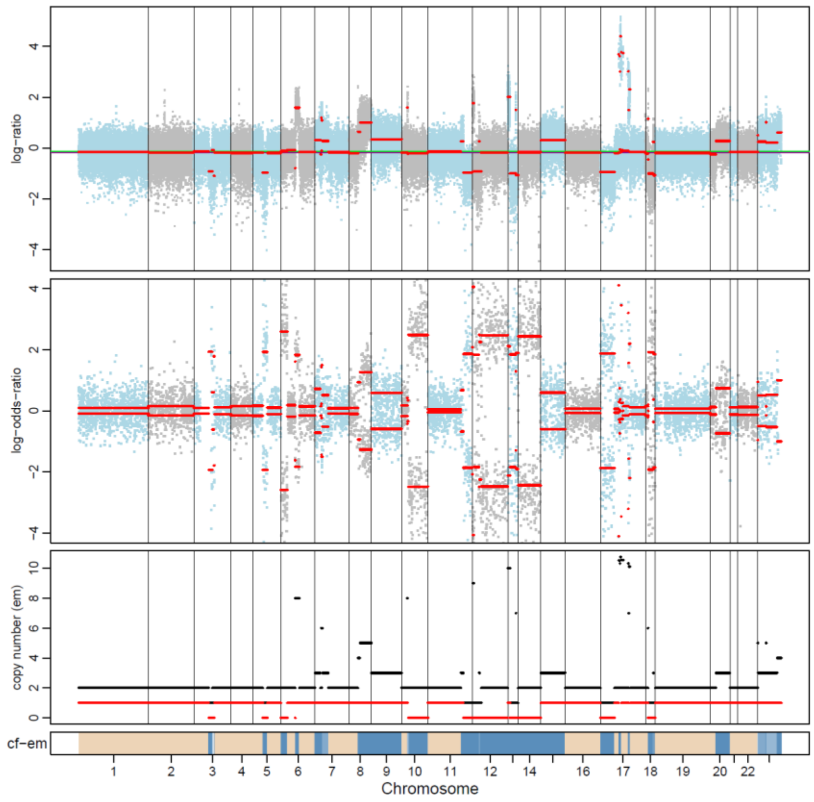

306-171-M2

306-171-M3

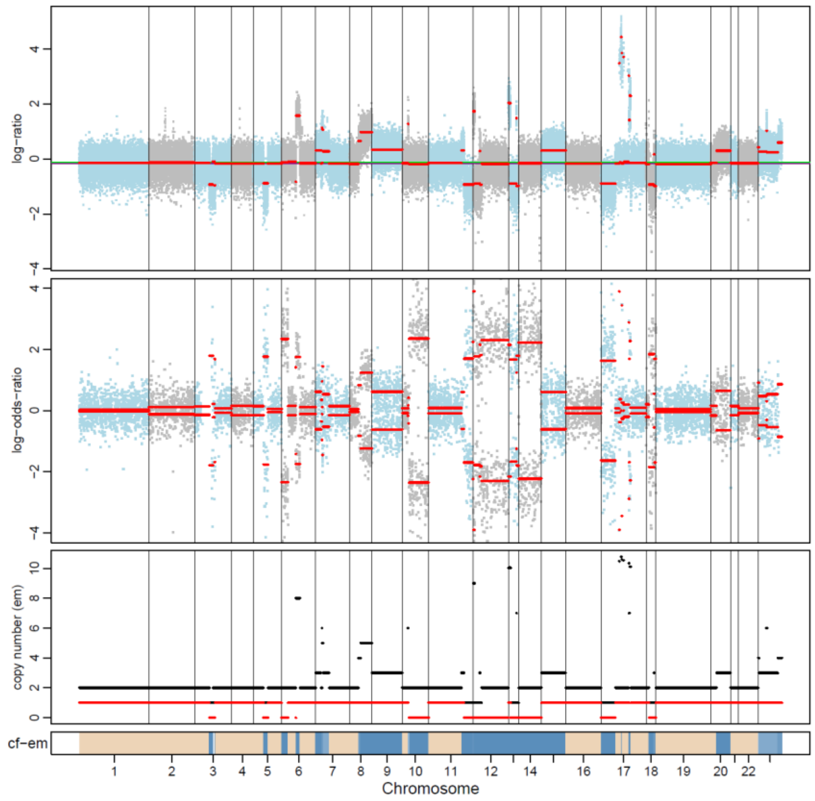

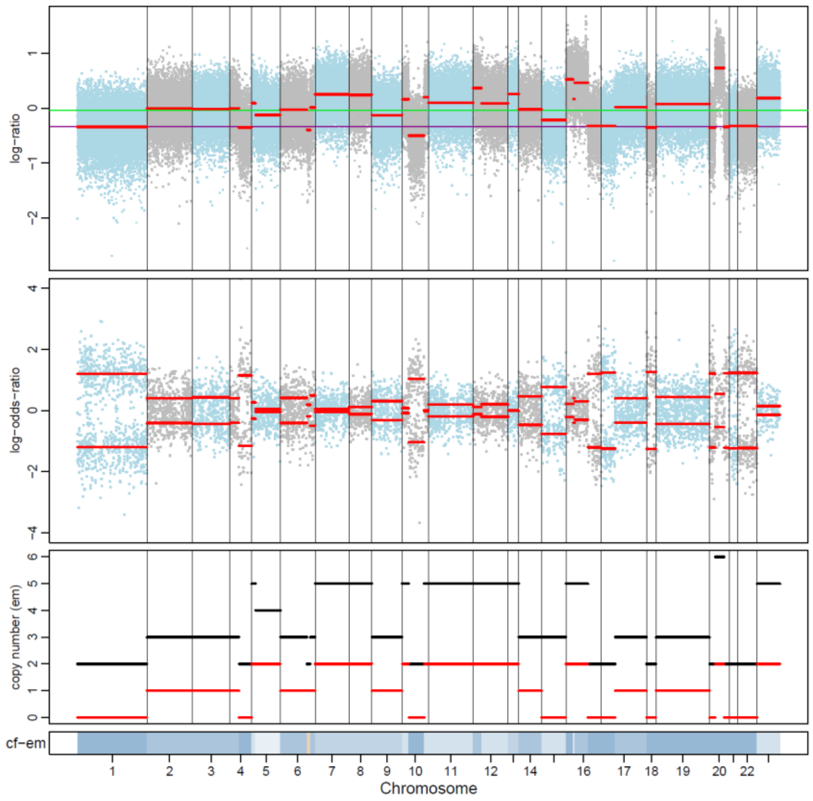

306-212-P

306-212-P1

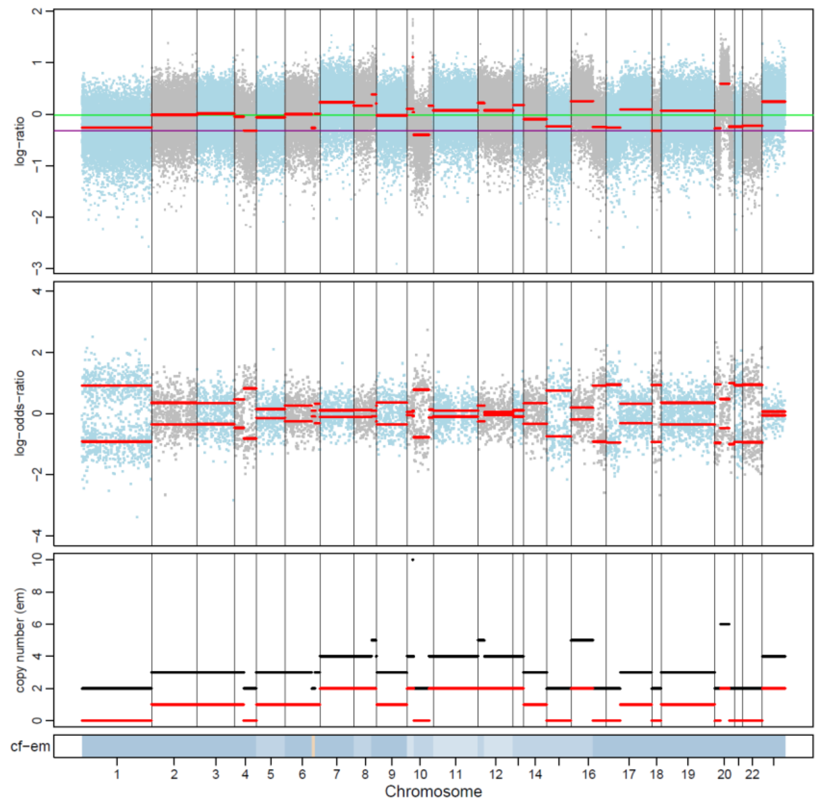

306-212-P2

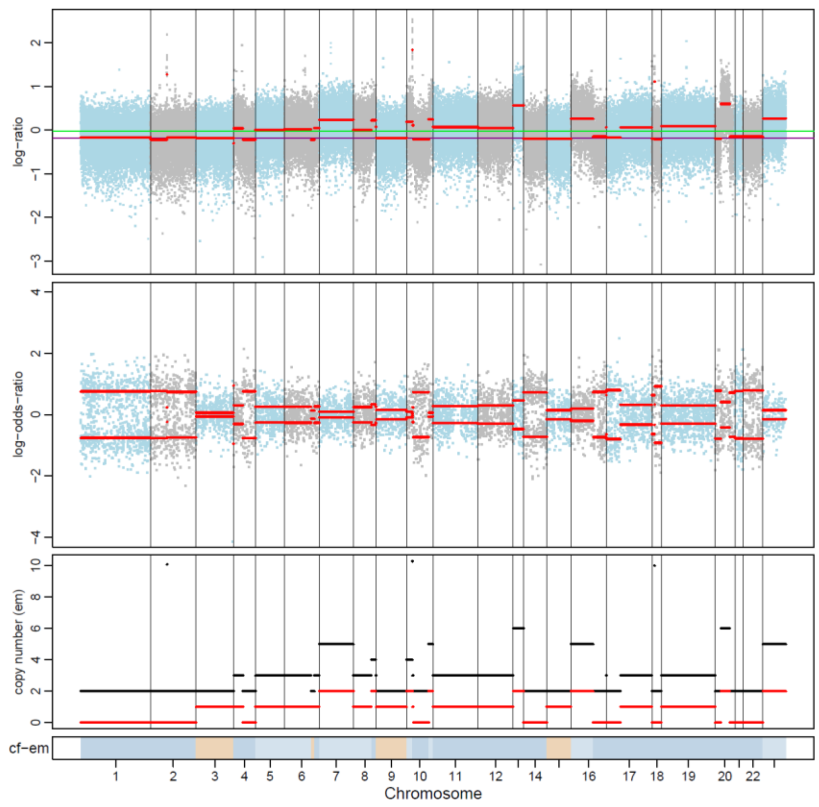

306-212-P3

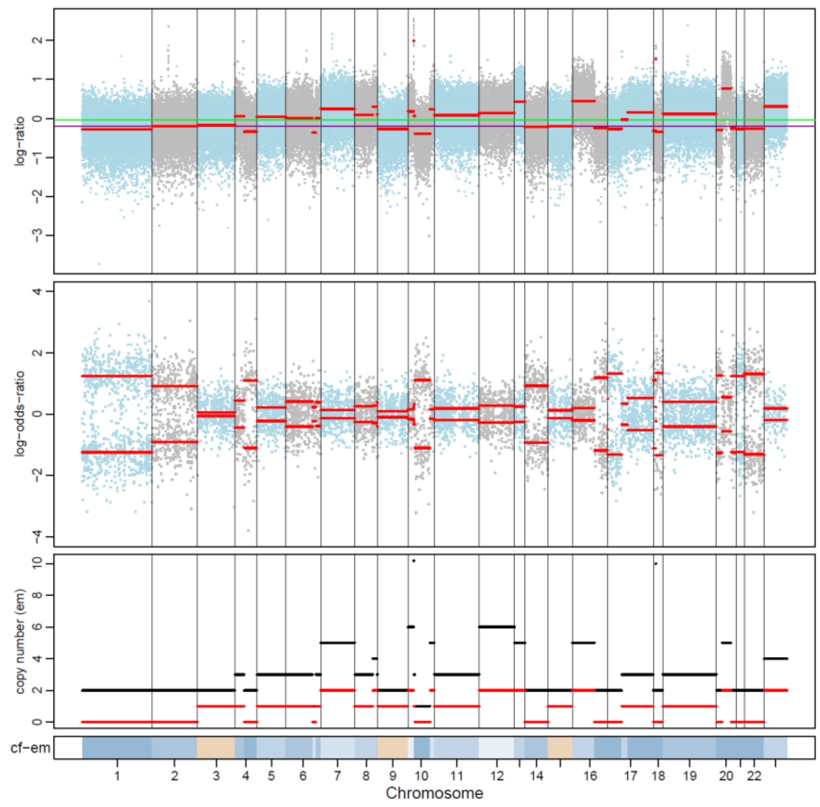

306-212-M

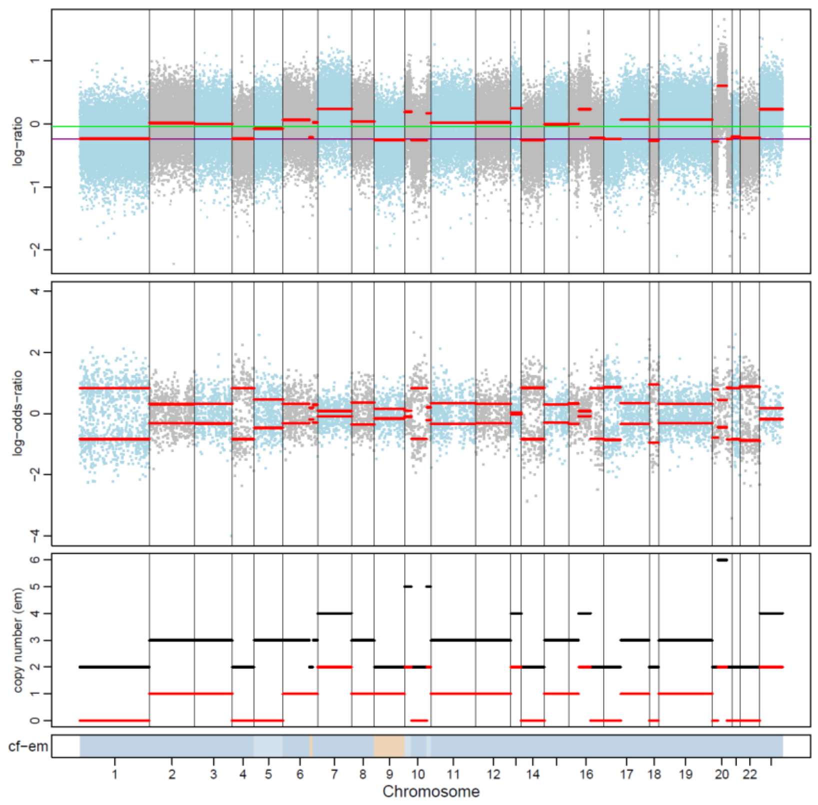

306-212-M1

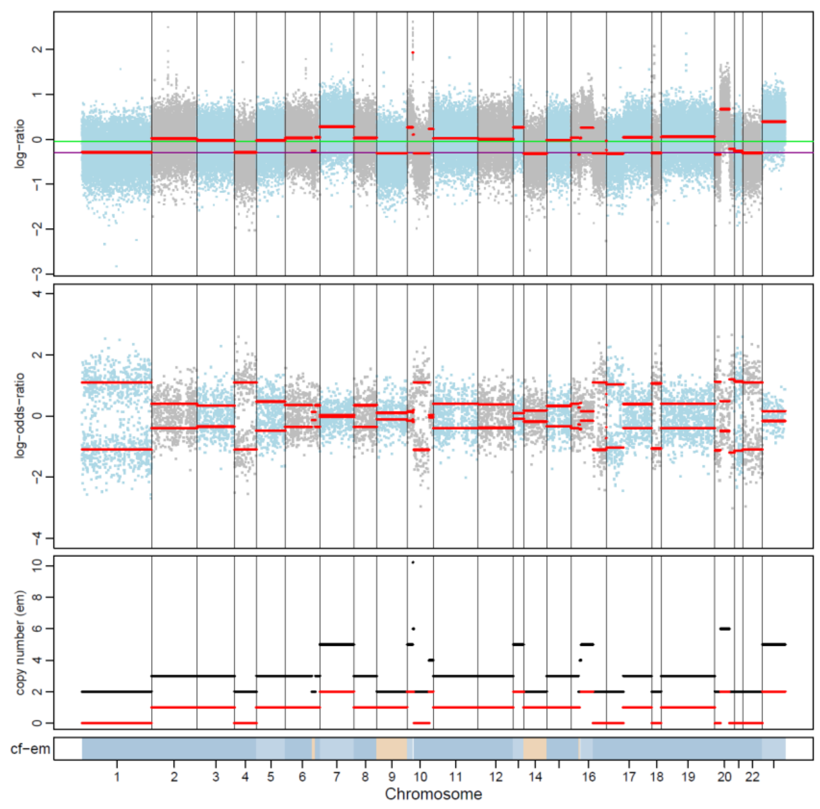

306-212-M2

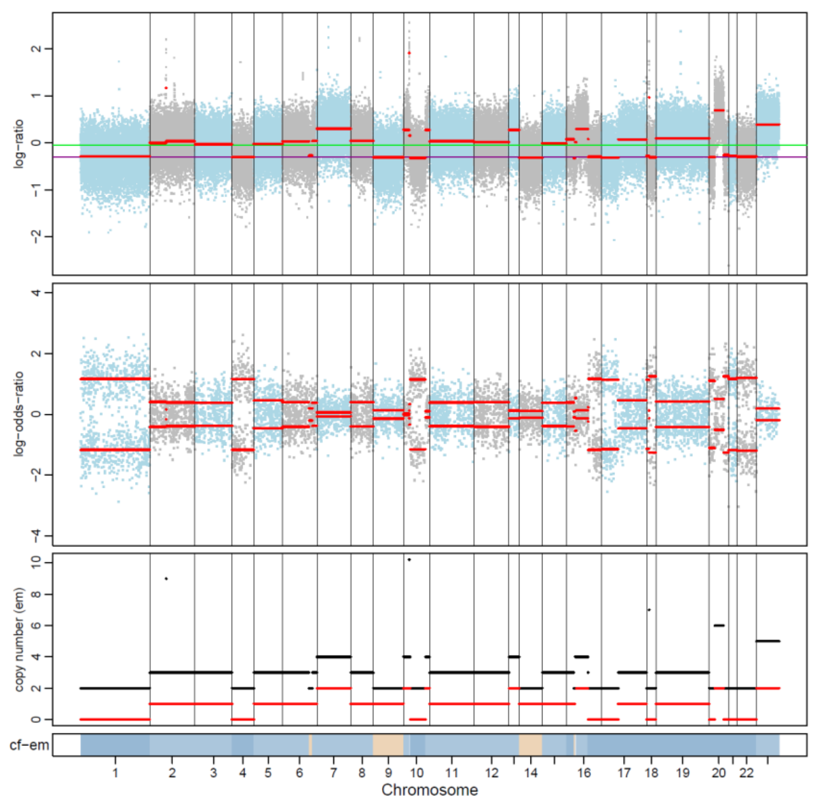

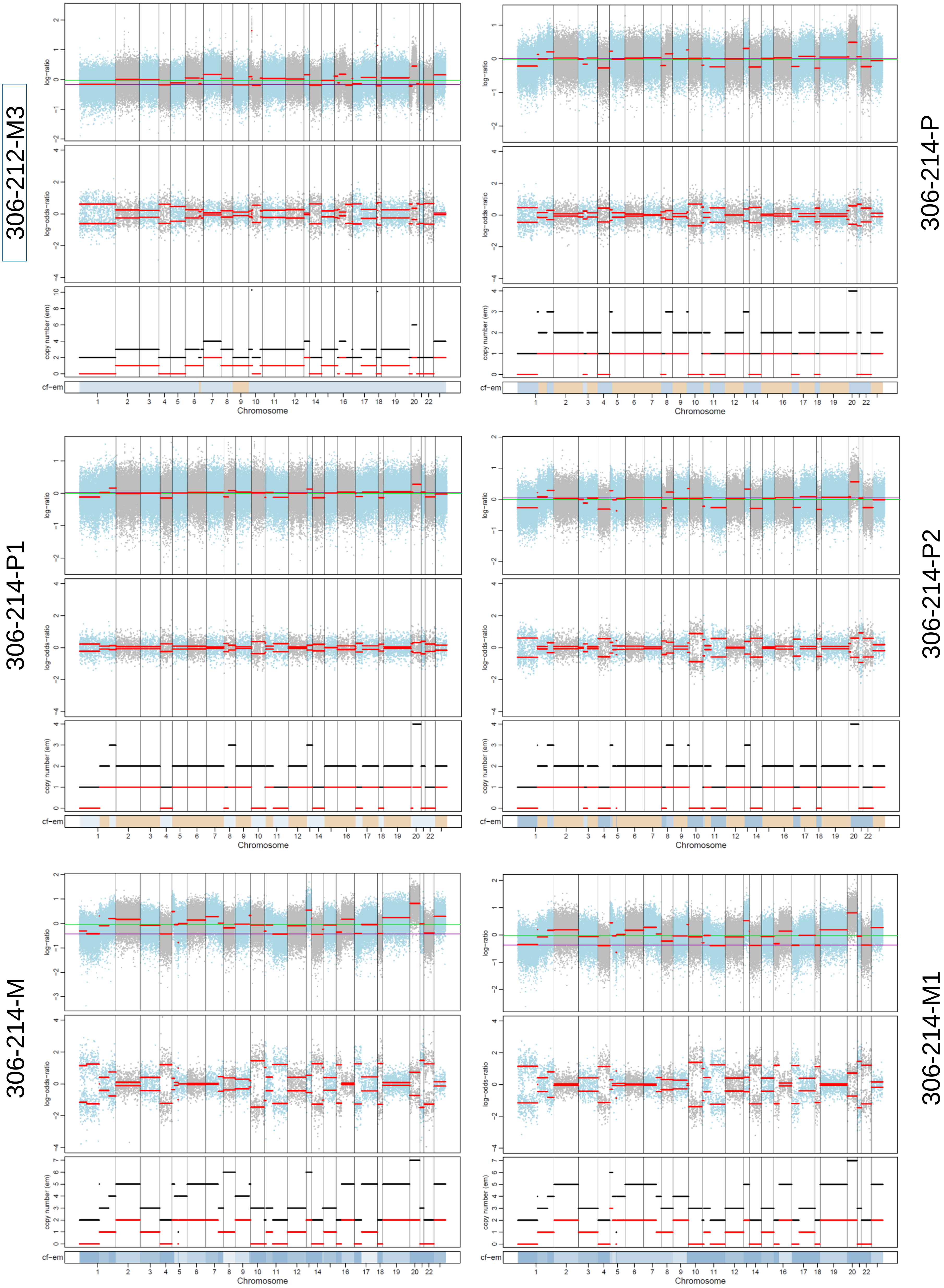
306-212-M3
306-214-P
306-214-P1
306-214-P2
306-214-M
306-214-M1
log-ratio
log-odds-ratio
copy number (em)
cf-em
Chromosome

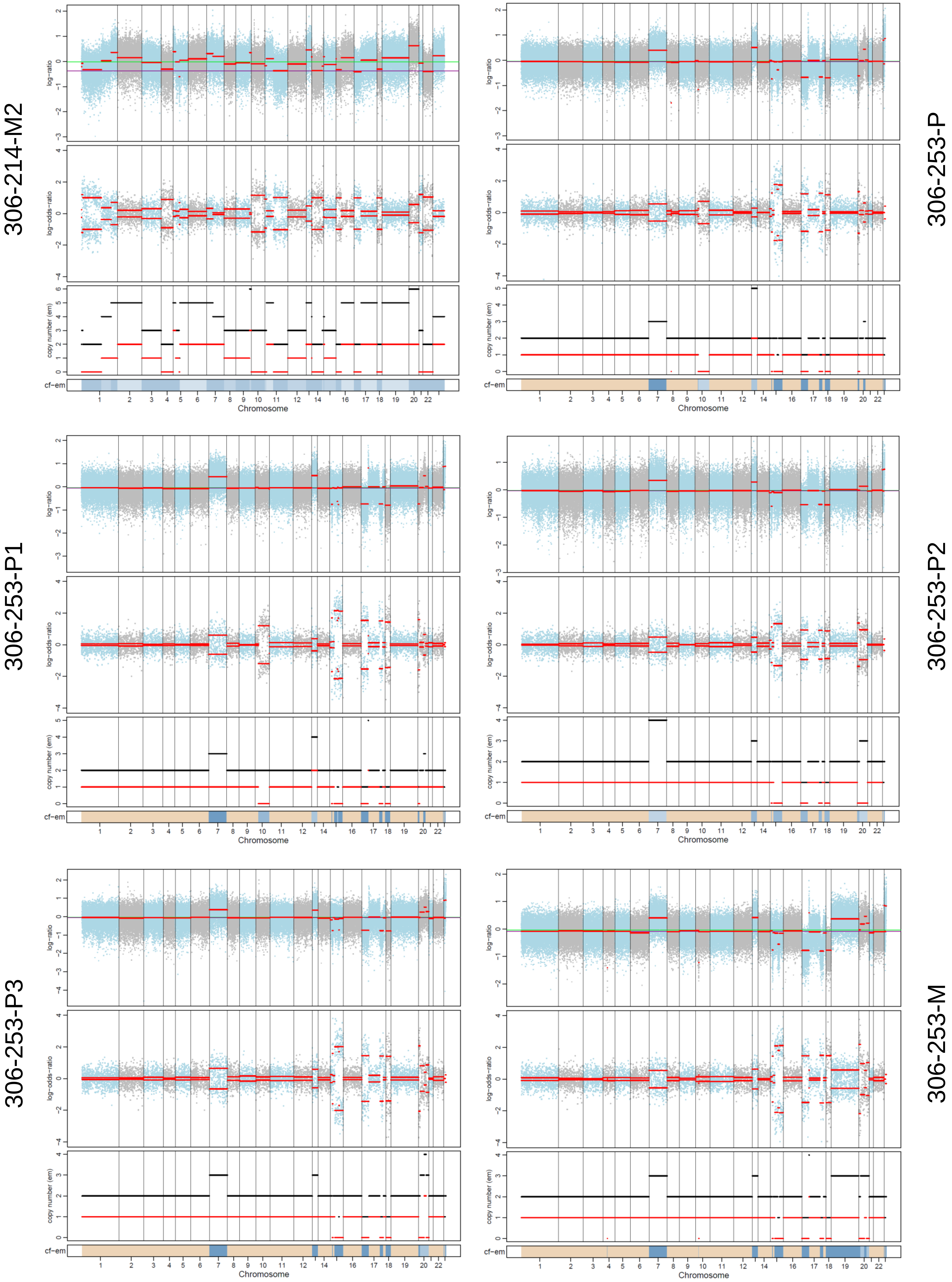
306-214-M2
306-253-P
306-253-P1
306-253-P2
306-253-P3
306-253-M
log-ratio
log-odds-ratio
copy number (em)
cf-em
Chromosome

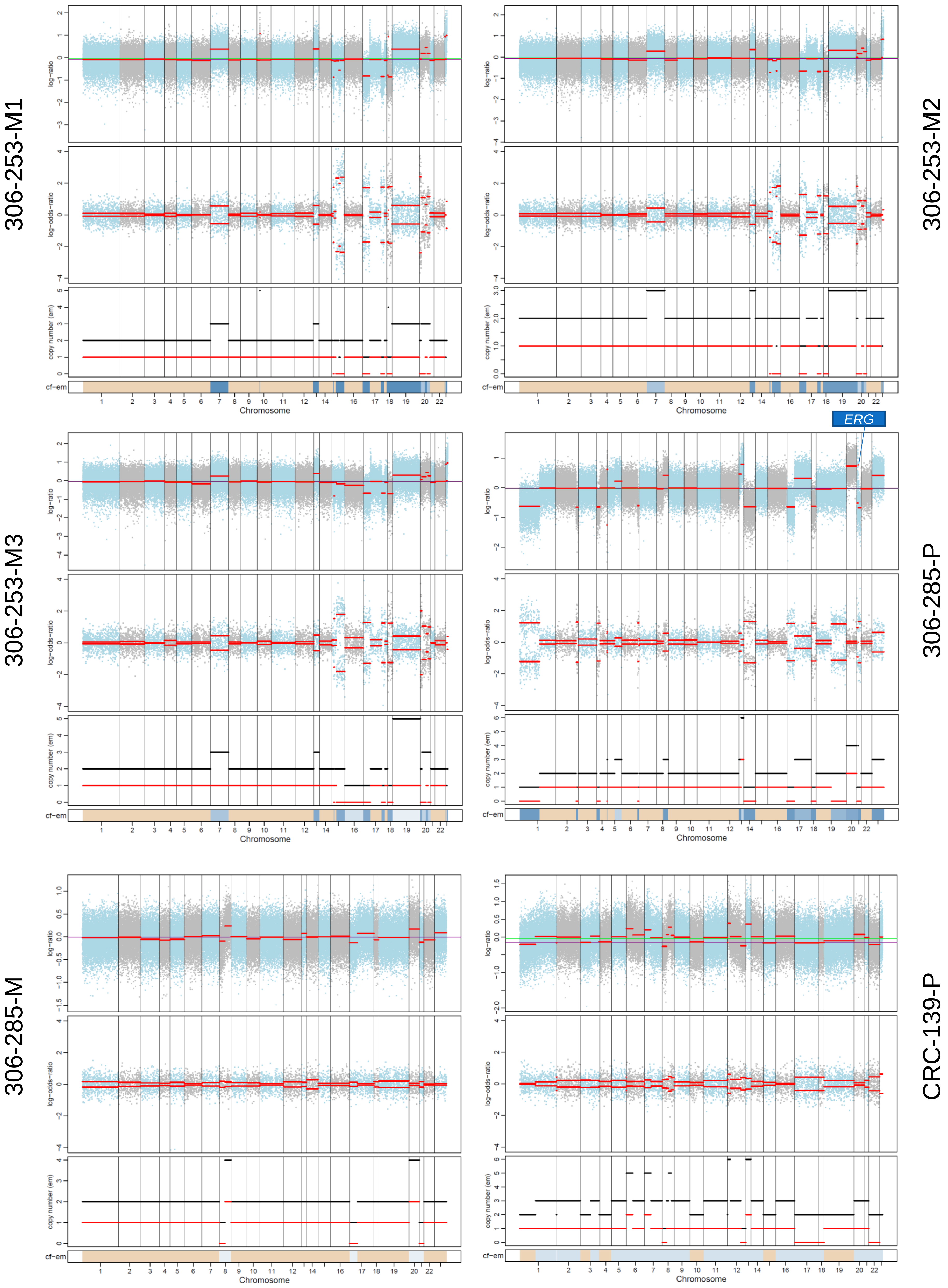
306-253-M1
306-253-M2
306-253-M3
306-285-P
306-285-M
CRC-139-P
ERG
log-ratio
log-odds-ratio
copy number (em)
cf-em
Chromosome

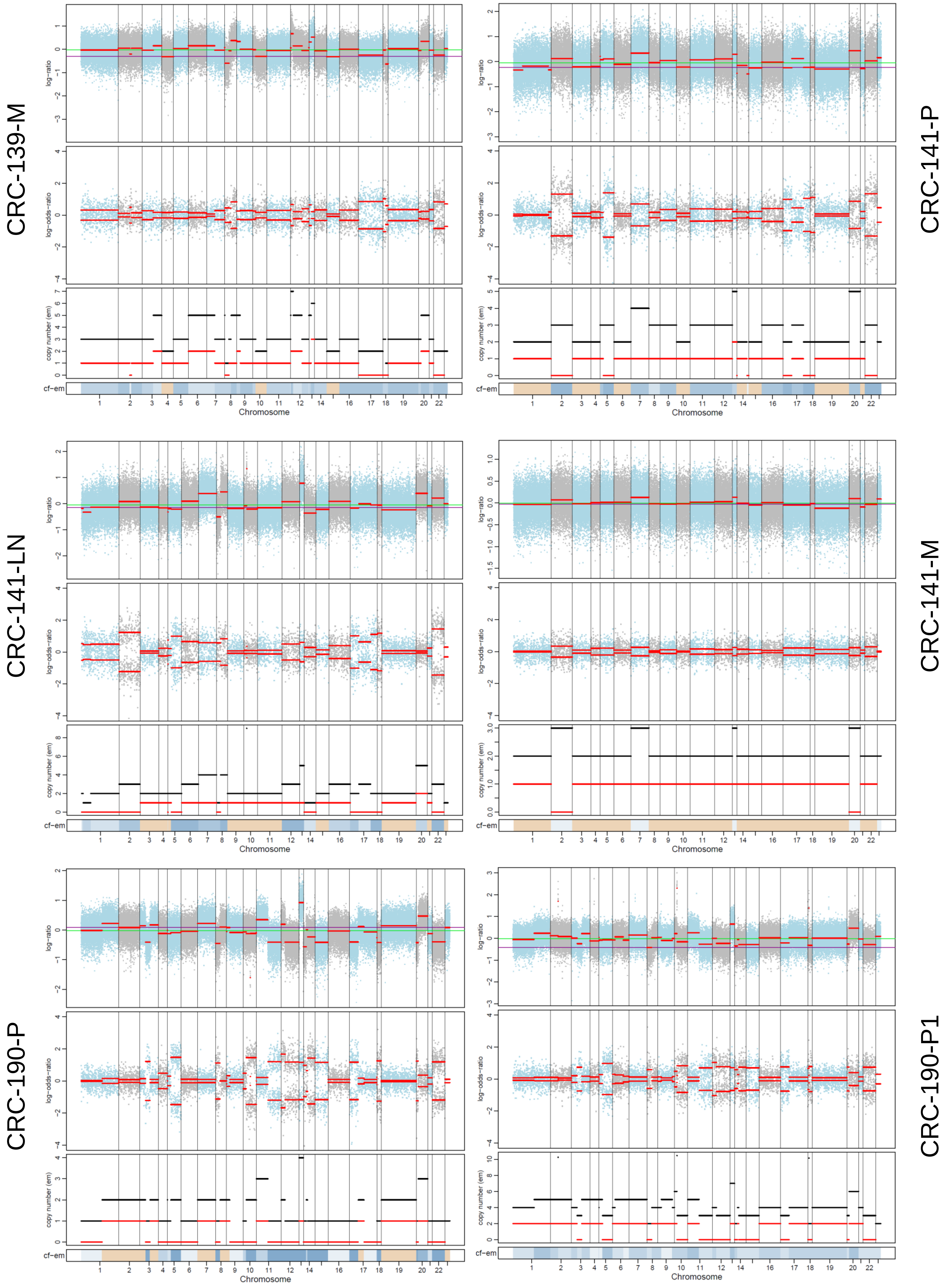
CRC-139-M
CRC-141-P
CRC-141-LN
CRC-141-M
CRC-190-P
CRC-190-P1
log-ratio
log-odds-ratio
copy number (em)
cf-em
Chromosome

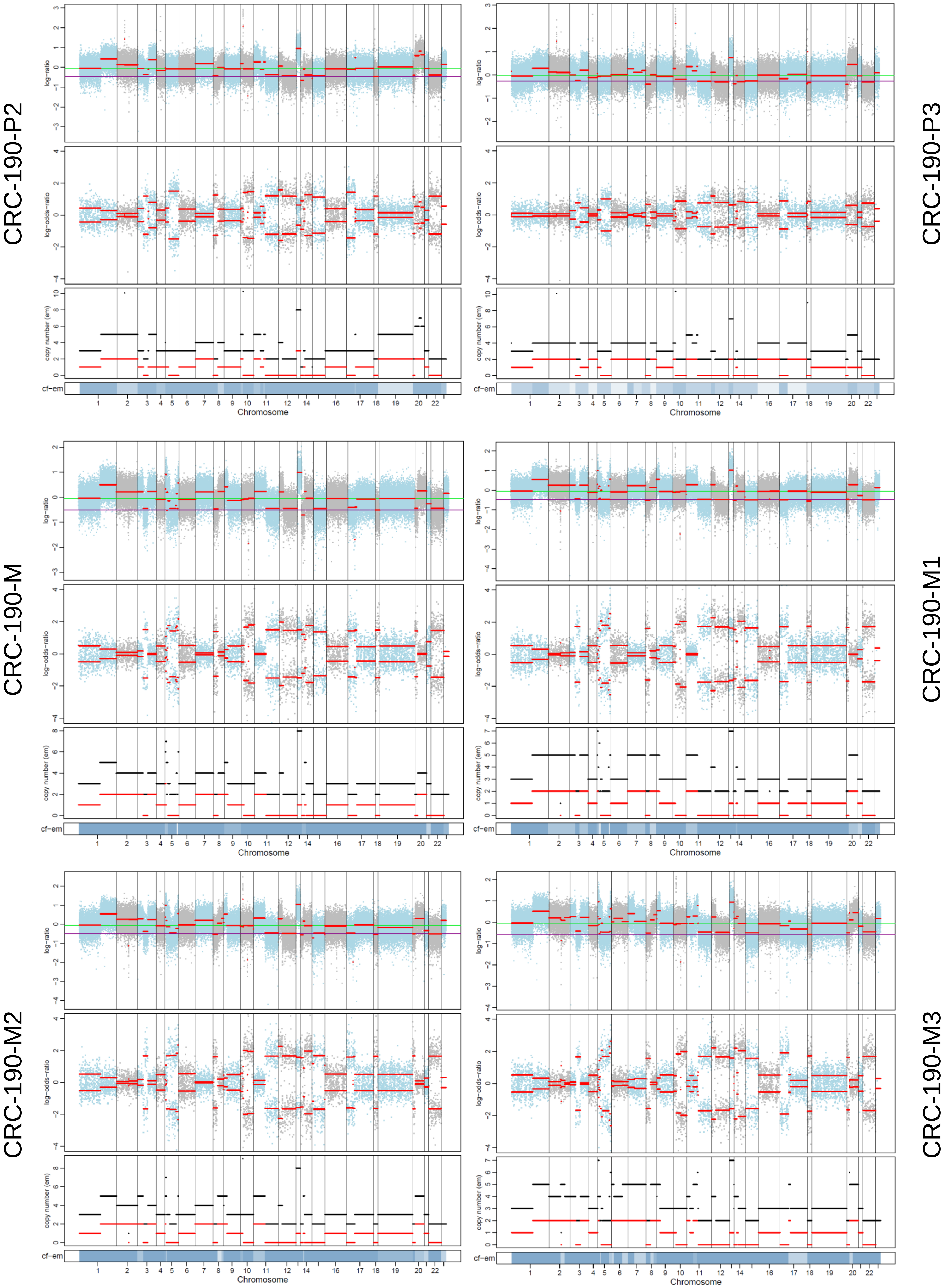
CRC-190-P2
CRC-190-P3
CRC-190-M
CRC-190-M1
CRC-190-M2
CRC-190-M3
log-ratio
log-odds-ratio
copy number (em)
cf-em
Chromosome
1 2 3 4 5 6 7 8 9 10 11 12 14 16 17 18 19 20 22

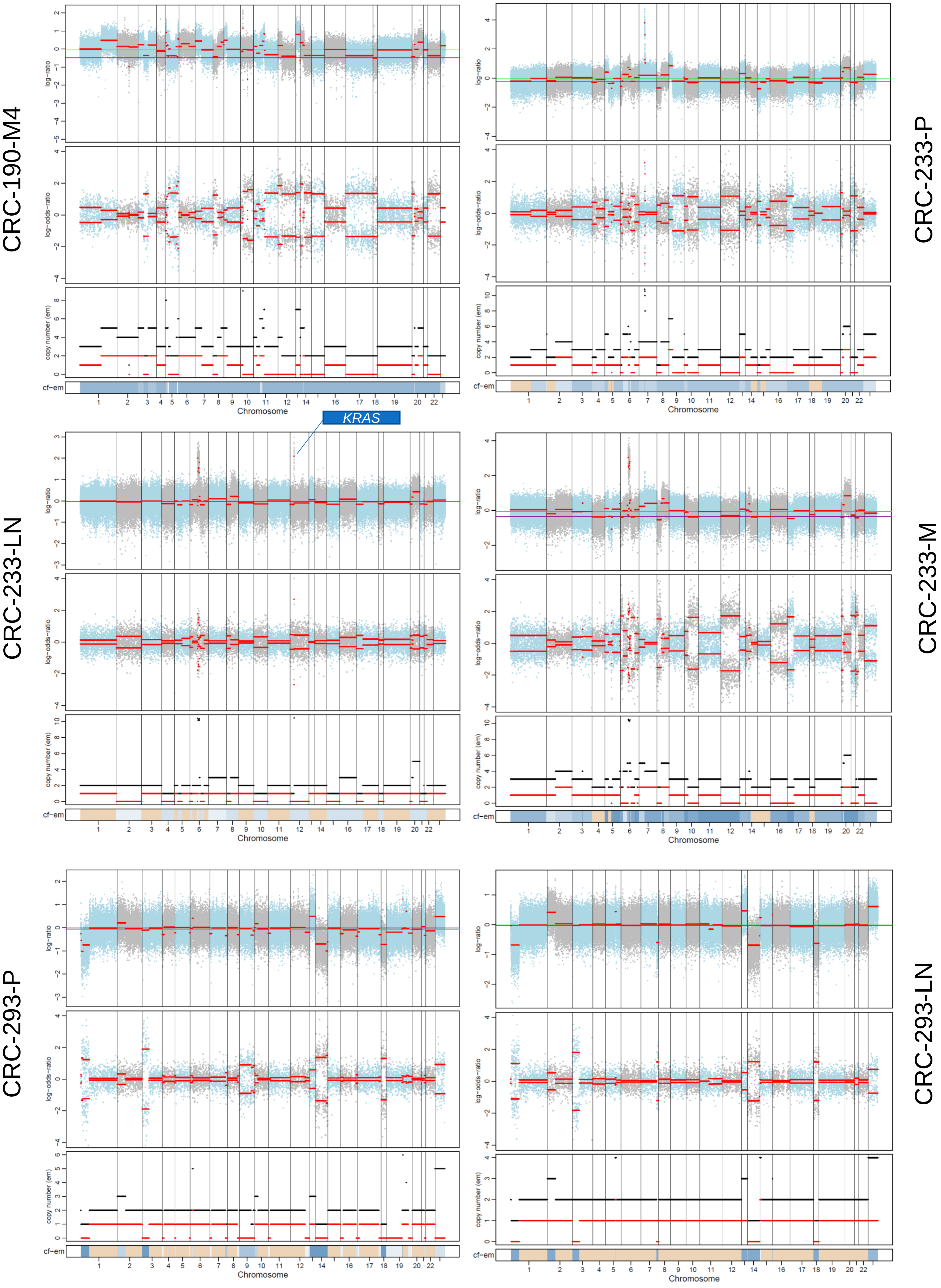
CRC-190-M4
CRC-233-P
CRC-233-LN
CRC-233-M
CRC-293-P
CRC-293-LN
KRAS
log-ratio
log-odds-ratio
copy number (em)
cf-em
Chromosome

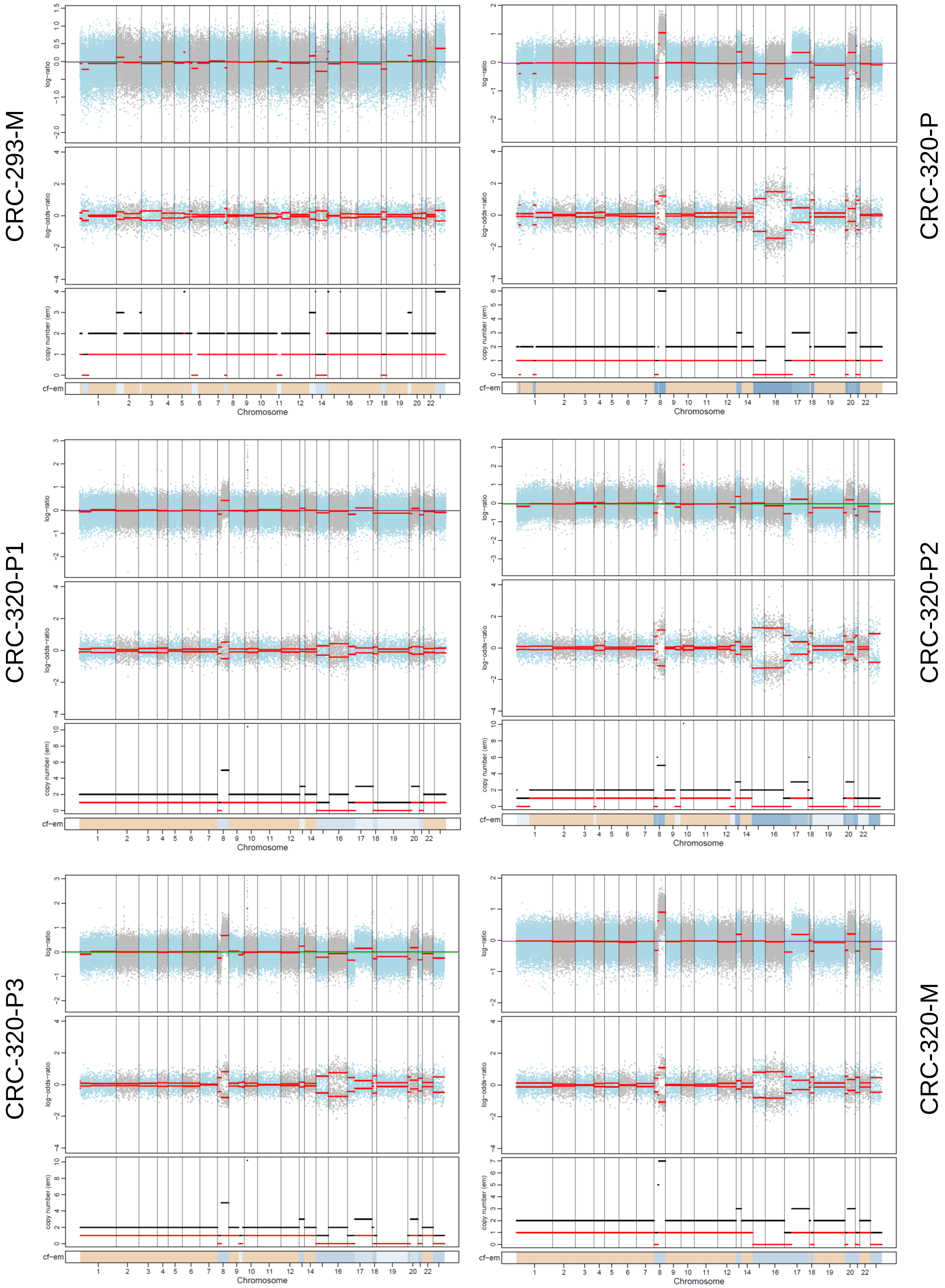
CRC-293-M
CRC-320-P
CRC-320-P1
CRC-320-P2
CRC-320-P3
CRC-320-M
log-ratio
log-odds-ratio
copy number (em)
cf-em
Chromosome

CRC-320-M1

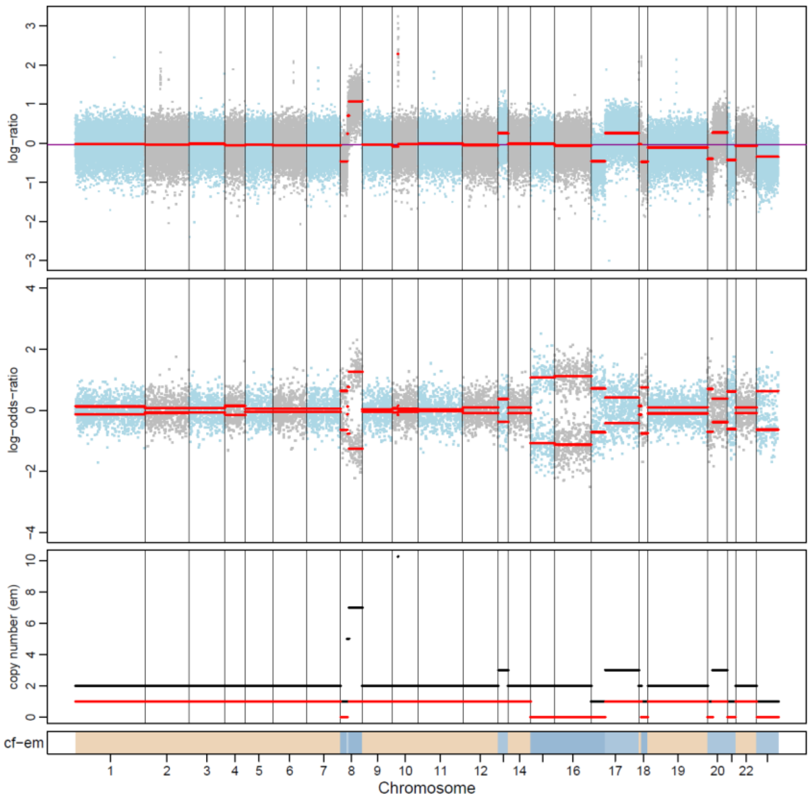

CRC-320-M2

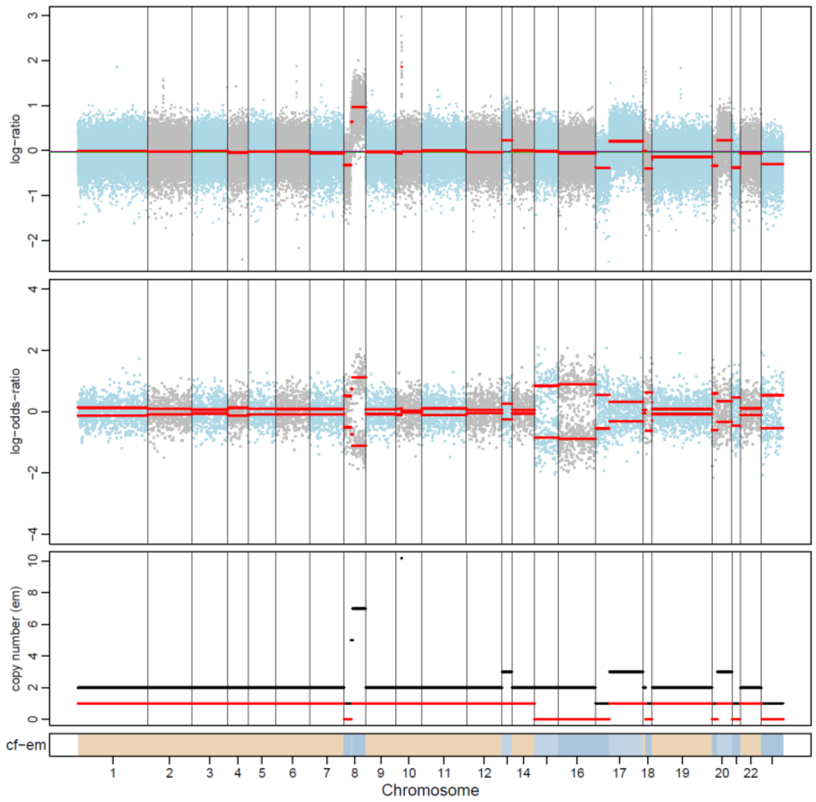

CRC-320-M3

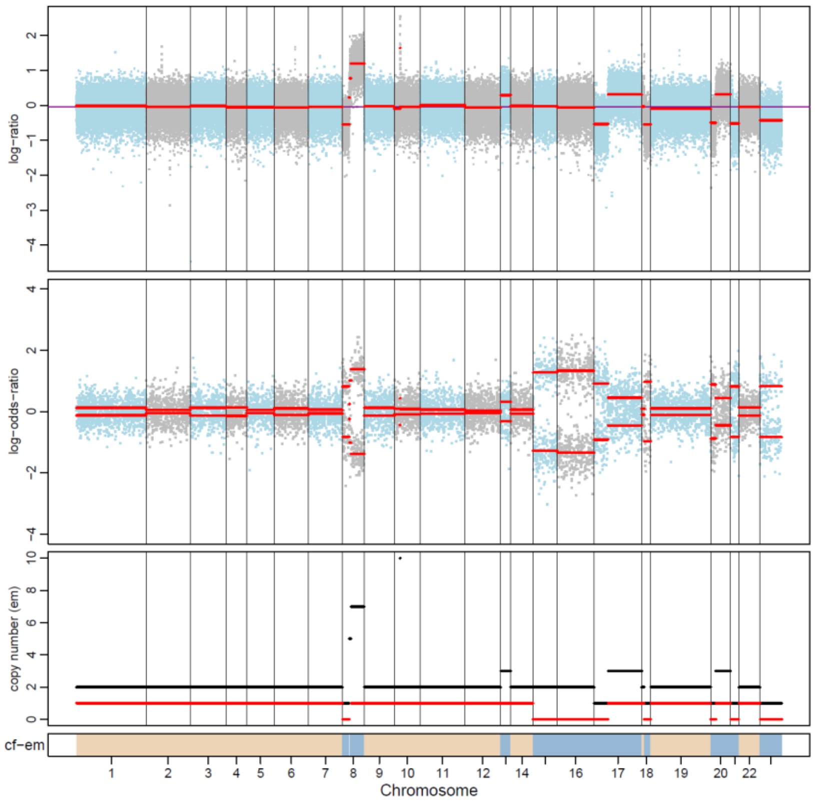

CRC-320-M4

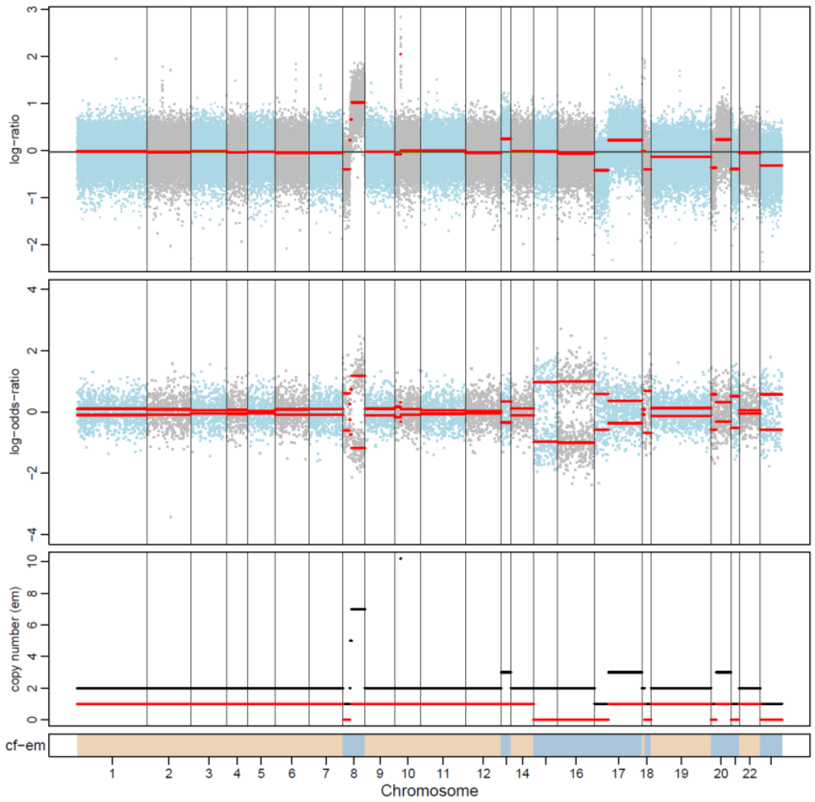

CRC-363-P

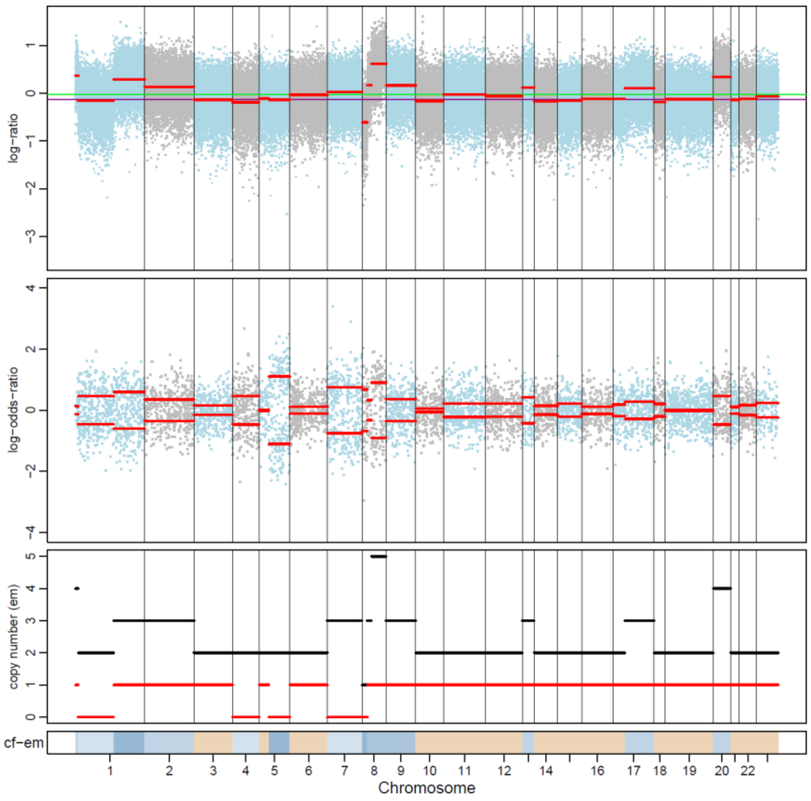

CRC-363-M

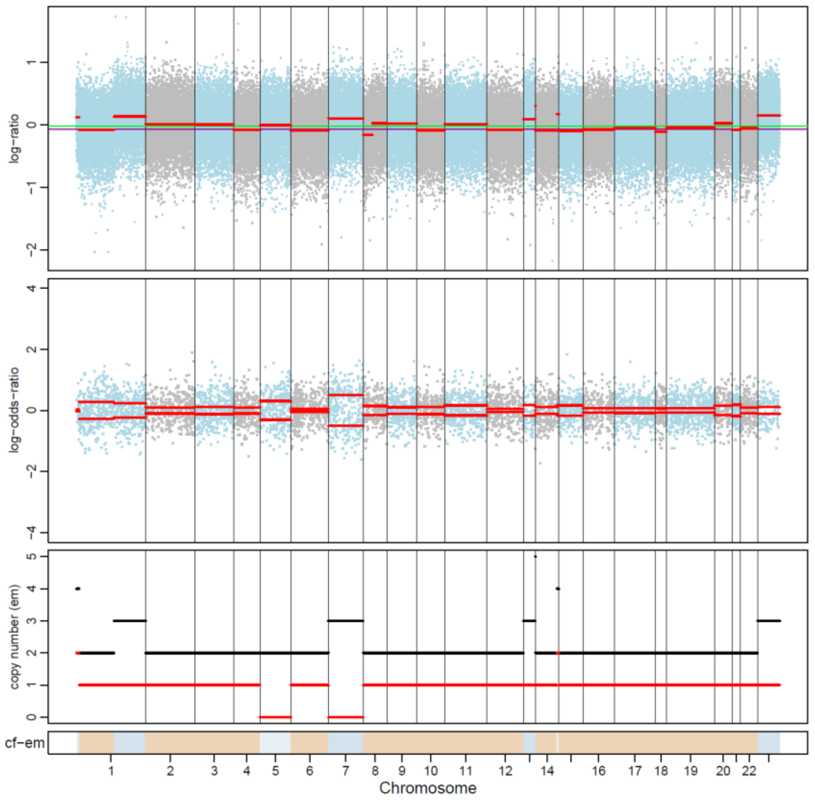

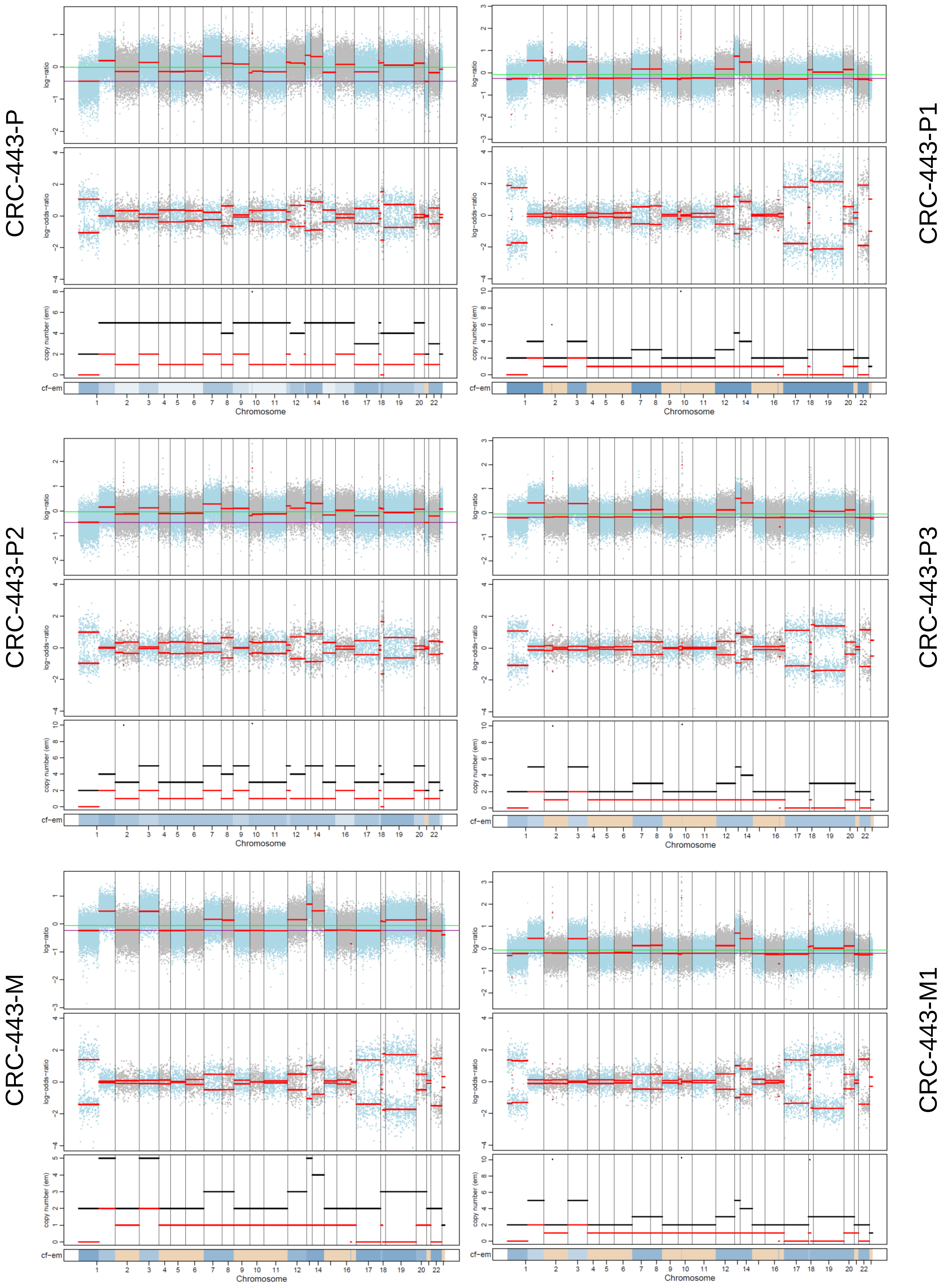
CRC-443-P
CRC-443-P1
CRC-443-P2
CRC-443-P3
CRC-443-M
CRC-443-M1
log−ratio
log−odds−ratio
copy number (em)
cf−em
1 2 3 4 5 6 7 8 9 10 11 12 14 16 17 18 19 20 22
Chromosome

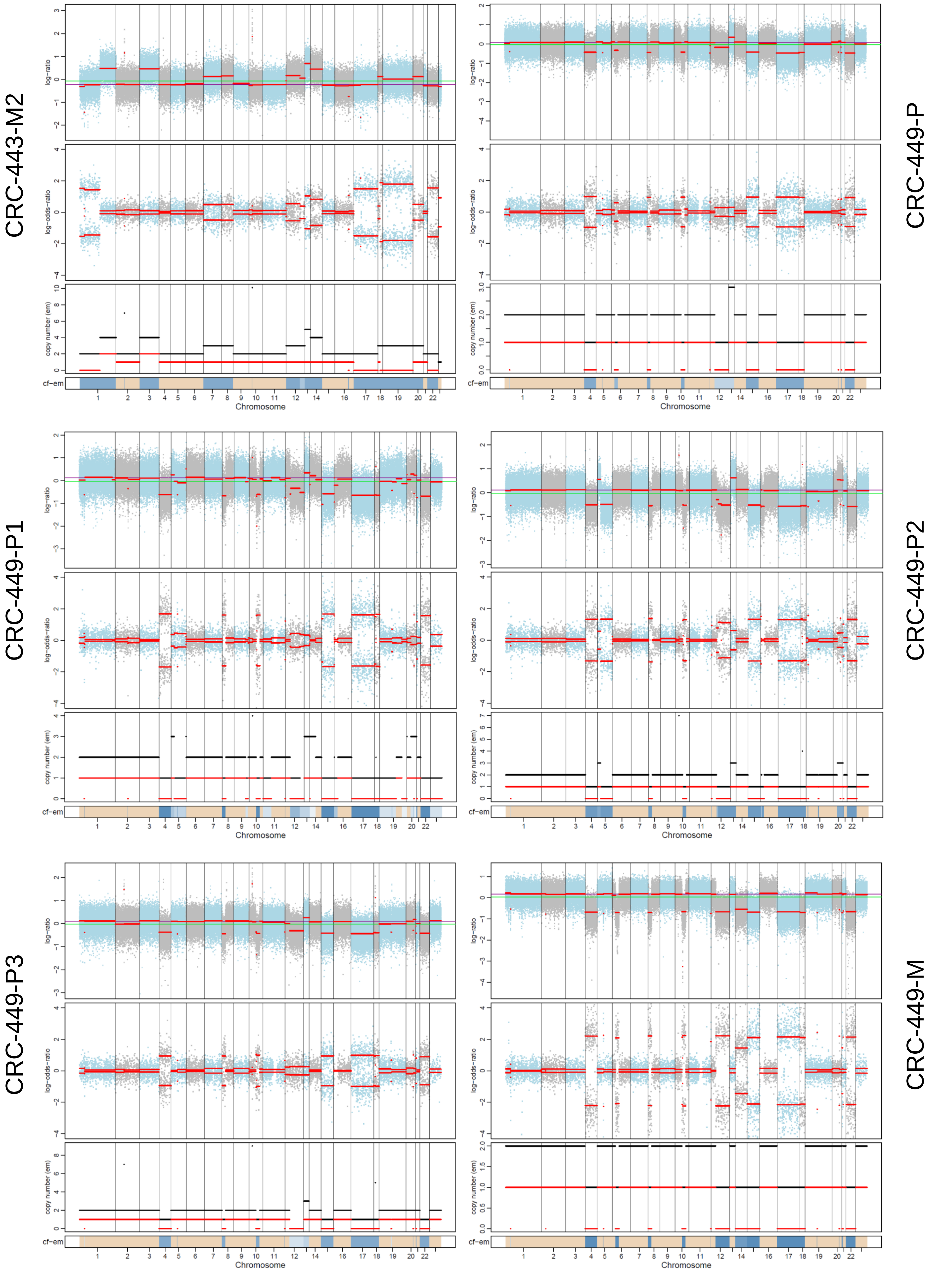
CRC-443-M2
CRC-449-P
CRC-449-P1
CRC-449-P2
CRC-449-P3
CRC-449-M
log-ratio
log-odds-ratio
copy number (em)
cf-em
Chromosome

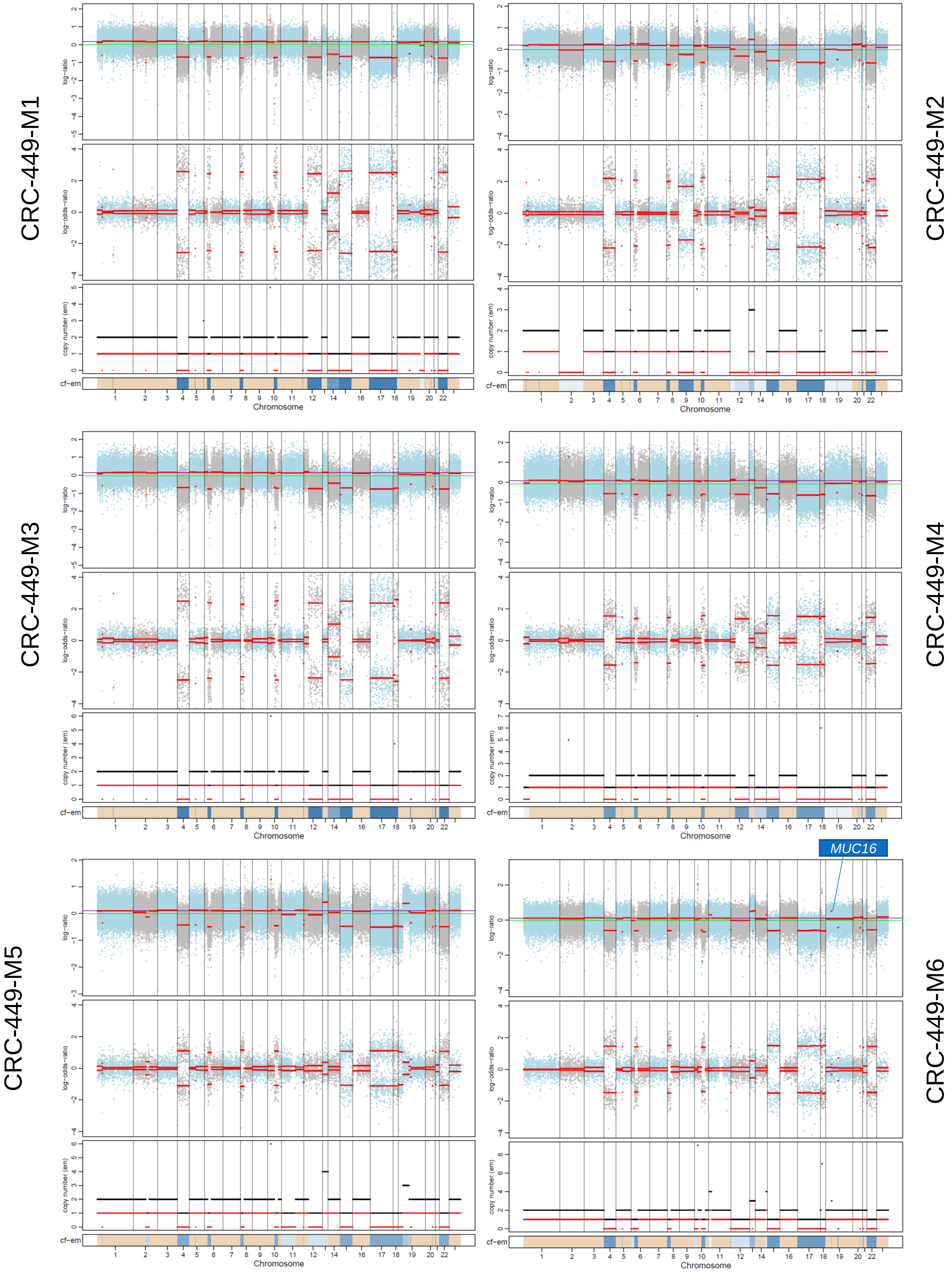
CRC-449-M1
CRC-449-M2
CRC-449-M3
CRC-449-M4
CRC-449-M5
CRC-449-M6
MUC16
log−ratio
log−odds−ratio
copy number (em)
cf−em
Chromosome

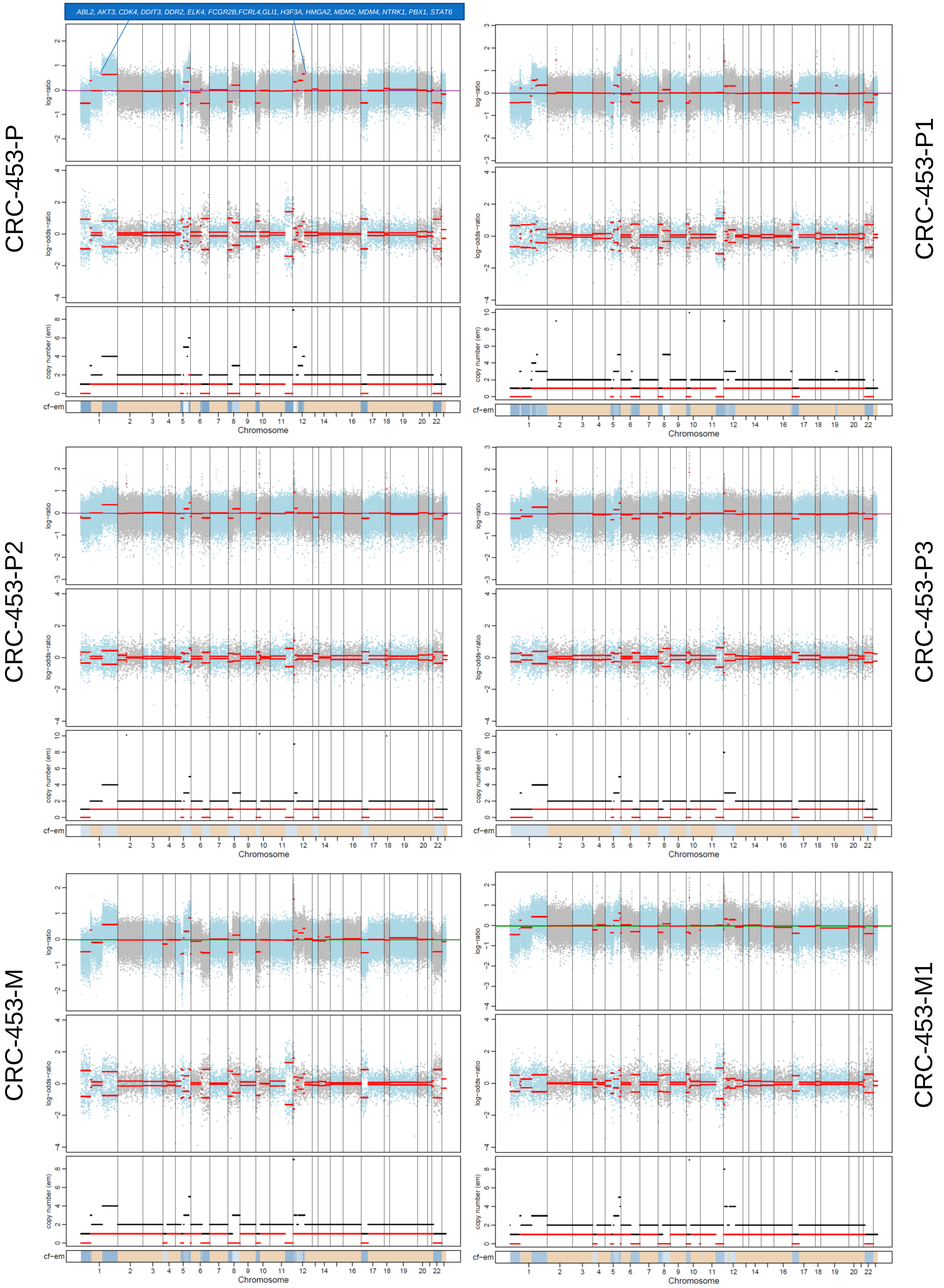
ABL2, AKT3, CDK4, DDIT3, DDR2, ELK4, FCGR2B,FCRL4,GLI1, H3F3A, HMGA2, MDM2, MDM4, NTRK1, PBX1, STAT6
CRC-453-P
CRC-453-P1
CRC-453-P2
CRC-453-P3
CRC-453-M
CRC-453-M1
log−ratio
log−odds−ratio
copy number (em)
cf−em
Chromosome

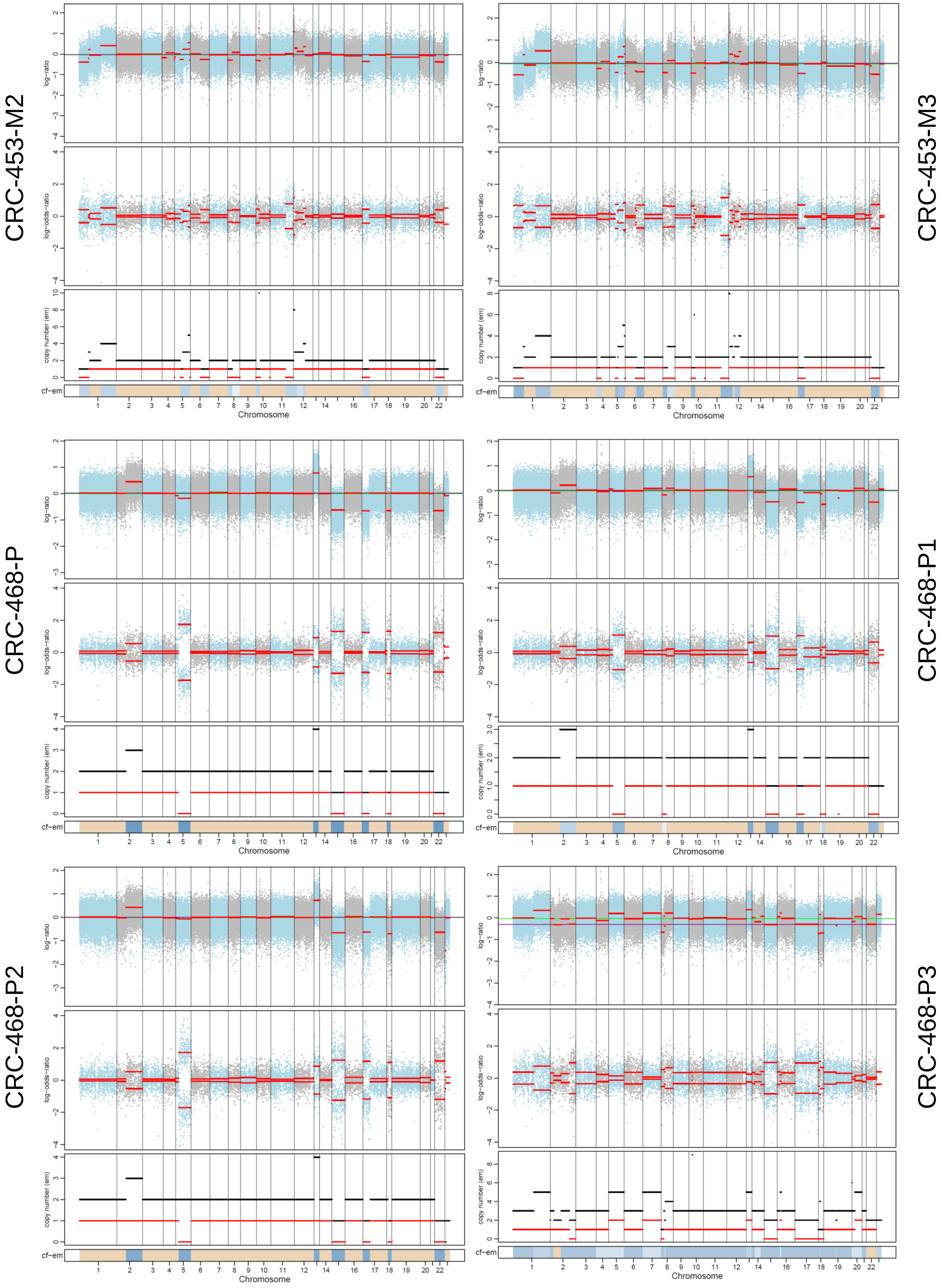
CRC-453-M2
CRC-453-M3
CRC-468-P
CRC-468-P1
CRC-468-P2
CRC-468-P3
log−ratio
log−odds−ratio
copy number (em)
cf−em
Chromosome

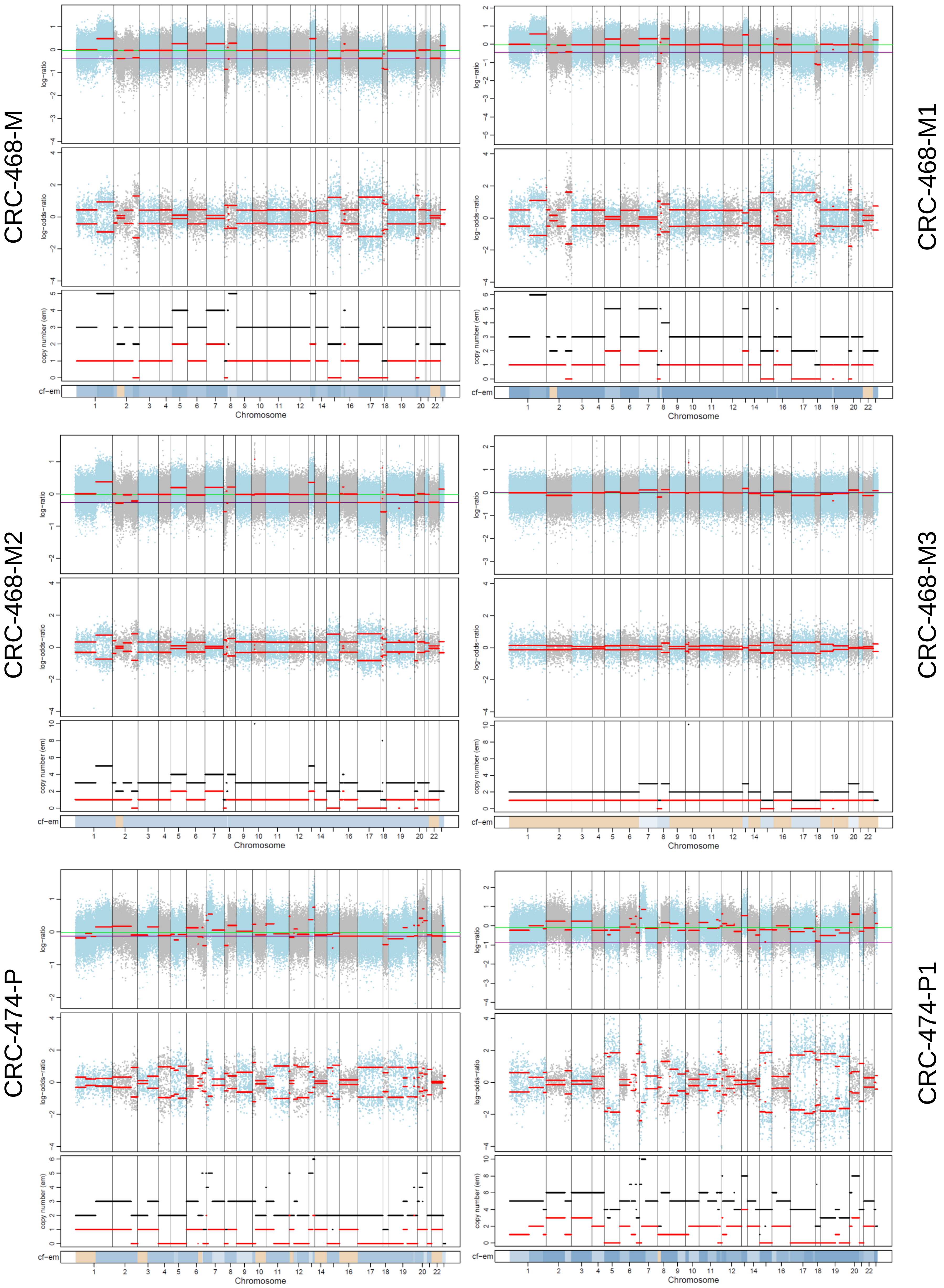
CRC-468-M
CRC-468-M1
CRC-468-M2
CRC-468-M3
CRC-474-P
CRC-474-P1
log-ratio
log-odds-ratio
copy number (em)
cf-em
Chromosome

CRC-474-P2

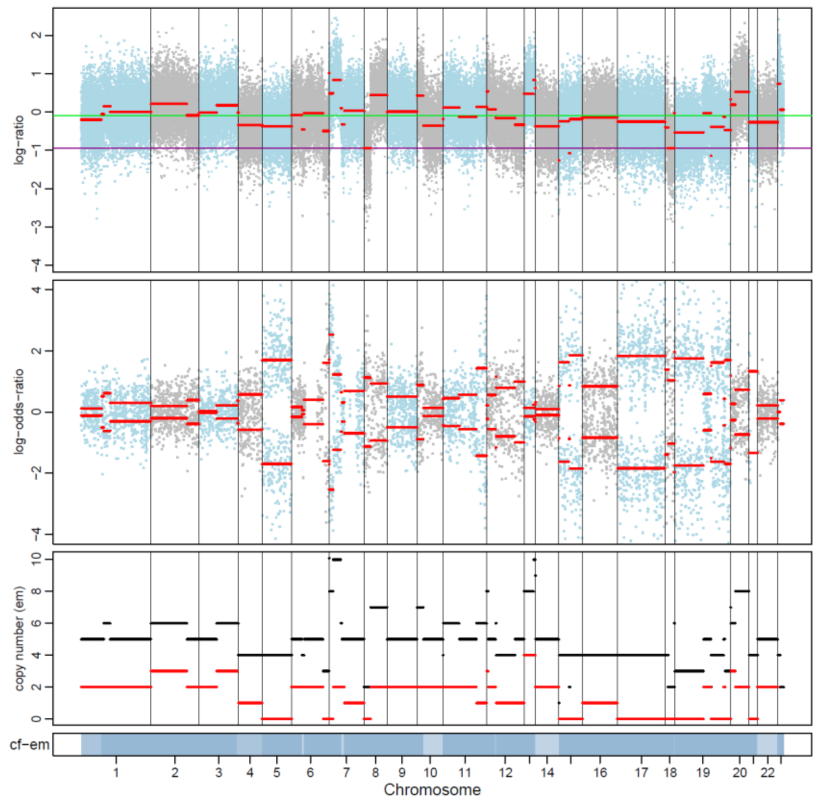

CRC-474-P3

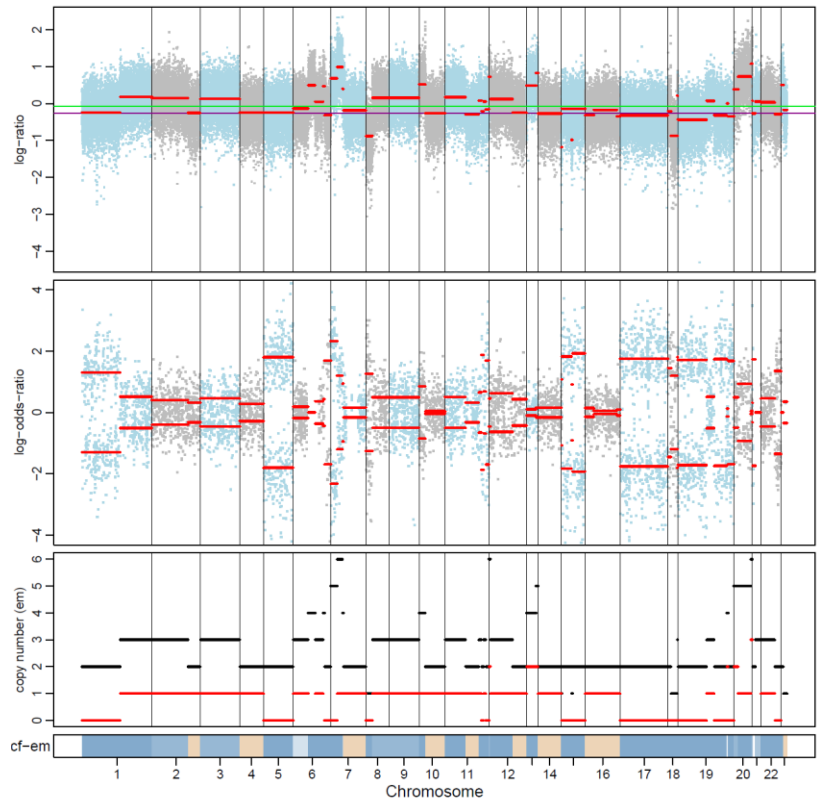

CRC-474-M

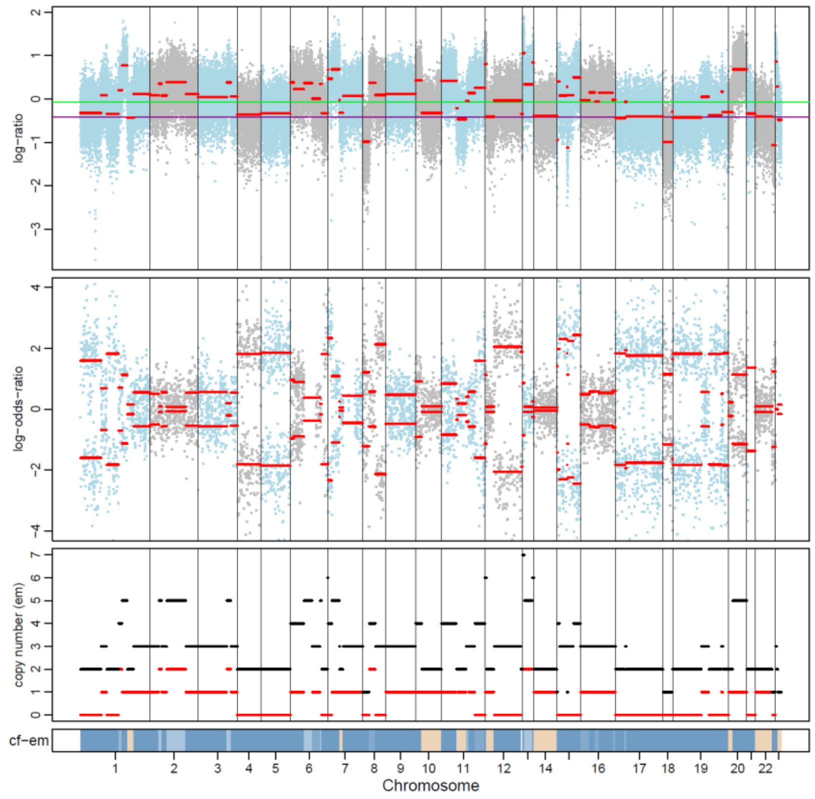

CRC-474-M1

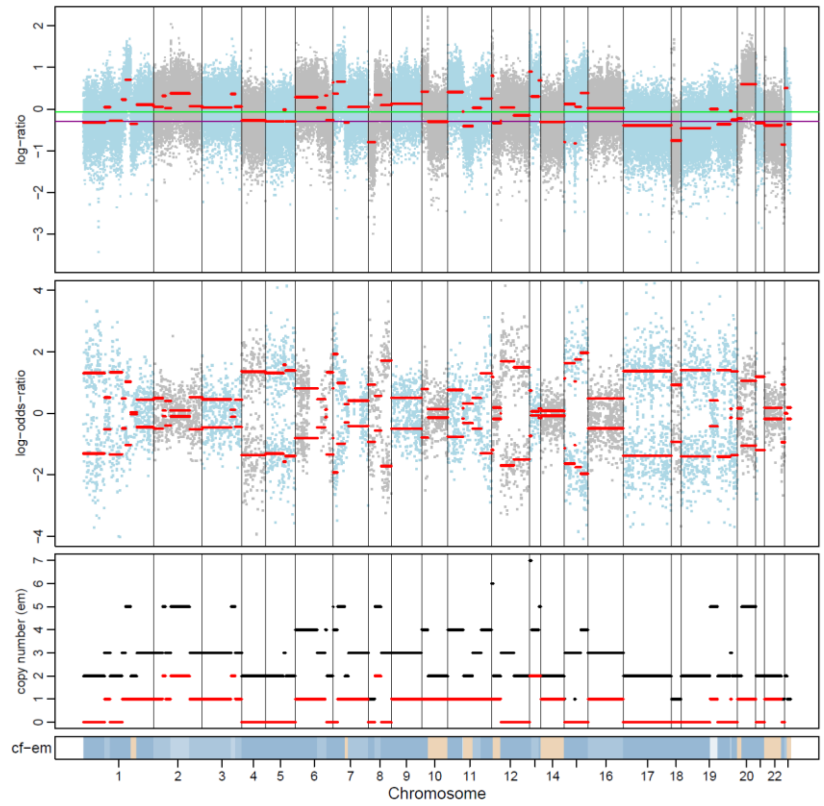

CRC-474-M2

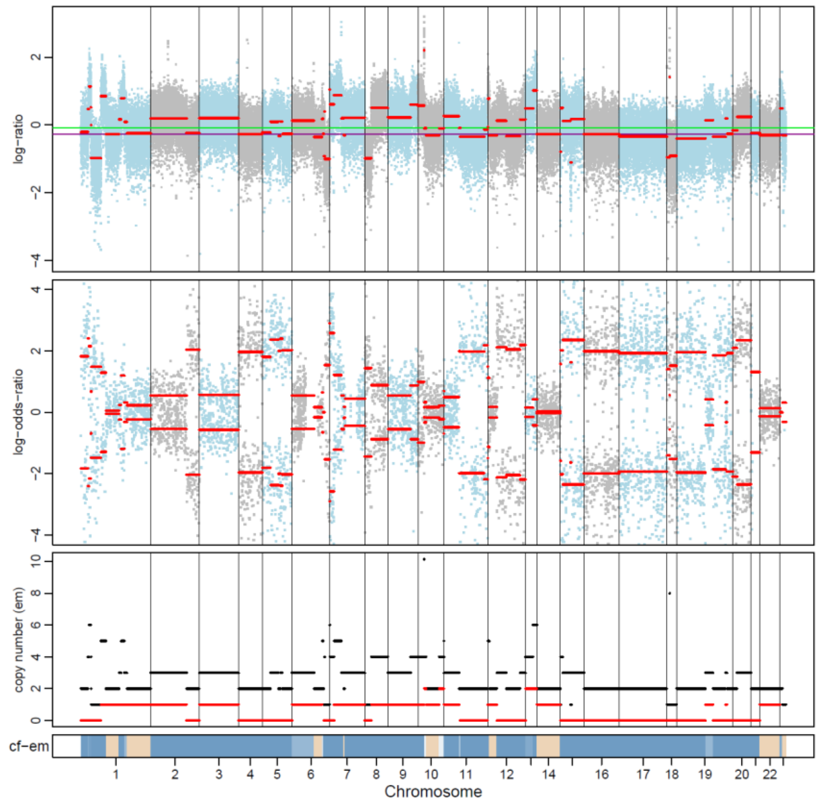

CRC-474-M3

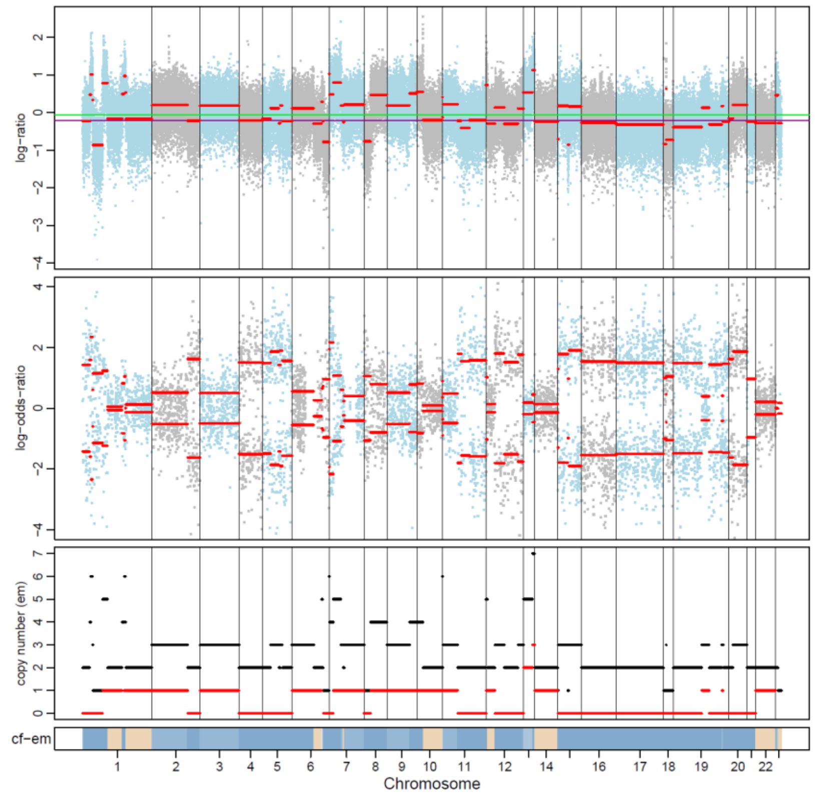

CRC-474-M4

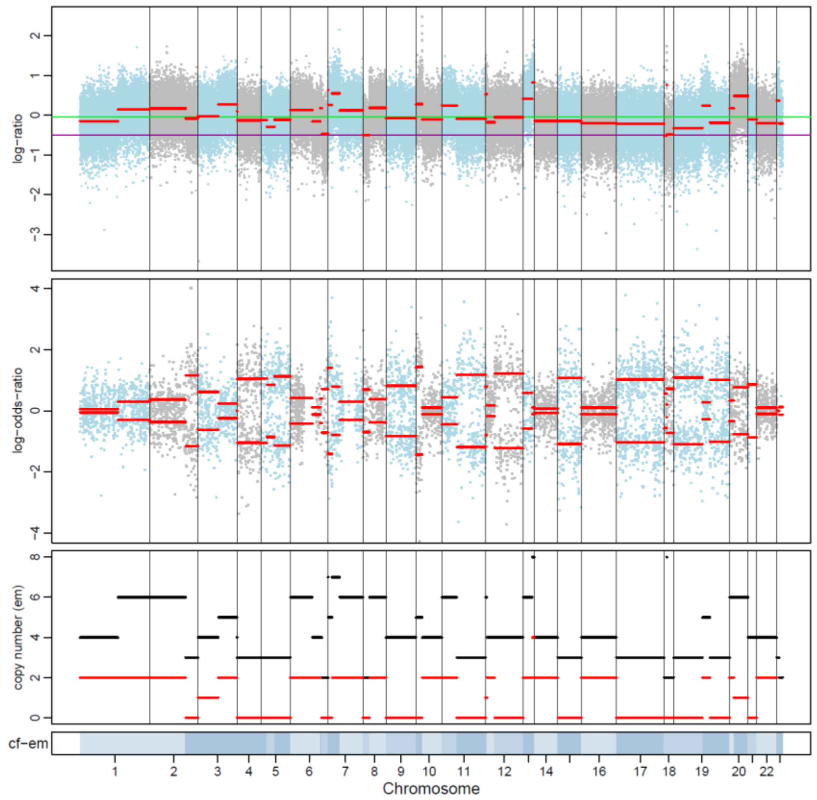

CRC-477-P

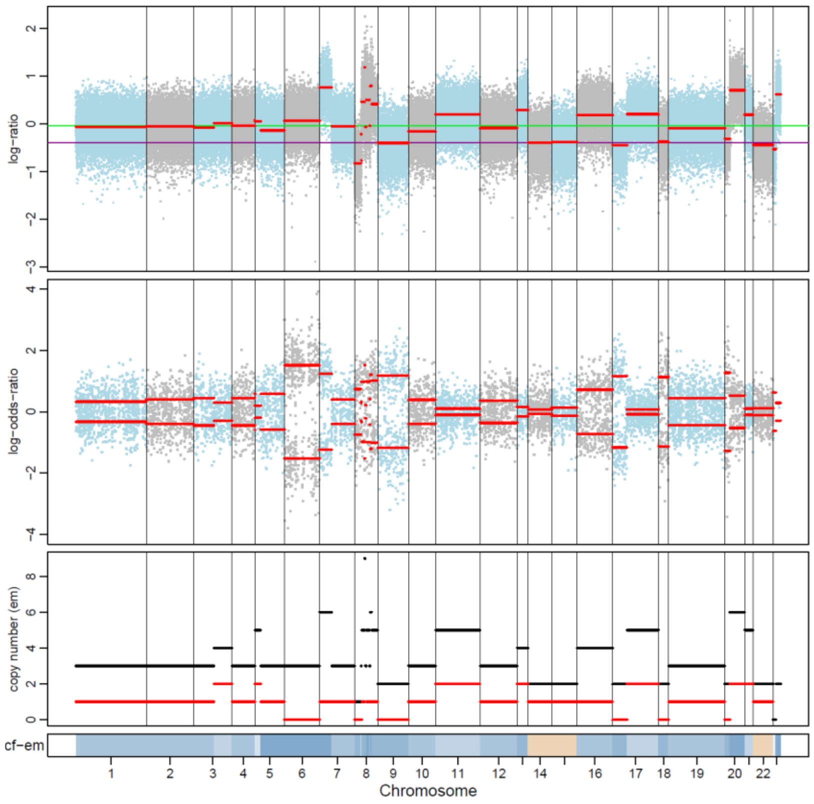

CRC-477-P1

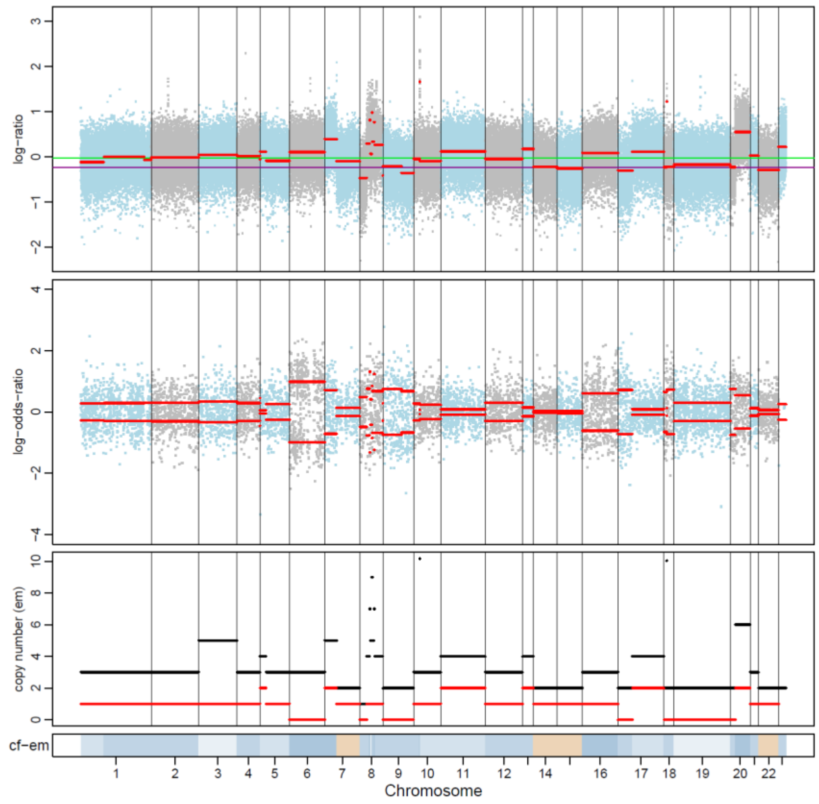

CRC-477-P2

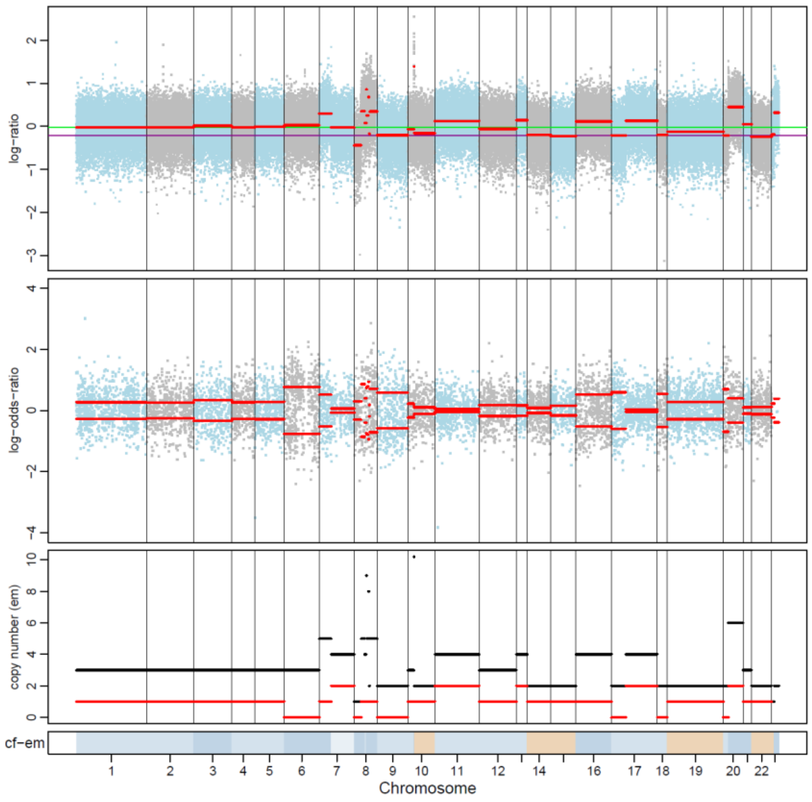

CRC-477-P3

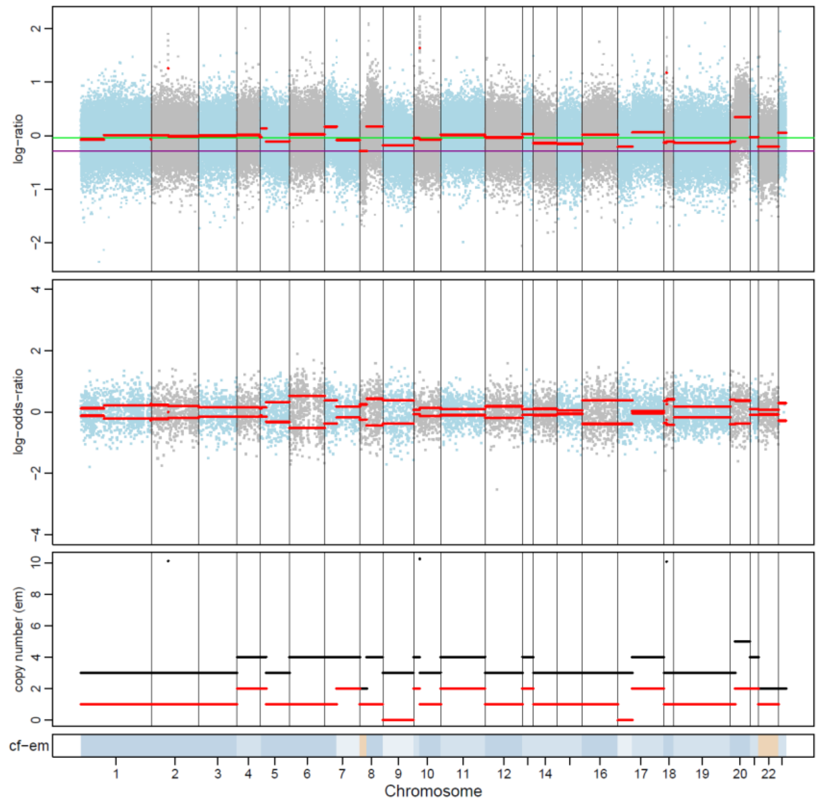

CRC-477-M

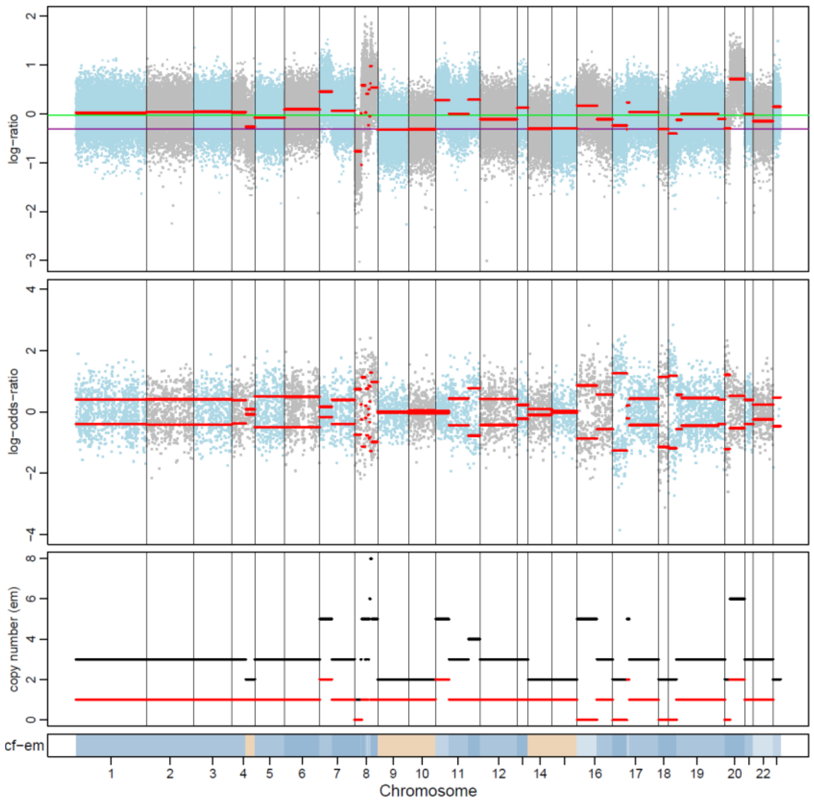

CRC-477-M1

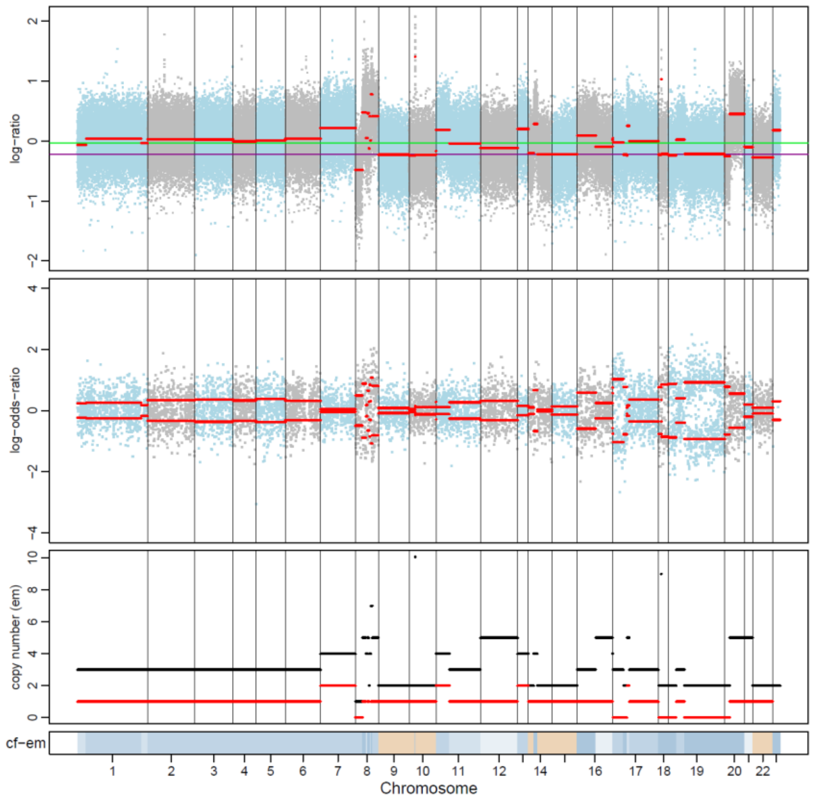

CRC-477-M2

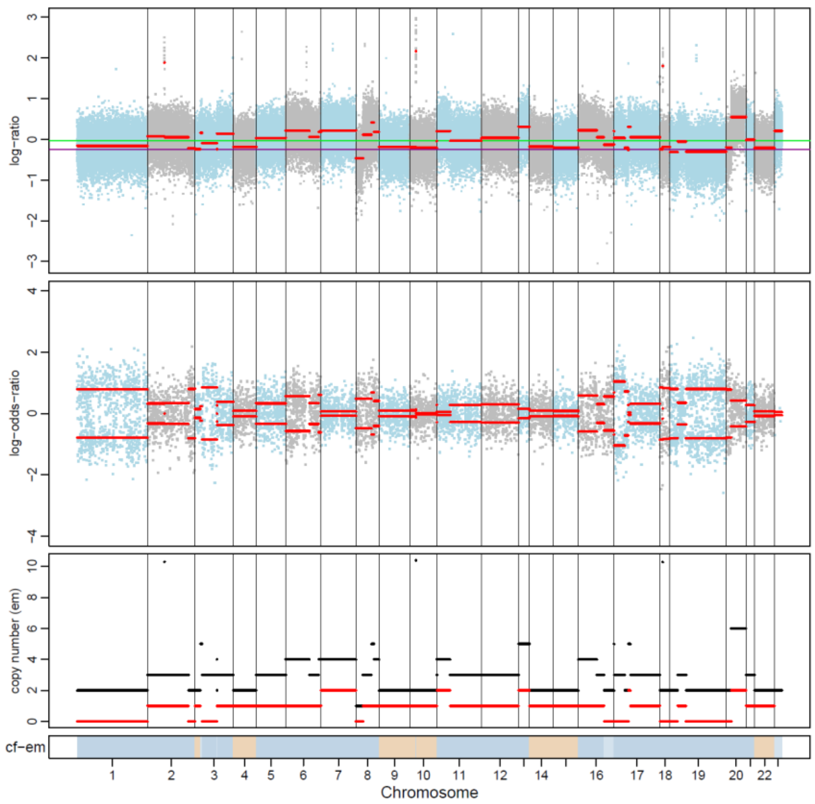

CRC-477-M3

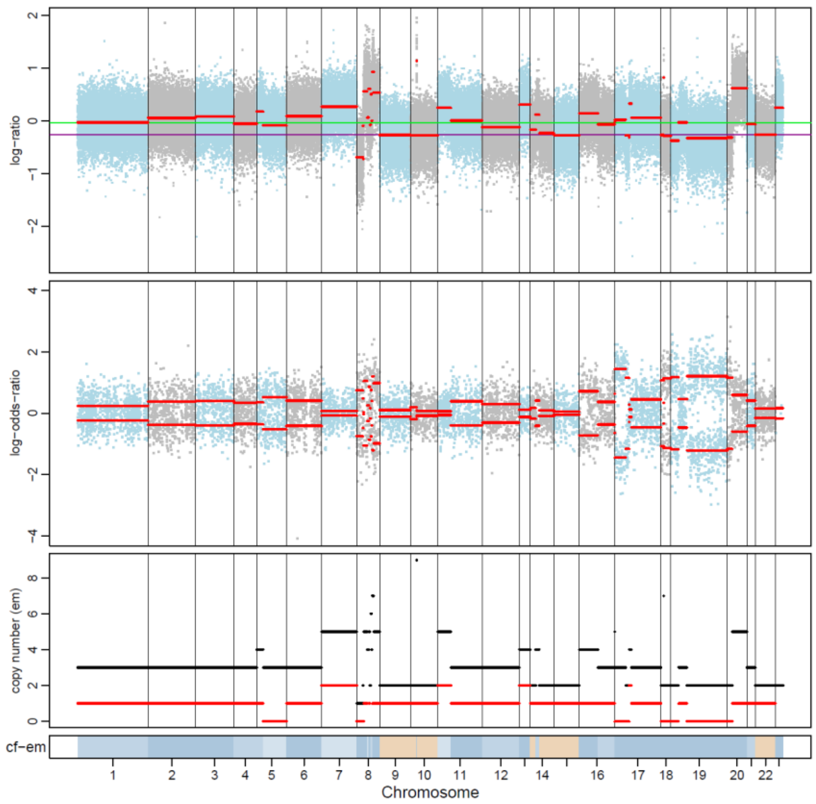

CRC-477-M4

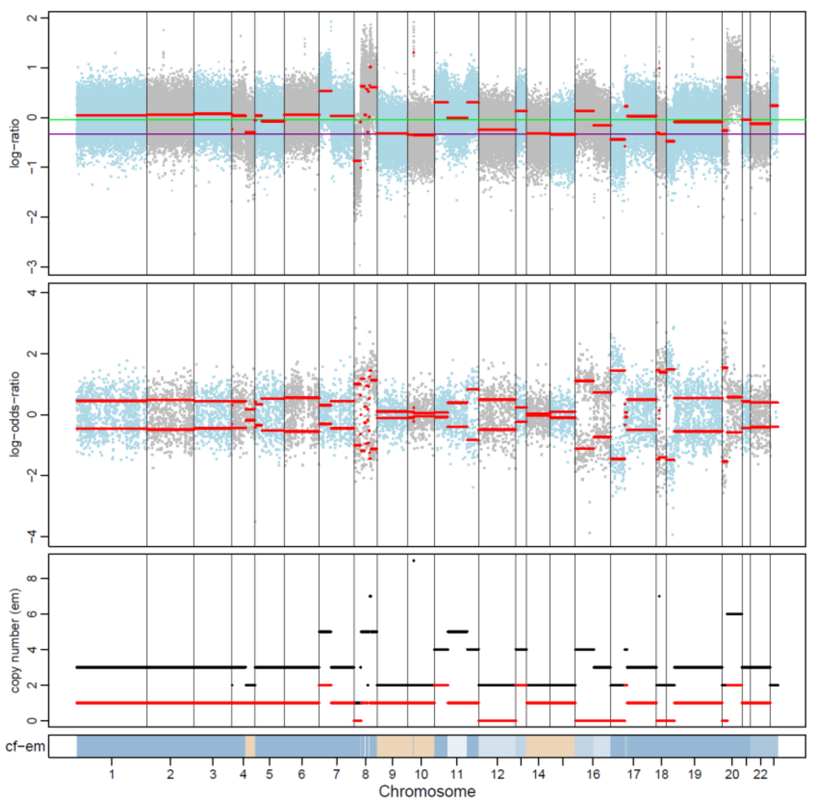

CRC-477-M5

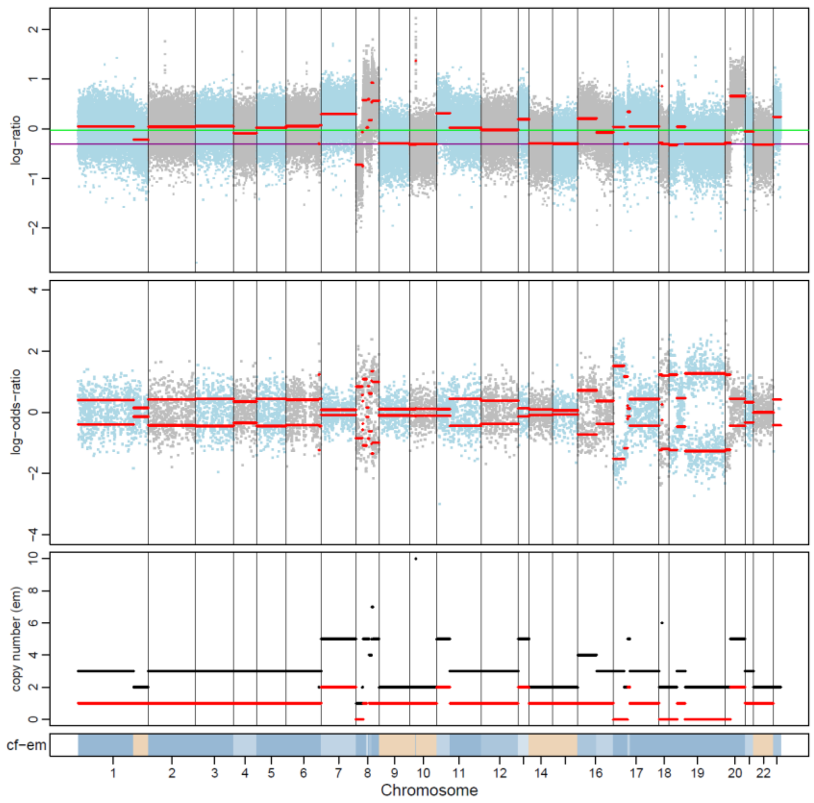

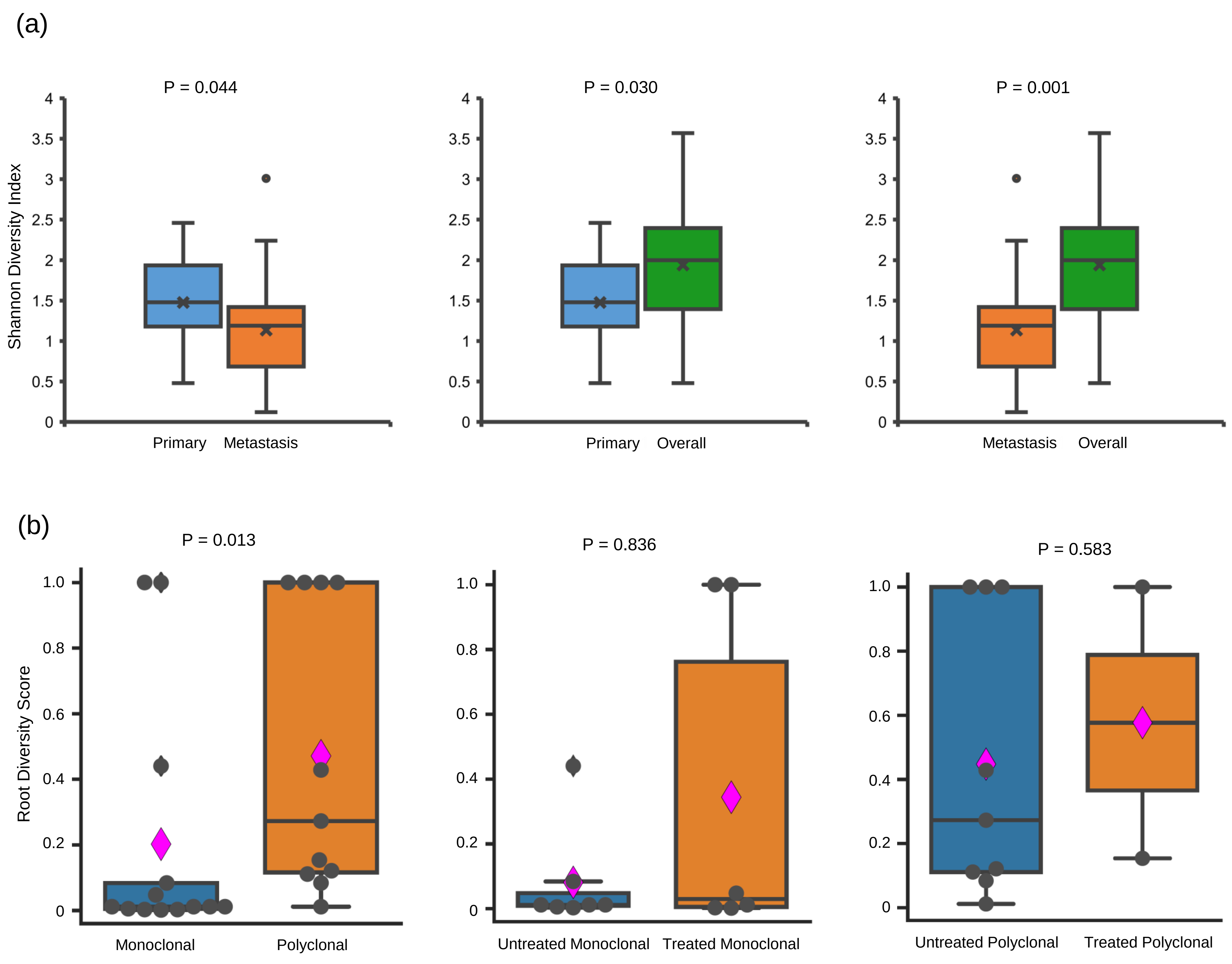

Figure S2: Box plot representation of tumour heterogeneity, via (a) Shannon Diversity Index (SDI) (b) Root Diversity Scores (RDS) of 21 samples with multiple sampled regions

(a)

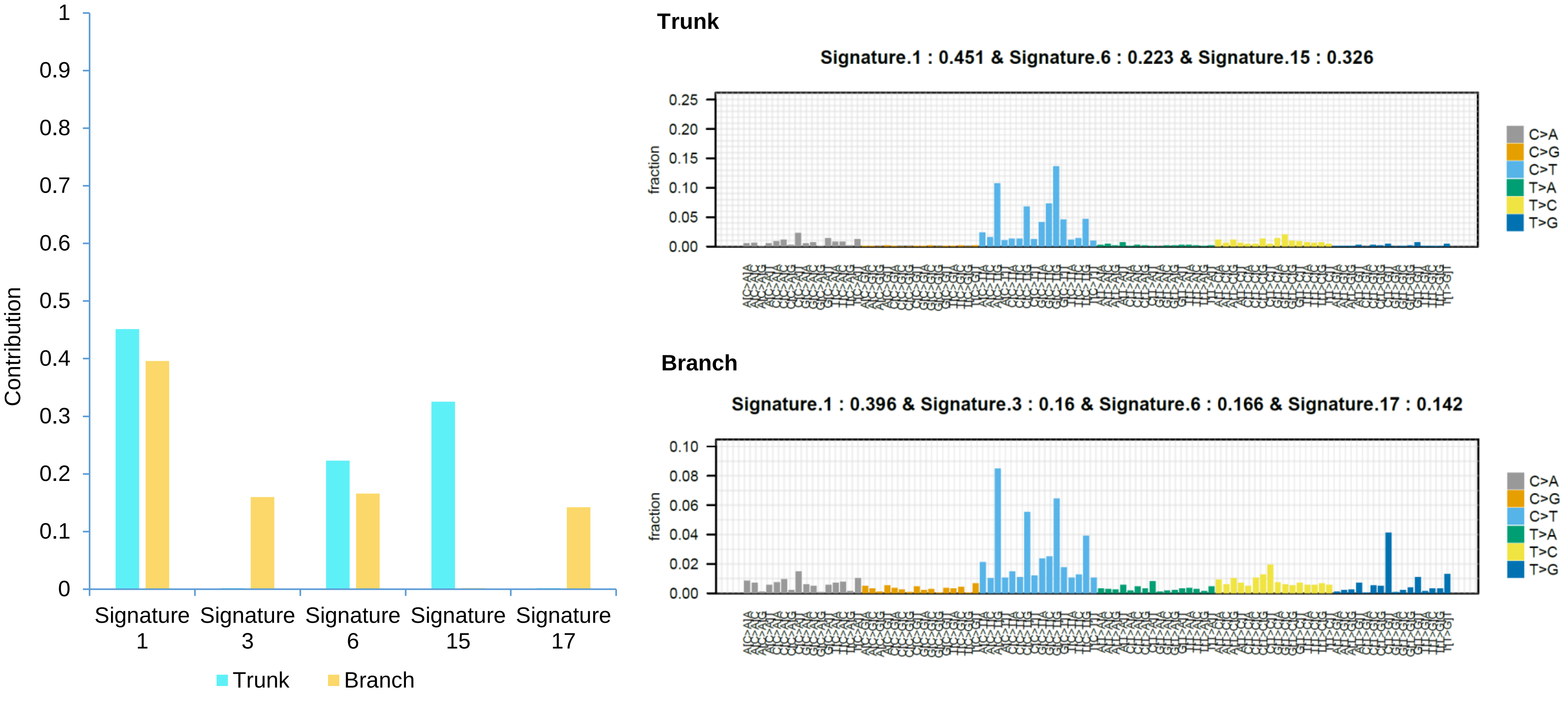

(b)

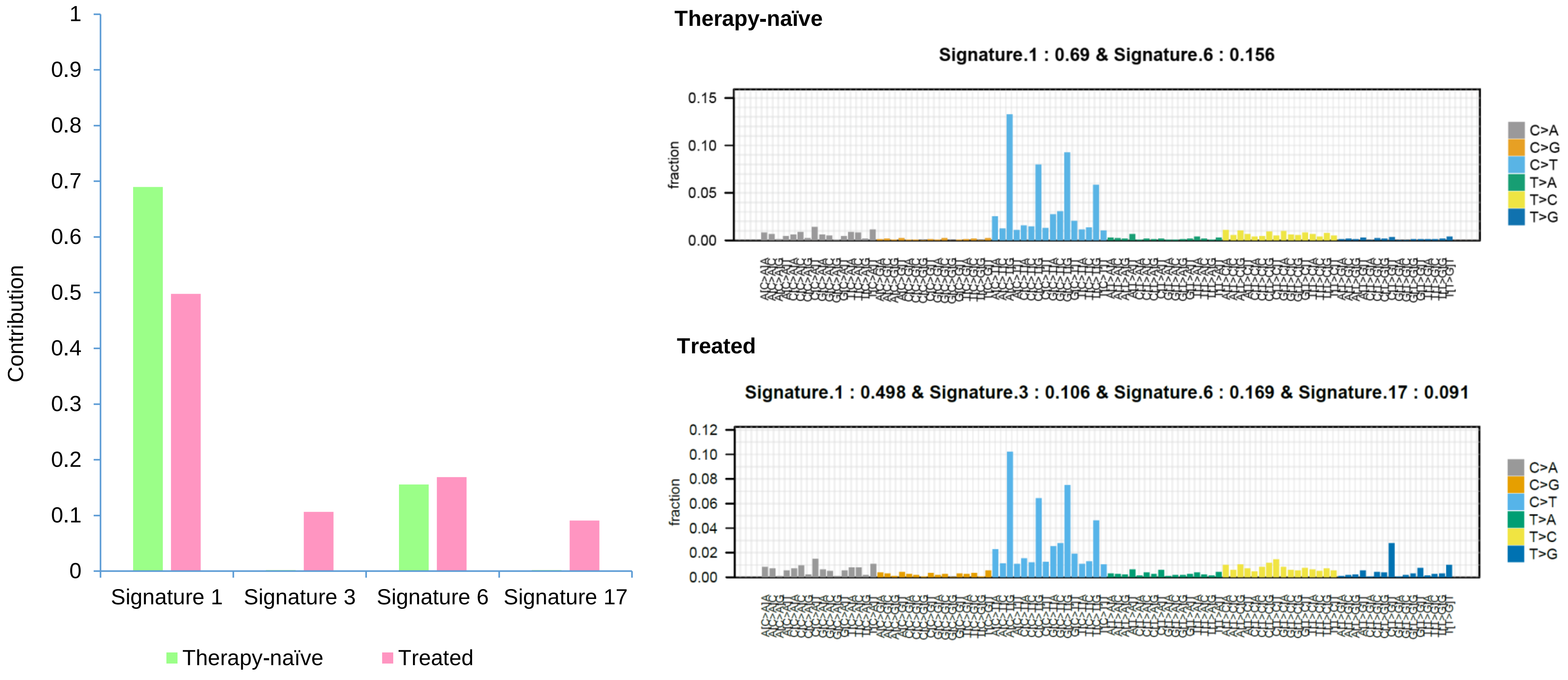

Figure S3: Contribution of all mutational signatures, from the 30 published COSMIC mutational signatures, across the metastatic CRC cohort for (a) Trunk and branch single nucleotide base mutations; and single nucleotide base mutations in (b) Therapy-naïve and treated cases.
The raw plots have also been included. Signature 1 relates to age at diagnosis; Signature 3 is associated with defective DNA homologous repair; Signatures 6 and 15 are related to defective DNA mismatch repair; and Signature 17 is partially related to 5-fluorouracil and capecitabine chemotherapy

Figure S4: Phylogenetic trees.

Phylogenetic trees for 30 colorectal cancer tumors with metastasis. For each sample only the optimal tree derived using LICHeE (Lineage Inference for Cancer Heterogeneity and Evolution) is presented. Driver genes are highlighted in red; non-driver cancer gene events are denoted in blue and non-cancer events in black. Parallel events converging at a particular gene have been underlined. The middle top pane shows the complete phylogenetic tree as constructed based on mutation clusters, with primary and metastatic phylogenetic trees, with metastatic site and %final molecular time at primary tumour, shown separately in a black box. Different colored nodes represent distinct clones, with the number of mutations contributing to the cluster displayed inside each node. Below are the phylogenetic trees shown for each region, where clusters not found in a given region have been represented as grey empty nodes. “P”, represents regions from primary tumors; “LN-M”, represents tumor tissue from lymph node metastases; “M”, represents metastatic tumor tissue. Beneath each regional phylogenetic tree, a grid of a 100 representative cells shows the mutational clusters present via coinciding cluster colors.

083-03

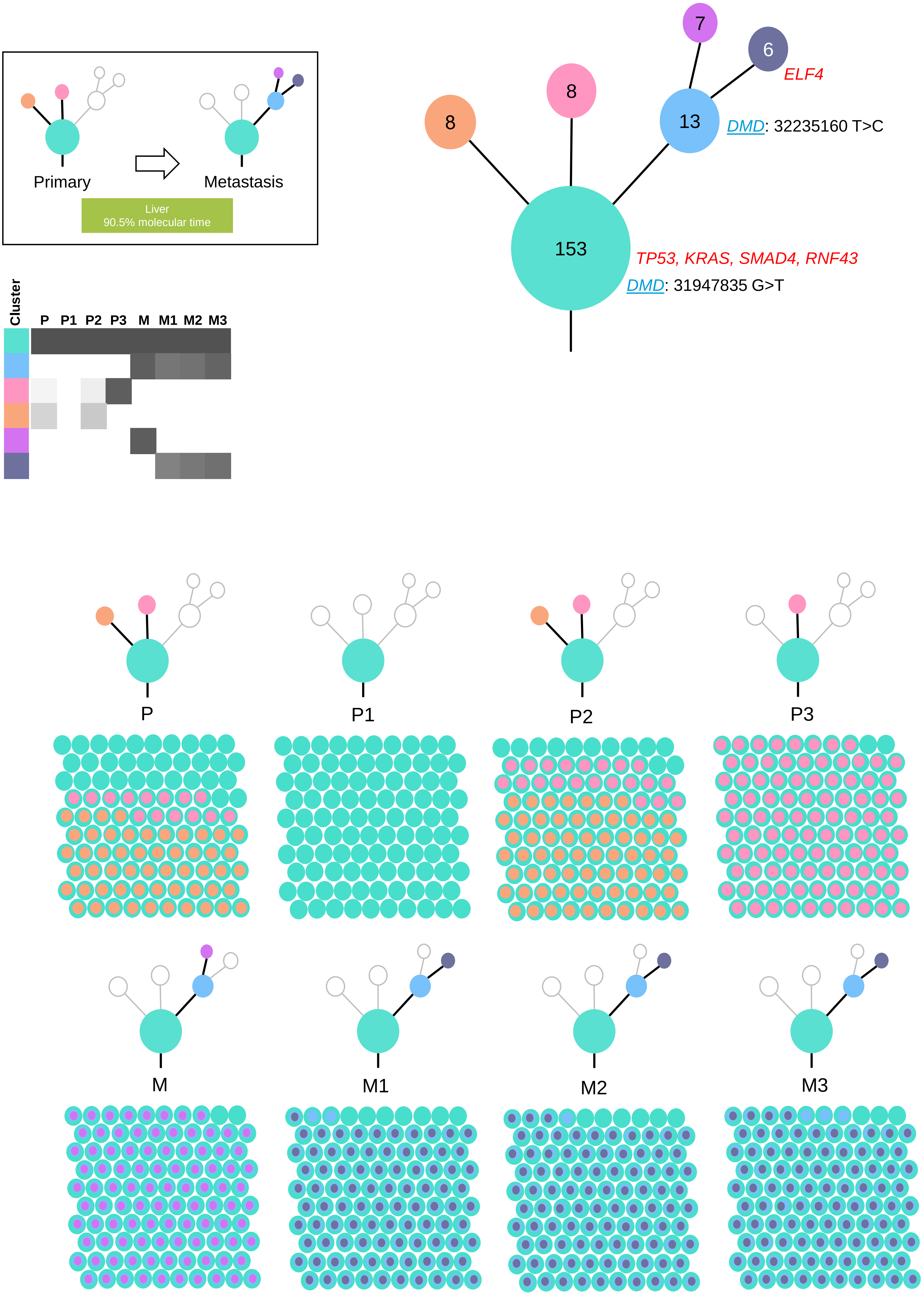
Primary
Metastasis
Liver
90.5% molecular time
Cluster
P P1 P2 P3 M M1 M2 M3
7
6
ELF4
8
8
13
DMD: 32235160 T>C
153
TP53, KRAS, SMAD4, RNF43
DMD: 31947835 G>T
P
P1
P2
P3
M
M1
M2
M3

083-32

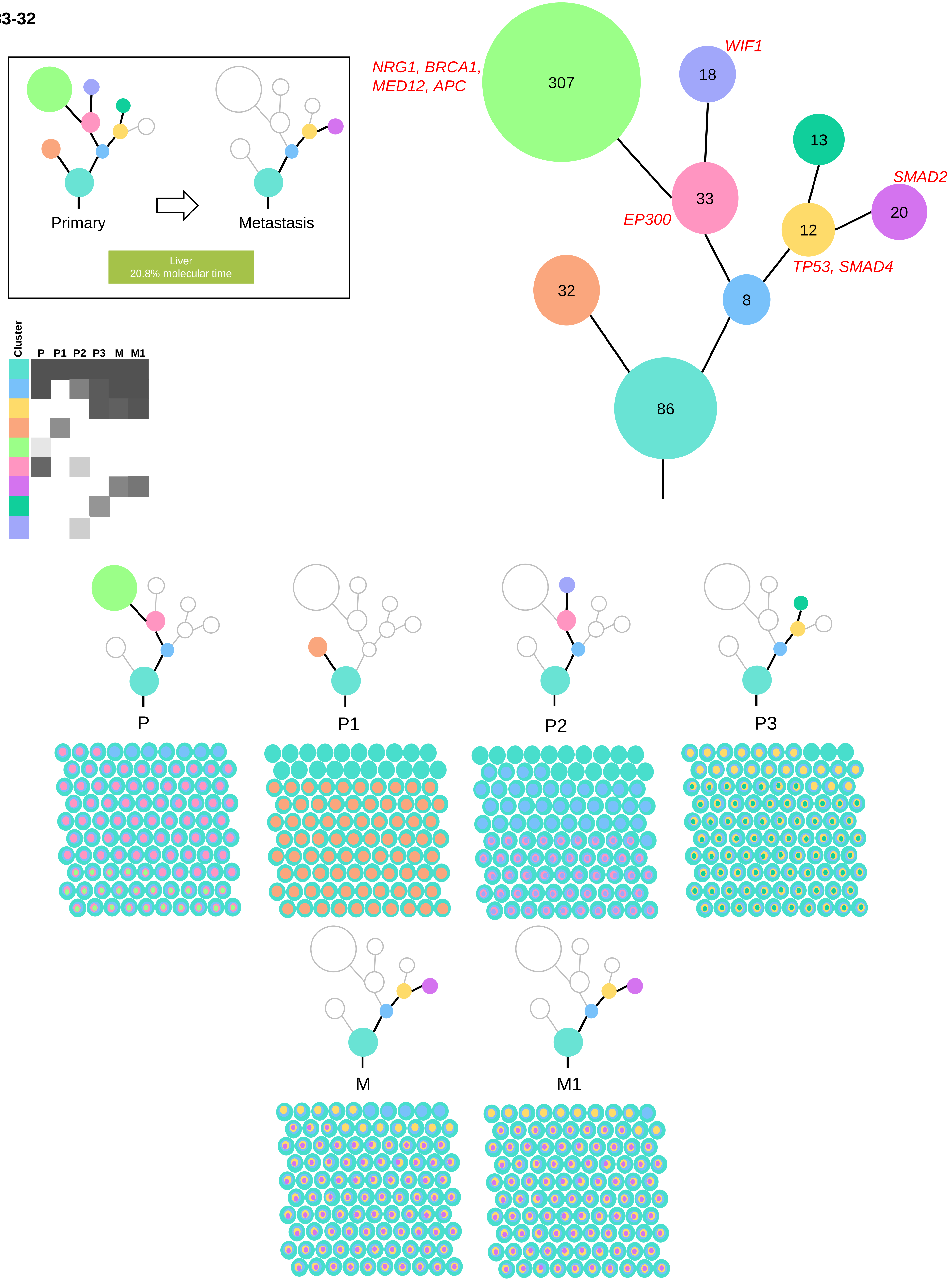
Primary
Metastasis
Liver
20.8% molecular time
NRG1, BRCA1,
MED12, APC
307
WIF1
18
13
SMAD2
33
20
EP300
12
TP53, SMAD4
32
8
86
Cluster
P
P1
P2
P3
M
M1

112-038

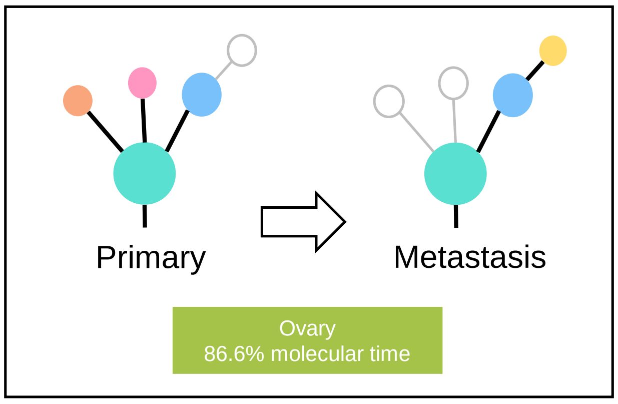

Primary
Metastasis
Ovary
86.6% molecular time

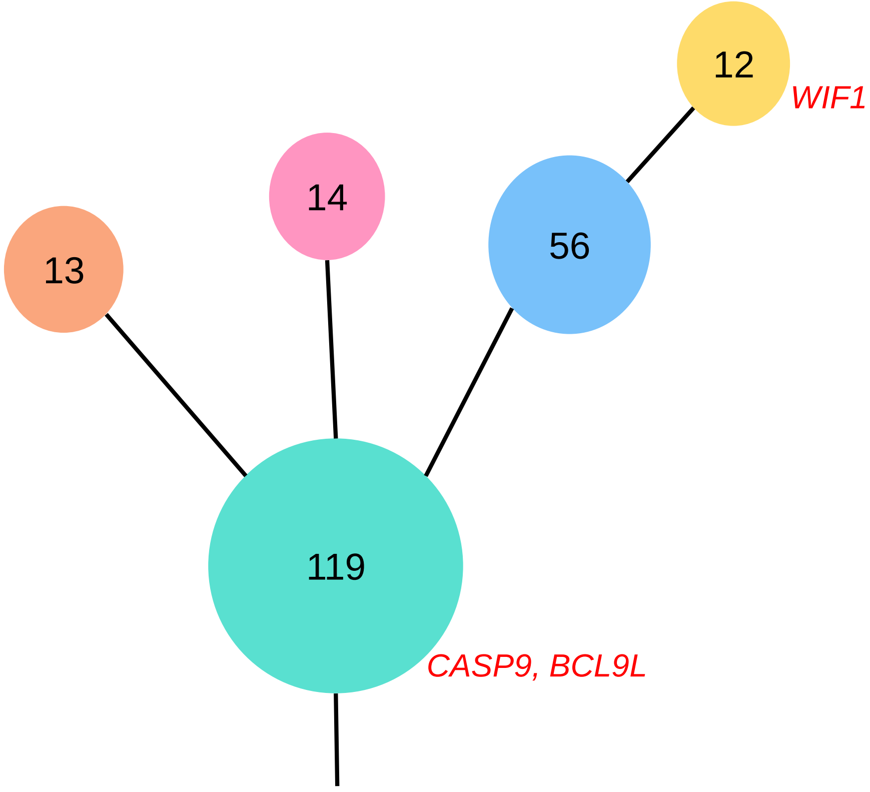

12
WIF1
14
13
56
119
CASP9, BCL9L

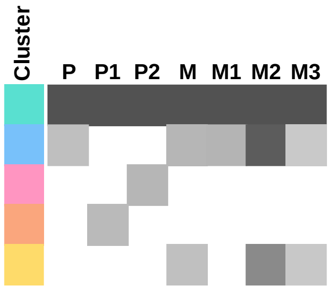

Cluster
P P1 P2 M M1 M2 M3

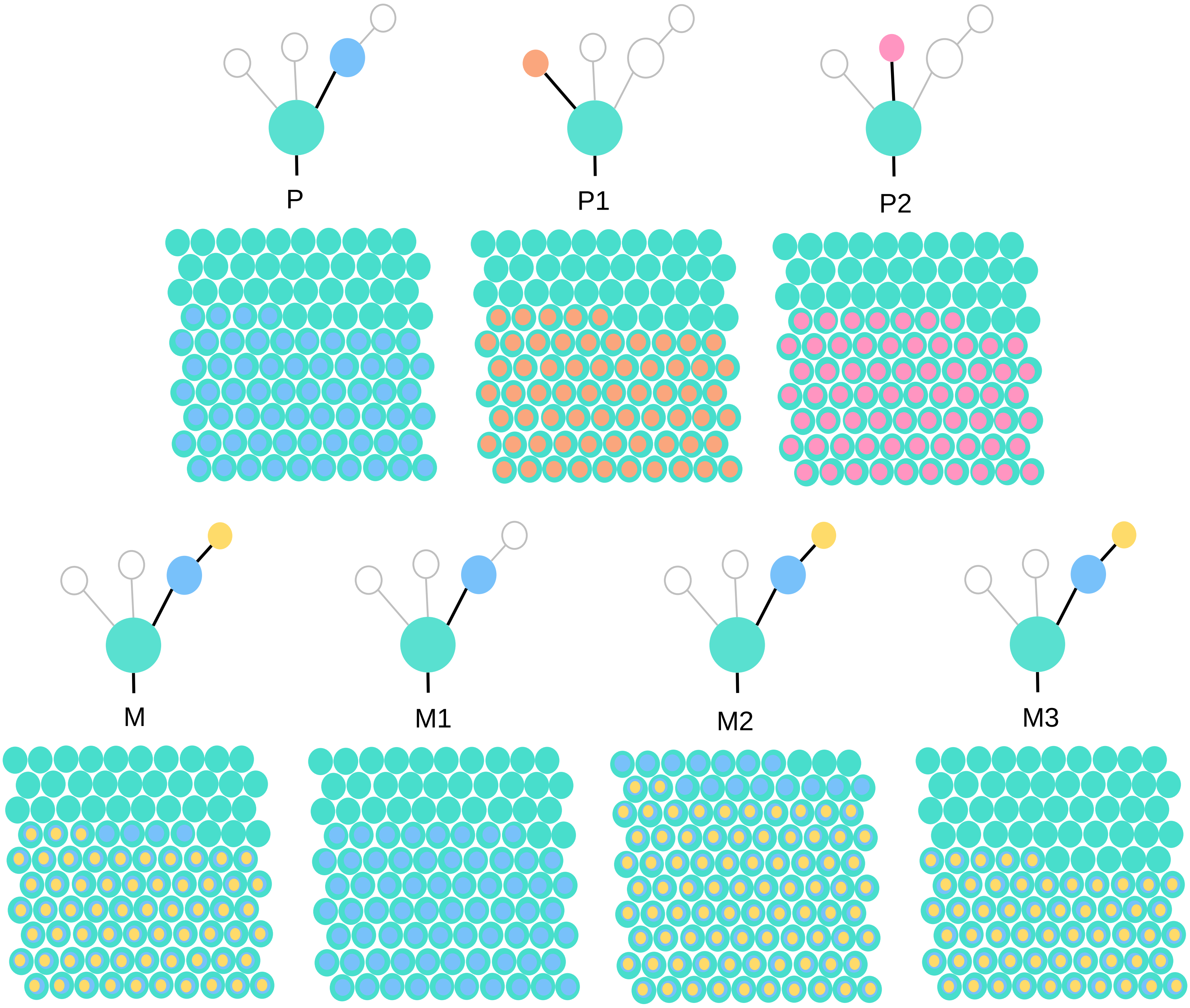

P
P1
P2
M
M1
M2
M3

112-058

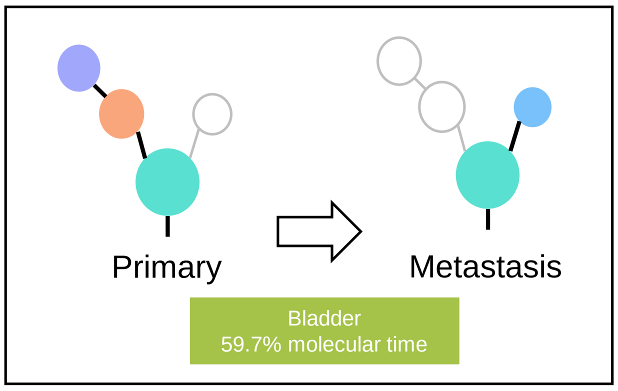
Primary
Metastasis
Bladder
59.7% molecular time

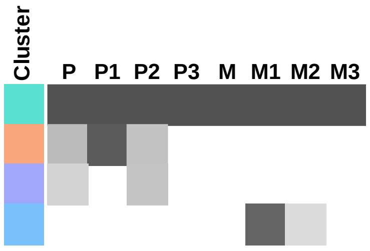
Cluster
P
P1
P2
P3
M
M1
M2
M3

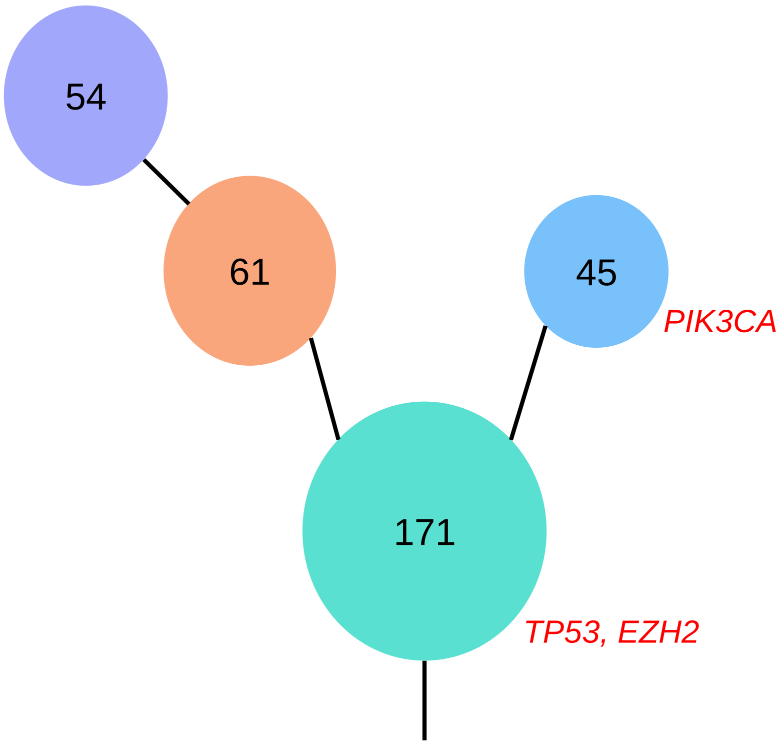
54
61
45
PIK3CA
171
TP53, EZH2

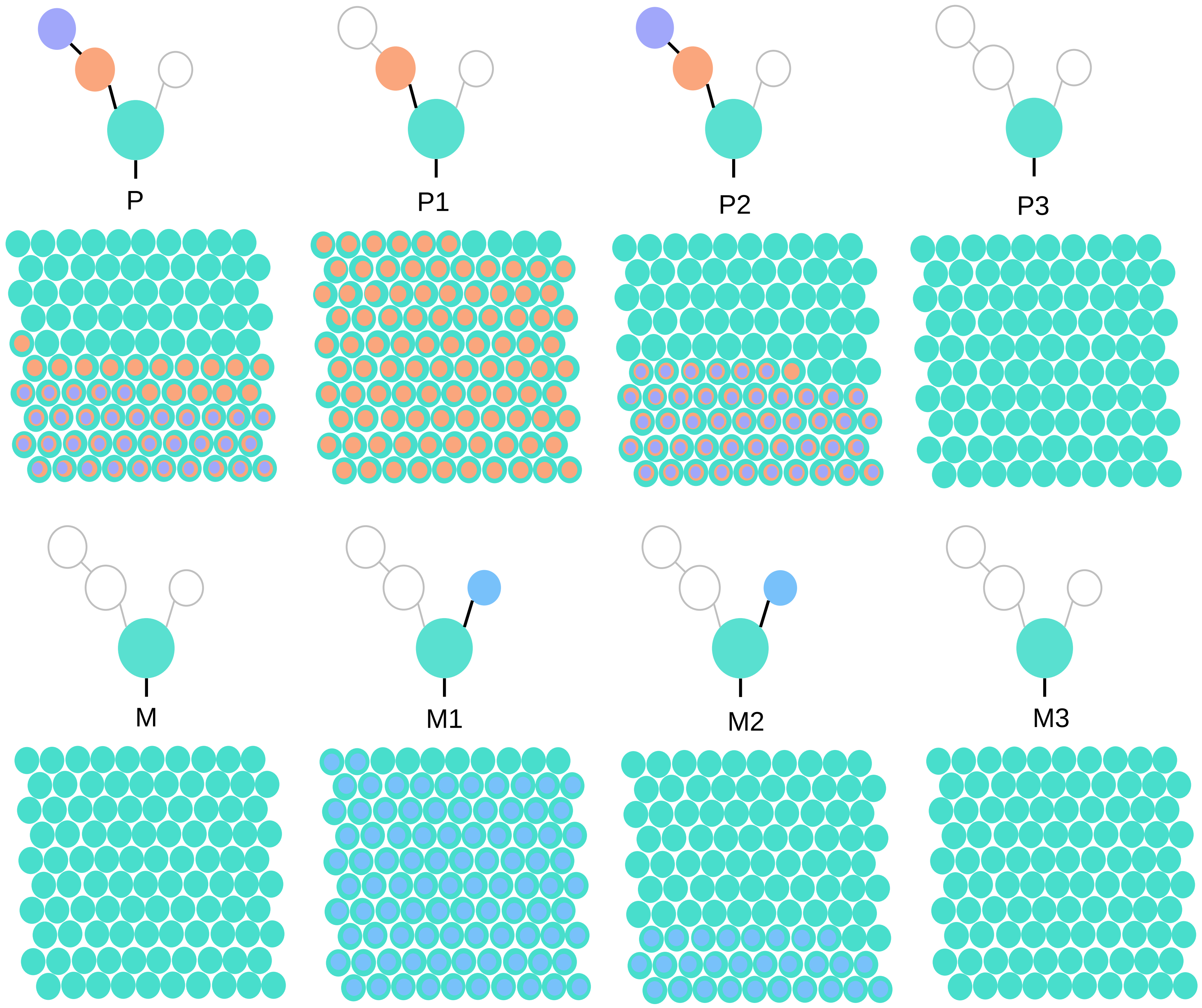
P
P1
P2
P3
M
M1
M2
M3

112-091

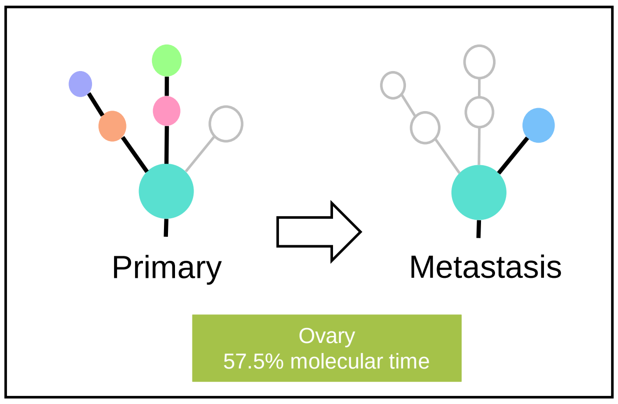
Primary
Metastasis
Ovary
57.5% molecular time

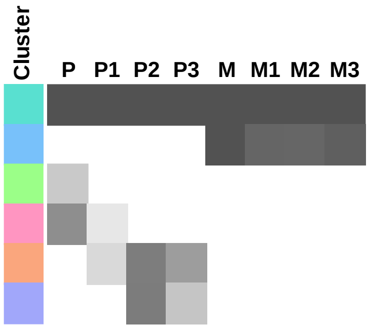
Cluster
P P1 P2 P3 M M1 M2 M3

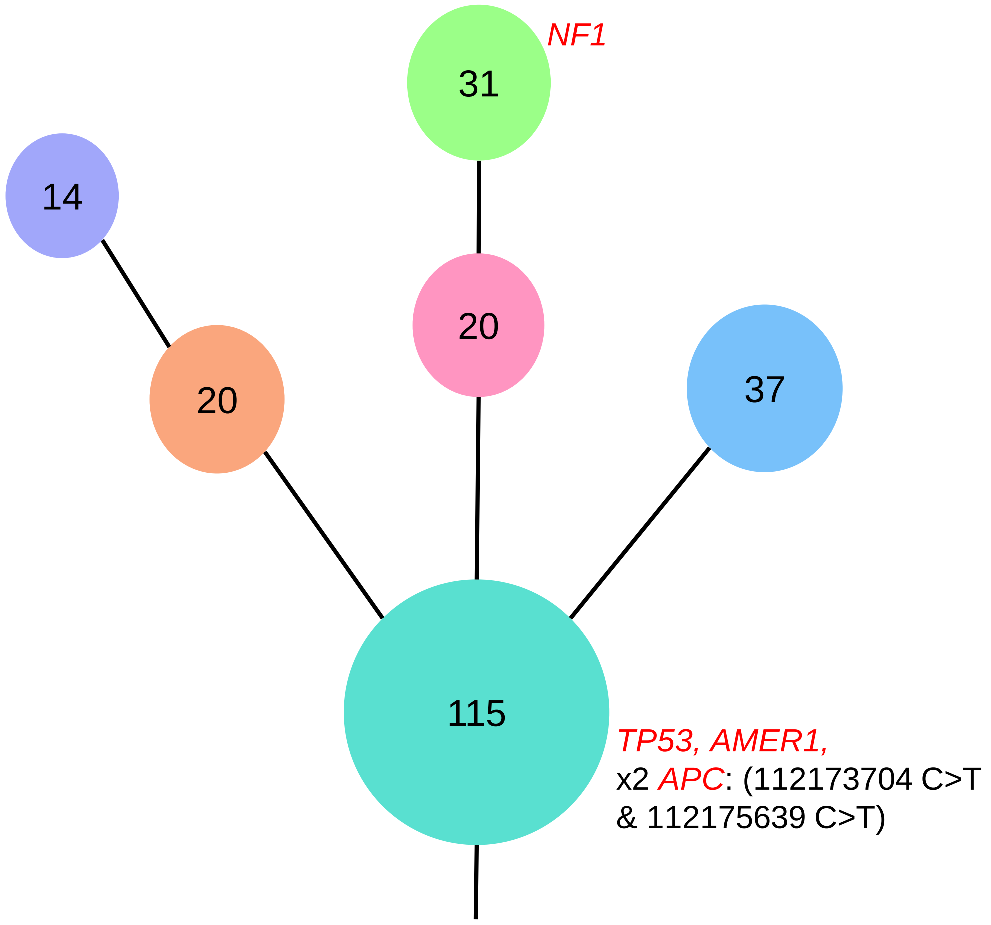
NF1
31
14
20
20
37
115
TP53, AMER1,
x2 APC: (112173704 C>T
& 112175639 C>T)

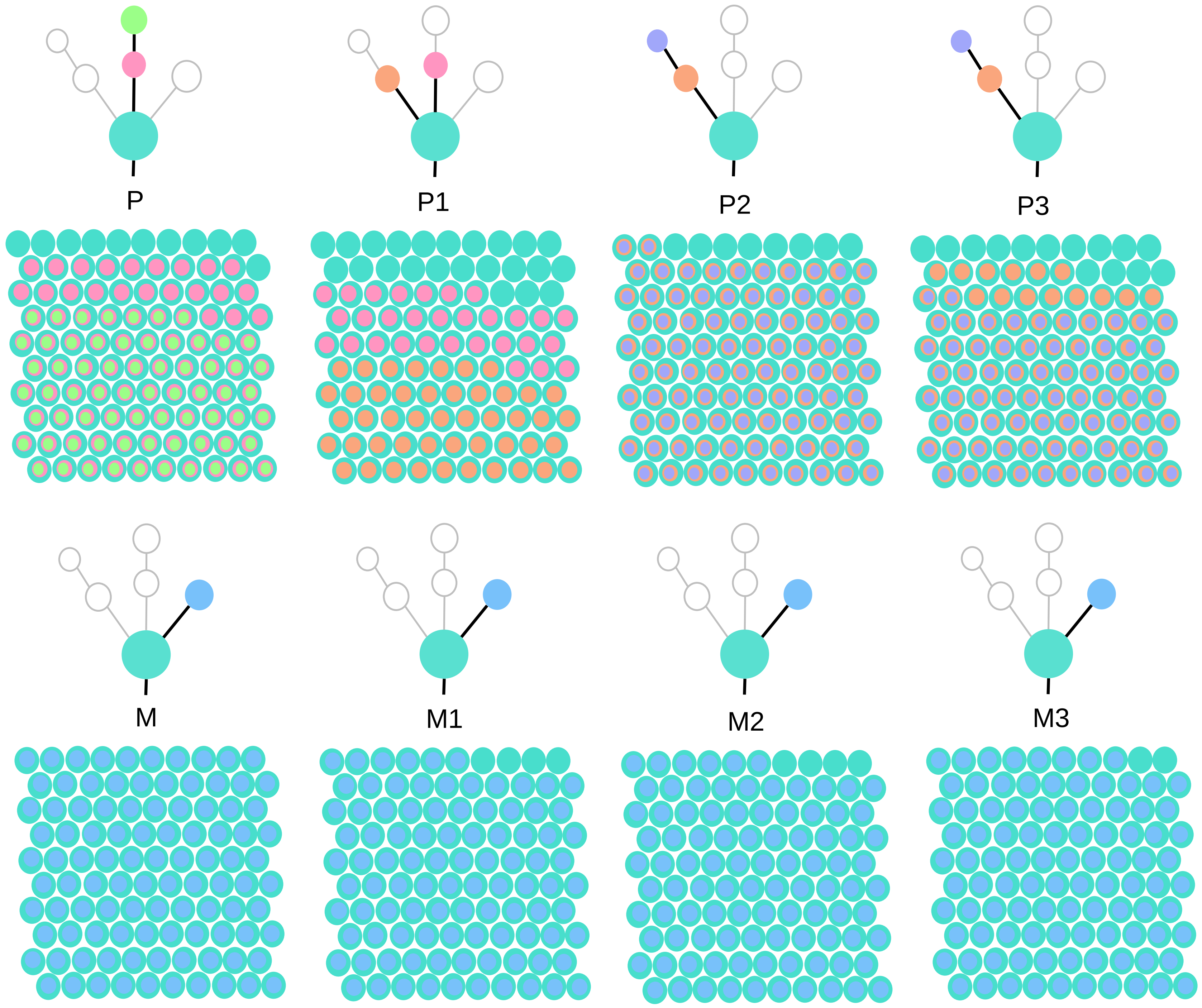
P
P1
P2
P3
M
M1
M2
M3

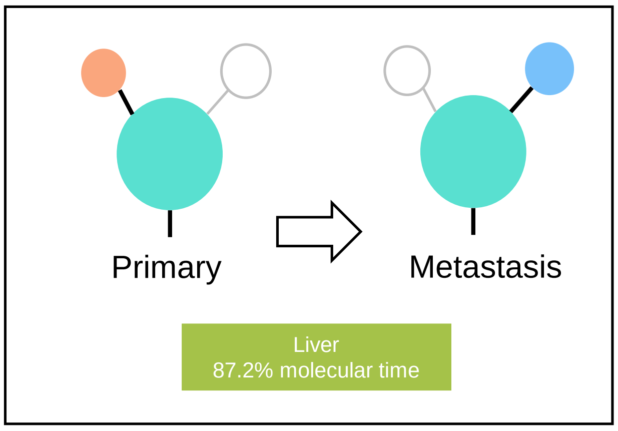
Primary
Metastasis
Liver
87.2% molecular time

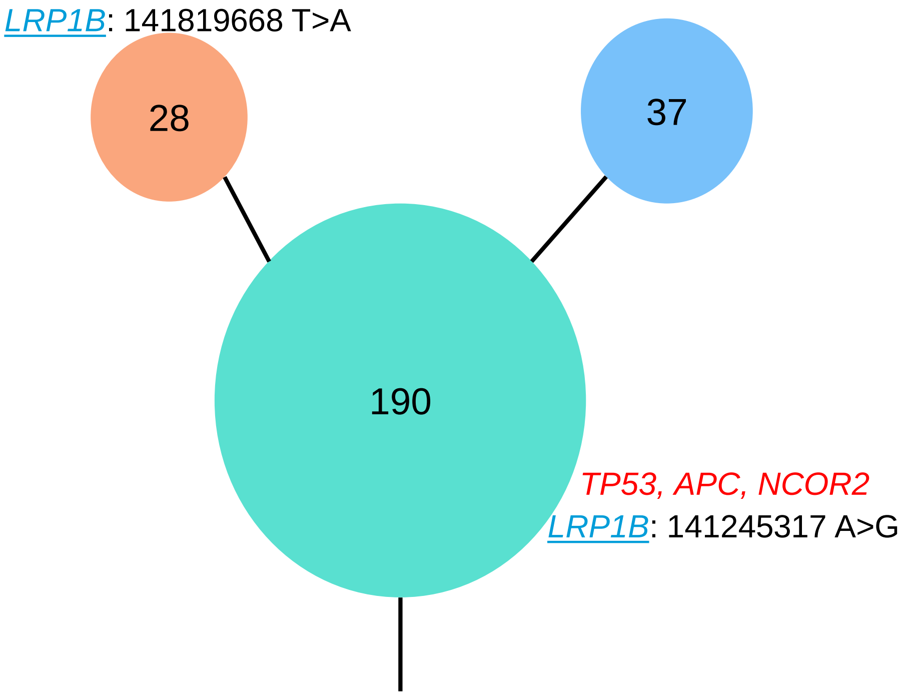
LRP1B: 141819668 T>A
28
37
190
TP53, APC, NCOR2
LRP1B: 141245317 A>G

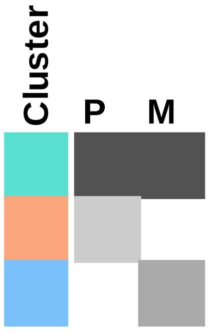
Cluster
P
M

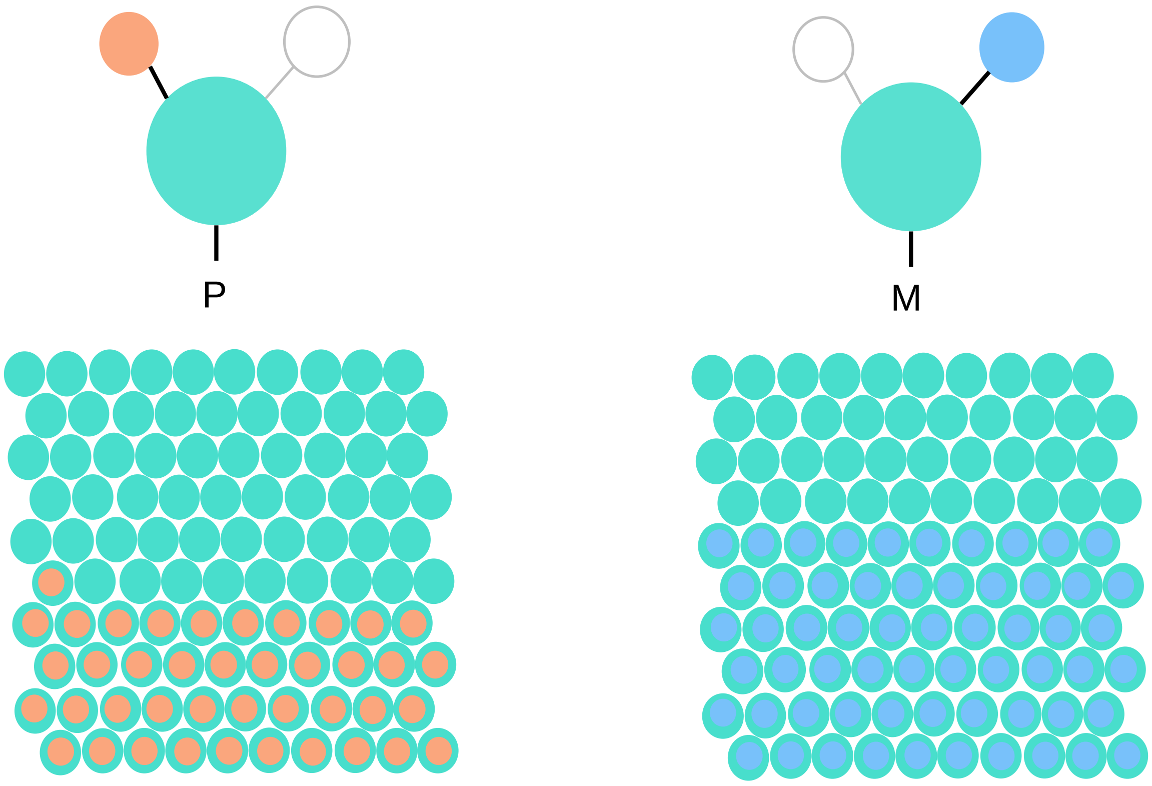
P
M

197-064

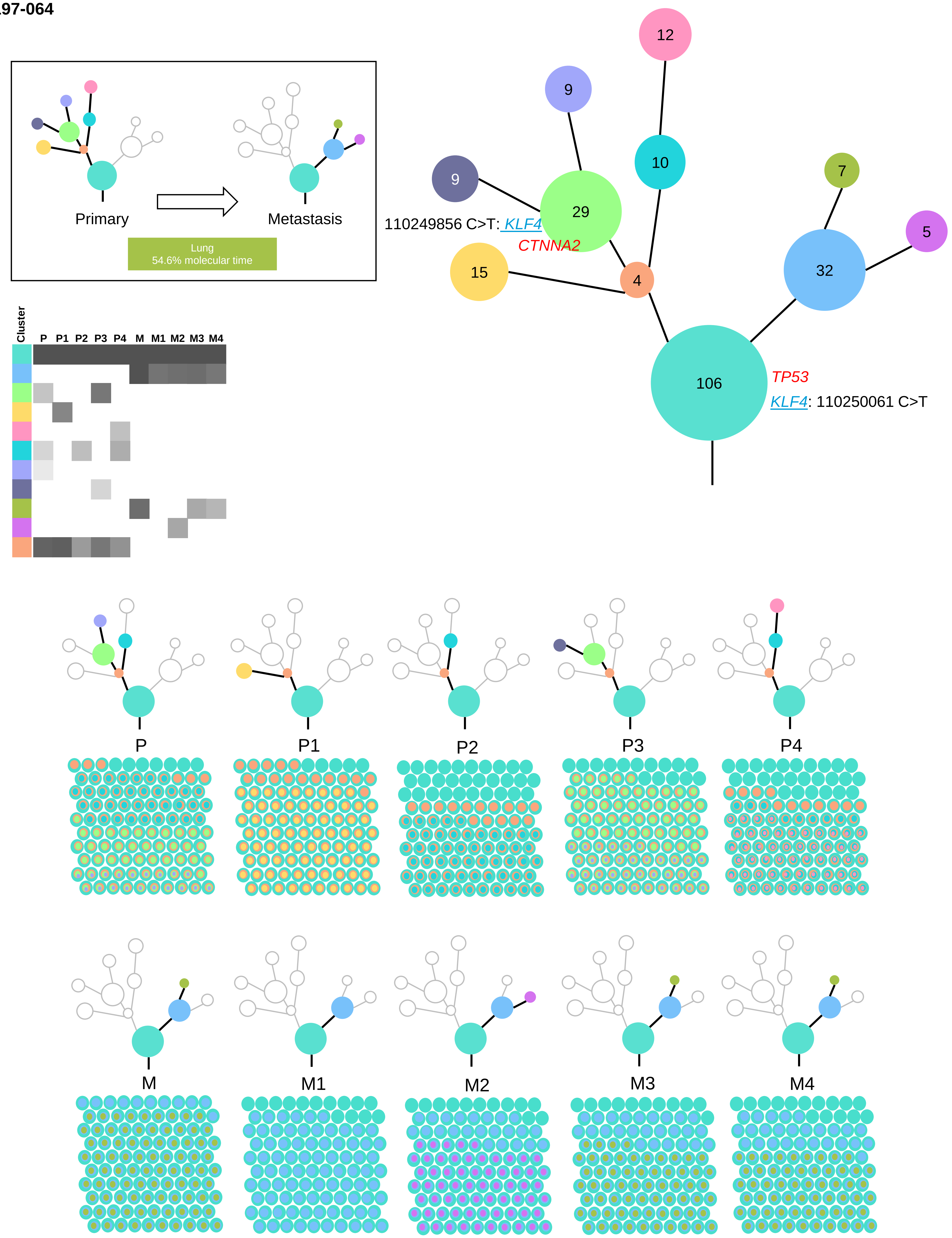

Primary
Metastasis
Lung
54.6% molecular time
12
9
10
9
29
7
5
110249856 C>T: KLF4
CTNNA2
32
15
4
106
TP53
KLF4: 110250061 C>T
Cluster
P P1 P2 P3 P4 M M1 M2 M3 M4
P
P1
P2
P3
P4
M
M1
M2
M3
M4

197-144

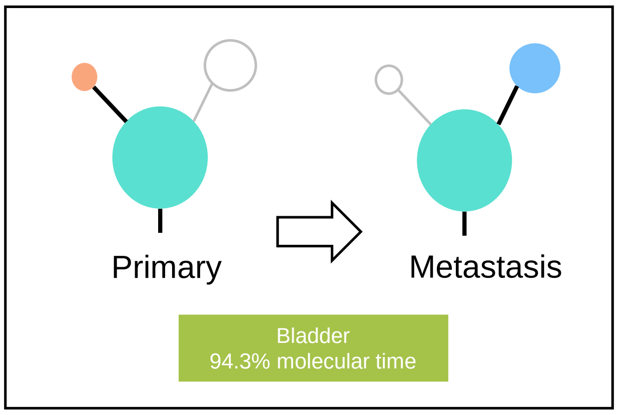
Primary
Metastasis
Bladder
94.3% molecular time

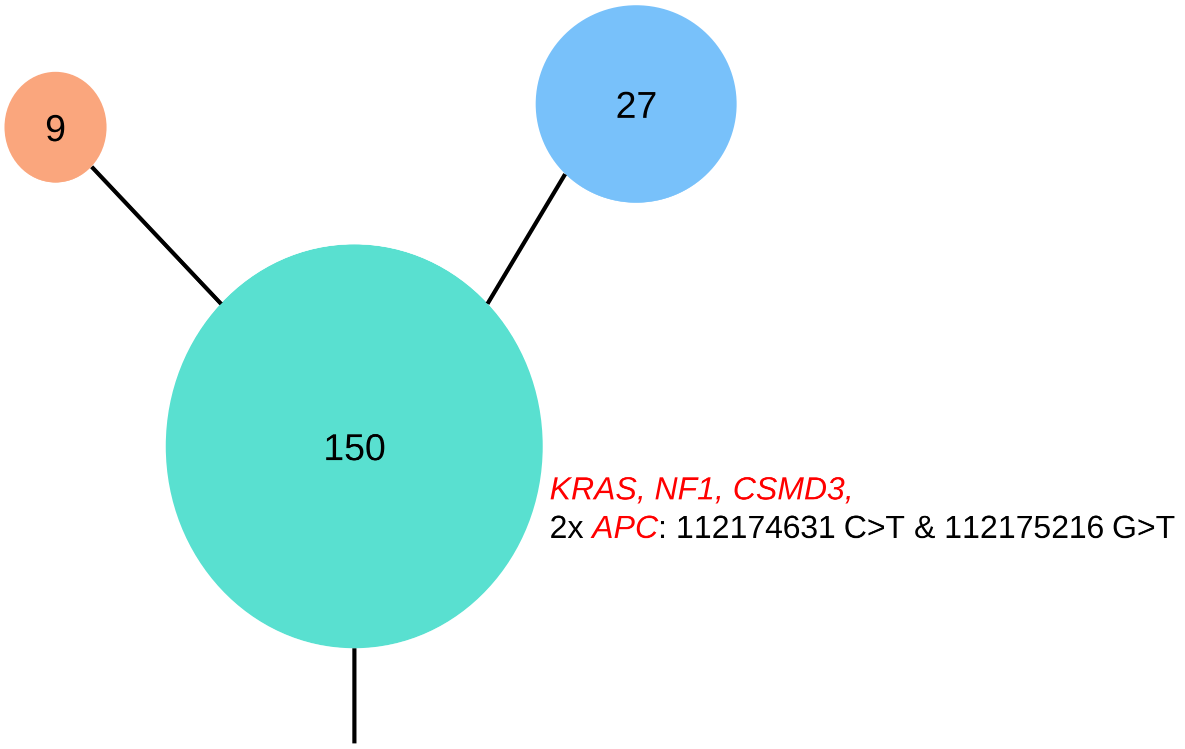
9
27
150
KRAS, NF1, CSMD3,
2x APC: 112174631 C>T & 112175216 G>T

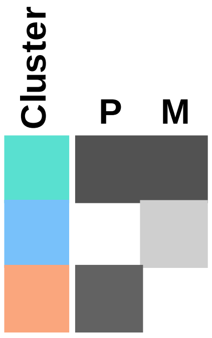
Cluster
P
M

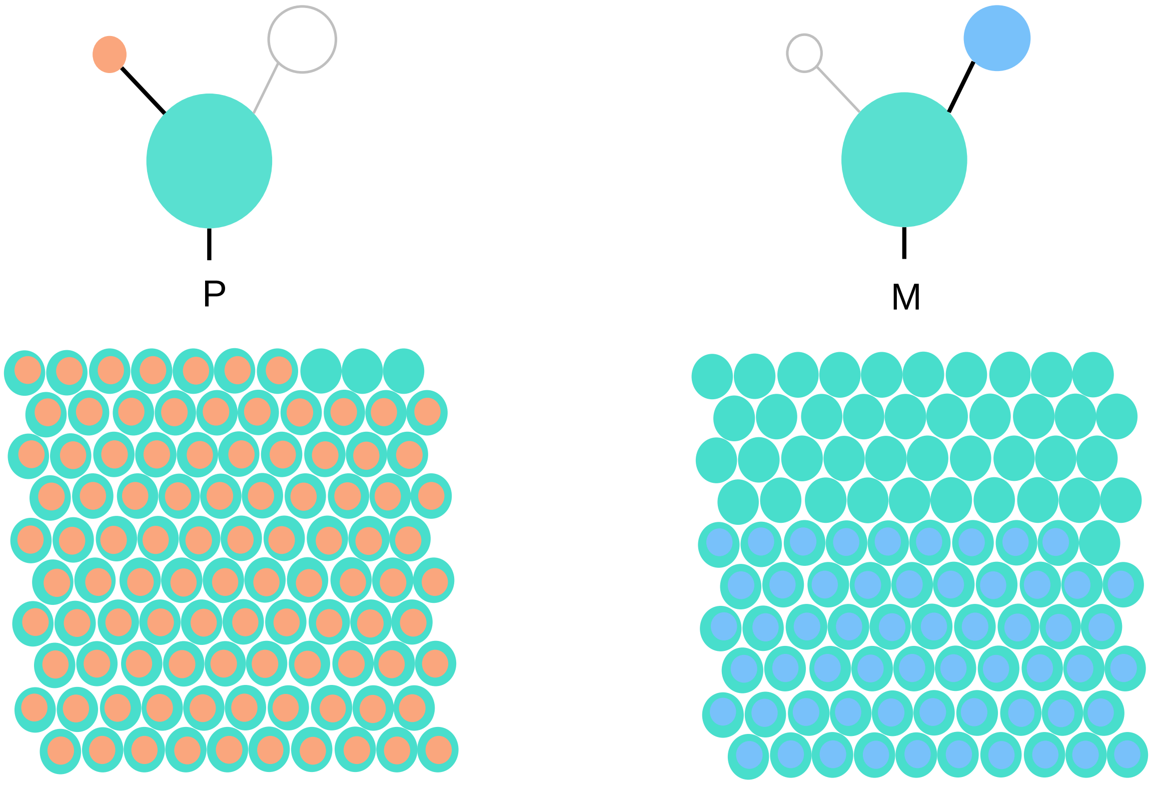
P
M

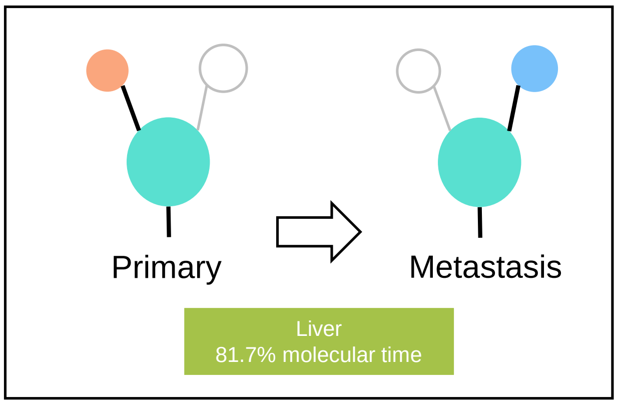
Primary
Metastasis
Liver
81.7% molecular time

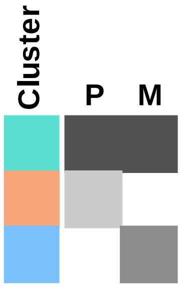
Cluster
P
M

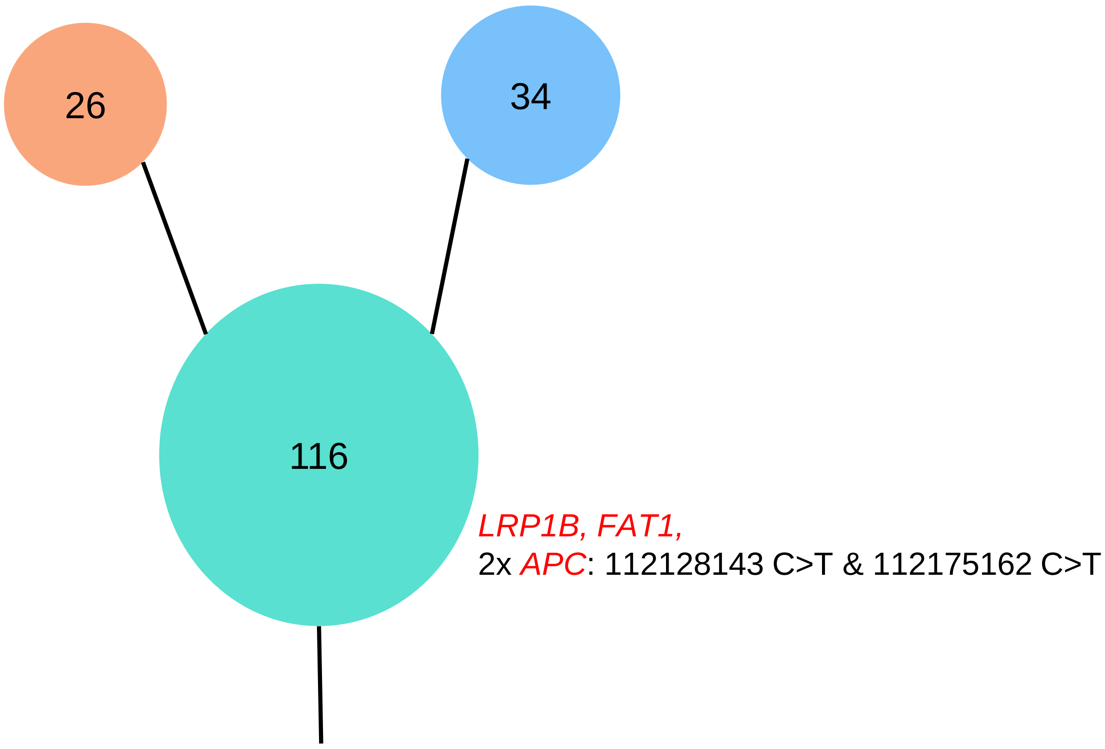
26
34
116
LRP1B, FAT1,
2x APC: 112128143 C>T & 112175162 C>T

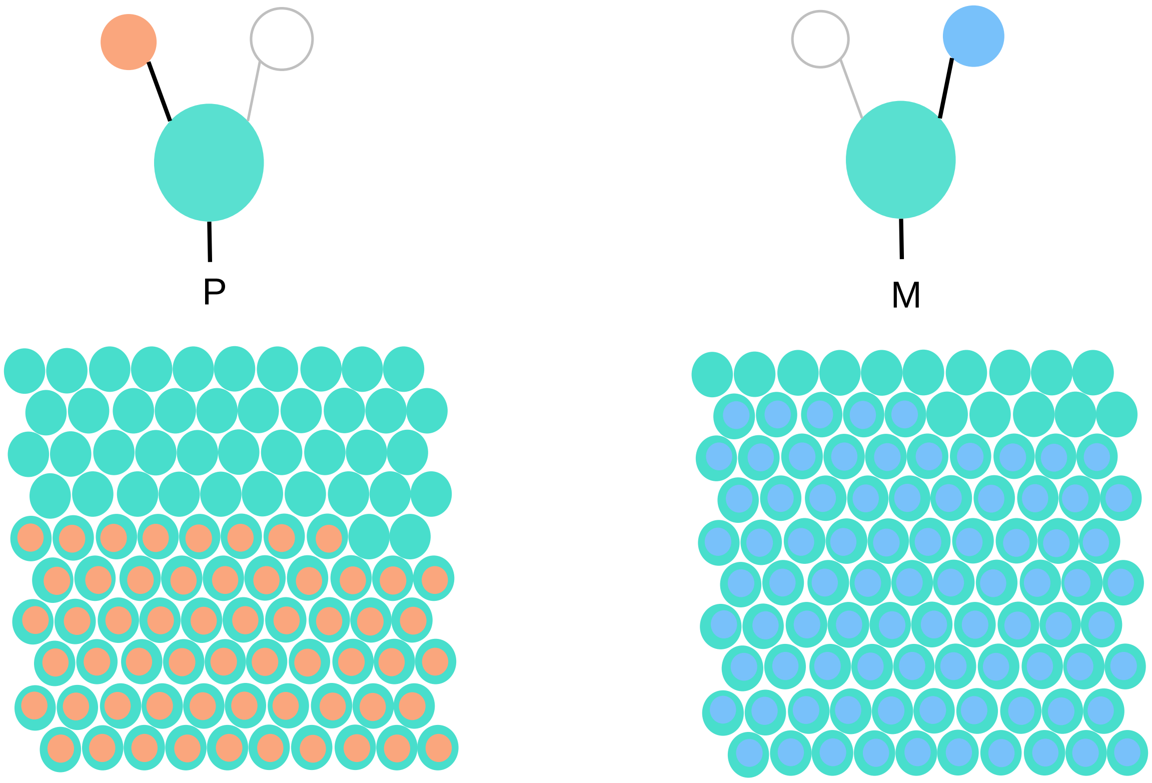
P
M

306-052

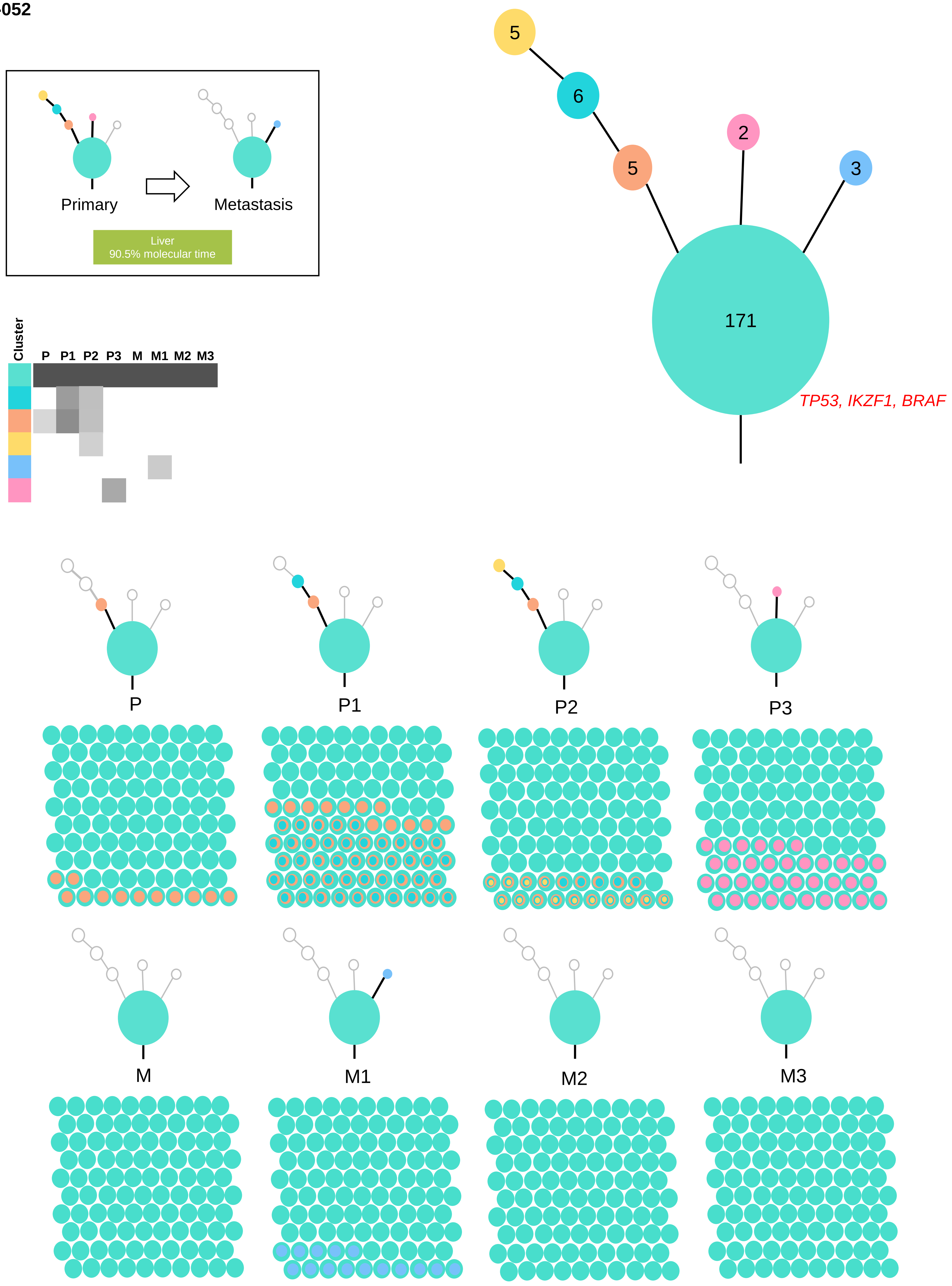

Primary
Metastasis
Liver
90.5% molecular time
Cluster
P P1 P2 P3 M M1 M2 M3
5
6
5
2
3
171
TP53, IKZF1, BRAF
P
P1
P2
P3
M
M1
M2
M3

306-090

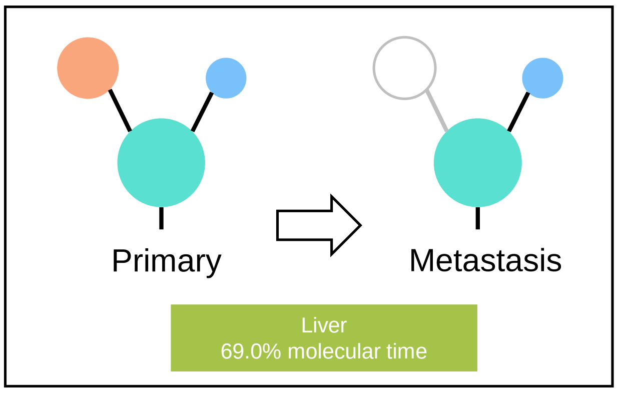

Primary
Metastasis
Liver
69.0% molecular time

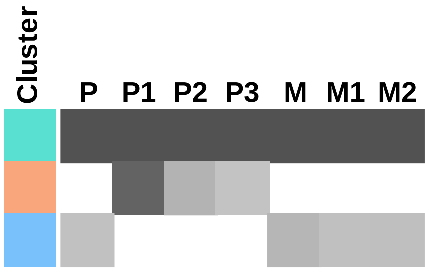

Cluster
P
P1
P2
P3
M
M1
M2

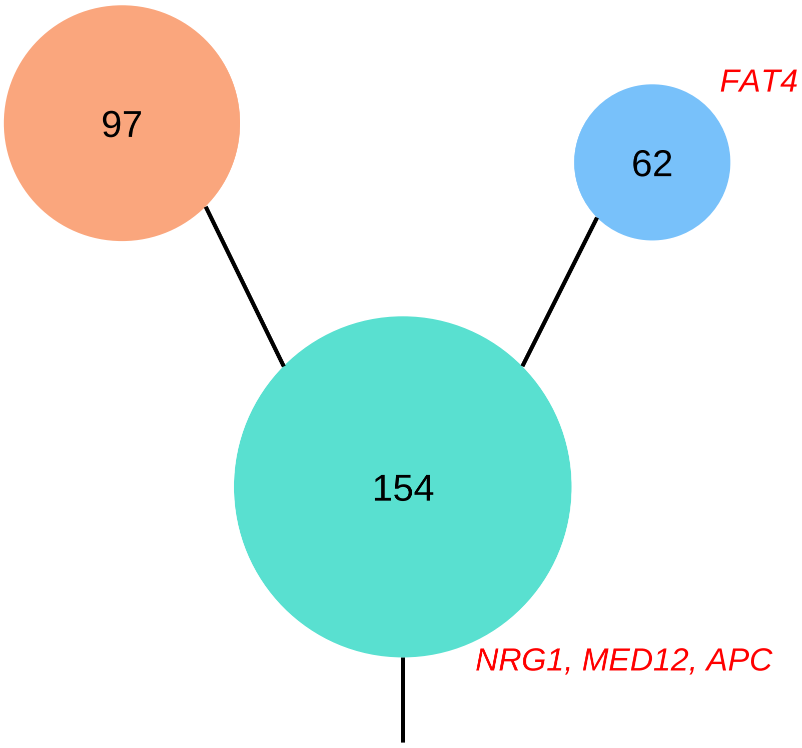

97
62
154
FAT4
NRG1, MED12, APC

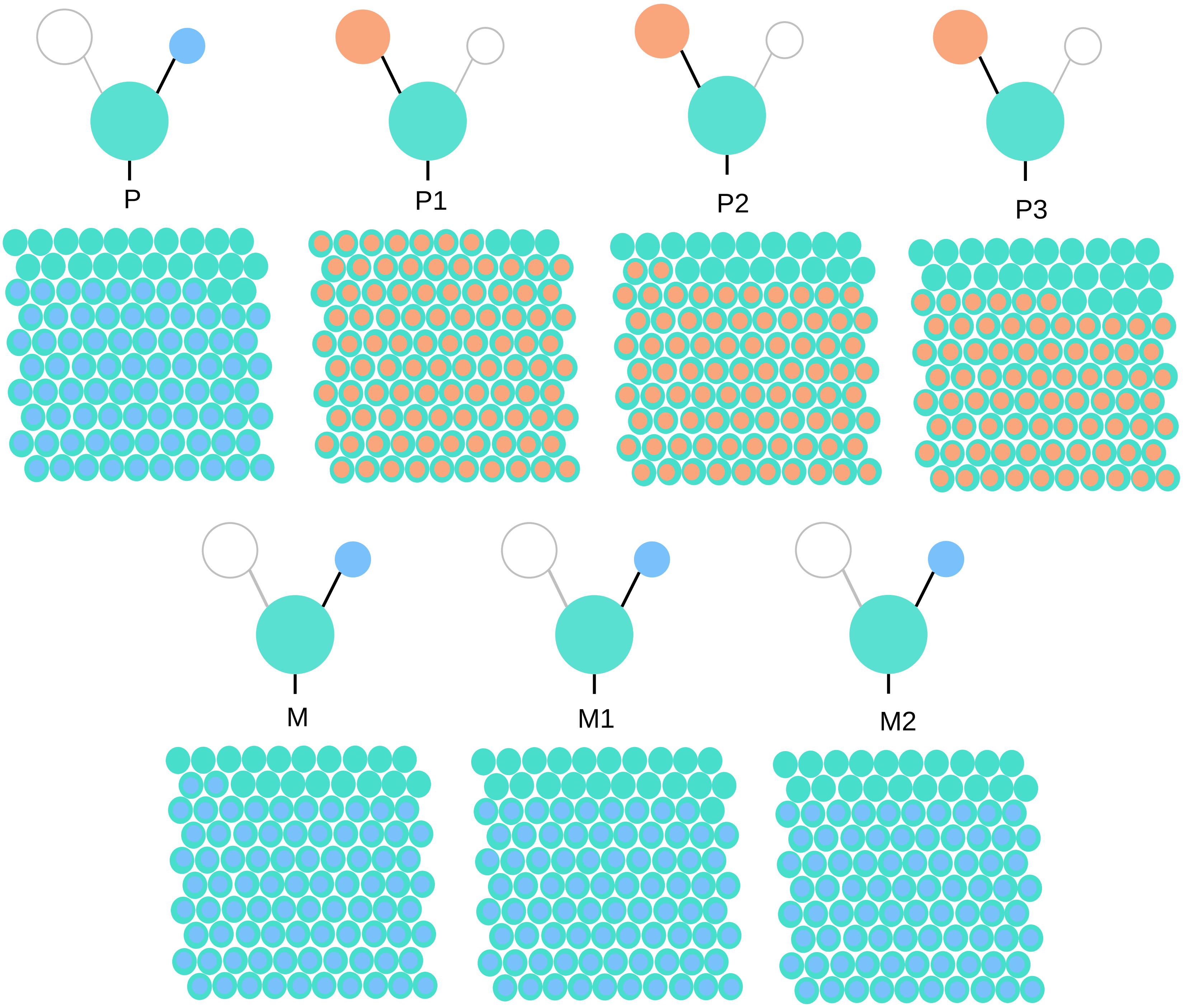

P
P1
P2
P3
M
M1
M2

306-165

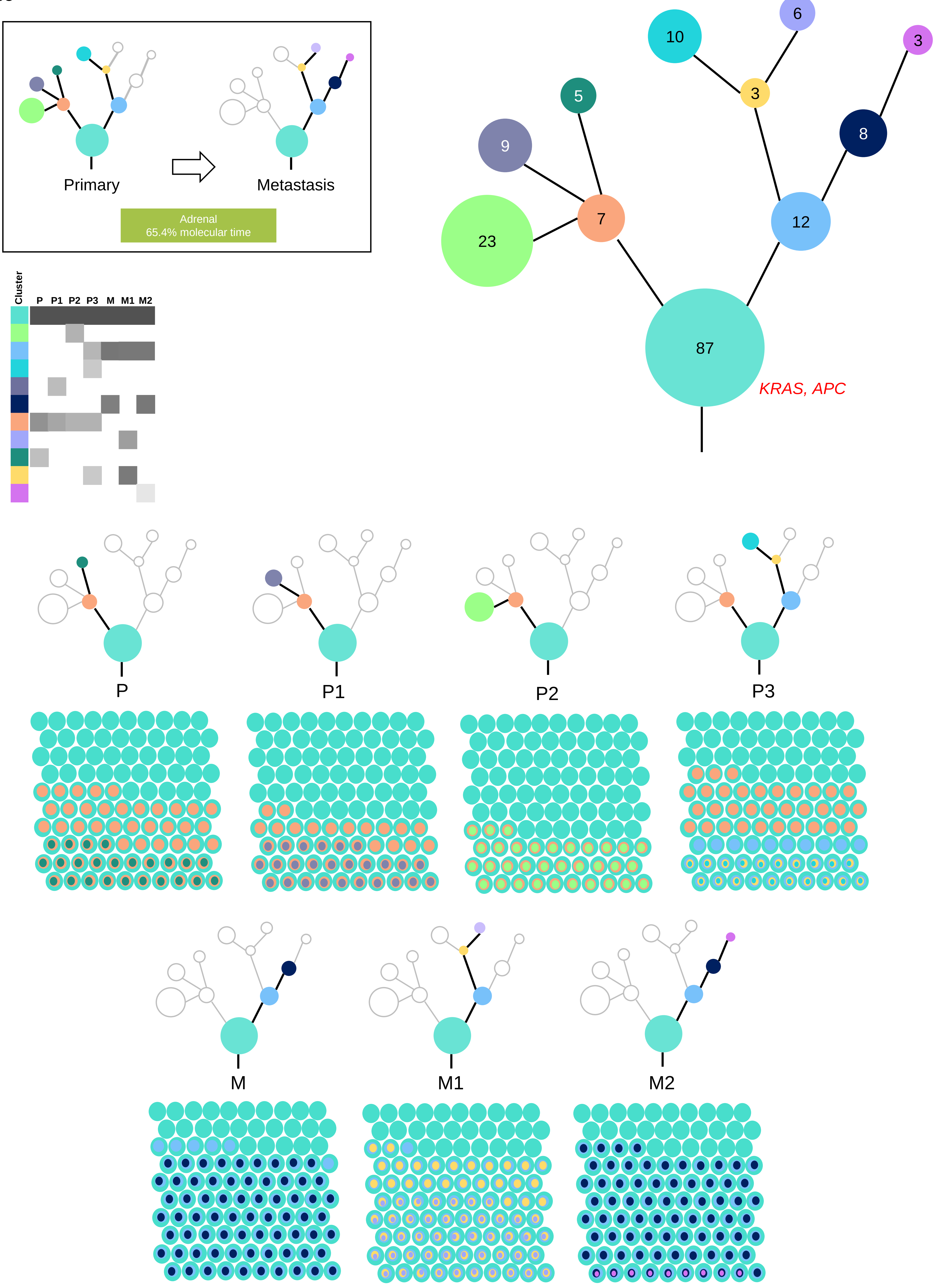
Primary
Metastasis
Adrenal
65.4% molecular time
Cluster
P P1 P2 P3 M M1 M2
10
6
3
5
9
3
8
7
12
23
87
KRAS, APC
P
P1
P2
P3
M
M1
M2

306-171

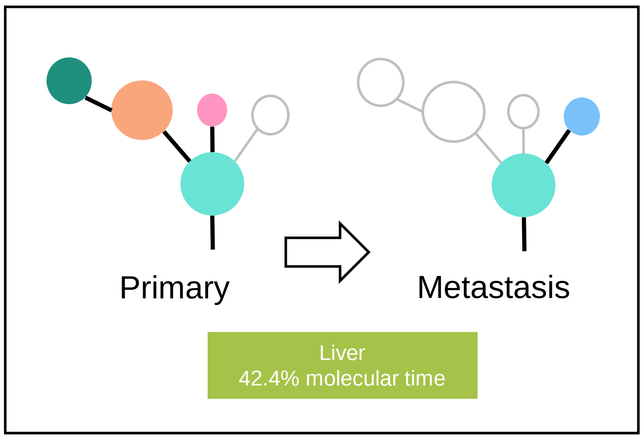
Primary
Metastasis
Liver
42.4% molecular time

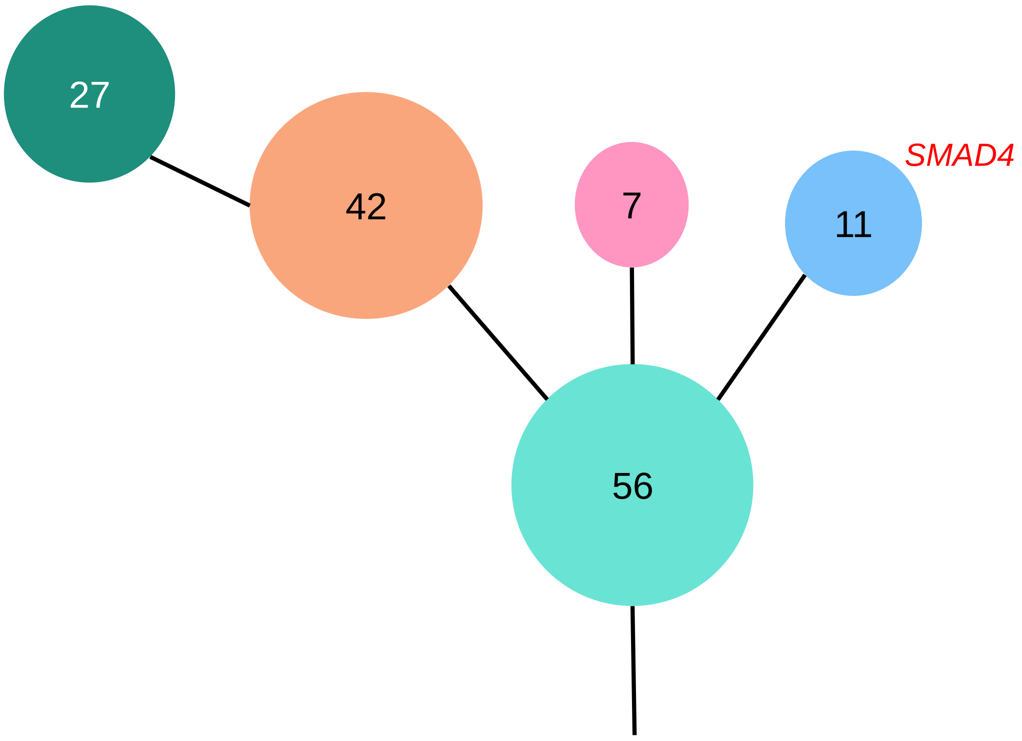
27
42
7
11
SMAD4
56

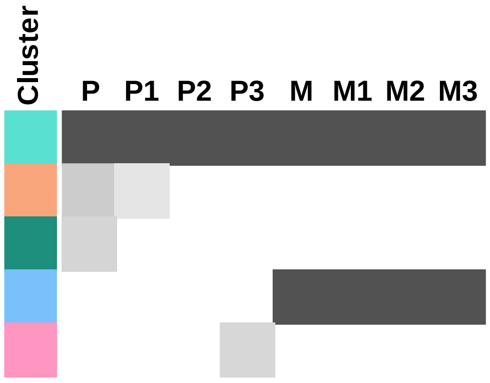
Cluster
P P1 P2 P3 M M1 M2 M3

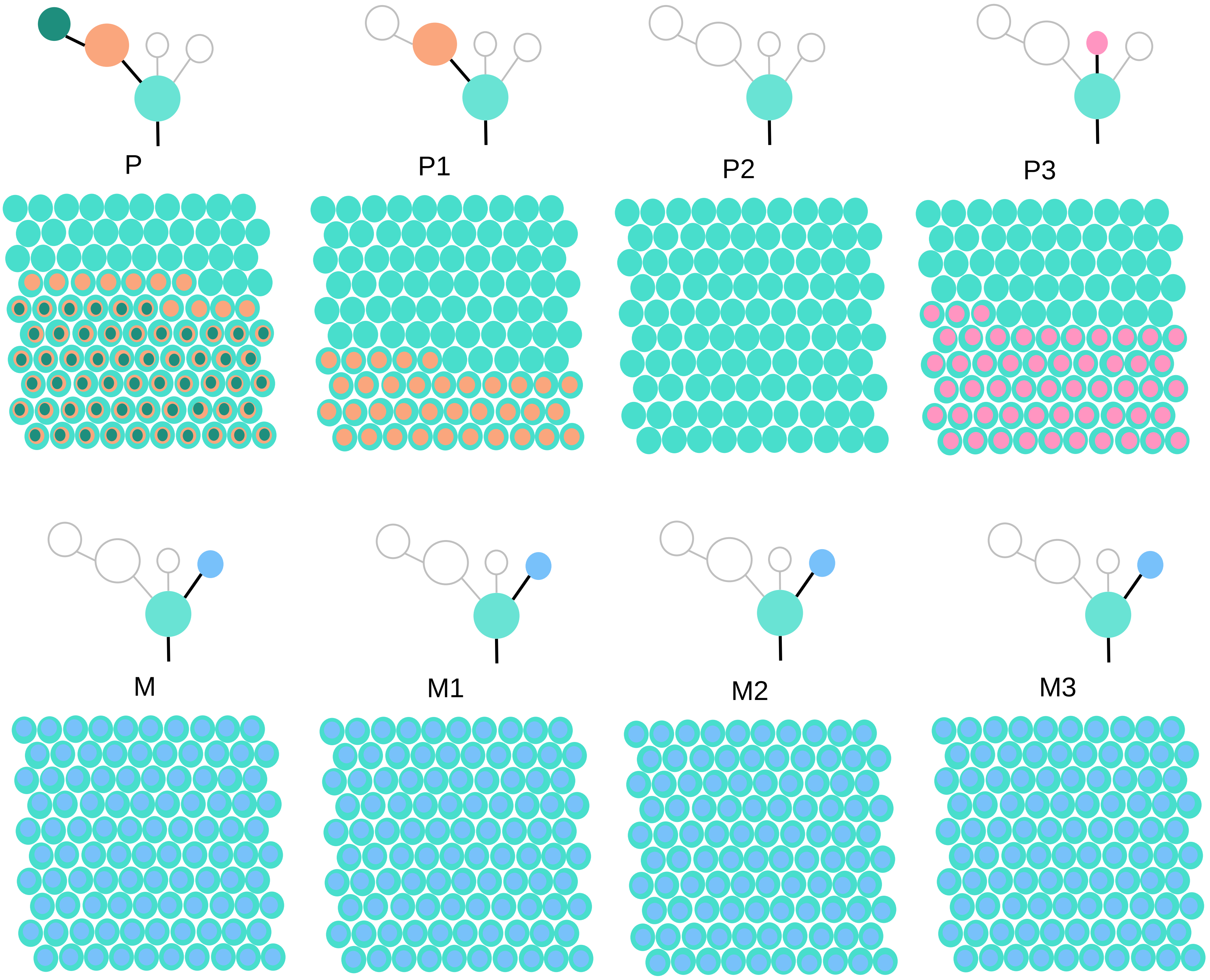
P
P1
P2
P3
M
M1
M2
M3

306-212

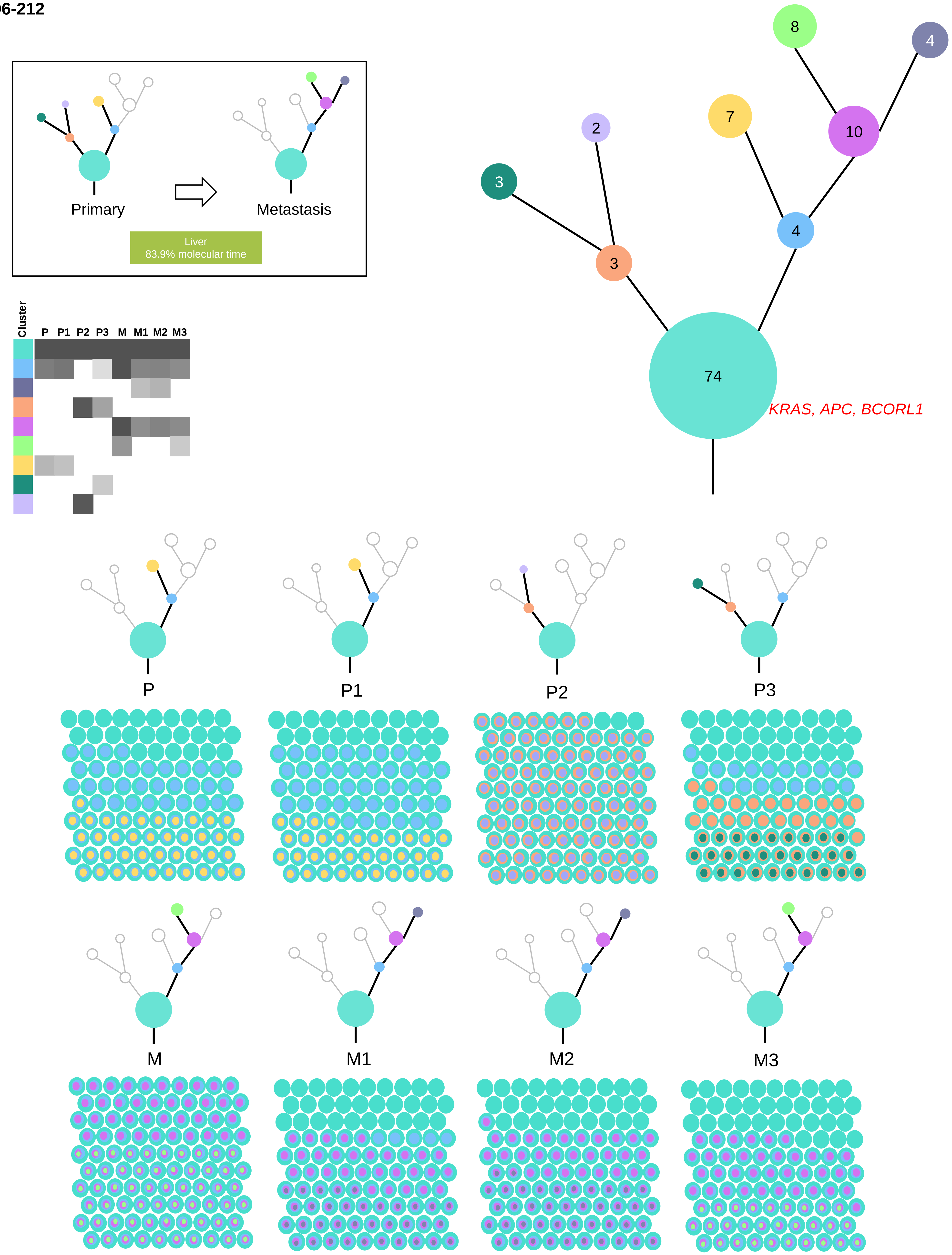
Primary
Metastasis
Liver
83.9% molecular time
Cluster
P P1 P2 P3 M M1 M2 M3
74
3
3
2
4
7
10
8
4
KRAS, APC, BCORL1
P
P1
P2
P3
M
M1
M2
M3

306-214

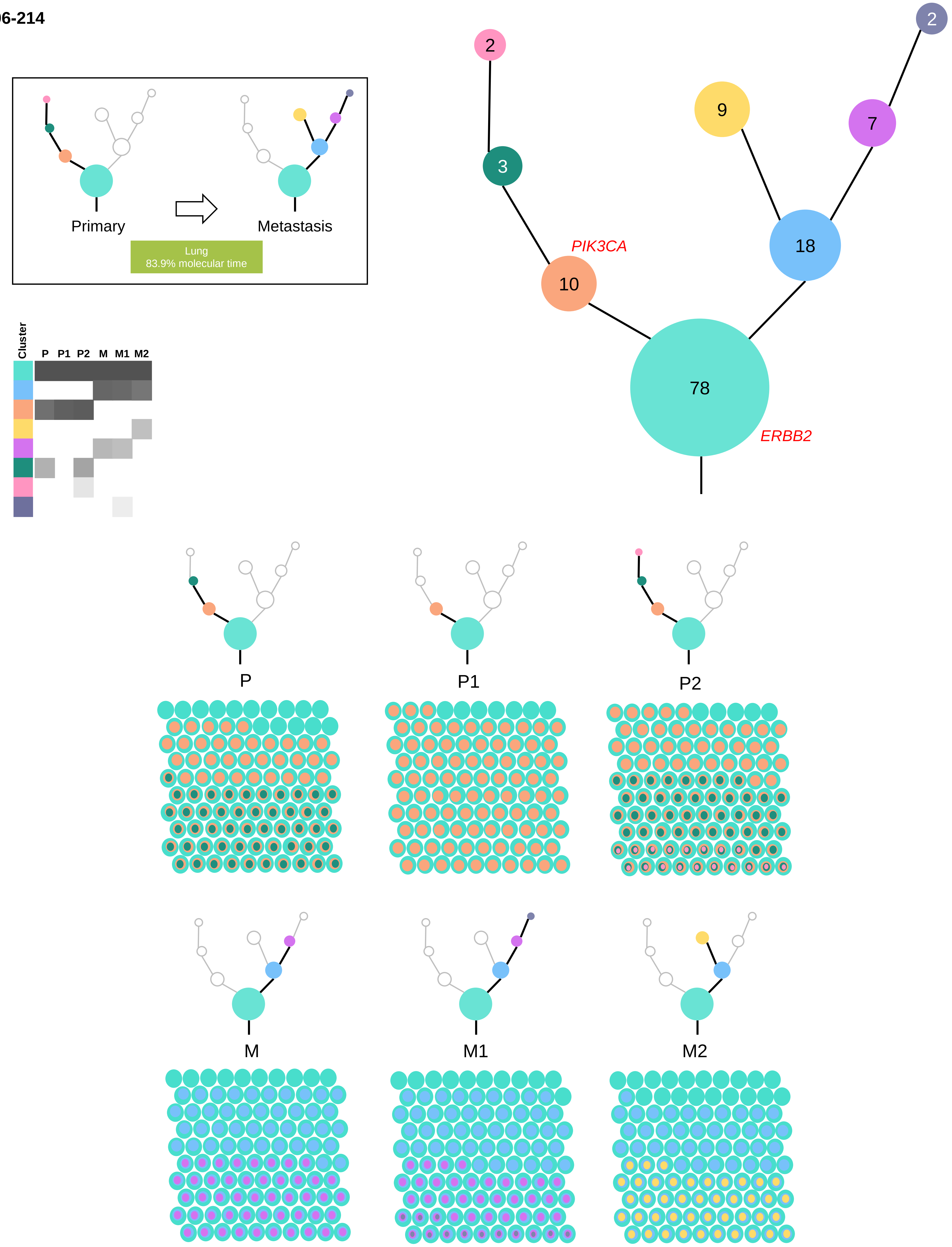
Primary
Metastasis
Lung
83.9% molecular time
Cluster
P P1 P2 M M1 M2
2
3
10
PIK3CA
78
ERBB2
9
18
7
2
P
P1
P2
M
M1
M2

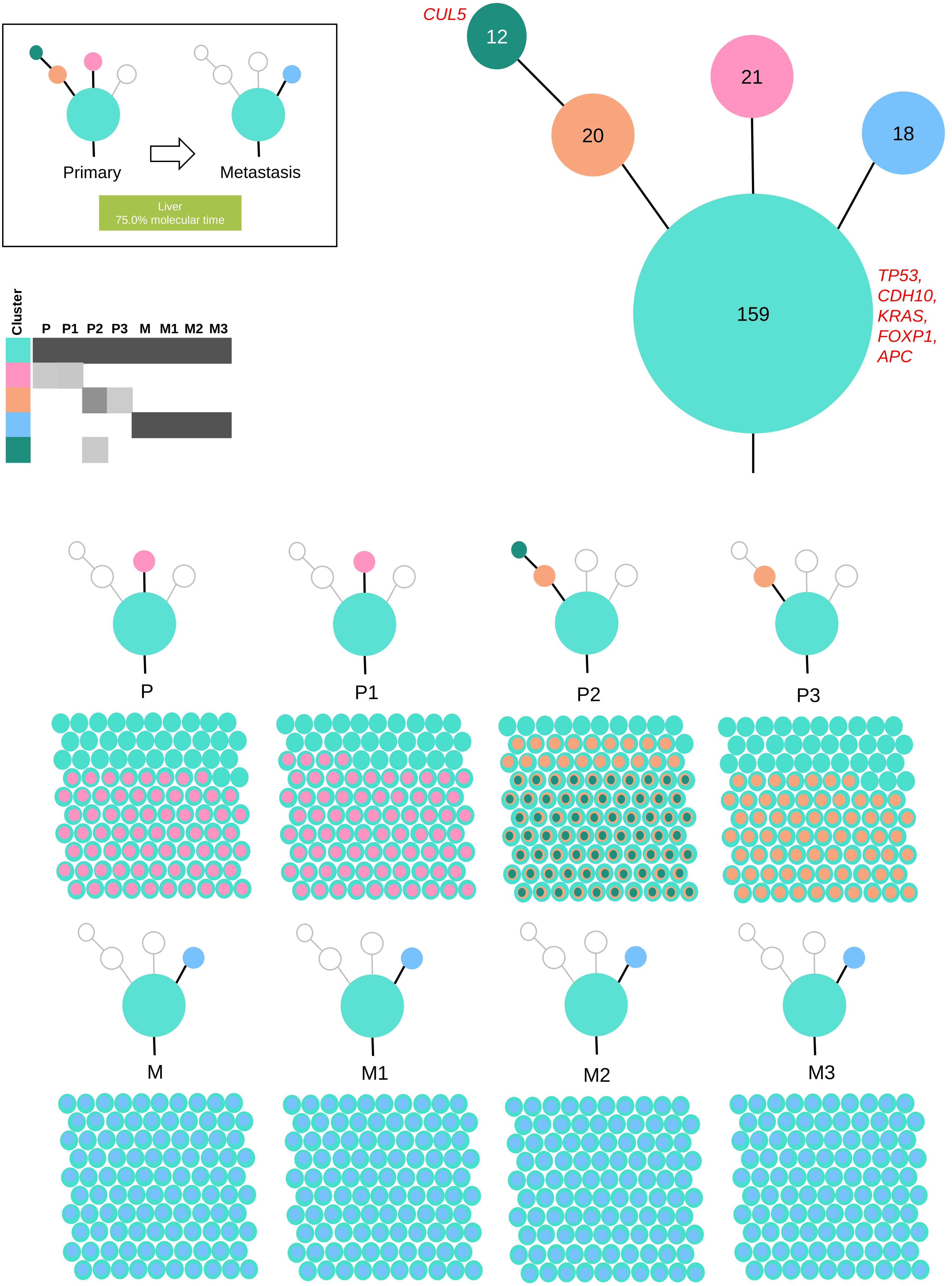
Primary
Metastasis
Liver
75.0% molecular time
CUL5
12
20
21
18
159
TP53,
CDH10,
KRAS,
FOXP1,
APC
Cluster
P
P1
P2
P3
M
M1
M2
M3

306-285

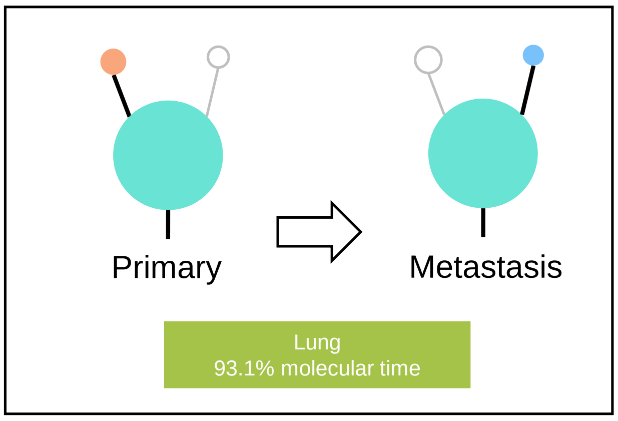
Primary
Metastasis
Lung
93.1% molecular time

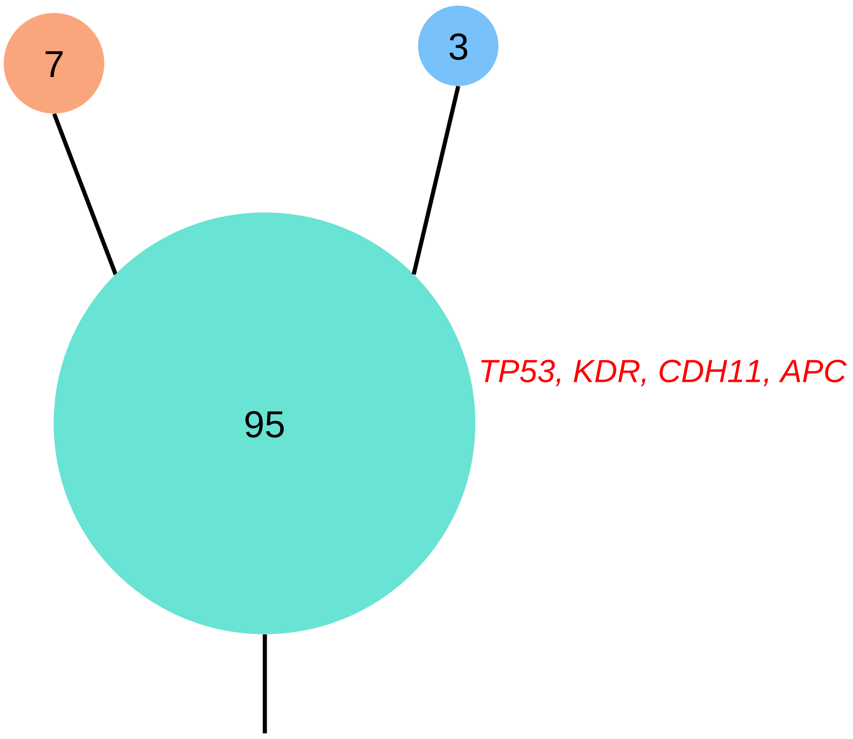
7
3
95
TP53, KDR, CDH11, APC

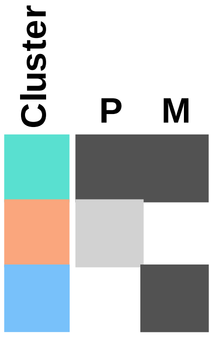
Cluster
P
M

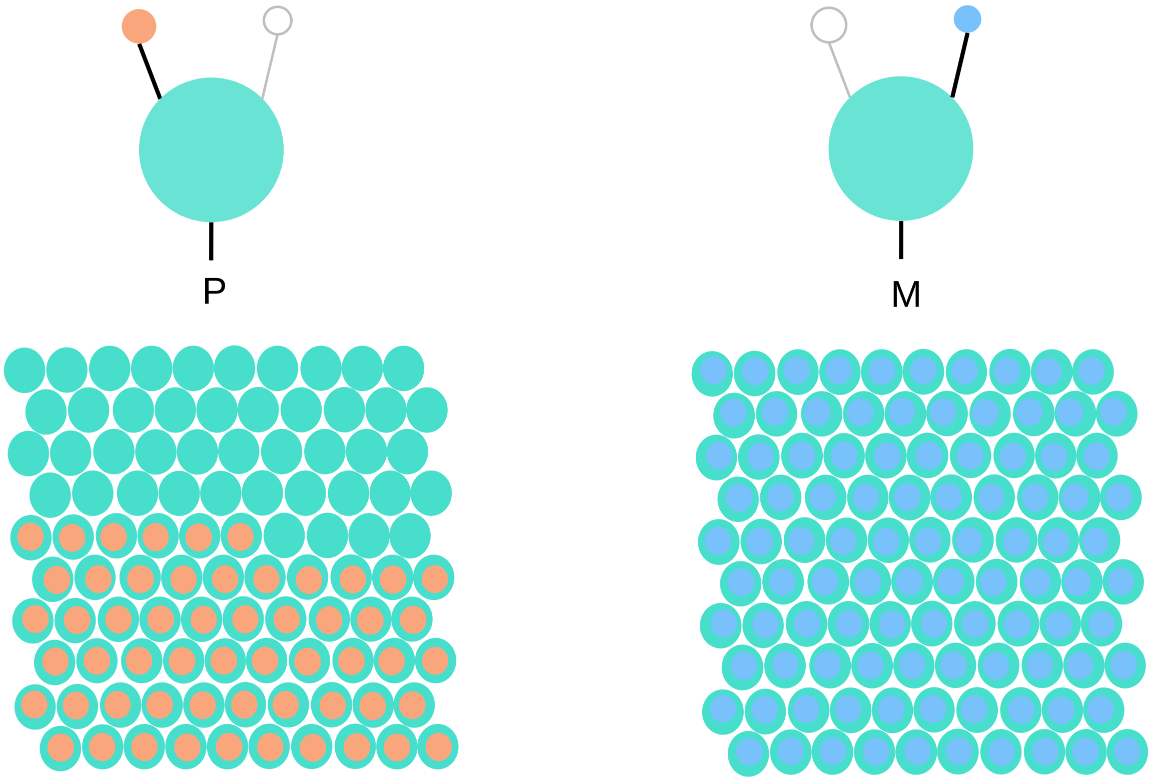
P
M

CRC-139

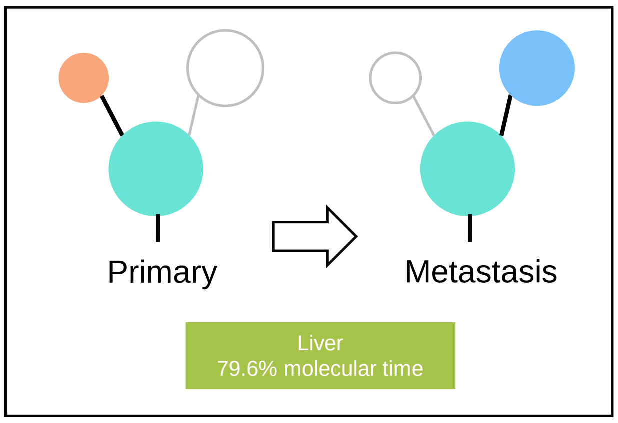
Primary
Metastasis
Liver
79.6% molecular time

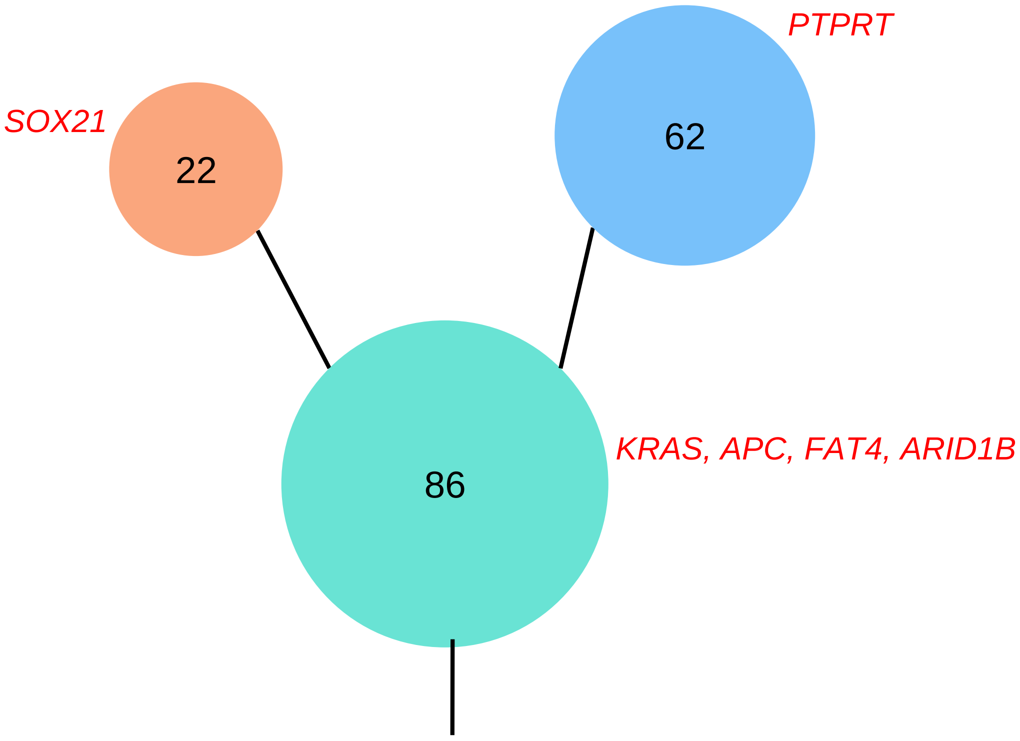
SOX21
22
PTPRT
62
86
KRAS, APC, FAT4, ARID1B

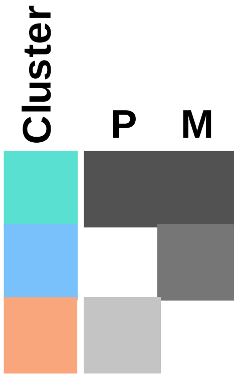
Cluster
P
M

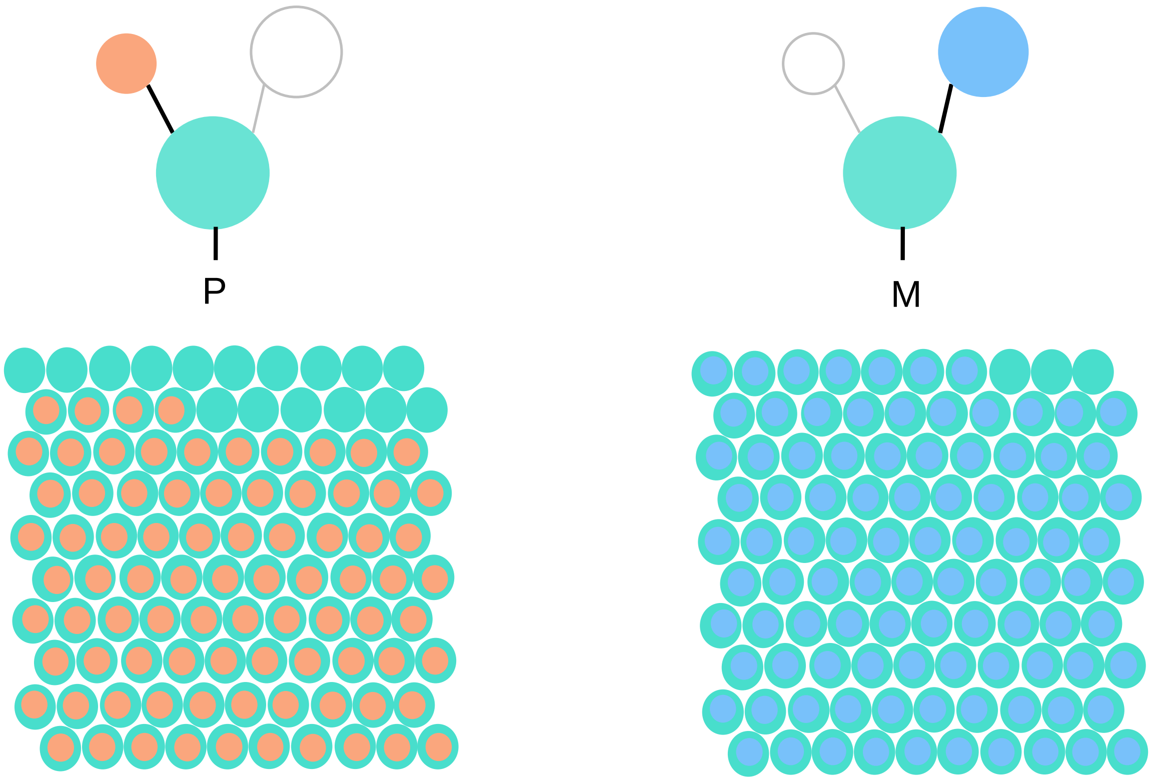
P
M

CRC-141

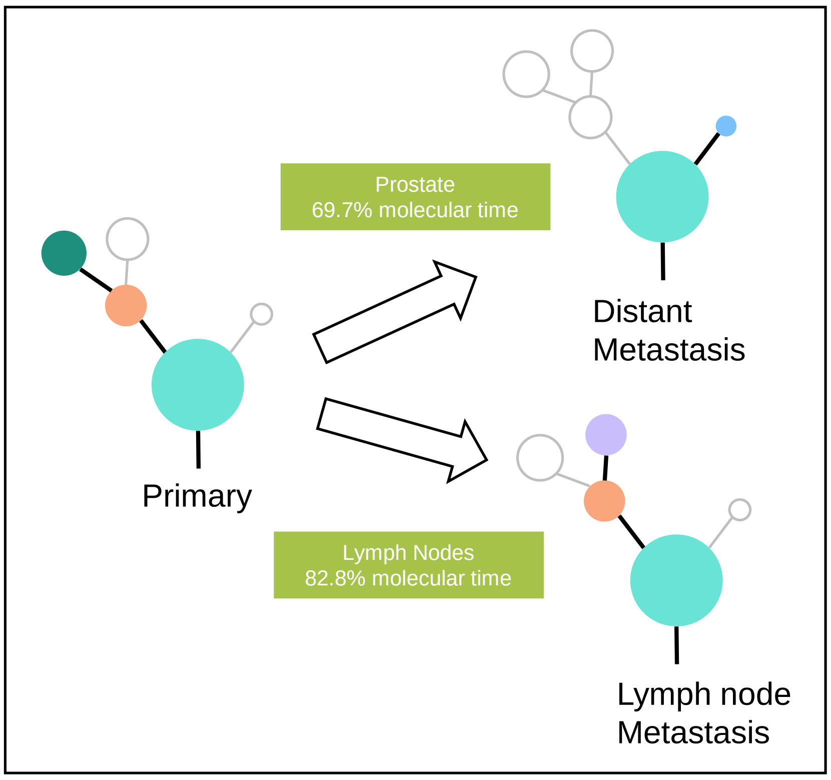
Prostate
69.7% molecular time
Distant
Metastasis
Primary
Lymph Nodes
82.8% molecular time
Lymph node
Metastasis

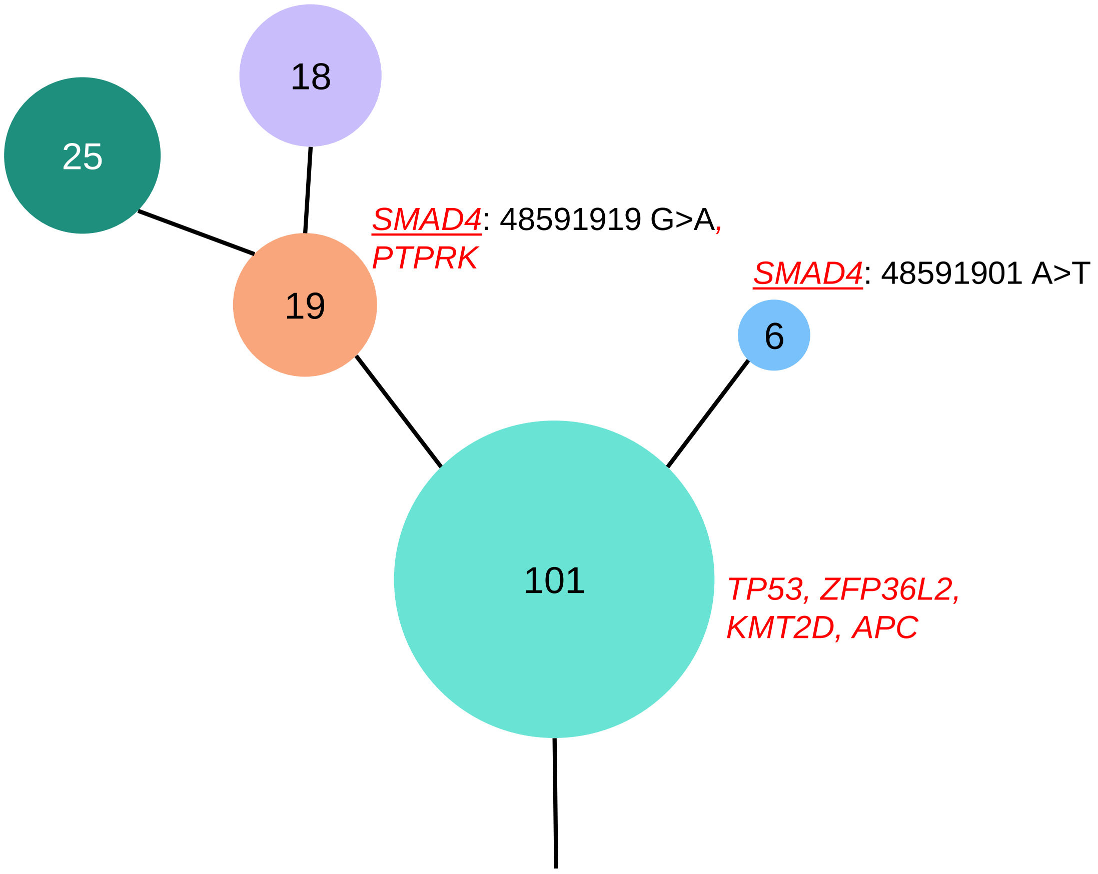
18
25
SMAD4: 48591919 G>A,
PTPRK
19
SMAD4: 48591901 A>T
6
101
TP53, ZFP36L2,
KMT2D, APC

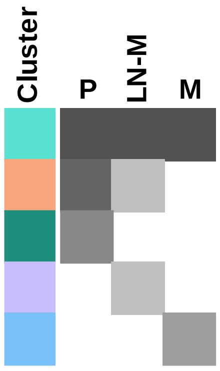
Cluster
P
LN-M
M

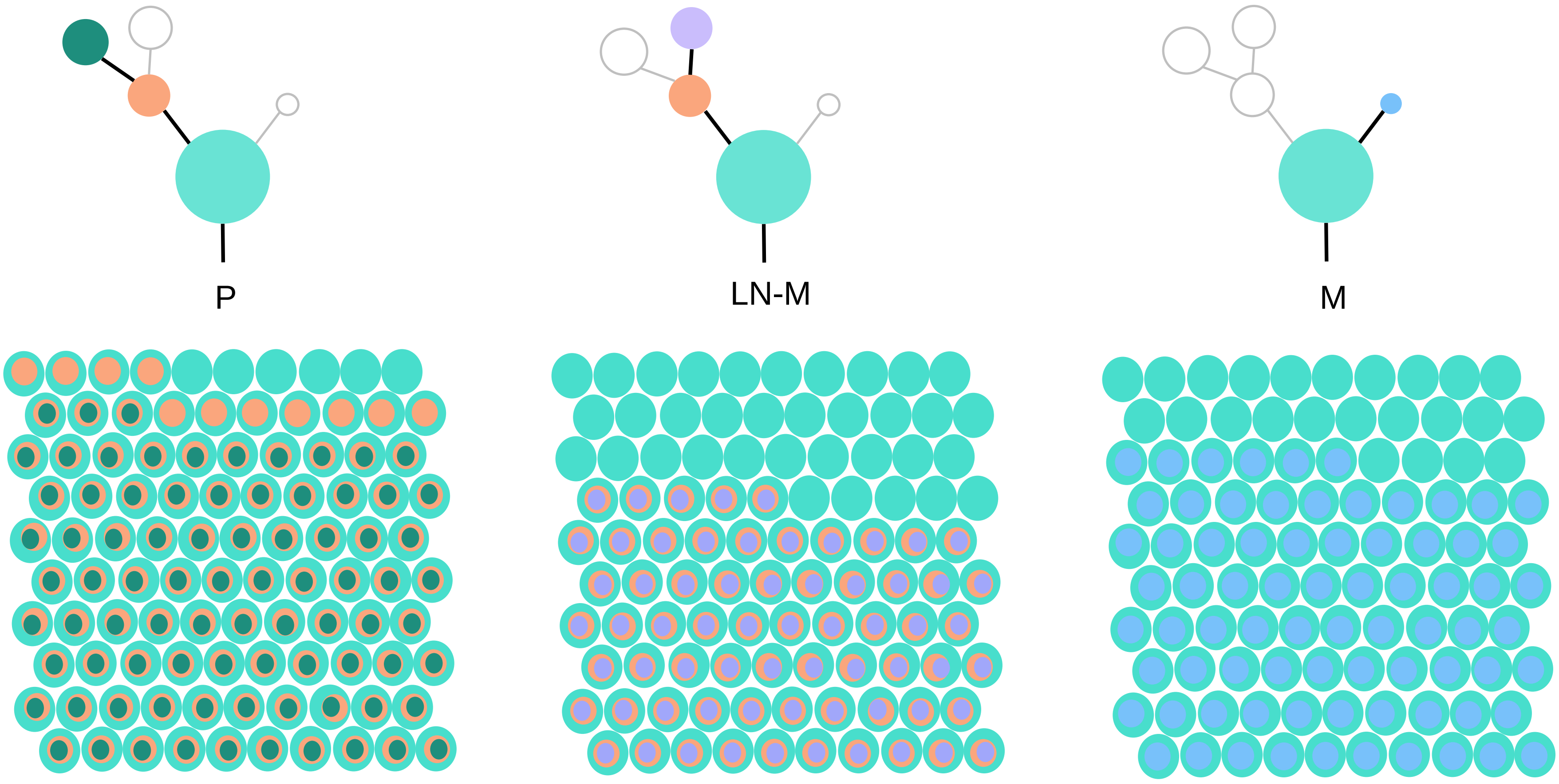
P
LN-M
M

CRC-190

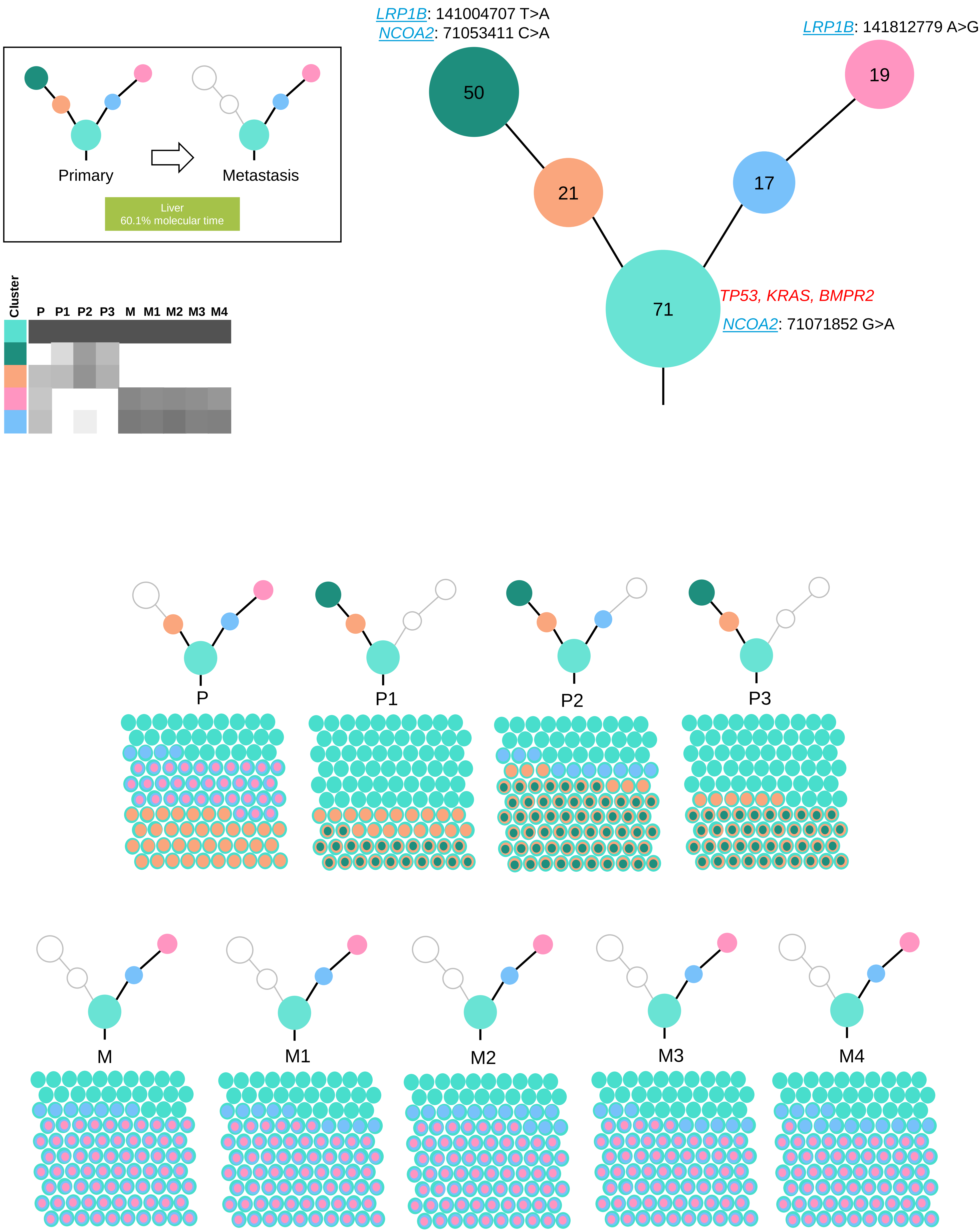

Primary
Metastasis
Liver
60.1% molecular time
Cluster
P P1 P2 P3 M M1 M2 M3 M4
LRP1B: 141004707 T>A
NCOA2: 71053411 C>A
LRP1B: 141812779 A>G
50
19
21
17
71
TP53, KRAS, BMPR2
NCOA2: 71071852 G>A
P
P1
P2
P3
M
M1
M2
M3
M4

CRC-233

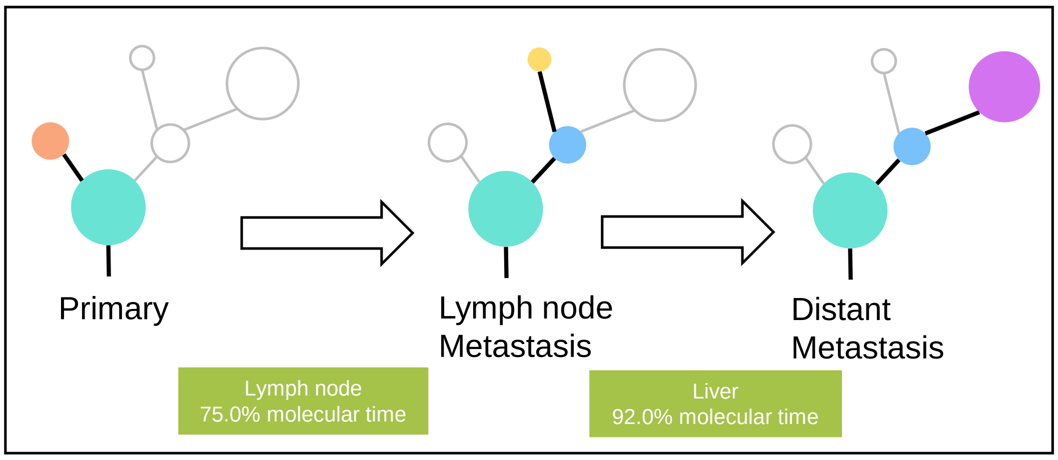

Primary
Lymph node Metastasis
Distant Metastasis
Lymph node
75.0% molecular time
Liver
92.0% molecular time

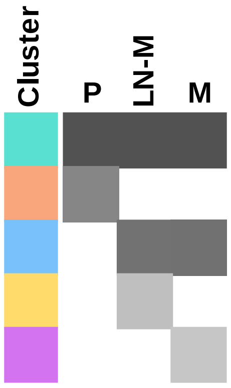

Cluster
P
LN-M
M

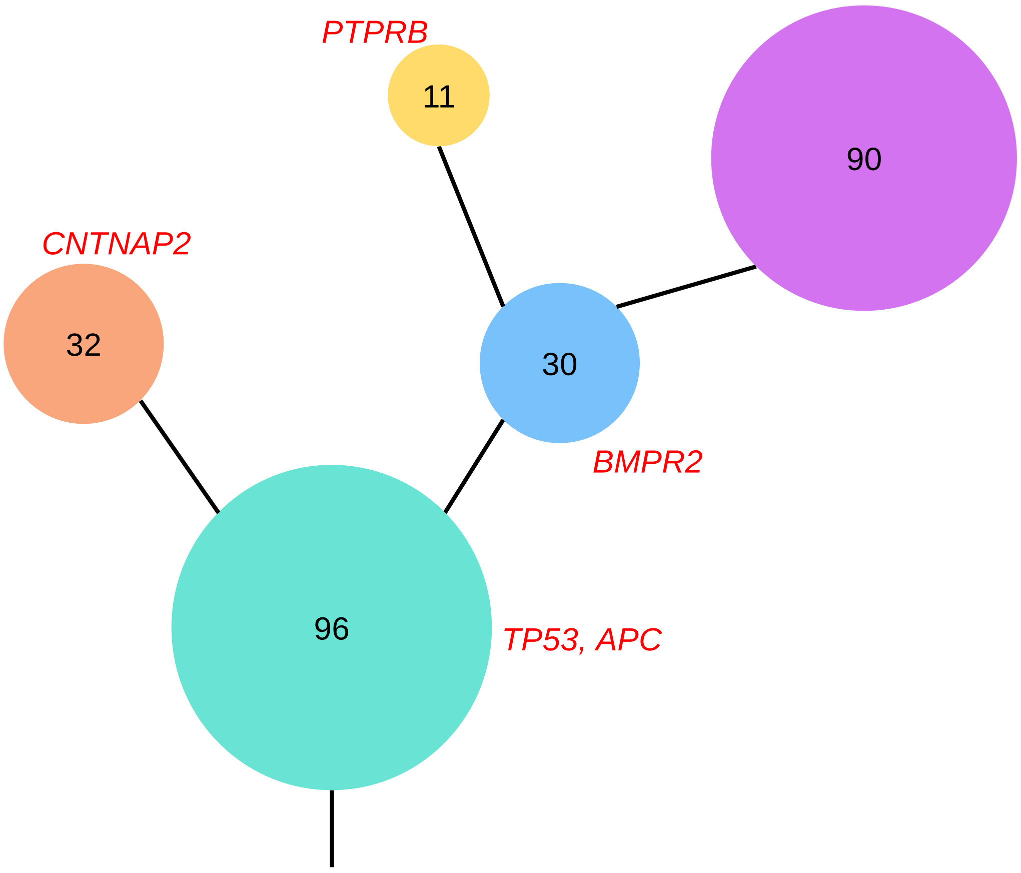

PTPRB
11
90
CNTNAP2
32
30
BMPR2
96
TP53, APC

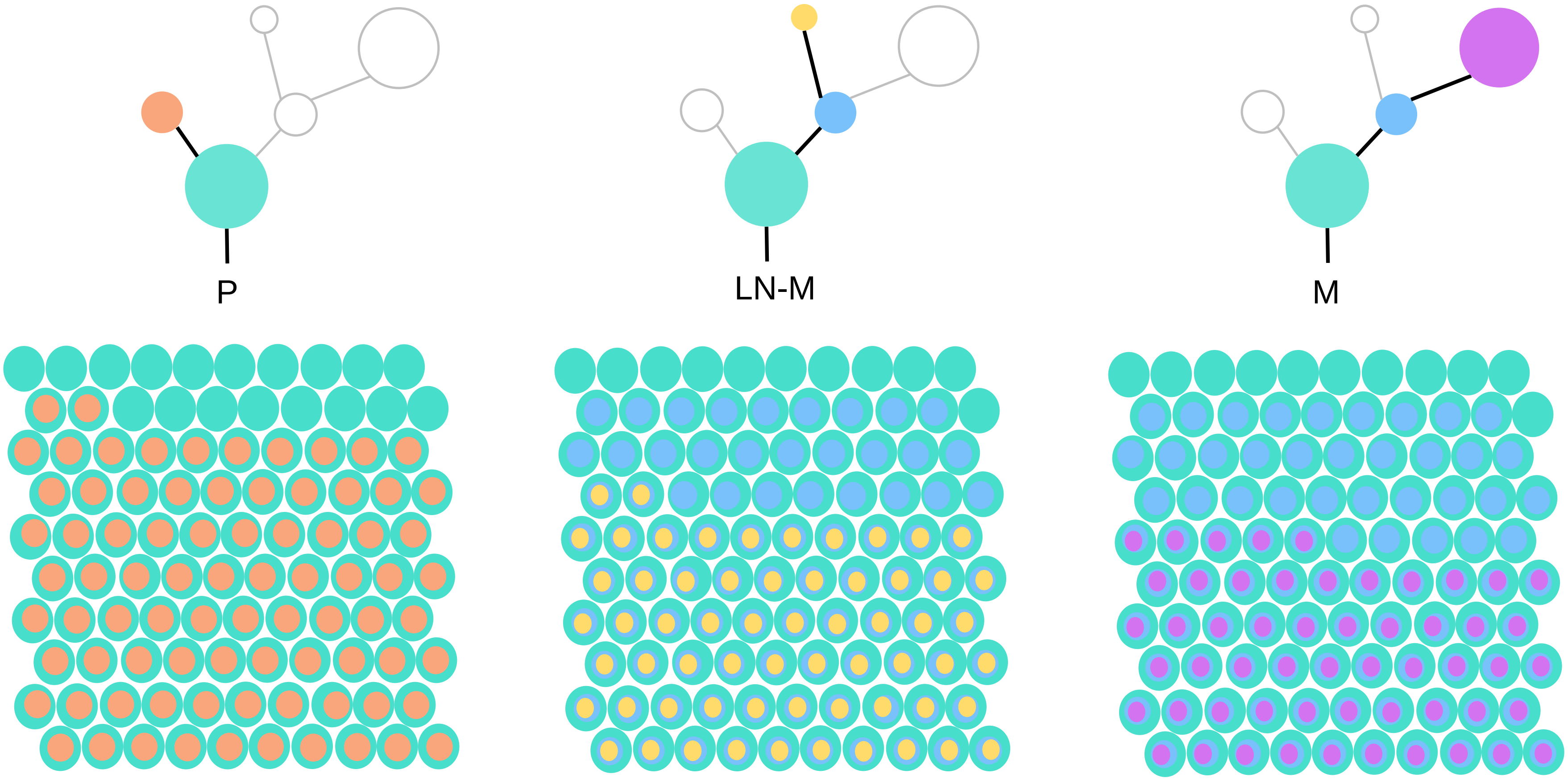

P
LN-M
M

CRC-293

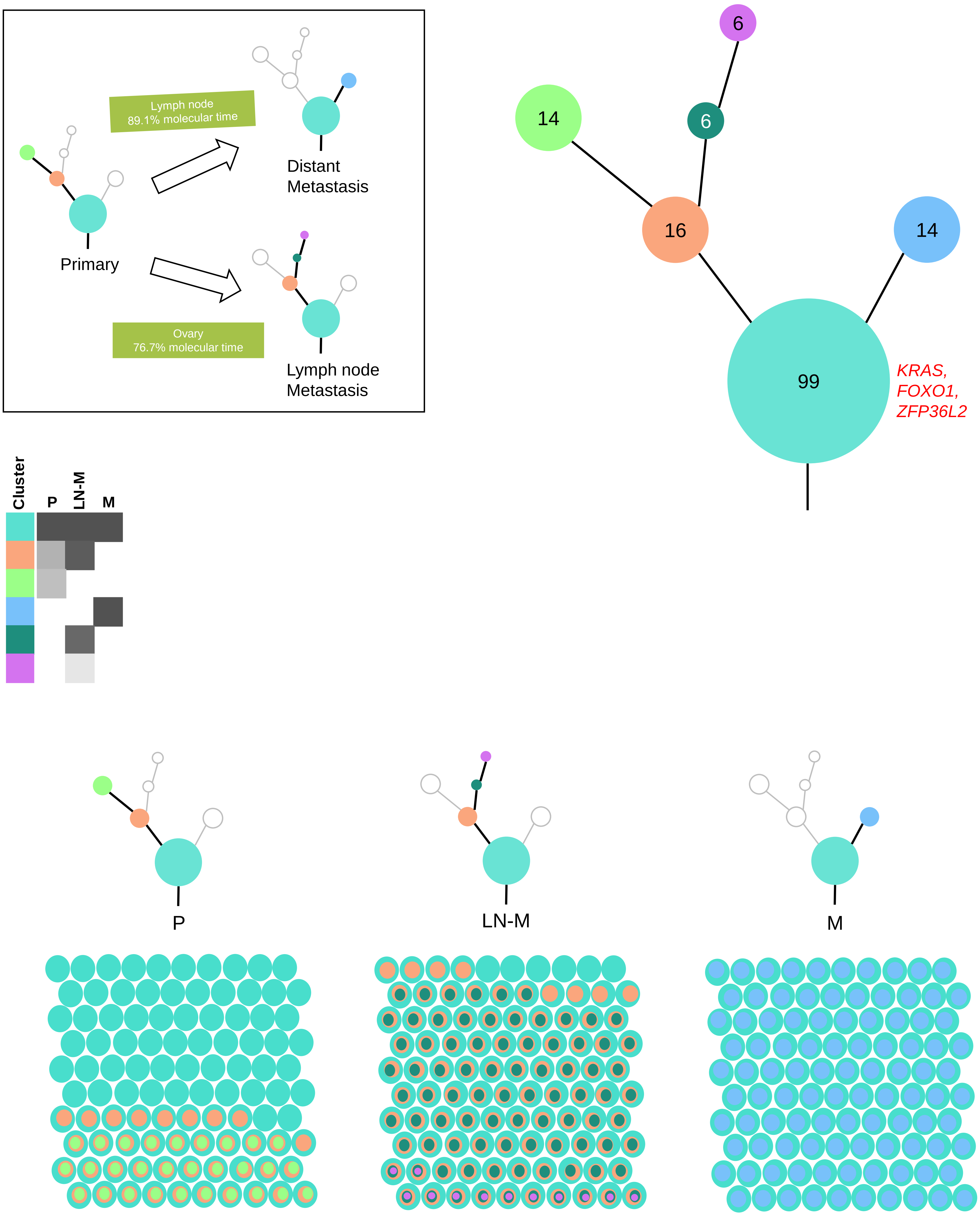
Lymph node
89.1% molecular time
Distant
Metastasis
Primary
Ovary
76.7% molecular time
Lymph node
Metastasis
6
14
6
16
14
99
KRAS,
FOXO1,
ZFP36L2
Cluster
P
LN-M
M
P
LN-M
M

CRC-320

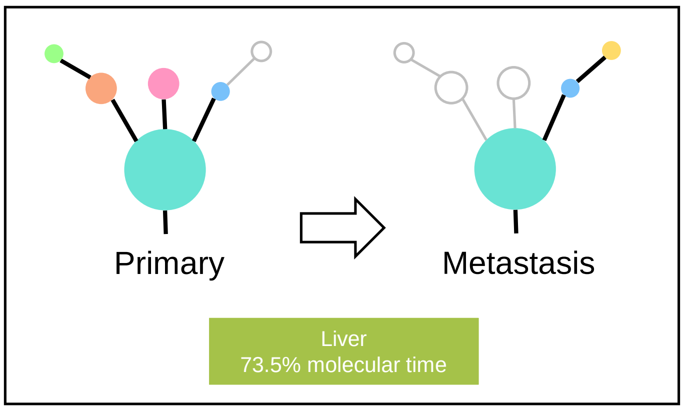
Primary
Metastasis
Liver
73.5% molecular time

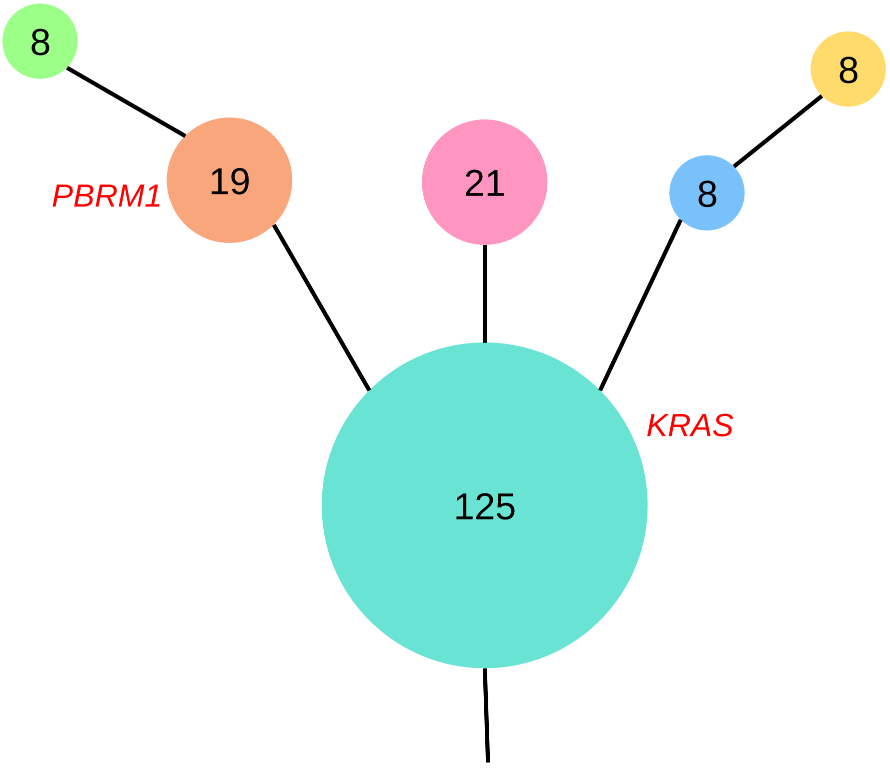
8
19
PBRM1
21
8
8
KRAS
125

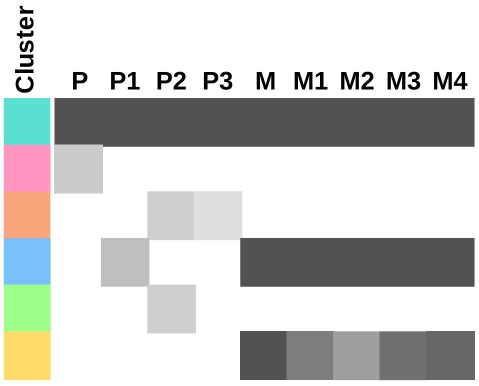
Cluster
P
P1
P2
P3
M
M1
M2
M3
M4

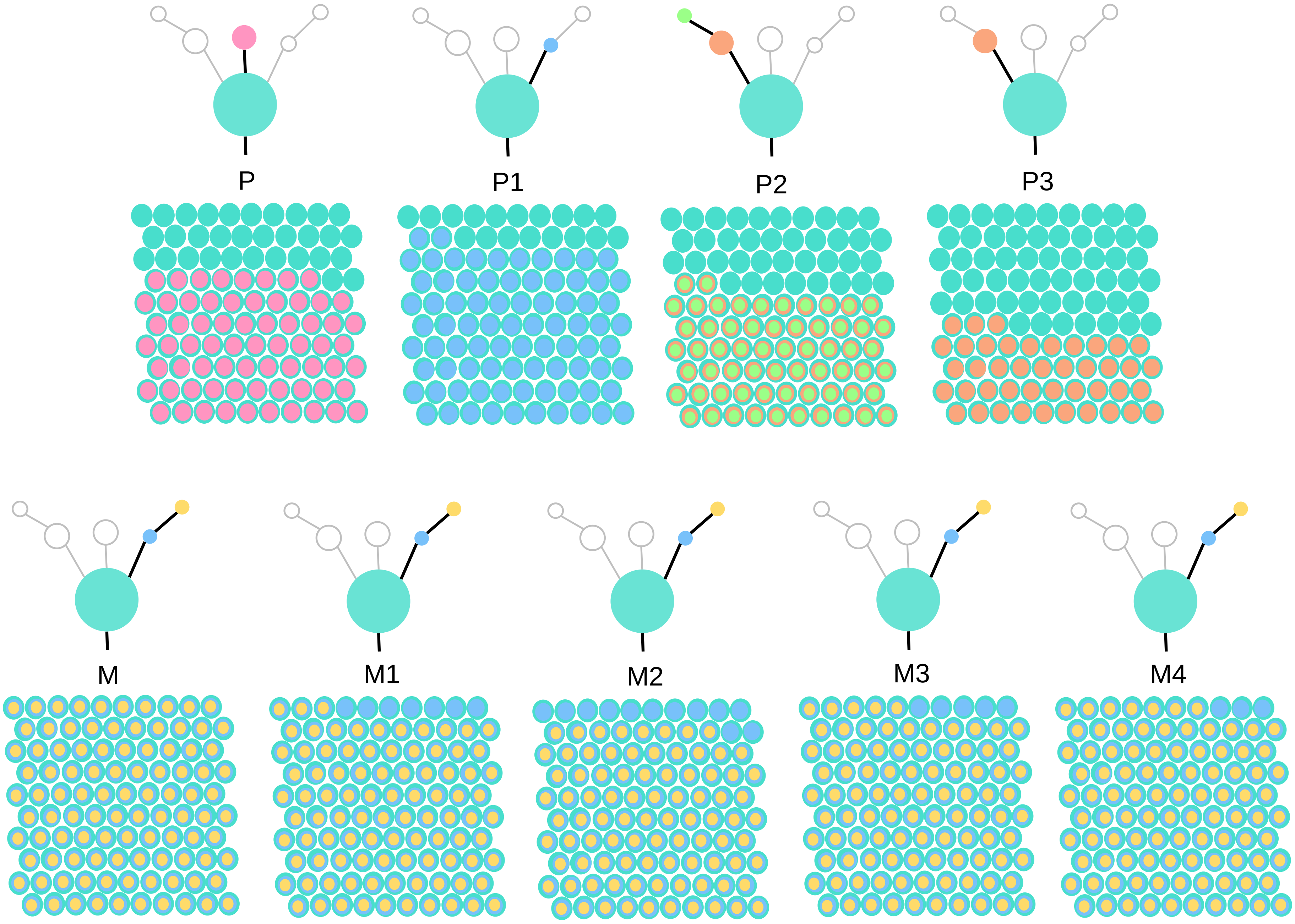
P
P1
P2
P3
M
M1
M2
M3
M4

CRC-363

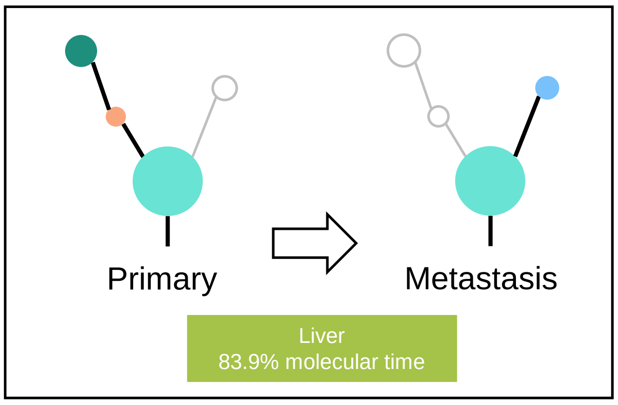
Primary
Metastasis
Liver
83.9% molecular time

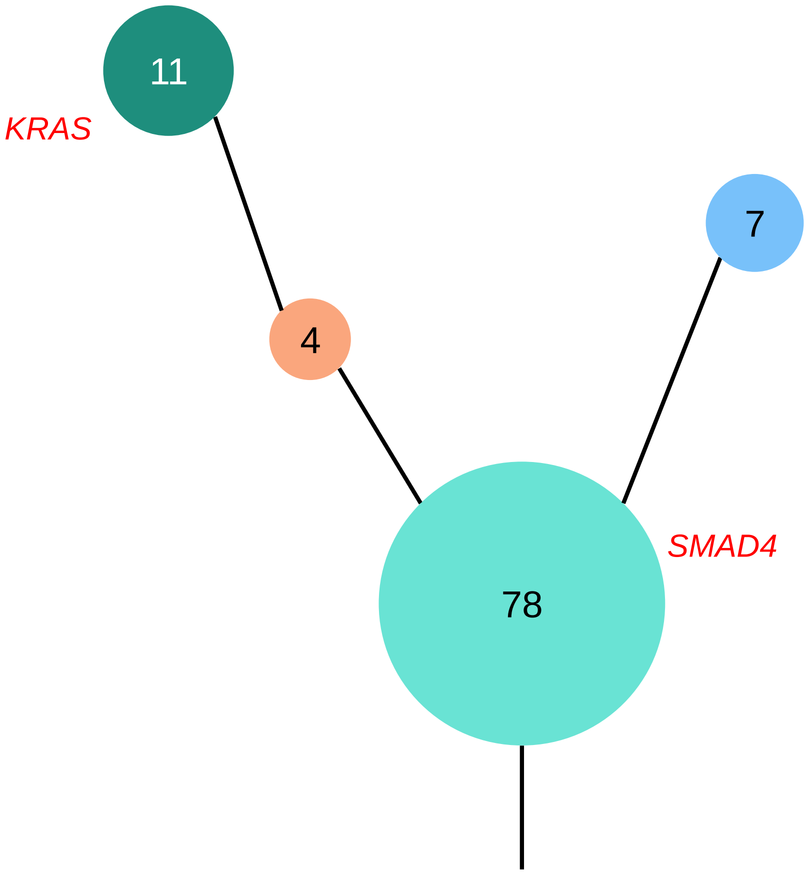
11
KRAS
4
7
78
SMAD4

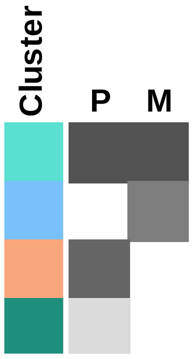
Cluster
P
M

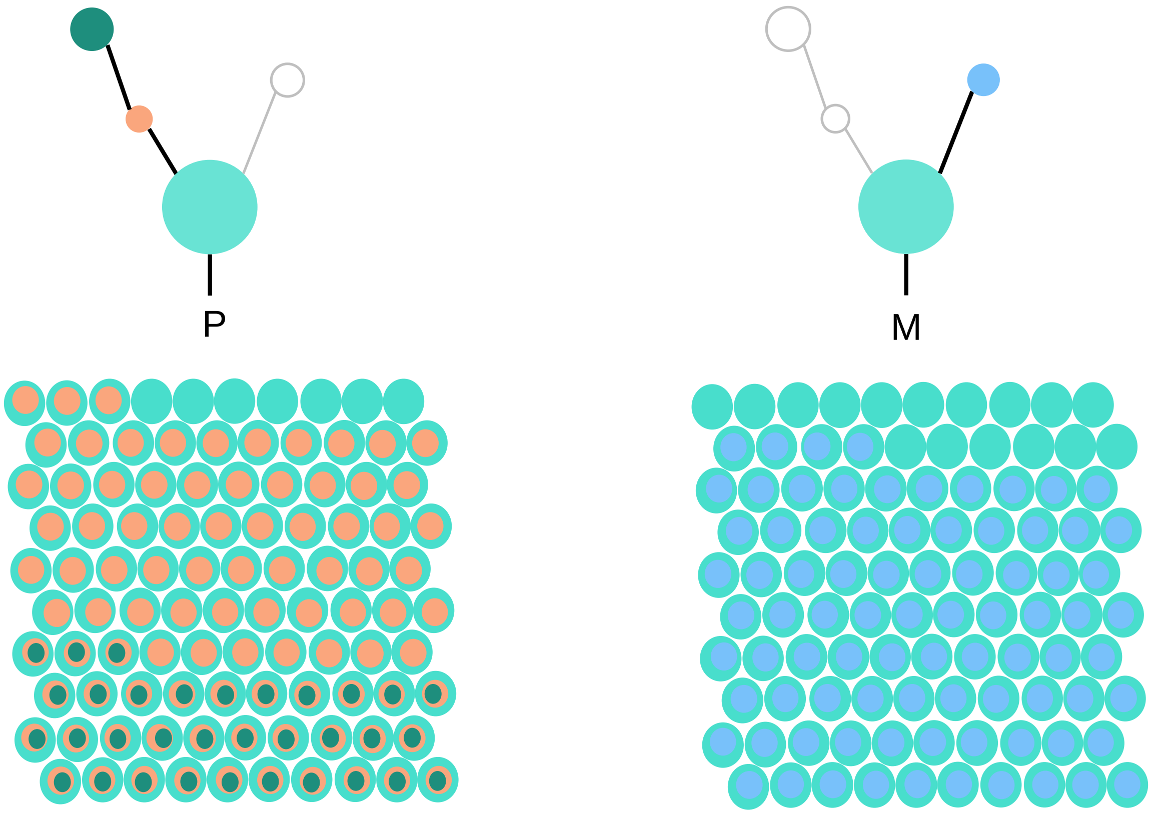
P
M

CRC-443

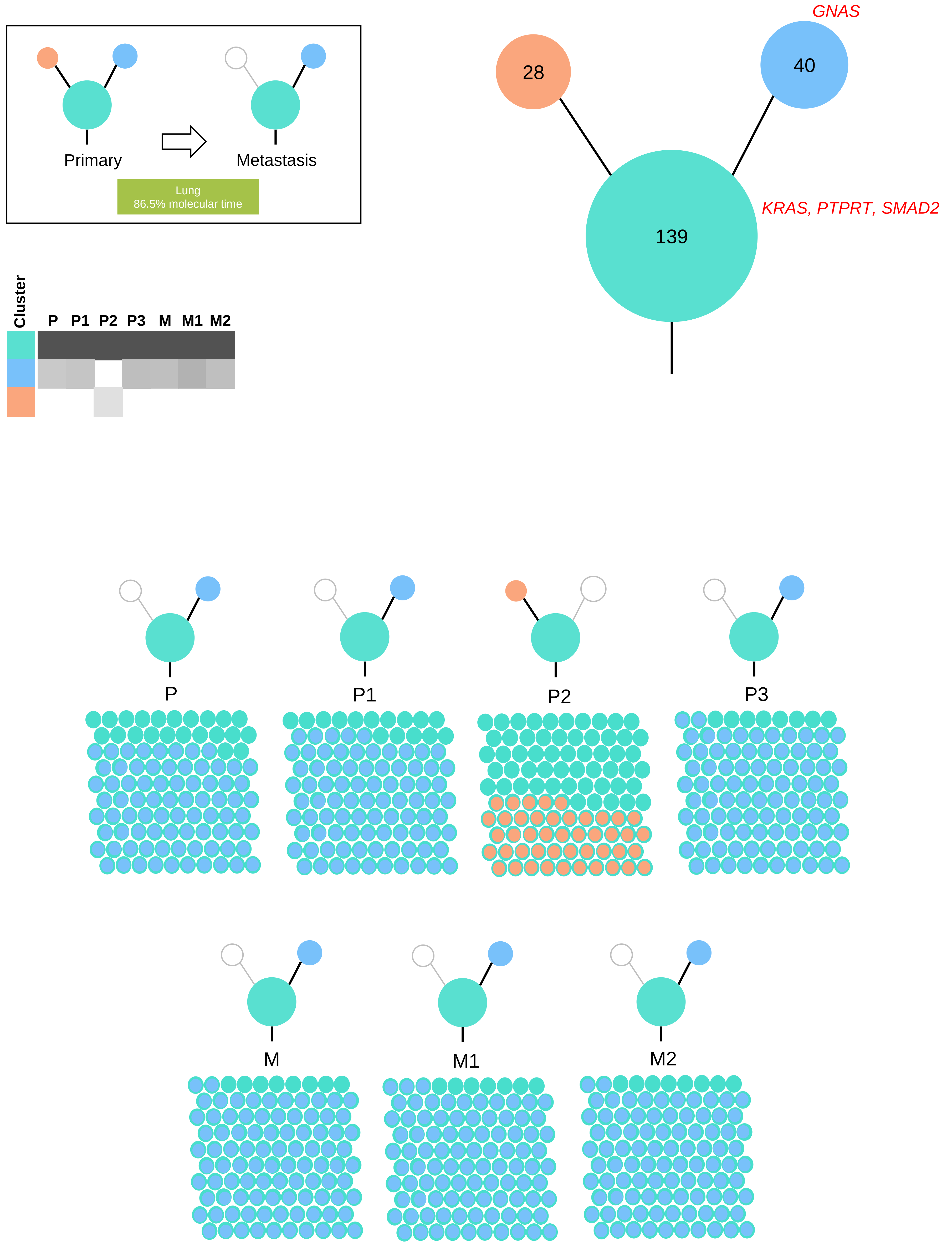
Primary
Metastasis
Lung
86.5% molecular time
Cluster
P
P1
P2
P3
M
M1
M2
GNAS
28
40
139
KRAS, PTPRT, SMAD2
P
P1
P2
P3
M
M1
M2

CRC-449

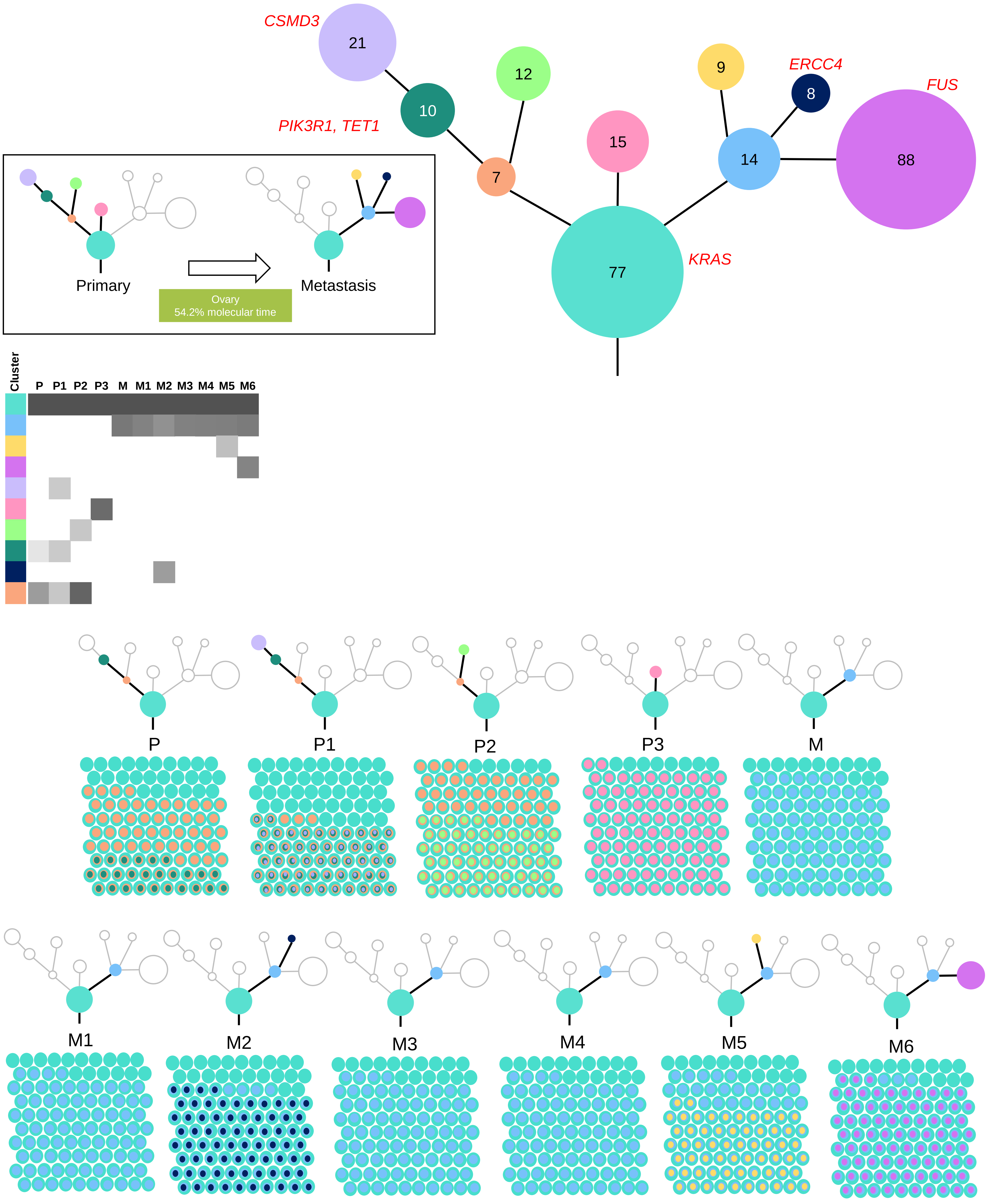
CSMD3
21
12
9
ERCC4
8
FUS
10
PIK3R1, TET1
15
7
14
88
77
KRAS
Primary
Metastasis
Ovary
54.2% molecular time
Cluster
P P1 P2 P3 M M1 M2 M3 M4 M5 M6
P
P1
P2
P3
M
M1
M2
M3
M4
M5
M6

CRC-453

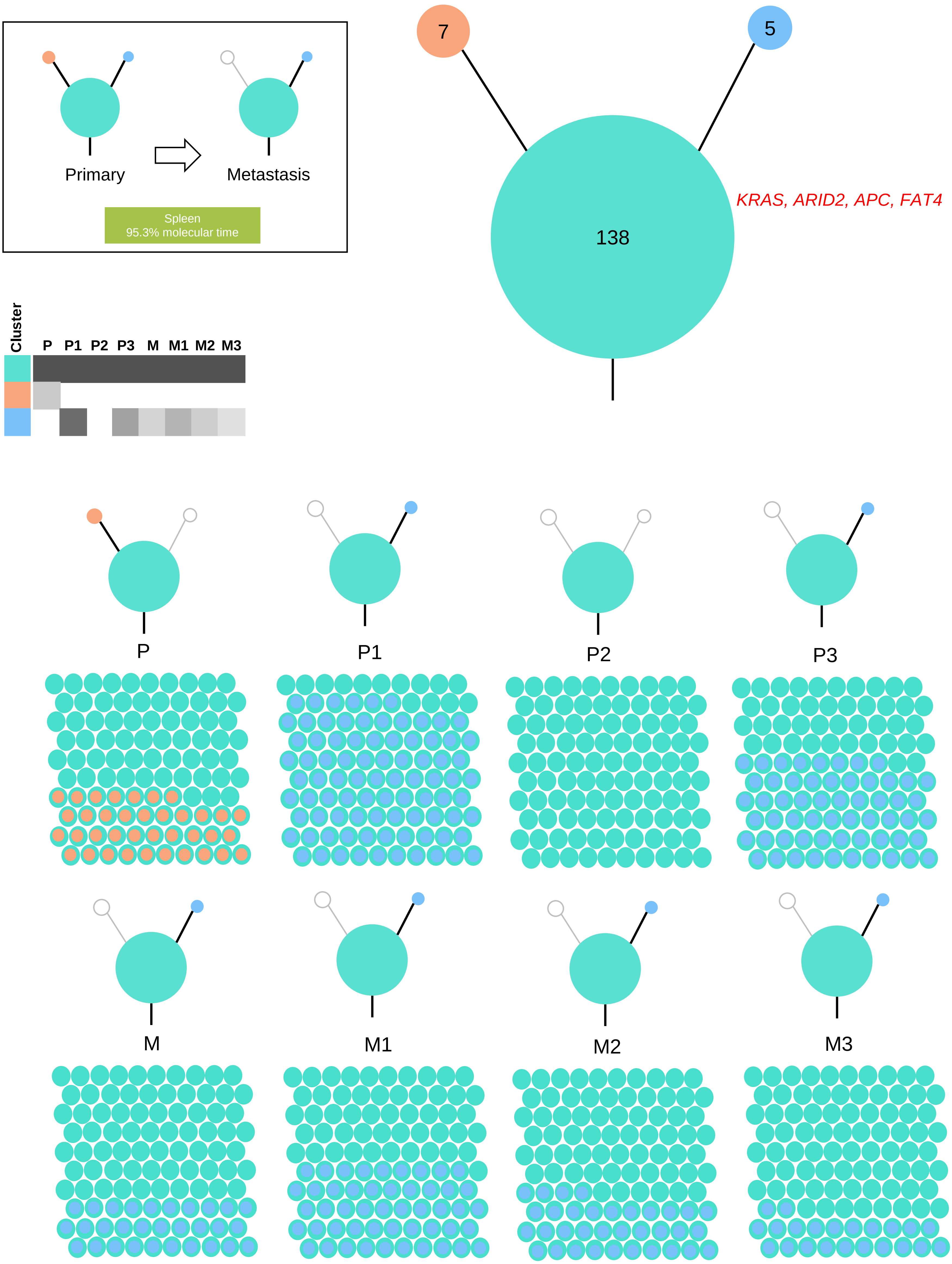
Primary
Metastasis
Spleen
95.3% molecular time
7
5
138
KRAS, ARID2, APC, FAT4
Cluster
P P1 P2 P3 M M1 M2 M3
P
P1
P2
P3
M
M1
M2
M3

CRC-468

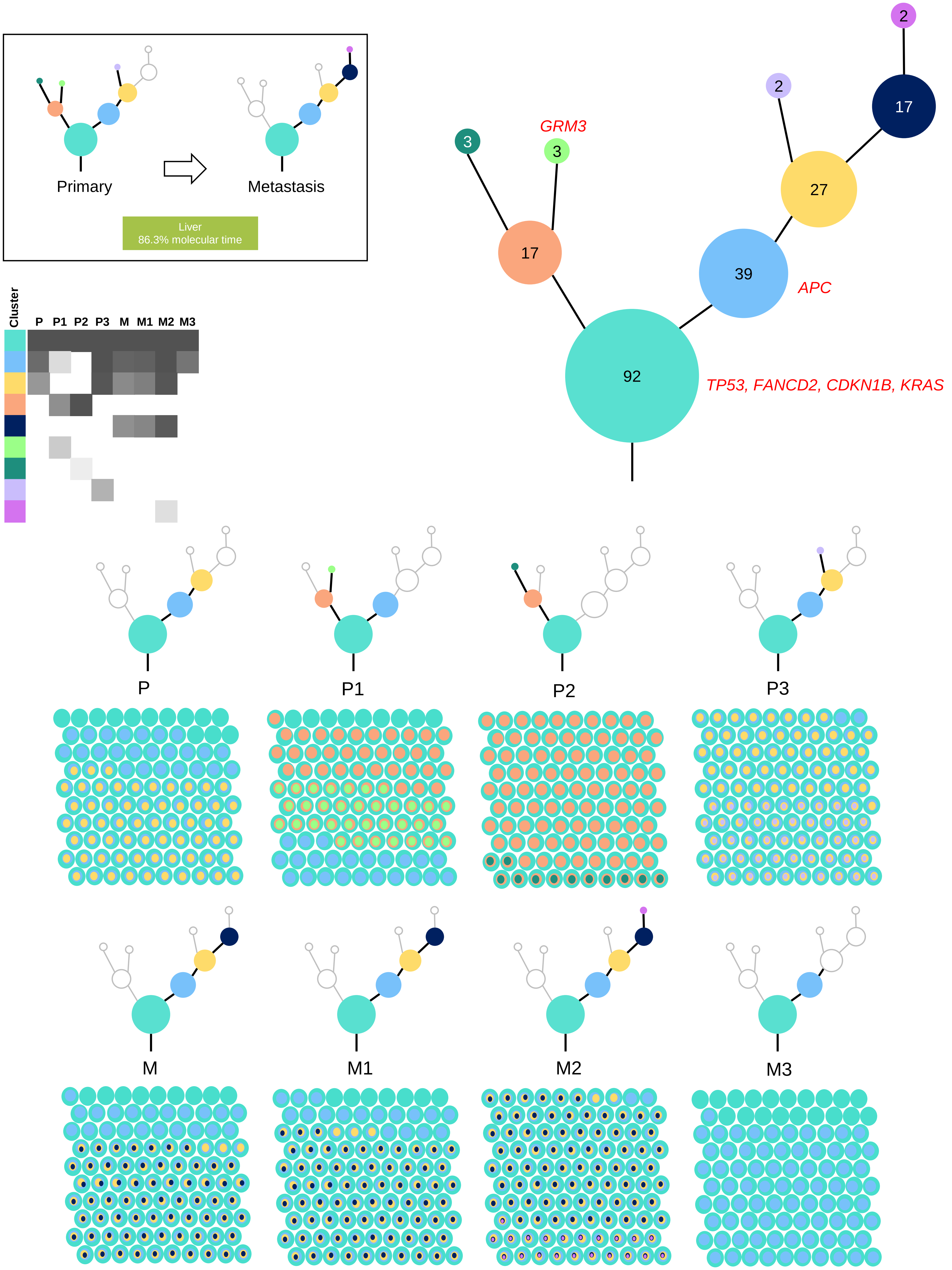
Primary
Metastasis
Liver
86.3% molecular time
Cluster
P
P1
P2
P3
M
M1
M2
M3
GRM3
APC
TP53, FANCD2, CDKN1B, KRAS
92
39
27
17
17
3
3
2
2

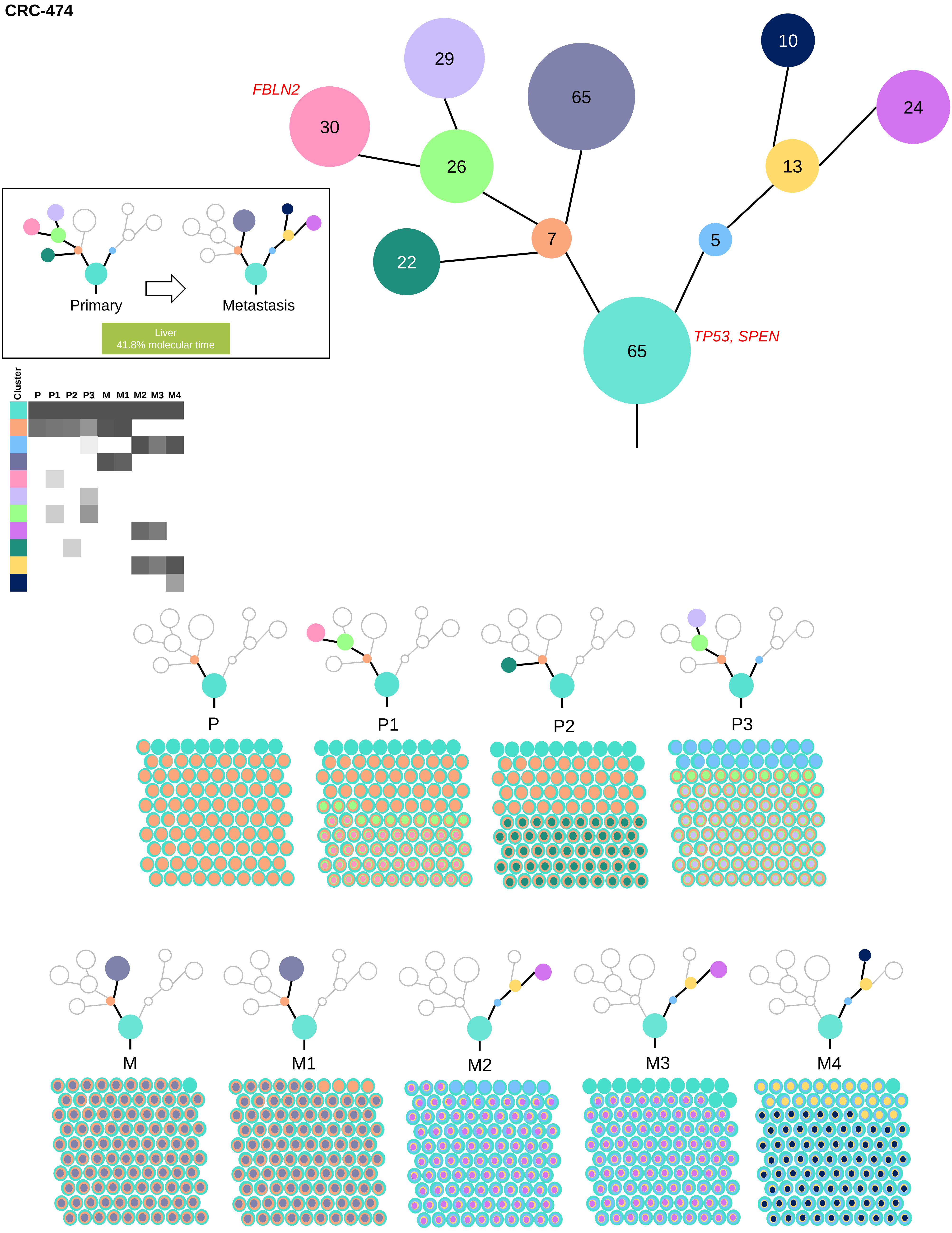
CRC-474
FBLN2
TP53, SPEN
30
29
26
65
7
22
65
5
13
10
24
Primary
Metastasis
Liver
41.8% molecular time
Cluster
P P1 P2 P3 M M1 M2 M3 M4
P
P1
P2
P3
M
M1
M2
M3
M4

CRC-477

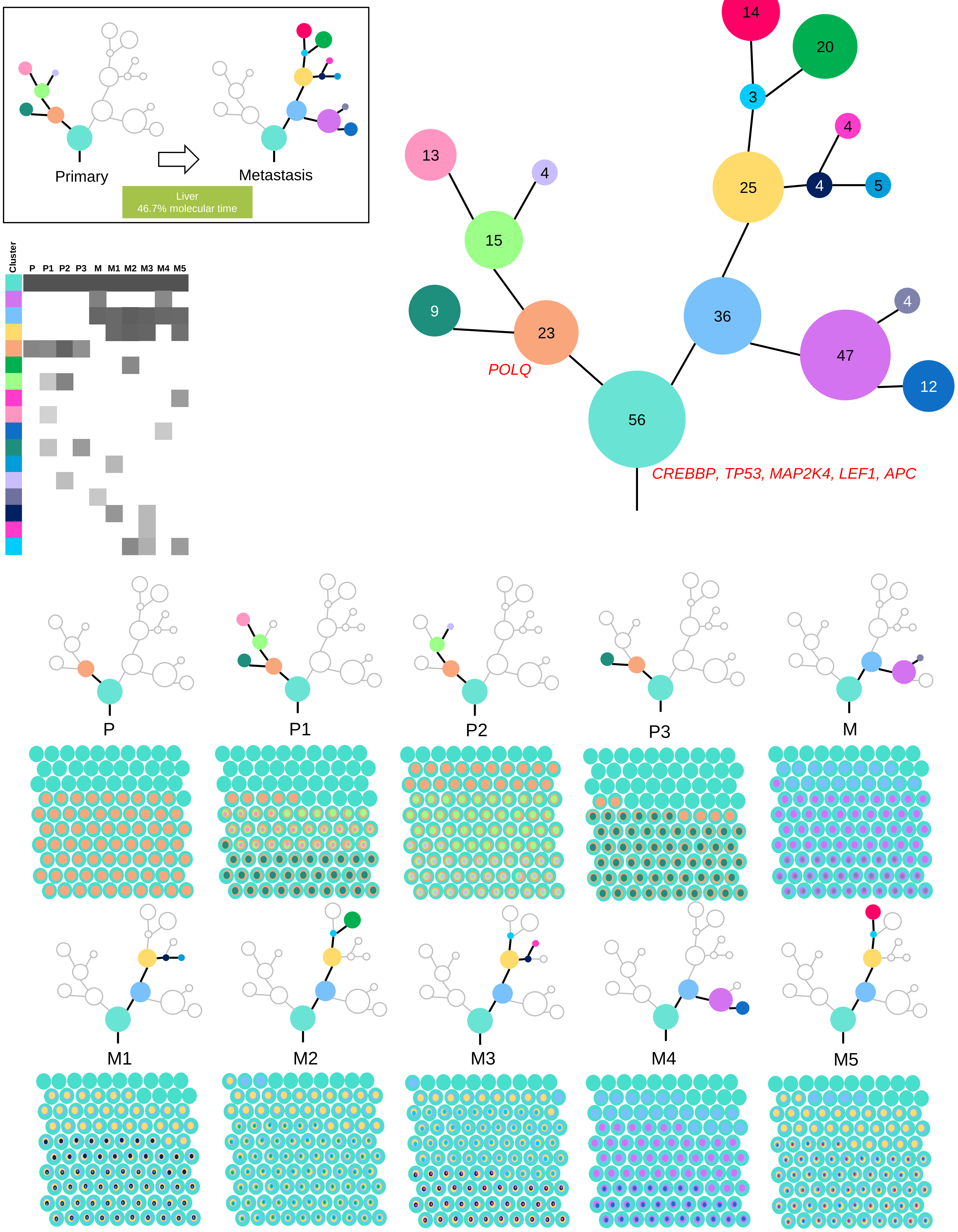
Primary
Metastasis
Liver
46.7% molecular time
Cluster
P P1 P2 P3 M M1 M2 M3 M4 M5
14
20
3
4
13
4
25
4
5
15
9
23
36
4
47
12
56
POLQ
CREBBP, TP53, MAP2K4, LEF1, APC
P
P1
P2
P3
M
M1
M2
M3
M4
M5

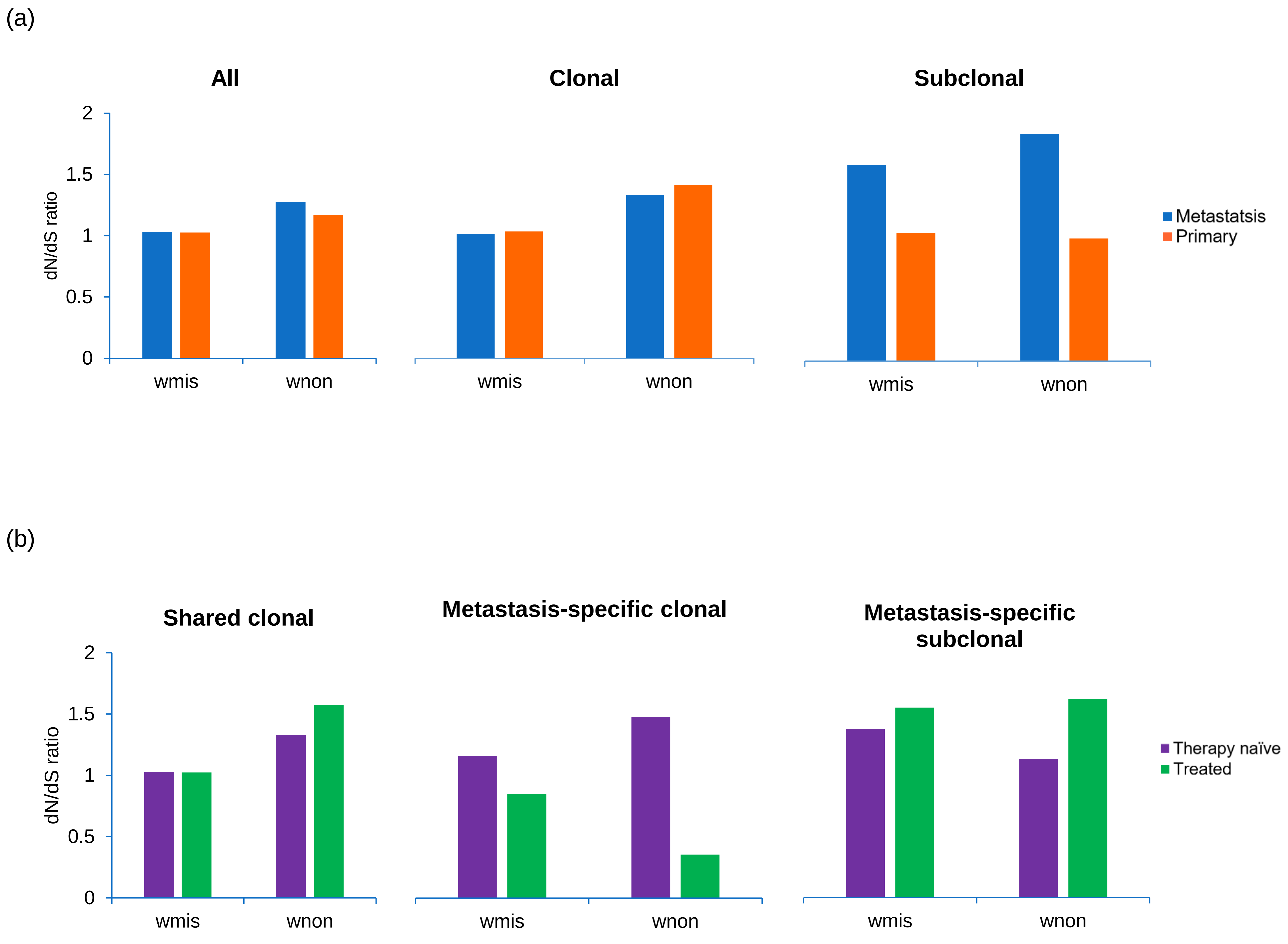

Figure S5: Selection in primary CRC and metastatic tumour lesions. Selection using ratios of substitution rate at nonsynonymous sites to those at synonymous sites (dN/dS), in (a) all, clonal and subclonal nonsense and missense mutations in primary CRC tumours and metastatic lesions; and in (b) shared clonal, metastasis-specific clonal and subclonal nonsense and missense mutations in therapy-naïve and treated tumours.
